# Photoinduced Triphenylphosphine and Iodide Salt Promoted Reductive Decarboxylative Coupling

**DOI:** 10.1002/advs.202307241

**Published:** 2024-01-17

**Authors:** Jia‐Xin Wang, Ming‐Chen Fu, Lu‐Yu Yan, Xi Lu, Yao Fu

**Affiliations:** ^1^ Hefei National Research Center for Physical Sciences at the Microscale iChEM CAS Key Laboratory of Urban Pollutant Conversion Anhui Province Key Laboratory of Biomass Clean Energy University of Science and Technology of China Hefei 230026 China; ^2^ School of Chemistry and Chemical Engineering Hefei University of Technology Hefei 230009 China; ^3^ Key Laboratory of Precision and Intelligent Chemistry Department of Applied Chemistry School of Chemistry and Materials Science University of Science and Technology of China Hefei 230026 China

**Keywords:** divergent synthesis, electron donor–acceptor complex, photochemical reduction, photoinduced decarboxylative coupling, photoinduced organic synthesis

## Abstract

The transient electron donor–acceptor (EDA) complex has been an emerging area in the photoinduced organic synthesis field, generating radicals without exogenous transition‐metal or organic dye‐based photoredox catalysts. The catalytic platform to form suitable photoactive EDA complexes for photochemical reduction reactions remains underdeveloped. Herein, a general photoinduced reductive alkylation via the EDA complex strategy is described. A simple yet multifunctional system, triphenylphosphine and iodide salt, promotes the photoinduced decarboxylative hydroalkylation, and reductive defluorinative decarboxylative alkylation of trifluoromethyl alkenes, to access trifluoromethyl alkanes and *gem*‐difluoroalkenes. Moreover, decarboxylative hydroalkylation can be applied to more kinds of electron‐deficient alkenes as a general Giese addition reaction.

## Graphical abstract

Photoinduced reductive alkylation via a transient electron donor–acceptor (EDA) complex strategy is reported. A simple yet multifunctional system, triphenylphosphine and iodide salt, enables the divergent synthesis of trifluoromethyl alkanes and *gem*‐difluoroalkenes through decarboxylative hydroalkylation and reductive decarboxylative defluorinative alkylation between redox‐active esters with α‐CF_3_‐styrenes.

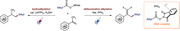

## 1 Introduction

**Figure 1:**
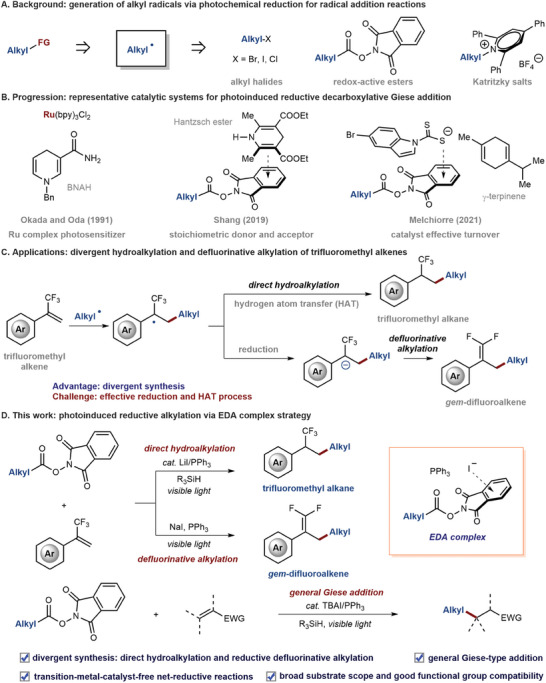
Photoinduced reductive alkylation via an EDA complex strategy. FG, functional group; bpy, 2,2′‐bipyridine; Bn, benzyl; EWG, electron‐withdrawing group; TBAI, tetrabutylammonium iodide.

C(sp^3^) centers are commonly found in organic molecules and make these compounds in three‐dimensional structures.^[^ ^1^ ^]^ Carbon–carbon and carbon–heteroatom bond formation reactions have significant impacts on complex molecular synthesis. The generation of alkyl radicals from radical precursors via the photochemical reduction process under mild conditions provides fascinating solutions to construct carbon–carbon and carbon–heteroatom bonds (**Figure** **1**).^[^ ^2^ ^]^ Among them, redox‐active esters (*N*‐hydroxyphthalimide esters, RAEs), which are stable and can be easily prepared from the corresponding carboxylic acids, have been extensively explored (Figure 1A).^[^ ^3^ ^]^ The well‐known Giese radical addition reaction forms carbon–carbon bonds through the addition of carbon‐centered radicals to alkenes, commonly electron‐deficient alkenes.^[^ ^4^ ^]^ Successive efforts have been devoted to the development of efficient catalytic systems for photoinduced decarboxylative Giese addition (Figure 1B).^[^ ^5^ ^]^ For example, Okada and Oda conducted a pioneering study in photoinduced decarboxylative Giese radical addition reaction of *N*‐hydroxyphthalimide ester using ruthenium complex as the photosensitizer.^[^ ^6^ ^]^ More recently, the transient electron donor–acceptor (EDA) complex has been the emerging area in the photoinduced organic synthesis field, generating radicals without exogenous transition‐metal or organic dye‐based photoredox catalysts.^[^ ^7^ ^]^ For example, Shang reported that the EDA complexes, aggregated by the mix of stoichiometric Hantzsch ester and *N*‐hydroxyphthalimide ester, could participate in the decarboxylative conjugated addition.^[^ ^8^ ^]^ Melchiorre and co‐workers made a breakthrough in developing a sulfur anion‐based organic catalyst, that enables the formation of a photoactive EDA complex to convert redox‐active esters into corresponding alkyl radicals to undergo photochemical reduction reactions.^[^ ^9^ ^]^ Despite these achievements, a catalytic platform, forming a suitable photoactive EDA complex with redox‐active esters, to enable photochemical reductive decarboxylation remains highly required.

On the other hand, fluorinated organic molecules have broad applications in the pharmaceutical, agrochemical, and material industries.^[^ ^10^ ^]^ Among the fluorine‐containing motifs, trifluoromethyl alkanes and *gem*‐difluoroalkenes are of particular importance and have attracted much attention in the modification of bioactive molecules.^[^ ^11^ ^]^ In recent years, progress has been made in the facile conversion of readily available α‐CF_3_‐styrenes to *gem*‐difluoroalkenes^[^ ^12^ ^]^ and trifluoromethyl alkanes.^[^ ^13^ ^]^ However, Giese‐type hydroalkylation to access trifluoromethyl alkanes and defluorinative alkylation to *gem*‐difluoroalkenes are two competing processes.^[^ ^14^ ^]^ Exploring simple and efficient methods for divergent alkylation of readily available α‐CF_3_‐styrenes using a multifunctional system, especially using a transition‐metal‐free condition, remains challenging.

Our recent works revealed that iodide anion and triphenylphosphine in combination with RAEs would form an EDA complex, which absorbs visible light to deliver alkyl radicals.^[^ ^15^ ^]^ This strategy has successful applications in several redox‐neutral reactions but is incapable of a photochemical reduction process. We envisaged that with the addition of a proper reductant, the NaI/PPh_3_ system might be expanded to photochemical reductive couplings. For example, the alkyl radical, generated from the EDA complex assembled by iodide salts and RAEs, would smoothly occur radical addition with trifluoromethyl alkanes to give α‐CF_3_ carbon radicals. Then, the divergent alkylation of trifluoromethyl alkenes might be achieved by fine‐tuning reaction conditions, selectively undergoing the hydrogen atom transfer (HAT) process or single‐electron‐transfer (SET) coupled *β*‐F elimination process, respectively (Figure 1C).

Herein, we describe a general photoinduced reductive alkylation via an EDA complex strategy (Figure 1D). A simple yet multifunctional system, triphenylphosphine and iodide salt, promotes the photoinduced decarboxylative hydroalkylation and reductive defluorinative decarboxylative alkylation of trifluoromethyl alkenes. Various trifluoromethyl alkanes and *gem*‐difluoroalkenes are obtained with high chemoselectivity and excellent functional group tolerance. Mechanistic investigations reveal that hydrosilane serves as both hydrogen atom transfer reagent and single‐electron reductant in decarboxylative hydroalkylation reaction, while triphenylphosphine suppresses the electron back transfer and thus serves as a single‐electron reductant in reductive decarboxylative and defluorinative cross‐coupling. Moreover, decarboxylative hydroalkylation is applied to more kinds of electron‐deficient alkenes to realize a general Giese‐type addition reaction.

## 2 Results and Discussion

### 2.1 Screening of Reaction Conditions

As shown in **Table** **1**, we selected the α‐CF_3_‐styrene **1** and alkyl radical precursor **2** as the model substrates. After a comprehensive investigation of all reaction parameters (see Supporting Information for details), we found that the mixture of LiI (20 mol %), PPh_3_ (20 mol %), and (TMS)_3_SiH (tris(trimethylsilyl)silane, 1.5 equiv.) in tetrahydrofuran (THF, 0.1 mol/L) succeeded for the decarboxylative hydroalkylation, irradiated by violet LEDs (427 nm) at room temperature. The decarboxylative hydroalkylation product **3** was obtained in a 95% ^19^F NMR (nuclear magnetic resonance) yield and a 93% isolated yield (entry 1), without the observation of any reductive defluorinative product **4**. Different solvents showed significant influences on the reaction outcomes. The yield of **3** was much lower when using CH_3_CN, CH_2_Cl_2_, or dimethyl sulfoxide (DMSO) as the solvent (entries 2–4). The reaction was completely shut down when using amide solvents, such as *N*,*N*‐dimethylformamide (DMF) and *N*,*N*‐dimethylacetamide (DMA) (entries 5–6). We also texted other hydrosilanes, such as Ph_2_MeSiH, Ph_3_SiH, and Et_3_SiH, but failed to deliver the desired product **3** (see Table S2, Supporting Information). Interestingly, 1,4‐cyclohexadiene could be used instead of (TMS)_3_SiH, and product **3** was detected in 23% yield (entry 7), indicating the involvement of a hydrogen atom transfer process. The yield of **3** dramatically decreased when reducing the amount of (TMS)_3_SiH to 1.0 equiv. (entry 8). The control experiment showed that PPh_3_ was unnecessary, which was theoretically plausible. But then again, a catalytic amount of PPh_3_ could significantly improve the reaction efficiency (entry 9). Besides, lithium iodide, hydrogen atom transfer reagent, and light irradiation were indispensable for the success of the decarboxylative hydroalkylation (entry 10). As for the reductive decarboxylative defluorinative alkylation process, the desired product **4** was formed in a 93% ^19^F NMR yield and with a 90% isolated yield, in the presence of NaI (0.5 equiv.) and PPh_3_ (1.0 equiv.) in DMA solvent with the irradiation of blue LEDs (440 nm) at room temperature (entry 11). The reaction was suppressed when using THF instead of DMA as the solvent (entry 12). Other solvents and iodide salts were also investigated (see Table S8 and S9, Supporting Information), but all exhibited lower efficiency than DMA and NaI. The yield of the desired product **4** decreased to 7% upon decreasing the amount of PPh_3_ to a 10 mol% catalytic amount (entry 13). As expected, product **4** was not observed without PPh_3_, NaI, or the light‐irradiation (entries 14–15). Furthermore, successful gram‐scale reactions illustrated the synthetic value of our methods for divergent conversion of α‐CF_3_‐styrenes.

**Table 1:** Optimization of the Reaction Conditions.

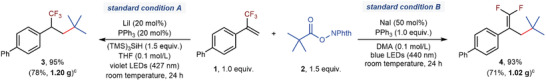			
Entry	Variation	Yield of 3 (%)	Yield of 4 (%)
**Variation from standard condition A** ^a)^			
1	none	95 (93^b)^)	0
2	CH_3_CN instead of THF	42	trace
3	CH_2_Cl_2_ instead of THF	8	15
4	DMSO instead of THF	13	7
5	DMA instead of THF	trace	0
6	DMF instead of THF	trace	0
7	1,4‐cyclohexadiene instead of (TMS)_3_SiH	23	0
8	(TMS)_3_SiH (1.0 equiv.)	64	0
9	without PPh_3_	42	0
10	without LiI, hydrogen atom transfer reagent, or light‐irradiation	0	0
**Variation from standard condition B** ^a)^			
11	none	0	93 (90^b)^)
12	THF instead of DMA	0	Trace
13	PPh_3_ (10 mol%)	0	7
14	no PPh_3_	0	0
15	without NaI, or light‐irradiation	0	0

^a)^ Reaction conditions: standard condition A, **1** (1.0 equiv.), **2** (1.5 equiv.), LiI (20 mol%), PPh_3_ (20 mol%), (TMS)_3_SiH (1.5 equiv.), THF (0.1 mol L^−1^), 427 nm violet LEDs, room temperature, 24 h; standard condition B, **1** (1.0 equiv.), **2** (1.5 equiv.), NaI (50 mol%), PPh_3_ (1.0 equiv.), DMA (0.1 mol L^−1^), 440 nm blue LEDs, room temperature, 24 h. Yields were determined by ^19^F NMR using benzotrifluoride as an internal standard. 0.2 mmol scales reaction;

^b)^ Isolated yield in parenthesis. 0.2 mmol scales reactionl

^c)^ Reactions conducted on 5.0 mmol scales. Isolated yields.

LED, light emitting diode; Phth, phthalimide.

### 2.2 Substrate Scope Evaluation

**Figure 2:**
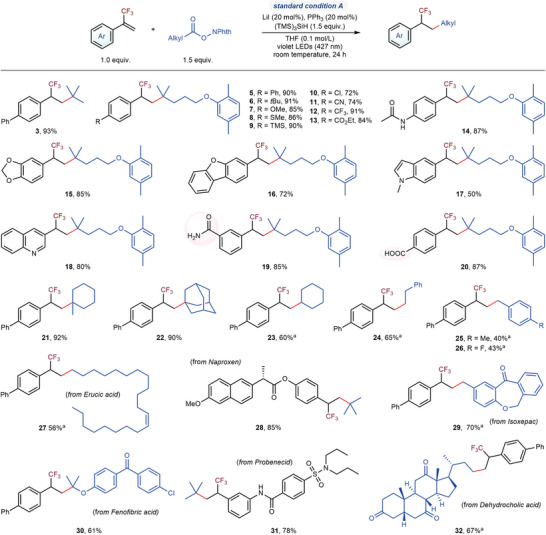
Scope for the synthesis of trifluoromethyl alkanes. Standard condition A, trifluoromethyl alkene (1.0 equiv.), *N*‐hydroxyphthalimide ester (1.5 equiv.), LiI (20 mol%), PPh_3_ (20 mol%), (TMS)_3_SiH (1.5 equiv.), THF (0.1 mol/L), 427 nm violet LEDs, room temperature, 24 h. Reactions were conducted on 0.2 mmol scales. Isolated yields. ^a)^2‐(Diphenylphosphanyl)pyridine (20 mol%) was used, instead of PPh_3_ (20 mol%). TMS, trimethylsilyl.

With the optimized reaction conditions, we next focused on exploring the substrate scope of α‐CF_3_‐styrenes and RAEs to evaluate the generality of this decarboxylative hydroalkylation process (**3**, **5**–**32**). As shown in **Figure** **2**, a variety of α‐CF_3_‐styrenes bearing electron‐donating (**6**–**9**, ‐*t*Bu, ‐OMe, ‐SMe, and ‐TMS) or electron‐withdrawing substituents (**11**–**13**, ‐CN, ‐CF_3_, and ‐CO_2_Et) reacted smoothly, furnishing the corresponding trifluoromethyl alkanes with good‐to‐excellent yields. This simple reaction system exhibited broad functional group compatibility. Thioether (**8**), silyl (**9**), aryl chloride (**10**), cyano (**11**), ester (**13**), and amide (**14**) groups were all well tolerated. Heterocyclic skeletons were commonly found in natural products and drug molecules. This reaction could well tolerate many heteroaromatic rings, such as 1,3‐benzodioxole (**15**), dibenzofuran (**16**), indole (**17**), and quinoline (**18**). Notably, more sensitive groups, such as an amide containing N–H bonds (**19**) and an unprotected carboxyl group (**20**), remained intact in this transformation, highlighting the high functional group tolerance. In addition to tertiary REAs, primary and secondary REAs were also readily converted to the corresponding products in good yields (**23** and **24**). Benzylic radicals are prone to form the homocoupling by‐products. Under our reaction conditions, both electron‐rich (**25**) and electron‐deficient (**26**) benzylic redox‐active esters were suitable substrates to deliver trifluoromethyl alkane products, although further efforts should be devoted to improving the coupling efficiency. To further demonstrate the potential applications of this protocol, the late‐stage functionalization of the derivatives from natural products and drug molecules was conducted. Derivatives of erucic acid (**27**), naproxen (**28**), isoxepac (**29**), fenofibric acid (**30**), probenecid (**31**), and dehydrocholic acid (**32**), were proceeded smoothly to form the corresponding hydroalkylation products. Various functional groups, such as an alkenyl double bond (**27**), ketones (**29**, **30**, and **32**), an amide (**31**), and a sulfonamide (**31**) were all well tolerated. These results demonstrated that this method offers a powerful tool for efficiently constructing complex trifluoromethyl alkanes with a broad substrate scope and good functional group compatibility.

**Figure 3:**
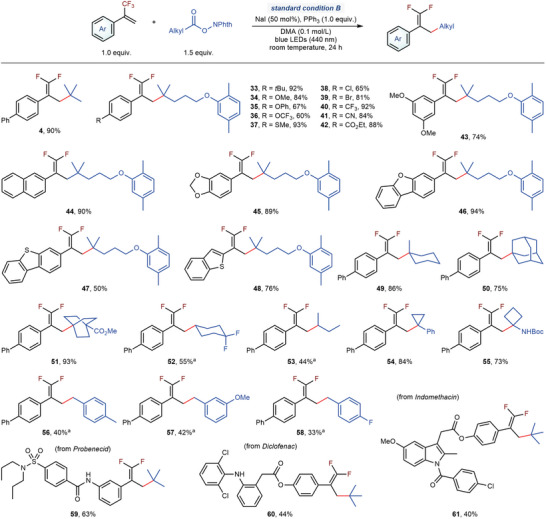
Scope for the synthesis of *gem*‐difluoroalkenes. Standard condition B, trifluoromethyl alkene (1.0 equiv.), *N*‐hydroxyphthalimide ester (1.5 equiv.), NaI (50 mol%), PPh_3_ (1.0 equiv.), DMA (0.1 mol/L), 440 nm blue LEDs, room temperature, 24 h. Reactions were conducted on 0.2 mmol scales. Isolated yields. ^a)^Tetrahexylammonium iodide (50 mol%) was used, instead of NaI (50 mol%). Boc, *tert*‐butoxycarbonyl.

This simple protocol also showed a broad substrate scope for reductive decarboxylative defluorinative alkylation. As depicted in **Figure** **3**, the electronic nature of the substituents on the aromatic ring of α‐CF_3_‐styrenes has no significant impact on the reaction efficiency (**4**, **33**–**44**). Many functional groups, such as ether (**34** and **35**), trifluoromethoxy (**36**), thioether (**37**), aryl chloride (**38**), aryl bromide (**39**), trifluoromethyl (**40**), cyano (**41**), and ester (**42**), were demonstrated to be compatible. Many heteroaromatic rings, such as 1,3‐benzodioxole (**45**), dibenzofuran (**46**), dibenzothiophene (**47**), and benzothiophene (**48**), posed no problem. RAEs derived from cyclic tertiary carboxylic acids (**49** and **50**), cyclic or acyclic secondary (**52** and **53**) aliphatic carboxylic acids, benzyl carboxylic acid (**54**, **56**–**58**), and α‐amino acids (**55**) were reactive to provide target *gem*‐difluoroalkenes in moderate‐to‐good yields. RAE derived from bicyclo[2.2.2]octane bridgehead carboxylic acid also gave the desired product (**51**) successfully. Due to the unique properties of rigid non‐conjugated hydrocarbons,^[^ ^16^ ^]^ Our protocol enabled the convenient installation of saturated carbocyclic structures, providing a potential opportunity for drug discovery. Finally, a series of RAEs derived from drug molecules, such as probenecid (**59**), diclofenac (**60**), and indomethacin (**61**), underwent the reductive decarboxylative defluorinated alkylation smoothly, exhibiting the potential of this transformation towards the late‐stage modification of complex compounds. Notably, no obvious hydroalkylation products were observed in all these cases, highlighting the excellent orthogonal selectivity of different conditions in our divergent synthesis.

### 2.3 Extended Applications

**Figure 4:**
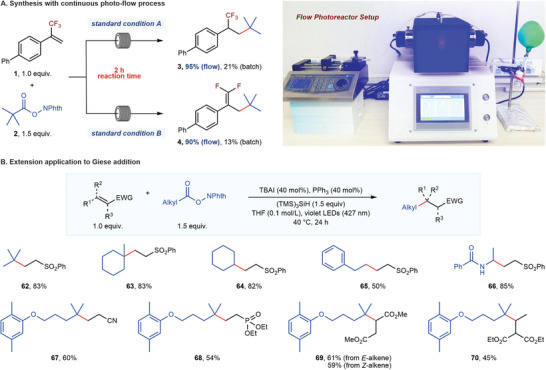
Extended applications. Continuous photo‐flow synthesis was conducted on 0.2 mmol scales (see Supporting Information for details). Giese addition reaction condition, alkene (1.0 equiv.), *N*‐hydroxyphthalimide ester (1.5 equiv.), TBAI (40 mol%), PPh_3_ (40 mol%), (TMS)_3_SiH (1.5 equiv.), THF (0.1 mol/L), 427 nm violet LEDs, 40 °C, 24 h. Giese addition reactions were conducted on 0.1 mmol scales. Isolated yields.

We examined several promising applications of our reactions, including the continuous‐flow synthesis and the extension to general Giese‐type addition reactions (**Figure** **4**). Gratifyingly, the continuous photo‐flow process could significantly reduce the reaction times. In a decreased 2 hours reaction time, the efficiency of this protocol could be significantly improved in continuous‐flow reactors compared to the batch reaction process (Figure 4A). These results demonstrated the practicality and versatility of our method. In addition, this catalytic system's simple operations and low costs appeal to industrial applications to large‐scale manufacture.^[^ ^17^ ^]^

To our delight, with a slight modification on the standard condition A, this decarboxylative hydroalkylation could be successfully extended to the general Giese‐type addition, which is of fundamental importance and great utility in organic synthesis.^[^ ^5^ ^]^ As shown in Figure 4B, RAEs derived from tertiary (**62** and **63**), secondary (**64**), primary (**65**) carboxylic acids, and α‐amino acids (**66**) were all suitable substrates in this Giese‐type reaction. Moreover, Michael acceptors bearing assorted electron‐withdrawing groups, such as sulfones (**62**∼**66**), nitriles (**67**), and phosphonate (**68**), were all able to react in good yields. Finally, internal alkenes (**69** and **70**) with large steric hindrance, even the trisubstituted one (**70**), could be converted into the desired products.

### 2.4 Mechanistic Investigation

**Figure 5:**
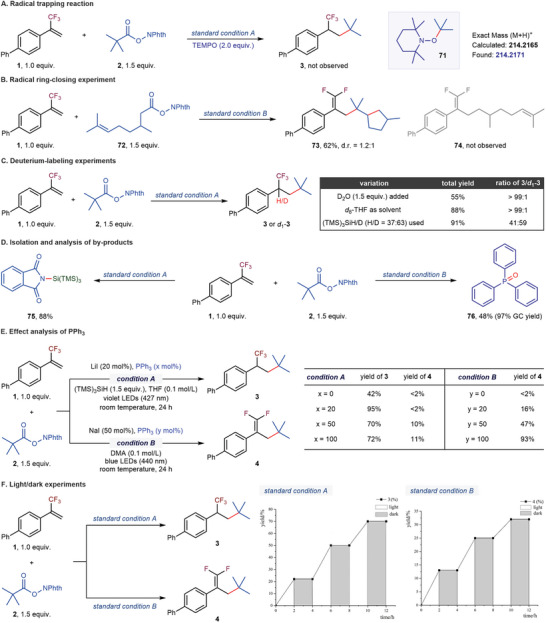
Mechanistic studies. For more details, see Supporting Information. d.r., diastereomeric ratio; GC, gas chromatography.

We conducted a series of mechanistic experiments to better shed light on the reaction mechanism (**Figure** **5**). Firstly, the radical trapping reaction showed that the reaction would be completely shut down in the presence of 2,2,6,6‐tetramethyl‐1‐piperidinyloxy (TEMPO) as a radical scavenger. The TEMPO‐adduct **71** was detected in the reaction mixture (Figure 5A). The reaction between α‐CF_3_‐styrene **1** and citronellic acid‐derived redox‐active ester **72** afforded the cyclic product **73** in a 62% yield (Figure 5B). These results revealed the radical nature of the activation process of *N*‐hydroxyphthalimide esters. A series of deuterium‐labeling experiments were carried out to validate the fact that the hydrogen atom transfer process occurred after the formation of α‐CF_3_ benzyl radical in the hydroalkylation process (Figure 5C). When adding D_2_O as an additive or using *d*_8_‐THF as the solvent, deuterated product *d*_1_‐**3** was not observed. As expected, when using a mixture of (TMS)_3_SiH and (TMS)_3_SiD, deuterated product *d*_1_‐**3** was observed. These results indicated that the hydrogen atom transfer process might be involved in the final hydrogenation step, rather than a carbanion protonation mechanism,^[^ ^13b^ ^]^ and the hydrogen atom originated from the silane. We also successfully isolated (TMS)_3_Si‐NPhth (**75**) from the reaction mixture under standard condition A (Figure 5D), which implied the formation of a silyl cation intermediate. We speculated that the resulting silyl radical from the hydrogen atom transfer process could be oxidized by the iodide radical, and delivered iodide anion to furnish the redox cycle. We also isolated triphenylphosphine oxide (**76**) under standard condition B (Figure 5D). The presence of PPh_3_ has significant influences on the outcomes of hydroalkylation and reductive defluorinative alkylation of α‐CF_3_‐styrenes (Figure 5E). As for the hydroalkylation process, adding a catalytic amount (20 mol%) of PPh_3_ could give the desired product **3** in a 95% yield, due to forming a thermodynamically stable [I‐PPh_3_]^•^ radical to maintain the catalytic activity.^[^ ^15^ ^]^ However, when the amount of PPh_3_ was increased to 0.5 equiv. or even 1.0 equiv., the undesired product **4** was formed in the reaction system, simultaneously with a lower yield of the target product **3**. For reductive defluorinative alkylation, the yield of the desired product **4** was related to the amount of PPh_3_ in a linear correlation. These results implied that the resulting α‐CF_3_ benzyl radical might likely undergo a single electron transfer (SET) reduction by the PPh_3_ to generate a benzyl anion.^[^ ^18^ ^]^ Finally, we performed a light/dark experiment to verify the effect of light irradiation (Figure 5F). Hydroalkylation and defluorinative alkylation of α‐CF_3_‐styrenes only took place under continuous irradiation.

**Figure 6:**
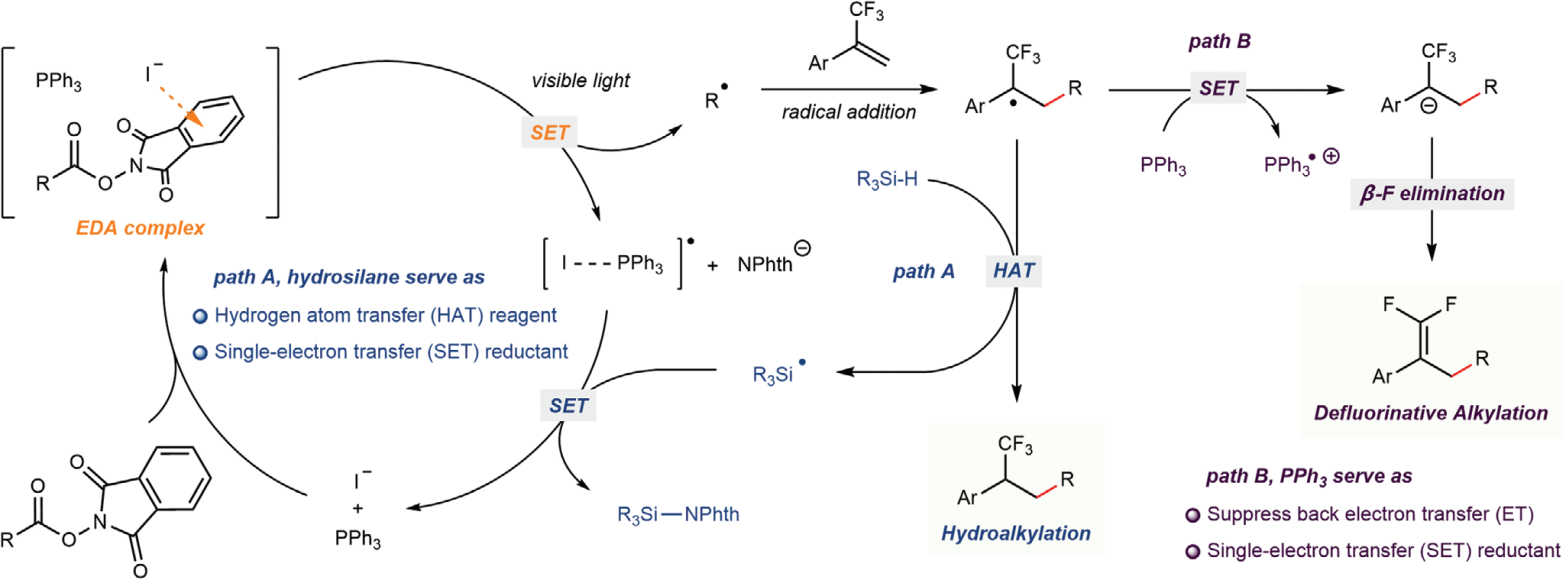
Proposed mechanism.

Based on the above mechanistic results, a plausible reaction mechanism was proposed in **Figure** **6**. Firstly, the alkyl radical, generated from the EDA complex assembled by iodide salts and RAEs, underwent radical addition across trifluoromethyl alkenes to generate α‐CF_3_ benzyl radicals. Subsequently, the divergent alkylation of trifluoromethyl alkenes was achieved by fine‐tuning the reaction conditions, selectively undergoing the hydrogen atom transfer process or the single‐electron reduction coupled β‐F elimination process, respectively. For the hydroalkylation reaction, the hydrosilanes served as a suitable hydrogen source for the hydrogen atom transfer process and acted as a single‐electron reductant to regenerate iodide anions (path A).^[^ ^9^ ^]^ Giese addition reactions proceeded similarly to path A. In the reductive decarboxylative defluorinative alkylation process, α‐CF_3_ benzyl radical could be reduced by triphenylphosphine (E_1/2_ = +0.98 V versus SCE, saturated calomel electrode),^[^ ^19^ ^]^ delivering benzyl anions which smoothly underwent β‐F elimination to produce *gem*‐difluoroalkenes (path B).

## 3 Conclusion

We report a general photoinduced reductive alkylation via an EDA complex strategy to enable divergent decarboxylative hydroalkylation and reductive decarboxylative defluorinative alkylation between α‐CF_3_‐styrenes with *N*‐hydroxyphthalimide esters. These processes are driven by the photoactivity of EDA complexes assembled from iodide salts with RAEs. This protocol allows the simple and efficient preparation of various trifluoromethyl alkanes and *gem*‐difluoroalkenes with high chemoselectivity and excellent functional group tolerance. Preliminary mechanistic studies reveal that the alkyl radicals are generated from the EDA complex and undergo the radical addition across trifluoromethyl alkenes to generate α‐CF_3_ benzyl radicals, and then hydrogen atom transfer occurs as the final hydrogenation step to deliver trifluoromethyl alkanes in the hydroalkylation reaction, and PPh_3_ could serve as a reductant for benzyl anions generation from α‐CF_3_ radicals in the reductive defluorinative alkylation process. The mild transition‐metal‐free reaction conditions, late‐stage functionalization of complex molecules, gram‐scale synthesis, and successful preformation in a continuous flow fashion, are attractive features and highlight the synthetic potential value of our methods. Moreover, decarboxylative hydroalkyltion of universal electron‐deficient alkenes is realized as an extension to the general Giese‐type addition reaction. Further investigations to develop an asymmetric decarboxylative hydroalkyltion are ongoing in our laboratory.

## 4 Experimental Section

### Typical Procedure for Photoinduced Decarboxylative Hydroalkylation

Trifluoromethyl alkene (0.2 mmol, 1.0 equiv., if solid), *N*‐hydroxyphthalimide ester (0.3 mmol, 1.5 equiv., if solid), LiI (20 mol%, 0.04 mmol), and PPh_3_ (20 mol%, 0.04 mmol) were added into a 10 mL transparent Schlenk tube equipped with a stirring bar. The tube was evacuated and filled with argon (repeated three times). To these solids, anhydrous THF (2 mL) was added using a gastight syringe under an argon atmosphere. Then, trifluoromethyl alkene (0.2 mmol, 1.0 equiv., if liquid), *N*‐hydroxyphthalimide ester (0.3 mmol, 1.5 equiv., if liquid), and (TMS)_3_SiH (0.3 mmol, 1.5 equiv.) were added using a gastight syringe under an argon atmosphere. The reaction mixture was stirred under irradiation with 427 nm violet LEDs, maintained at approximately room temperature in the air‐conditioning room of 25 °C. After 24 h, the mixture was quenched with 10 mL water, and extracted with ethyl acetate (3 × 10 mL). The organic layers were combined, dried over anhydrous Na_2_SO_4_, and concentrated on the rotary evaporator. The residue was purified via column chromatography on silica gel to give the product (eluent: petroleum ether/ethyl acetate).

### Typical Procedure for Photoinduced Reductive Decarboxylative Defluorinative Alkylation

Trifluoromethyl alkene (0.2 mmol, 1.0 equiv., if solid), *N*‐hydroxyphthalimide ester (0.3 mmol, 1.5 equiv., if solid), NaI (0.1 mmol, 50 mol%), and PPh_3_ (0.2 mmol, 1.0 equiv.) were added into a 10 mL transparent Schlenk tube equipped with a stirring bar. The tube was evacuated and filled with argon (repeated three times). To these solids, anhydrous DMA (2 mL) was added using a gastight syringe under an argon atmosphere. Then, trifluoromethyl alkene (0.2 mmol, 1.0 equiv., if liquid) and *N*‐hydroxyphthalimide ester (0.3 mmol, 1.5 equiv., if liquid) were added using a gastight syringe under an argon atmosphere. The reaction mixture was stirred under irradiation with 440 nm blue LEDs, maintained at approximately room temperature in the air‐conditioning room of 25 °C. After 24 h, the mixture was quenched with 10 mL water, and extracted with ethyl acetate (3 × 10 mL). The organic layers were combined, dried over anhydrous Na_2_SO_4_, and concentrated on the rotary evaporator. The residue was purified via column chromatography on silica gel to give the product (eluent: petroleum ether/ethyl acetate).

## Conflict of Interest

The authors declare no conflict of interest.

## Supporting information

Supporting Information

## Data Availability

The data that support the findings of this study are available in the supplementary material of this article.
