# Supplementary material for: Photoinduced Triphenylphosphine and Iodide Salt Promoted Reductive Decarboxylative Coupling
Source: Adv Sci (Weinh). 2024 Jan 17;11(12):2307241. doi: 10.1002/advs.202307241 (PMC10966558; doi:10.1002/advs.202307241)

ADVANCED SCIENCE
Open Access

# Supporting Information

for *Adv. Sci.*, DOI 10.1002/advs.202307241

Photoinduced Triphenylphosphine and Iodide Salt Promoted Reductive Decarboxylative Coupling

*Jia-Xin Wang, Ming-Chen Fu*, Lu-Yu Yan, Xi Lu* and Yao Fu**

# Supporting Information

## Photoinduced Triphenylphosphine and Iodide Salt Promoted Reductive Decarboxylative Coupling

Jia-Xin Wang, Ming-Chen Fu*, Lu-Yu Yan, Xi Lu*, and Yao Fu*

E-mail: fuyao@ustc.edu.cn; luxi@mail.ustc.edu.cn; mcfu@hfut.edu.cn

## Table of Contents

1. General Information S3

2. Procedure for the Synthesis of Substrates S4

3. Investigation of the Key Reaction Parameters S5

4. General Procedure and Spectral Data S12

5. General Procedures for Scaling Up S54

6. General Procedure of Continuous-Flow Reactions S55

7. Preliminary Mechanistic Studies S57

8. References S62

9. NMR Spectra S63

# 1. General Information

## A. Materials:

All reactions were carried out in oven-dried Schlenk tubes under argon atmosphere (purity≥99.999%) unless otherwise mentioned. Commercial reagents were purchased from Adamas-beta, TCI and Aldrich, and used without further purification unless otherwise stated. Flash column chromatographic purification of products was accomplished using forced-flow chromatography on Silica Gel (200-300 mesh). All capillary tubing and microfluidic fittings and syringe pumps were purchased from Baoding Di Chuang Electronic Technology Co., Ltd., flow photocatalytic reactor was purchased from Anhui Kexin Microfluidic Chemical Technology Co., Ltd. The LED lamps were purchased from Kessil (PR160-390 nm, 427 nm, 440 nm, 456 nm, 467 nm). The Photo Reaction Setup was purchased from Anhui kemi machinery technology Co., Ltd.

## B. Analytical Methods:

$^{1}$H-NMR, $^{19}$F-NMR and $^{13}$C-NMR spectra were recorded on Bruker Avance 400, 500 or 600 spectrometer at ambient temperature. Data for $^{1}$H-NMR are reported as follows: chemical shift (ppm, scale), multiplicity (s = singlet, d = doublet, t = triplet, q = quartet, m = multiplet and/or multiplet resonances, br = broad), coupling constant (Hz), and integration. Data for $^{13}$C-NMR were reported in terms of chemical shift (ppm, scale), multiplicity, and coupling constant (Hz). HRMS analysis was performed on Finnigan LCQ advantage Max Series MS System. ESI-mass data or EI-mass data were acquired using a Acquity UPLC-Xevo G2 QTof）(1307223S, 1307219S) Instrument equipped with an ESI or EI source and controlled by Xcalibur software.

## 2. Procedure for the Synthesis of Substrates

### 2.1 General Procedure for preparation of Trifluoromethyl alkenes[1]

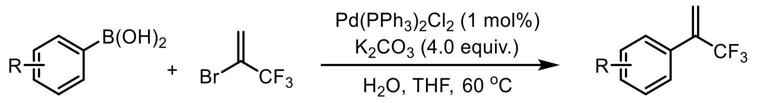


To a Schlenk tube equipped a magnetic stir bar, boronic acid (5 mmol, 1.0 equiv.), and $Pd(PPh_3)_2Cl_2$ (35.1 mg, 1 mol%) were added. The vessel was evacuated and filled with argon (three times), and then THF (15 mL) and aqueous $K_2CO_3$ (2.0 M, 10 mL) were added. After the addition of 2-bromo-3,3,3-trifluoropropene (1.0 mL, 10 mmol, 2.0 equiv.), the reaction mixture was stirred at 60 °C overnight under an argon atmosphere. The resultant mixture was cooled to room temperature, quenched with saturated aqueous $NH_4Cl$, and extracted with EtOAc (3 × 15 mL). The combined organic phases were dried over anhydrous $Na_2SO_4$, filtered, and concentrated under reduced pressure. The residue was purified by column chromatography on silica gel (petroleum ether/ethyl acetate) to give the desired corresponding trifluoromethyl alkene.

### 2.2 General Procedure for preparation of The Redox Active Esters[2]

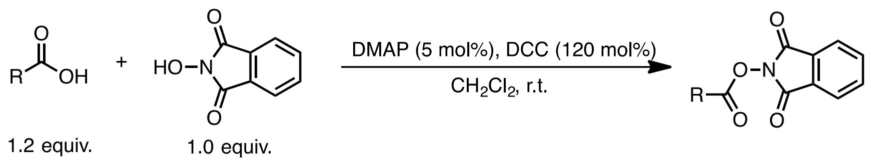


The corresponding alkyl carboxylic acids or *N*-protected amino acids (12 mmol, 1.2 equiv), *N*-hydroxyphthalimide (10 mmol, 1.0 equiv), and 4-dimethylaminopyridine (0.5 mmol, 5 mol %) were mixed in a flask with a magnetic stirring bar. 40 mL dry $CH_2Cl_2$ was added, then a solution of *N, N´*-dicyclohexylcarbodiimide (12 mmol, 120 mol%) in $CH_2Cl_2$ (15 mL) was added slowly at room temperature. The reaction mixture was stirred at room temperature for 1 h. After *N*-hydroxyphthalimide was completely converted, the white precipitate was filtered off and the solution was concentrated under vacuum. Corresponding redox active esters were purified by column chromatography on silica gel ($CH_2Cl_2$ or petroleum ether/ethyl acetate as eluent).

## 3. Investigation of the Key Reaction Parameters

## 3.1 Optimization of the reaction conditions for hydroalkylation

**Table S1. Screening of Solvents**

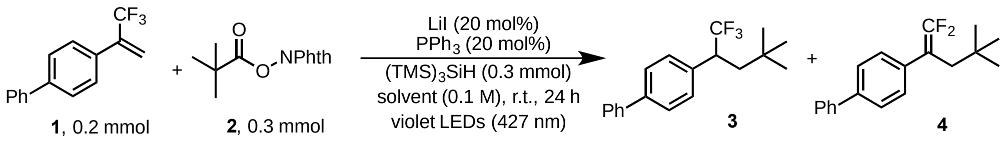


| Entry | Solvent | Yield of **3** (%) | Yield of **4** (%) |
|---|---|---|---|
| 1 | DMA | trace | 0 |
| 2 | DMF | trace | 0 |
| 3 | THF | 95 (93) | 0 |
| 4 | acetone | 25 | 0 |
| 5 | 1,4-dioxane | 18 | trace |
| 6 | $CH_2Cl_2$ | 8 | 15 |
| 7 | DMSO | 13 | 7 |
| 8 | $CH_3CN$ | 42 | trace |

Reaction conditions: **1** (0.2 mmol), **2** (0.3 mmol), LiI (20 mol%), $PPh_3$ (20 mol%), $(TMS)_3SiH$ (0.3 mmol), solvent (0.1 M), r.t., 24 h, 427 nm violet LEDs. The yield was determined by $^{19}F$ NMR used benzotrifluoride as an internal standard; the isolated yield was given in parenthes.

**Table S2. Screening of H-Sources**

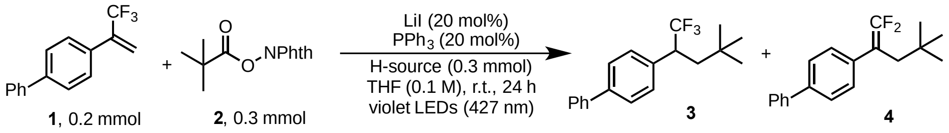


| Entry | H-source | Yield of **3** (%) | Yield of **4** (%) |
|---|---|---|---|
| 1 | $Ph_2MeSiH$ | trace | 0 |
| 2 | $Ph_3SiH$ | trace | 0 |
| 3 | 1,1,1,3,3,3-hexafluoropropan-2-ol | trace | 0 |
| 4 | $(TMS)_3SiH$ | 93 | 0 |
| 5 | $(Et)_3SiH$ | trace | 0 |
| 6 | 1,4-cyclohexadiene | 23 | 0 |
| 7 | 1,4-cyclohexadien | 23 | 0 |
| 8 | $H_2O$ | trace | 0 |

Reaction conditions: **1** (0.2 mmol), **2** (0.3 mmol), LiI (20 mol%), $PPh_3$ (20 mol%), H-source (0.3 mmol), THF (0.1 M), r.t., 24 h, 427 nm violet LEDs. The yield was determined by $^{19}F$ NMR used benzotrifluoride as an internal standard.

**Table S3. Screening of I-Sources**

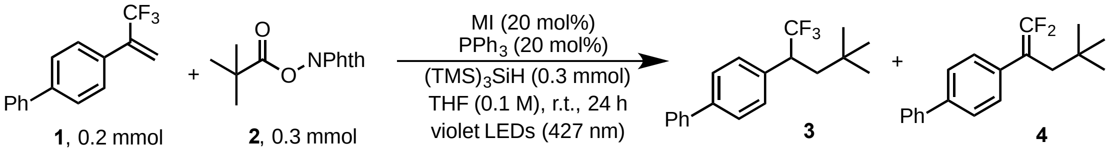


| Entry | I-source | Yield of **3** (%) | Yield of **4** (%) |
|---|---|---|---|
| 1 | LiI | 93 | 0 |
| 2 | NaI | 75 | trace |
| 3 | KI | 65 | trace |
| 4 | Tetrabutylammonium Iodide | 40 | 24 |
| 5 | Tetrahexylaminium Iodide | 37 | 32 |

Reaction conditions: **1** (0.2 mmol), **2** (0.3 mmol), MI (20 mol%), $PPh_3$ (20 mol%), $(TMS)_3SiH$ (0.3 mmol), THF (0.1 M), r.t., 24 h, 427 nm violet LEDs. The yield was determined by $^{19}F$ NMR used benzotrifluoride as an internal standard.

**Table S4. Screening of Light Sources**

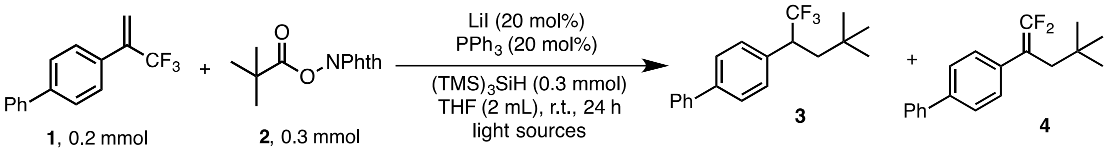


| Entry | Light sources | Yield of **3** (%) | Yield of **4** (%) |
|---|---|---|---|
| 1 | 390 nm violet LEDs | 63 | 0 |
| 2 | 427 nm violet LEDs | 93 | 0 |
| 3 | 440 nm blue LEDs | 76 | 0 |
| 4 | 456 nm blue LEDs | 76 | 0 |
| 5 | 467 nm blue LEDs | 64 | 0 |

Reaction conditions: **1** (0.2 mmol), **2** (0.3 mmol), LiI (20 mol%), $PPh_3$ (20 mol%), $(TMS)_3SiH$ (0.3 mmol), THF (0.1 M), r.t., 24 h, light sources. The yield was determined by $^{19}F$ NMR used benzotrifluoride as an internal standard.

**Table S5. Control Experiments**

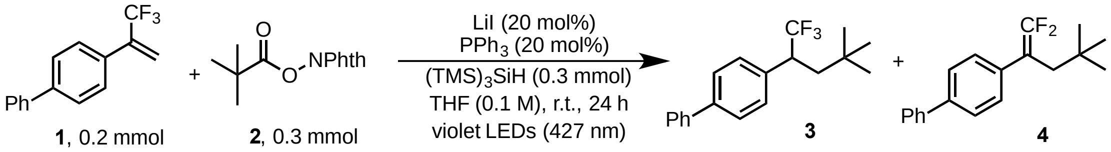


| Entry | Variation from standard conditions | Yield of **3** (%) | Yield of **4** (%) |
|---|---|---|---|
| 1 | No $(TMS)_3SiH$ | 0 | 0 |
| 2 | No LiI | 0 | 0 |
| 3 | No $PPh_3$ | 42 | 0 |
| 4 | No light | 0 | 0 |
| 5 | $(TMS)_3SiH$ (0.2 mmol) | 64 | 0 |
| 6 | LiI (0.3 mmol), $PPh_3$ (0.3 mmol) | 76 | 10 |
| 7 | LiI (0.1 mmol), $PPh_3$ (0.1 mmol) | 78 | 9 |
| 8 | LiI (20 mol%), $PPh_3$ (20 mol%) | 93 | trace |
| 9 | LiI (10 mol%), $PPh_3$ (10 mol%) | 51 | trace |

Reaction conditions: **1** (0.2 mmol), **2** (0.3 mmol), LiI (20 mol%), $PPh_3$ (20 mol%), $(TMS)_3SiH$ (0.3 mmol), THF (0.1 M), r.t., 24 h, 427 nm violet LEDs. The yield was determined by $^{19}F$ NMR used benzotrifluoride as an internal standard.

We previously compared the UV-vis absorption spectra of transiently assembled chromophores composed of an iodide salt, $PPh_3$ and a RAE with the UV-vis absorption spectra observed in the absence of $PPh_3$, the spectra were highly similar. Therefore, the decarboxylative alkylation could occur without $PPh_3$ with a low efficiency, which was theoretically plausible. The similar experimental results have also been reported in recent relevant studies. It is worth mentioning that, we found the presence of $PPh_3$ facilitated the electron transfer between NaI and RAE, and also crucial to suppress back electron transfer from the phthalimide radical anion to $I^•$, by forming thermodynamically stable $[I\text{-}PPh_3]^•$, and to prevent the formation of $I_2$, which was found to be detrimental. As a result, addition of a catalytic amount of $PPh_3$ would improve the efficiency, but it's not necessary.

**Table S6. Screening of Triarylphosphines**

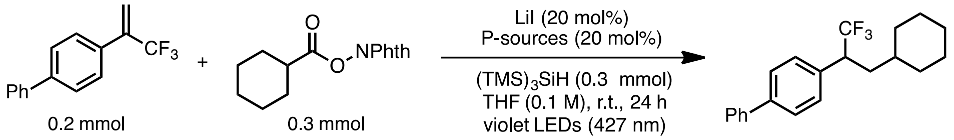


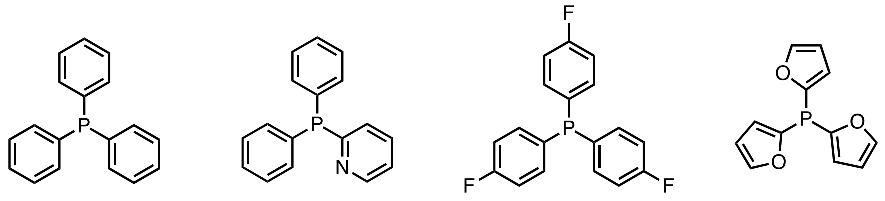


**L1**, 40% **L2**, 66% **L3**, 60% **L4**, 28%

Reaction conditions: **1** (0.2 mmol), **2** (0.3 mmol), LiI (20 mol%), P-sources (20 mol%), $(TMS)_3SiH$ (0.3 mmol), THF (0.1 M), r.t., 24 h, 427 nm violet LEDs. The yield was determined by $^{19}F$ NMR used benzotrifluoride as an internal standard.

**Table S7. The Effect of $PPh_3$**

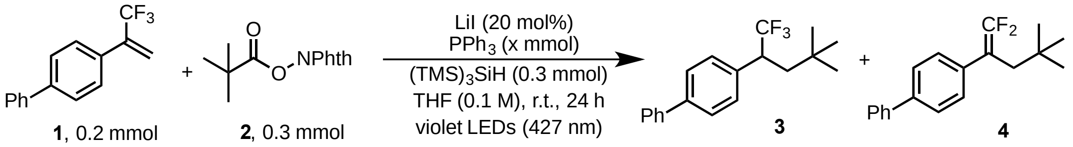


| Entry | Variation from standard conditions | Yield of **3** (%) | Yield of **4** (%) |
|---|---|---|---|
| 1[a] | none | 93 | 0 |
| 2 | $PPh_3$ (0.1 mmol) | 70 | 10 |
| 3 | $PPh_3$ (0.2 mmol) | 72 | 11 |
| 4 | $PPh_3$ (0.3 mmol) | 72 | 12 |
| 5 | $PPh_3$ (0.4 mmol) | 70 | 12 |

Reaction conditions: **1** (0.2 mmol), **2** (0.3 mmol), LiI (20 mol%), $PPh_3$ (x mmol), $(TMS)_3SiH$ (0.3 mmol), THF (0.1 M), r.t., 24 h, 427 nm violet LEDs. The yield was determined by $^{19}F$ NMR used benzotrifluoride as an internal standard. [a]The isolated yield of 2-(1,1,1,3,3,3-hexamethyl-2-(trimethylsilyl)trisilan-2-yl)isoindoline-1,3-dione was 88%.

### 3.2 Optimization of the reaction conditions for defluorinative alkylation

**Table S8. Screening of Solvents**

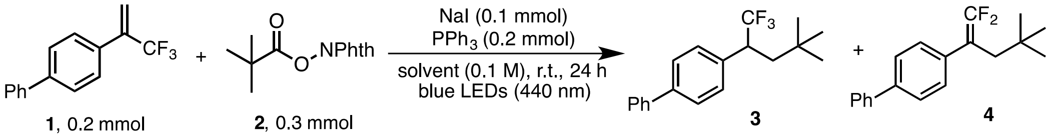


| Entry | Solvent | Yield of **3** (%) | Yield of **4** (%) |
|---|---|---|---|
| 1 | DMA | 0 | 93 |
| 2 | DMF | 0 | 55 |
| 3 | THF | 0 | trace |
| 4 | acetone | trace | 43 |
| 5 | 1,4-dioxane | 0 | 0 |
| 6 | DMSO | 0 | 0 |
| 7 | $CH_3CN$ | 0 | 11 |

Reaction conditions: **1** (0.2 mmol), **2** (0.3 mmol), NaI (0.1 mmol), $PPh_3$ (0.2 mmol), solvent (0.1 M), 24 h, r.t., 440 nm blue LEDs. The yield was determined by $^{19}F$ NMR used benzotrifluoride as an internal standard.

**Table S9. Screening of I-Sources**

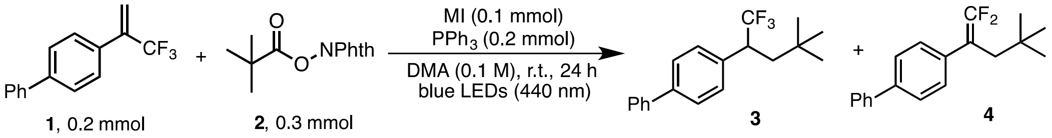


| Entry | I-source | Yield of **3** (%) | Yield of **4** (%) |
|---|---|---|---|
| 1 | NaI | 0 | 93 |
| 2 | KI | 0 | 65 |
| 3 | LiI | 0 | 74 |
| 4 | CsI | 0 | 0 |
| 5 | Tetramethylammonium Iodide | 0 | 58 |
| 6 | Tetraethylammonium Iodide | 0 | 65 |
| 7 | Tetrabutylammonium Iodide | 0 | 51 |
| 8 | Tetrahexylaminium Iodide | 0 | 75 |

Reaction conditions: **1** (0.2 mmol), **2** (0.3 mmol,), NaI (0.1 mmol), $PPh_3$ (0.2 mmol), DMA (0.1 M), r.t, 24 h, 440 nm blue LEDs. The yield was determined by $^{19}F$ NMR used benzotrifluoride as an internal standard.

**Table S10. Screening of Light Sources**

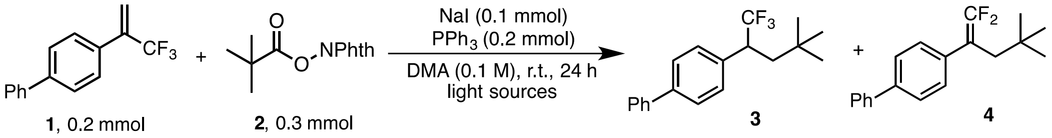


| Entry | Light sources | Yield of **3** (%) | Yield of **4** (%) |
|---|---|---|---|
| 1 | 390 nm violet LEDs | 0 | 86 |
| 2 | 427 nm violet LEDs | 0 | 88 |
| 3 | 440 nm blue LEDs | 0 | 93 |
| 4 | 456 nm blue LEDs | 0 | 85 |
| 5 | 467 nm blue LEDs | 0 | 22 |

Reaction conditions: **1** (0.2 mmol), **2** (0.3 mmol), NaI (0.1 mmol), $PPh_3$ (0.2 mmol), DMA (0.1 M), r.t., 24 h, light. The yield was determined by $^{19}F$ NMR used benzotrifluoride as an internal standard.

**Table S11. Control Experiments**

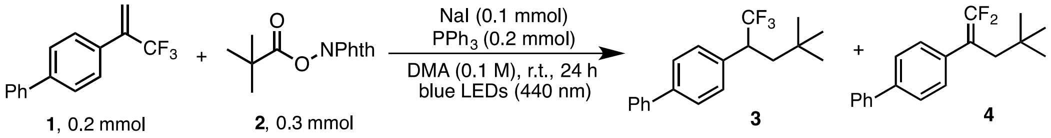


| Entry | Variation from standard conditions | Yield of **3** (%) | Yield of **4** (%) |
|---|---|---|---|
| 1 | No NaI | 0 | 0 |
| 2 | No $PPh_3$ | 0 | 0 |
| 3 | No light | 0 | 0 |
| 4 | NaI (0.2 mmol), $PPh_3$ (0.2 mmol) | 0 | 88 |
| 5 | NaI (0.1 mmol), $PPh_3$ (0.2 mmol) | 0 | 93 |
| 6 | NaI (0.1 mmol), $PPh_3$ (0.1 mmol) | 0 | 53 |

Reaction conditions: **1** (0.2 mmol), **2** (0.3 mmol), NaI (0.1 mmol), $PPh_3$ (0.2 mmol), DMA (2 mL), r.t., 24 h, 440 nm blue LEDs. The yield was determined by $^{19}F$ NMR used benzotrifluoride as an internal standard.

**Table S12. The Effect of $PPh_3$**

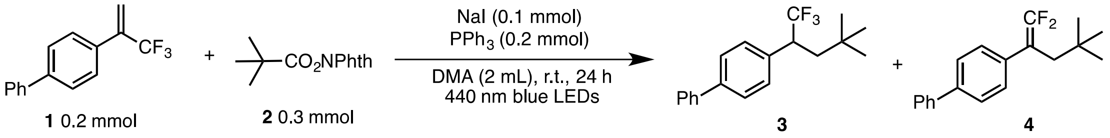


| Entry | Variation from standard conditions | Yield of **3** (%) | Yield of **4** (%) |
|---|---|---|---|
| 1[a] | none | 0 | 93 |
| 2 | NaI (0.1 mmol), $PPh_3$ (10 mol%) | 0 | 7 |
| 3 | NaI (0.1 mmol), $PPh_3$ (20 mol%) | 0 | 16 |
| 4 | NaI (20 mol%), $PPh_3$ (0.1 mmol) | 0 | 35 |
| 5 | NaI (0.1 mmol), $PPh_3$ (0.1 mmol) | 0 | 47 |
| 6 | added $H_2O$ (0.4 mmol) | 0 | 94 |

Reaction conditions: **1** (0.2 mmol), **2** (0.3 mmol), NaI (0.1 mmol), $PPh_3$ (0.2 mmol), DMA (2 mL), r.t., 24 h, 440 nm blue LEDs. The yield was determined by $^{19}F$ NMR used benzotrifluoride as an internal standard. [a]The isolated yield of triphenylphosphine oxide was 48% (97% GC yield).

The oxygen atom of triphenylphosphine oxide originated from both reagent or solvent residual water and reaction post-treatment water. On the other hand, we examined the reaction with additional 2.0 equiv. water, attaining the same coupling efficiency (94% $^{19}F$ NMR yield). Therefore, we didn't process the anhydrous treatment on commercially available reagents, because these reactions were not sensitive to water.

**Table S13. Unsuccessful Substrate**

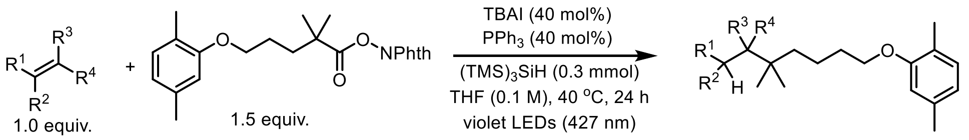


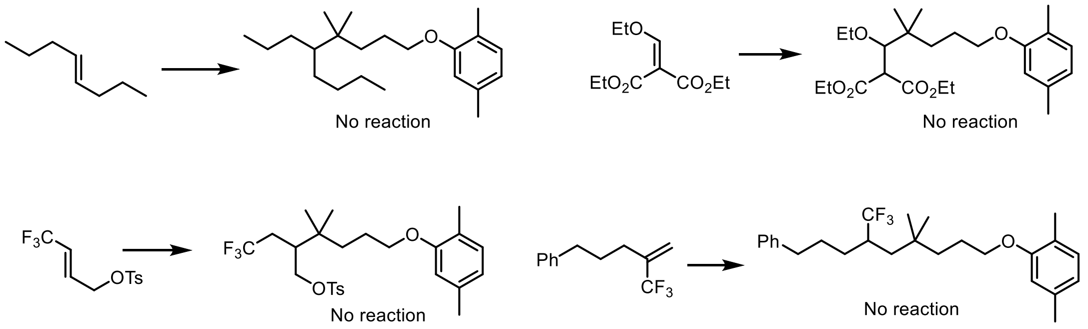

# 4. General Procedure and Spectral Data

## 4.1 General Procedure

**General Procedure A for hydroalkylation:** The redox active esters (1.5 equiv., 0.3 mmol) (if solid), trifluoromethyl alkenes (1.0 equiv., 0.2 mmol) (if solid), LiI (20 mol%, 0.04 mmol), $PPh_3$ (20 mol%, 0.04 mmol) were placed in a 10 mL transparent Schlenk tube equipped with a stirring bar. The tube was evacuated and filled with argon (repeated for three times). To this solid, trifluoromethyl alkenes (1.0 equiv., 0.2 mmol) (if liquid), $(TMS)_3SiH$ (1.5 equiv., 0.3 mmol) and anhydrous THF (0.1 M) were added using a gastight syringe under argon atmosphere. The reaction mixture was stirred under irradiation with 427 nm violet LEDs, maintained at approximately room temperature in the air-conditioned room. After 24 h, the mixture was quenched with 10 mL water, extracted with ethyl acetate (3 x 10 mL).The organic layers were combined and concentrated on rotary evaporator. The residue was purified *via* flash column chromatography on silica gel to give the product. (Eluent: petroleum ether/ethyl acetate).

**General Procedure B for defluorinative alkylation:** The redox active esters (1.5 equiv., 0.3 mmol) (if solid), trifluoromethyl alkenes (1.0 equiv., 0.2 mmol) (if solid), NaI (50 mol%, 0.1 mmol), $PPh_3$ (1.0 equiv., 0.2 mmol) were placed in a 10 mL transparent Schlenk tube equipped with a stirring bar. The tube was evacuated and filled with argon (repeated for three times). To this solid, trifluoromethyl alkenes (1.0 equiv., 0.2 mmol) (if liquid) and anhydrous DMA (0.1 M) were added using a gastight syringe under argon atmosphere. The reaction mixture was stirred under irradiation with 440 nm blue LEDs, maintained at approximately room temperature in the air-conditioned room. After 24 h, ethyl acetate (5 mL) was added to the reaction mixture. The resulting solution was washed with brine (3 x 10 mL) and dried over anhydrous $Na_2SO_4$. The organic layers were combined and concentrated on rotary evaporator. The residue was purified *via* flash column chromatography on silica gel to give the product. (Eluent: petroleum ether/ethyl acetate).

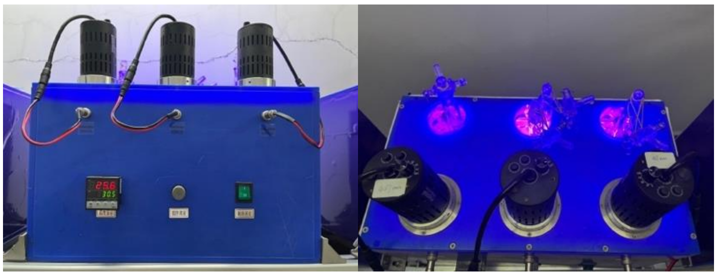

**Figure S1** The photo-reaction setup

### 4.2 Characterization data for the products

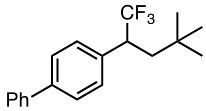


**4-(1,1,1-trifluoro-4,4-dimethylpentan-2-yl)-1,1'-biphenyl (3)**

Prepared following the general procedure A. Purification by column chromatography (Eluent: petroleum ether) provided the title compound (56.9 mg, 93% yield), as a white solid.

$^{1}$H NMR (500 MHz, Chloroform-*d*) δ 7.66 – 7.51 (m, 4H), 7.47 – 7.30 (m, 5H), 3.40 – 3.31 (m, 1H), 1.98 – 1.91 (m, 2H), 0.83 (s, 9H).

$^{13}$C NMR (126 MHz, Chloroform-*d*) δ 140.8, 140.5, 135.8, 129.8, 128.8, 127.4, 127.2, 127.1, 46.7 (q, *J* = 26.2 Hz), 42.1, 30.9, 29.8.

$^{19}$F NMR (471 MHz, Chloroform-*d*) δ -70.21 (d, *J* = 9.3 Hz).

HRMS (ESI) (m/z): $[M+Na]^+$ Calcd for $C_{19}H_{21}F_3Na^+$, 329.1488; Found 329.1494.

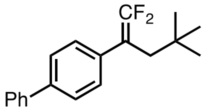


**4-(1,1-difluoro-4,4-dimethylpent-1-en-2-yl)-1,1'-biphenyl (4)[3]**

Prepared following the general procedure B. Purification by column chromatography (petroleum ether) provided the title compound (52.1 mg, 91% yield), as a colorless oil.

$^{1}$H NMR (400 MHz, Chloroform-*d*) δ 7.61 – 7.49 (m, 4H), 7.47 – 7.28 (m, 5H), 2.36 (t, *J* = 2.4 Hz, 2H), 0.83 (s, 9H).

$^{13}$C NMR (126 MHz, Chloroform-*d*) δ 154.6 (dd, *J* = 290.5, 287.6 Hz), 140.7, 139.8, 134.7 (dd, *J* = 4.4, 3.0 Hz), 128.9 (d, *J* = 1.8 Hz), 127.4, 127.1, 127.0, 91.0 (dd, *J* = 21.9, 12.7 Hz), 41.1, 32.9 (t, *J* = 2.5 Hz), 29.9.

$^{19}$F NMR (376 MHz, Chloroform-*d*) δ -89.18 (d, *J* = 40.2 Hz), -91.86 (d, *J* = 40.3 Hz).

The spectroscopic data were consistent with those previously reported in the literature[3].

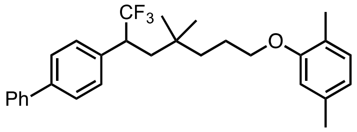


**4-(7-(2,5-dimethylphenoxy)-1,1,1-trifluoro-4,4-dimethylheptan-2-yl)-1,1'-biphenyl (5)**

Prepared following the general procedure A. Purification by column chromatography (Eluent: petroleum ether/ethyl acetate = 50/1) provided the title compound (81.8 mg, 90% yield), as a white solid.

$^{1}$H NMR (400 MHz, Chloroform-*d*) δ 7.51 – 7.44 (m, 4H), 7.39 – 7.20 (m, 5H), 6.91 (dd, *J* = 7.5, 2.7 Hz, 1H), 6.56 (d, *J* = 7.4 Hz, 1H), 6.45 (d, *J* = 4.0 Hz, 1H), 3.72 – 3.58 (m, 2H), 3.38 – 3.22 (m, 1H), 2.17 (s, 3H), 2.08 (s, 3H), 1.95 – 1.86 (m, 1H), 1.76 – 1.57 (m, 1H), 1.55 – 1.43 (m, 1H), 1.35 – 1.09 (m, 3H), 0.79 – 0.66 (m, 6H).

$^{13}$C NMR (101 MHz, Chloroform-*d*) δ 157.0, 140.9, 140.5, 136.5, 130.3, 129.8, 128.8, 127.5, 127.3, 127.1, 127.0, 123.5, 120.7, 111.9, 68.2, 46.3 (q, *J* = 26.2 Hz), 39.8, 38.2, 33.2, 27.7, 27.6, 24.1, 21.4, 15.9.

$^{19}$F NMR (376 MHz, Chloroform-*d*) δ -70.06 (d, *J* = 10.0 Hz).

HRMS (ESI) (m/z): $[M+Na]^+$ Calcd for $C_{29}H_{33}F_3NaO^+$, 477.2376; Found 477.2372.

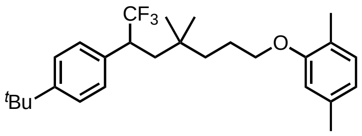


**2-((6-(4-(tert-butyl)phenyl)-7,7,7-trifluoro-4,4-dimethylheptyl)oxy)-1,4-dimethylbenzene (6)**

Prepared following the general procedure A. Purification by column chromatography (Eluent: petroleum ether/ethyl acetate = 50/1) provided the title compound (79.0 mg, 91% yield), as a colorless oil.

$^{1}$H NMR (400 MHz, Chloroform-*d*) δ 7.37 – 7.29 (m, 2H), 7.24 (d, *J* = 8.4 Hz, 2H), 6.99 (d, *J* = 7.1 Hz, 1H), 6.64 (d, J = 7.0 Hz, 1H), 6.54 (s, 1H), 3.76 – 3.60 (m, 2H), 3.40 – 3.21 (m, 1H), 2.29 (s, 3H), 2.16 (s, 3H), 2.00 – 1.87 (m, 2H), 1.76 – 1.61 (m,

1H), 1.58 – 1.42 (m, 1H), 1.38 – 1.30 (m, 1H), 1.29 (s, 9H), 1.24 – 1.12 (m, 1H), 0.80 (d, *J* = 2.9 Hz, 6H).

$^{13}$C NMR (126 MHz, Chloroform-*d*) δ 157.0, 150.9, 136.4, 133.5, 130.3, 129.0, 125.4, 123.5, 120.6, 111.9, 68.2, 46.1 (q, *J* = 26.0 Hz), 39.7, 38.0, 34.5, 33.1, 31.3, 27.7, 27.6, 24.1, 21.4, 15.8.

$^{19}$F NMR (376 MHz, Chloroform-*d*) δ -70.18 (d, *J* = 9.8 Hz).

HRMS (ESI) (m/z): $[M+H]^+$ Calcd for $C_{27}H_{38}F_3O^+$, 435.2869; Found 435.2872.

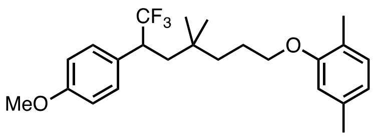


**1,4-dimethyl-2-((7,7,7-trifluoro-6-(4-methoxyphenyl)-4,4-dimethylheptyl)oxy)benzene (7)**

Prepared following the general procedure A. Purification by column chromatography (Eluent: petroleum ether/ethyl acetate = 50/1) provided the title compound (69.4 mg, 85% yield), as a colorless oil.

$^{1}$H NMR (400 MHz, Chloroform-*d*) δ 7.30 (d, *J* = 8.4 Hz, 2H), 7.07 (d, *J* = 7.5 Hz, 1H), 6.97 – 6.88 (m, 2H), 6.72 (dd, *J* = 7.3, 1.7 Hz, 1H), 6.63 (d, *J* = 1.6 Hz, 1H), 3.87 – 3.77 (m, 5H), 3.45 – 3.28 (m, 1H), 2.37 (s, 3H), 2.23 (d, *J* = 4.0 Hz, 3H), 1.98 (d, *J* = 5.9 Hz, 1H), 1.83 – 1.69 (m, 1H), 1.70 – 1.60 (m, 1H), 1.45 – 1.27 (m, 3H), 0.86 (d, *J* = 2.0 Hz, 6H).

$^{13}$C NMR (101 MHz, Chloroform-*d*) δ 159.3, 157.0, 136.5, 130.3, 129.6, 128.6, 123.5, 120.6, 114.0, 112.0, 68.3, 55.2, 45.8 (q, *J* = 26.2 Hz), 39.8, 38.2, 33.1, 27.6, 27.5, 24.1, 21.4, 15.8.

$^{19}$F NMR (376 MHz, Chloroform-*d*) δ -70.19 (d, *J* = 10.0 Hz).

HRMS (ESI) (m/z): $[M+H]^+$ Calcd for $C_{24}H_{32}F_3O_2^+$, 409.2349; Found 409.2354.

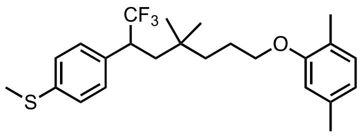


**(4-(7-(2,5-dimethylphenoxy)-1,1,1-trifluoro-4,4-dimethylheptan-2-yl)phenyl)(methyl)sulfane (8)**

Prepared following the general procedure A. Purification by column chromatography (Eluent: petroleum ether/ethyl acetate = 50/1) provided the title compound (72.9 mg, 86% yield), as a yellow oil.

$^{1}$H NMR (400 MHz, Chloroform-*d*) δ 7.14 (q, *J* = 8.4 Hz, 4H), 6.92 (d, *J* = 7.5 Hz, 1H), 6.58 (d, *J* = 7.4 Hz, 1H), 6.49 (d, *J* = 1.5 Hz, 1H), 3.77 – 3.55 (m, 2H), 3.29 – 3.14 (m, 1H), 2.36 (s, 3H), 2.22 (s, 3H), 2.09 (s, 3H), 1.88 – 1.81 (m, 1H), 1.68 – 1.56 (m, 1H), 1.55 – 1.44 (m, 1H), 1.36 – 1.05 (m, 3H), 0.71 (s, 6H).

$^{13}$C NMR (101 MHz, Chloroform-*d*) δ 155.9, 137.4, 135.4, 132.2, 129.2, 128.7, 125.3, 122.4, 119.6, 110.8, 67.1, 45.0 (q, *J* = 26.3 Hz), 38.7, 37.1, 32.0, 26.6, 26.4, 23.0, 20.4, 14.8, 14.4.

$^{19}$F NMR (376 MHz, Chloroform-*d*) δ -70.28 (d, *J* = 10.0 Hz).

HRMS (ESI) (m/z): $[M+Na]^+$ Calcd for $C_{24}H_{31}F_3NaOS^+$, 447.1940; Found 447.1946.

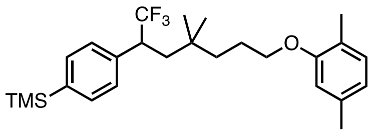


**(4-(7-(2,5-dimethylphenoxy)-1,1,1-trifluoro-4,4-dimethylheptan-2-yl)phenyl)trimethylsilane (9)**

Prepared following the general procedure A. Purification by column chromatography (Eluent: petroleum ether/ethyl acetate = 50/1) provided the title compound (81.0 mg, 90% yield), as a colorless oil.

$^{1}$H NMR (400 MHz, Chloroform-*d*) δ 7.47 (d, *J* = 7.6 Hz, 2H), 7.31 (d, *J* = 7.8 Hz, 2H), 7.00 (d, *J* = 7.4 Hz, 1H), 6.65 (d, *J* = 7.7 Hz, 1H), 6.55 (s, 1H), 3.81 – 3.51 (m, 2H), 3.38 – 3.07 (m, 1H), 2.30 (s, 3H), 2.16 (s, 3H), 1.95 (d, *J* = 3.7 Hz, 1H), 1.77 – 1.63 (m, 1H), 1.60 – 1.48 (m, 1H), 1.44 – 1.09 (m, 3H), 0.80 (s, 6H), 0.24 (s, 9H).

$^{13}C$ NMR (126 MHz, Chloroform-*d*) δ 158.1, 141.4, 138.2, 137.6, 134.7, 134.4, 131.4, 129.9, 124.6, 121.8, 113.0, 69.3, 47.7 (q, *J* = 26.0 Hz), 40.9, 39.2, 34.2, 28.8, 28.7, 25.2, 22.6, 17.0, 0.0.

$^{19}F$ NMR (376 MHz, Chloroform-*d*) δ -70.01 (d, *J* = 9.9 Hz).

HRMS (ESI) (m/z): $[M+H]^+$ Calcd for $C_{26}H_{38}F_3OSi^+$, 451.2639; Found 451.2646.

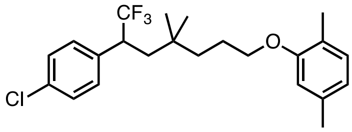


**2-((6-(4-chlorophenyl)-7,7,7-trifluoro-4,4-dimethylheptyl)oxy)-1,4-dimethylbenzene (10)**

Prepared following the general procedure A. Purification by column chromatography (Eluent: petroleum ether/ethyl acetate = 50/1) provided the title compound (59.3 mg, 72% yield), as a yellow oil.

$^1H$ NMR (400 MHz, Chloroform-*d*) δ 7.26 – 7.19 (m, 4H), 6.96 (d, *J* = 7.5 Hz, 1H), 6.61 (d, *J* = 7.5 Hz, 1H), 6.53 (s, 1H), 3.74 (m, 2H), 3.27 (m, 1H), 2.26 (s, 3H), 2.12 (s, 3H), 1.91 – 1.85 (m, 1H), 1.72 – 1.62 (m, 1H), 1.57 – 1.51 (m, 1H), 1.38 – 1.12 (m, 3H), 0.73 (d, *J* = 1.3 Hz, 6H).

$^{13}C$ NMR (151 MHz, Chloroform-*d*) δ 157.0, 136.6, 135.3, 134.1, 130.8, 130.4, 128.9, 123.6, 120.8, 112.1, 68.2, 46.1 (q, *J* = 27.2 Hz), 40.0, 38.4, 33.2, 27.7, 27.5, 24.1, 21.5, 15.9.

$^{19}F$ NMR (376 MHz, Chloroform-*d*) δ -70.29 (d, *J* = 9.8 Hz).

HRMS (ESI) (m/z): $[M+H]^+$ Calcd for $C_{23}H_{29}ClF_3O^+$, 413.1854; Found 413.1860.

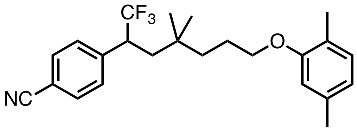


**4-(7-(2,5-dimethylphenoxy)-1,1,1-trifluoro-4,4-dimethylheptan-2-yl)benzonitrile (11)**

Prepared following the general procedure A. Purification by column chromatography (Eluent: petroleum ether/ethyl acetate = 50/1) provided the title compound (59.6 mg, 74% yield), as a yellow oil.

$^{1}$H NMR (400 MHz, Chloroform-*d*) δ 7.64 (m, 2H), 7.45 (m, 2H), 7.01 (dd, *J* = 7.5, 2.5 Hz, 1H), 6.67 (dd, *J* = 7.5, 1.7 Hz, 1H), 6.58 (d, *J* = 1.6 Hz, 1H), 3.91 – 3.74 (m, 2H), 3.48 – 3.36 (m, 1H), 2.31 (s, 3H), 2.17 (s, 3H), 2.03 – 1.89 (m, 1H), 1.77 – 1.65 (m, 1H), 1.66 – 1.53 (m, 1H), 1.45 – 1.20 (m, 3H), 0.78 (s, 6H).

$^{13}$C NMR (126 MHz, Chloroform-*d*) δ 156.9, 136.6, 132.4, 132.2, 130.4, 130.2, 129.2 (t, *J* = 2.5 Hz), 123.4, 120.8, 118.4, 112.0, 68.0, 46.8 (q, *J* = 26.5 Hz), 38.4, 33.1, 27.5, 27.4, 27.1, 24.0, 21.4, 15.8.

$^{19}$F NMR (376 MHz, Chloroform-*d*) δ -70.19 (d, *J* = 9.6 Hz).

HRMS (ESI) (m/z): $[M+H]^+$ Calcd for $C_{24}H_{29}F_3NO^+$, 404.2196; Found 404.2205.

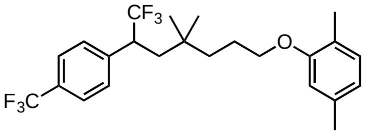


**1,4-dimethyl-2-((7,7,7-trifluoro-4,4-dimethyl-6-(4-(trifluoromethyl)phenyl)heptyl)oxy)benzene (12)**

Prepared following the general procedure A. Purification by column chromatography (Eluent: petroleum ether/ethyl acetate = 50/1) provided the title compound (81.2 mg, 91% yield), as a yellow oil.

$^{1}$H NMR (400 MHz, Chloroform-*d*) δ 7.61 (d, *J* = 8.0 Hz, 2H), 7.47 (d, *J* = 8.1 Hz, 2H), 7.01 (d, *J* = 7.5 Hz, 1H), 6.74 – 6.62 (m, 1H), 6.57 (d, *J* = 1.6 Hz, 1H), 3.91 – 3.68 (m, 2H), 3.50 – 3.32 (m, 1H), 2.30 (s, 3H), 2.17 (s, 3H), 2.06 – 1.90 (m, 2H), 1.79 – 1.66

(m, 1H), 1.65 – 1.53 (m, 1H), 1.43 – 1.34 (m, 1H), 1.31 – 1.17 (m, 1H), 0.85 – 0.48 (m, 6H).

$^{13}$C NMR (126 MHz, Chloroform-*d*) δ 156.9, 140.9, 136.6, 133.8 (d, *J* = 19.4 Hz), 130.3, 129.8, 128.7, 128.5 (d, *J* = 6.5 Hz), 125.6 (q, *J* = 3.9 Hz), 123.5, 120.8, 111.9, 68.1, 46.5 (q, *J* = 26.1 Hz), 40.0, 38.3, 33.1, 27.6, 27.4, 24.0, 21.4, 15.8.

$^{19}$F NMR (376 MHz, Chloroform-*d*) δ -62.61, -70.03 (d, *J* = 9.6 Hz).

HRMS (ESI) (m/z): $[M+H]^+$ Calcd for $C_{24}H_{29}F_6O^+$, 447.2117; Found 447.2126.

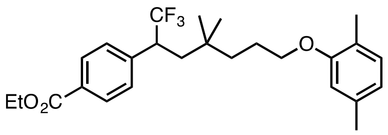


**ethyl 4-(7-(2,5-dimethylphenoxy)-1,1,1-trifluoro-4,4-dimethylheptan-2-yl)benzoate (13)**

Prepared following the general procedure A. Purification by column chromatography (Eluent: petroleum ether/ethyl acetate = 20/1) provided the title compound (75.6 mg, 84% yield), as a colorless oil.

$^{1}$H NMR (500 MHz, Chloroform-*d*) δ 8.02 (d, *J* = 8.4 Hz, 2H), 7.42 (d, *J* = 8.1 Hz, 2H), 7.00 (d, *J* = 7.5 Hz, 1H), 6.66 (dd, *J* = 7.5, 1.5 Hz, 1H), 6.57 (d, *J* = 1.5 Hz, 1H), 4.36 (q, *J* = 7.1 Hz, 2H), 3.82 – 3.75 (m, 2H), 3.51 – 3.34 (m, 1H), 2.30 (s, 3H), 2.16 (s, 3H), 2.02 – 1.92 (m, 2H), 1.77 – 1.66 (m, 1H), 1.63 – 1.51 (m, 1H), 1.38 (t, *J* = 7.2 Hz, 4H), 1.30 – 1.19 (m, 1H), 0.78 (d, *J* = 2.6 Hz, 6H).

$^{13}$C NMR (126 MHz, Chloroform-*d*) δ 166.2, 156.9, 141.7, 136.5, 130.3, 129.9, 129.5, 126.9 (d, *J* = 280.0 Hz), 128.1, 125.8, 123.5, 120.7, 112.0, 68.1, 61.1, 46.7 (q, *J* = 26.2 Hz), 40.0, 38.3, 33.1, 27.6, 27.4, 24.1, 21.4, 15.8, 14.3.

$^{19}$F NMR (471 MHz, Chloroform-*d*) δ -69.94 (d, *J* = 9.4 Hz).

HRMS (ESI) (m/z): $[M+H]^+$ Calcd for $C_{26}H_{34}F_3O_3^+$, 451.2455; Found 451.2459.

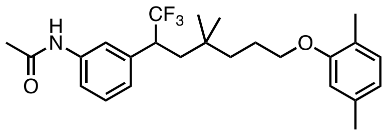


***N*-(3-(7-(2,5-dimethylphenoxy)-1,1,1-trifluoro-4,4-dimethylheptan-2-yl)phenyl)acetamide (14)**

Prepared following the general procedure A. Purification by column chromatography (Eluent: petroleum ether/ethyl acetate = 3/1) provided the title compound (75.7 mg, 87% yield), as a yellow solid.

$^{1}$H NMR (500 MHz, Chloroform-*d*) δ 7.53 – 7.44 (m, 2H), 7.40 (s, 1H), 7.31 – 7.22 (m, 1H), 7.07 (d, *J* = 7.7 Hz, 1H), 7.00 (d, *J* = 7.4 Hz, 1H), 6.65 (d, *J* = 7.4 Hz, 1H), 6.57 (d, *J* = 1.6 Hz, 1H), 3.76 (t, *J* = 6.4 Hz, 2H), 3.46 – 3.15 (m, 1H), 2.30 (s, 3H), 2.16 (s, 6H), 1.96 – 1.91 (m, 1H), 1.77 – 1.65 (m, 1H), 1.63 – 1.52 (m, 1H), 1.40 – 1.30 (m, 1H), 1.28 – 1.22 (m, 1H), 0.79 (d, *J* = 3.8 Hz, 6H).

$^{13}$C NMR (126 MHz, Chloroform-*d*) δ 168.5, 157.0, 138.2, 137.7, 136.5, 130.3, 129.2, 125.3, 123.5, 120.7, 120.6, 119.4, 112.0, 68.3, 46.6 (q, *J* = 26.4 Hz), 39.9, 38.2, 33.1, 27.6, 27.4, 24.6, 24.1, 21.4, 15.8.

$^{19}$F NMR (471 MHz, Chloroform-*d*) δ -70.05 (d, *J* = 10.1 Hz).

HRMS (ESI) (m/z): $[M+H]^+$ Calcd for $C_{25}H_{33}F_3NO_2^+$, 436.2458; Found 436.2466.

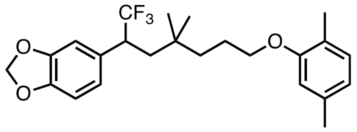


**5-(7-(2,5-dimethylphenoxy)-1,1,1-trifluoro-4,4-dimethylheptan-2-yl)benzo[*d*][1,3]dioxole (15)**

Prepared following the general procedure A. Purification by column chromatography (Eluent: petroleum ether/ethyl acetate = 20/1) provided the title compound (71.7 mg, 85% yield), as a yellow oil.

$^{1}$H NMR (400 MHz, Chloroform-*d*) δ 6.93 (d, *J* = 7.5 Hz, 1H), 6.74 (s, 1H), 6.69 (d, *J* = 2.3 Hz, 2H), 6.58 (d, *J* = 7.5 Hz, 1H), 6.50 (s, 1H), 5.84 (dd, *J* = 4.6, 1.6 Hz, 2H)

3.71 (t, $J$ = 6.4 Hz, 2H), 3.30 – 3.08 (m, 1H), 2.23 (s, 3H), 2.09 (s, 3H), 1.89 – 1.79 (m, 1H), 1.70 – 1.45 (m, 3H), 1.36 – 1.12 (m, 2H), 0.73 (s, 6H).

$^{13}$C NMR (151 MHz, Chloroform-*d*) δ 157.1, 147.9, 147.4, 136.6, 130.4, 123.6, 123.2, 120.8, 112.0, 109.3, 108.4, 101.3, 68.3, 46.3 (q, $J$ = 27.0 Hz), 40.0, 38.3, 33.2, 27.7, 27.5, 27.3, 24.2, 21.5, 15.9.

$^{19}$F NMR (376 MHz, Chloroform-*d*) δ -70.49 (d, $J$ = 9.7 Hz).

HRMS (ESI) (m/z): [M+H]$^+$ Calcd for $C_{24}H_{30}F_3O_3^+$, 423.2142; Found 423.2137.

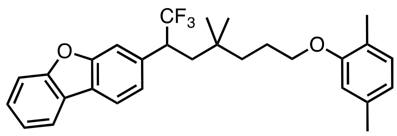


**3-(7-(2,5-dimethylphenoxy)-1,1,1-trifluoro-4,4-dimethylheptan-2-yl)dibenzo[*b*,*d*]furan (16)**

Prepared following the general procedure A. Purification by column chromatography (Eluent: petroleum ether/ethyl acetate = 20/1) provided the title compound (67.4 mg, 72% yield), as a white solid.

$^{1}$H NMR (500 MHz, Chloroform-*d*) δ 7.87 – 7.76 (m, 2H), 7.52 – 7.42 (m, 2H), 7.41 – 7.31 (m, 1H), 7.30 – 7.20 (m, 2H), 6.89 (d, $J$ = 7.4 Hz, 1H), 6.59 – 6.52 (m, 1H), 6.42 (d, $J$ = 1.5 Hz, 1H), 3.72 – 3.60 (m, 2H), 3.53 – 3.34 (m, 1H), 2.17 (s, 3H), 2.06 (s, 3H), 1.99 – 1.91 (m, 1H), 1.68 – 1.57 (m, 1H), 1.56 – 1.46 (m, 1H), 1.40 – 1.28 (m, 1H), 1.24 – 1.14 (m, 2H), 0.72 (d, $J$ = 2.5 Hz, 6H).

$^{13}$C NMR (126 MHz, Chloroform-*d*) δ 155.9, 155.5, 155.2, 135.4, 129.2, 126.3, 123.2, 123.1, 122.8, 122.5, 121.8, 119.7, 119.6, 119.6, 111.4, 110.9, 110.7, 67.1, 45.9 (q, $J$ = 26.2 Hz), 39.2, 37.2, 32.1, 26.6, 26.4, 26.2, 23.0, 20.3, 14.7.

$^{19}$F NMR (471 MHz, Chloroform-*d*) δ -70.02 (d, $J$ = 8.9 Hz).

HRMS (ESI) (m/z): [M+H]$^+$ Calcd for $C_{29}H_{32}F_3O_2^+$, 469.2349; Found 469.2355.

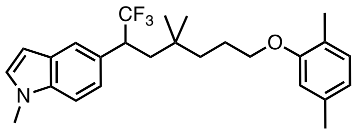

**5-(7-(2,5-dimethylphenoxy)-1,1,1-trifluoro-4,4-dimethylheptan-2-yl)-1-methyl-1*H*-indole (17)**

Prepared following the general procedure A. Purification by column chromatography (Eluent: petroleum ether/ethyl acetate = 10/1) provided the title compound (43.1 mg, 50% yield), as a white solid.

$^{1}$H NMR (500 MHz, Chloroform-*d*) δ 7.57 (d, *J* = 1.6 Hz, 1H), 7.30 – 7.23 (m, 1H), 7.20 – 7.14 (m, 1H), 7.02 (d, *J* = 3.2 Hz, 1H), 6.99 (d, *J* = 7.5 Hz, 1H), 6.64 (dd, *J* = 7.5, 1.5 Hz, 1H), 6.49 (d, *J* = 1.6 Hz, 1H), 6.45 (dd, *J* = 3.1, 0.9 Hz, 1H), 3.72 (s, 3H), 3.70 – 3.60 (m, 2H), 3.49 – 3.38 (m, 1H), 2.29 (s, 3H), 2.15 (s, 3H), 1.97 (dd, *J* = 14.3, 2.0 Hz, 1H), 1.70 (dd, *J* = 7.1, 5.6 Hz, 1H), 1.61 – 1.52 (m, 1H), 1.41 – 1.32 (m, 1H), 1.28 – 1.19 (m, 2H), 0.79 (s, 6H).

$^{13}$C NMR (126 MHz, Chloroform-*d*) δ 155.7, 135.1, 135.0, 128.9, 128.1, 127.2, 126.0, 122.2, 121.4, 120.5, 119.2, 110.5, 107.9, 99.6, 66.9, 45.3 (q, *J* = 25.9 Hz), 38.7, 36.8, 31.8, 31.5, 26.5, 26.2, 22.8, 20.1, 14.5.

$^{19}$F NMR (471 MHz, Chloroform-*d*) δ -70.25 (d, *J* = 10.3 Hz).

HRMS (ESI) (m/z): $[M+H]^+$ Calcd for $C_{26}H_{33}F_3NO^+$, 432.2509; Found 432.2517.

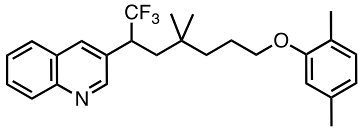

**3-(7-(2,5-dimethylphenoxy)-1,1,1-trifluoro-4,4-dimethylheptan-2-yl)quinoline (18)**

Prepared following the general procedure A. Purification by column chromatography (Eluent: petroleum ether/ethyl acetate = 10/1) provided the title compound (68.6 mg, 80% yield), as a white solid.

$^{1}$H NMR (500 MHz, Chloroform-*d*) δ 9.06 (d, *J* = 2.2 Hz, 1H), 8.37 – 8.24 (m, 2H), 7.99 (dd, *J* = 8.3, 1.4 Hz, 1H), 7.91 – 7.85 (m, 1H), 7.80 – 7.70 (m, 1H), 7.17 (d, *J* = 7.5 Hz, 1H), 6.82 (dd, *J* = 7.3, 1.6 Hz, 1H), 6.69 (d, *J* = 1.5 Hz, 1H), 3.93 (t, *J* = 6.3 Hz,

2H), 3.83 – 3.66 (m, 1H), 2.46 (s, 3H), 2.39 – 2.21 (m, 4H), 1.97 – 1.74 (m, 2H), 1.66 – 1.39 (m, 3H), 0.98 (d, *J* = 6.9 Hz, 6H).

$^{13}$C NMR (126 MHz, Chloroform-*d*) δ 156.9, 151.4, 147.7, 136.5, 136.2, 130.3, 130.0, 129.8, 129.2, 129.1, 127.8, 127.7 (d, *J* = 2.8 Hz), 127.2, 123.5, 120.8, 112.0, 68.1, 44.5 (q, *J* = 26.9 Hz), 40.0, 38.4, 33.2, 27.7, 27.5, 27.3, 24.3, 24.1, 21.4, 15.8.

$^{19}$F NMR (471 MHz, Chloroform-*d*) δ -69.97 (d, *J* = 10.2 Hz).

HRMS (ESI) (m/z): $[M+H]^+$ Calcd for $C_{26}H_{31}F_3NO^+$, 430.2352; Found 430.2360.

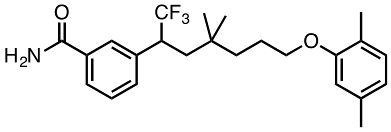


**3-(7-(2,5-dimethylphenoxy)-1,1,1-trifluoro-4,4-dimethylheptan-2-yl)benzamide (19)**

Prepared following the general procedure A. Purification by column chromatography (Eluent: petroleum ether/ethyl acetate = 3/1) provided the title compound (71.6 mg, 85% yield), as a white solid.

$^{1}$H NMR (400 MHz, Chloroform-*d*) δ 7.85 (d, *J* = 1.8 Hz, 1H), 7.69 (dt, *J* = 7.7, 1.4 Hz, 1H), 7.55 – 7.48 (m, 1H), 7.42 (t, *J* = 7.7 Hz, 1H), 7.00 (dd, *J* = 7.4, 0.8 Hz, 1H), 6.69 – 6.62 (m, 1H), 6.57 (d, *J* = 1.5 Hz, 1H), 6.15 (s, 2H), 3.77 (td, *J* = 6.4, 2.5 Hz, 2H), 3.42 (dt, *J* = 9.7, 5.6 Hz, 1H), 2.30 (s, 3H), 2.16 (s, 3H), 2.01 – 1.95 (m, 1H), 1.75 – 1.62 (m, 1H), 1.64 – 1.50 (m, 1H), 1.39 – 1.20 (m, 3H), 0.79 (d, *J* = 2.3 Hz, 6H).

$^{13}$C NMR (101 MHz, Chloroform-*d*) δ 169.1, 156.9, 137.6, 136.5, 133.8, 132.9, 130.3, 128.9, 128.7, 127.0 (q, *J* = 279.7 Hz), 123.5, 120.7, 112.0, 68.2, 46.5 (q, *J* = 26.4 Hz), 39.9, 38.2, 33.2, 27.6, 27.4, 24.0, 21.4, 15.8.

$^{19}$F NMR (376 MHz, Chloroform-*d*) δ -70.08 (d, *J* = 9.9 Hz).

HRMS (ESI) (m/z): $[M+Na]^+$ Calcd for $C_{24}H_{30}F_3NNaO_2^+$, 444.2121; Found 444.2130.

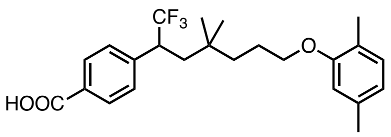

**4-(7-(2,5-dimethylphenoxy)-1,1,1-trifluoro-4,4-dimethylheptan-2-yl)benzoic acid (20)**

Prepared following the general procedure A. Purification by column chromatography (Eluent: petroleum ether/ethyl acetate = 3/1) provided the title compound (73.4 mg, 87% yield), as a colorless crystal.

$^{1}$H NMR (500 MHz, Chloroform-*d*) δ 8.21 – 7.99 (m, 2H), 7.47 (d, *J* = 8.1 Hz, 2H), 7.01 (d, *J* = 7.5 Hz, 1H), 6.65 (d, *J* = 7.5 Hz, 1H), 6.57 (d, *J* = 1.6 Hz, 1H), 4.04 – 3.58 (m, 2H), 3.48 – 3.23 (m, 1H), 2.30 (s, 3H), 2.17 (s, 3H), 2.02 – 2.00 (m, 1H), 1.79 – 1.69 (m, 1H), 1.65 – 1.56 (m, 1H), 1.26 (dd, *J* = 5.4, 2.3 Hz, 1H), 0.79 (d, *J* = 2.0 Hz, 6H).

$^{13}$C NMR (126 MHz, Chloroform-*d*) δ 170.6, 156.9, 142.8, 136.5, 130.5, 130.3, 129.6, 129.0, 123.5, 120.7, 111.9, 68.1, 40.0, 38.3, 33.1, 27.6, 27.4, 24.0, 21.4, 15.8.

$^{19}$F NMR (471 MHz, Chloroform-*d*) δ -69.87 (d, *J* = 10.0 Hz).

HRMS (ESI) (m/z): $[M-H]^-$ Calcd for $C_{24}H_{28}F_3O_3^-$, 421.1996; Found 421.1201.

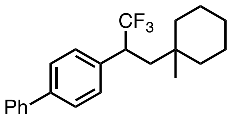

**4-(1,1,1-trifluoro-3-(1-methylcyclohexyl)propan-2-yl)-1,1'-biphenyl (21)**

Prepared following the general procedure A. Purification by column chromatography (petroleum ether) provided the title compound (63.7 mg, 92% yield), as a colorless oil.

$^{1}$H NMR (400 MHz, Chloroform-*d*) δ 7.76 – 7.57 (m, 4H), 7.54 – 7.35 (m, 5H), 3.55 – 3.35 (m, 1H), 2.06 – 1.99 (m, 2H), 1.54 – 1.41 (m, 3H), 1.40 – 1.28 (m, 5H), 1.13 (m, 2H), 0.82 (s, 3H).

$^{13}$C NMR (101 MHz, Chloroform-*d*) δ 139.5, 139.3, 134.9 (d, *J* = 2.0 Hz), 128.6, 127.6, 126.2, 126.0, 125.9, 44.6 (q, *J* = 26.1 Hz), 37.0, 36.8, 32.1, 25.0, 20.7, 20.6.

$^{19}$F NMR (376 MHz, Chloroform-*d*) δ -70.09 (d, *J* = 10.0 Hz).

HRMS (ESI) (m/z): $[M+H]^+$ Calcd for $C_{22}H_{26}F_3^+$, 347.1981; Found 347.1986.

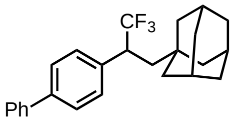


**(3*r*,5*r*,7*r*)-1-(2-([1,1'-biphenyl]-4-yl)-3,3,3-trifluoropropyl)adamantane (22)**

Prepared following the general procedure A. Purification by column chromatography (petroleum ether) provided the title compound (69.1 mg, 90% yield), as a white solid.

$^{1}$H NMR (400 MHz, Chloroform-*d*) δ 7.66 – 7.43 (m, 4H), 7.40 – 7.23 (m, 5H), 3.36 (m, 1H), 1.96 – 1.23 (m, 17H).

$^{13}$C NMR (101 MHz, Chloroform-*d*) δ 139.6, 139.4, 135.1, 128.7, 127.7, 126.3, 126.1, 126.0, 43.6 (q, *J* = 26.1 Hz), 41.8, 41.6, 35.8, 31.7, 27.4.

$^{19}$F NMR (376 MHz, Chloroform-*d*) δ -70.15 (d, *J* = 9.8 Hz).

HRMS (ESI) (m/z): $[M+Na]^+$ Calcd for $C_{25}H_{28}F_3^+$, 385.2138; Found 385.2140.

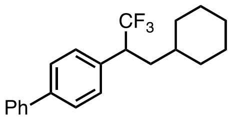


**4-(3-cyclohexyl-1,1,1-trifluoropropan-2-yl)-1,1'-biphenyl (23)**

Prepared following the general procedure A, using 2-(diphenylphosphanyl)pyridine (20 mol%, 0.04 mmol) instead of triphenylphosphine (20 mol%, 0.04 mmol). Purification by column chromatography (petroleum ether) provided the title compound (39.8 mg, 60% yield), as a white solid.

$^{1}$H NMR (600 MHz, Chloroform-*d*) δ 7.65 – 7.55 (m, 4H), 7.49 – 7.41 (m, 2H), 7.40 – 7.31 (m, 3H), 3.48 – 3.35 (m, 1H), 1.95 – 1.85 (m, 1H), 1.84 – 1.73 (m, 2H), 1.71 – 1.55 (m, 4H), 1.18 – 1.04 (m, 4H), 1.02 – 0.92 (m, 1H), 0.90 – 0.85 (m, 1H).

$^{13}$C NMR (126 MHz, Chloroform-*d*) δ 141.0, 140.7, 134.2, 129.6, 128.9, 127.6, 127.4, 127.2, 47.0 (q, *J* = 26.0 Hz), 36.1, 34.3, 34.0, 31.9, 29.9, 26.5, 26.2, 25.9.

$^{19}$F NMR (565 MHz, Chloroform-*d*) δ -69.66 (d, *J* = 10.7 Hz).

HRMS (ESI) (m/z): $[M+H]^+$ Calcd for $C_{21}H_{24}F_3^+$, 333.1825; Found 333.1819.

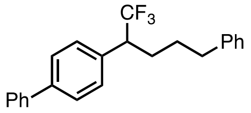

**4-(1,1,1-trifluoro-5-phenylpentan-2-yl)-1,1'-biphenyl (24)**

Prepared following the general procedure A, using 2-(diphenylphosphanyl)pyridine (20 mol%, 0.04 mmol) instead of triphenylphosphine (20 mol%, 0.04 mmol). Purification by column chromatography (petroleum ether) provided the title compound (46.0 mg, 65% yield), as a white solid.

$^1$H NMR (400 MHz, Chloroform-*d*) δ 7.64 (t, *J* = 8.4 Hz, 4H), 7.51 (t, *J* = 7.4 Hz, 2H), 7.40 (dd, *J* = 17.3, 7.7 Hz, 3H), 7.31 (d, *J* = 6.9 Hz, 2H), 7.24 (t, *J* = 7.1 Hz, 1H), 7.17 (d, *J* = 7.5 Hz, 2H), 3.47 – 3.17 (m, 1H), 2.76 – 2.59 (m, 2H), 2.21 – 2.06 (m, 1H), 2.08 – 1.95 (m, 1H), 1.69 – 1.54 (m, 2H).

$^{13}$C NMR (126 MHz, Chloroform-*d*) δ 140.4, 139.9, 139.3, 132.5, 128.2, 127.6, 127.2 (2C), 126.3, 126.2, 125.9, 124.8, 48.5 (q, *J* = 26.5 Hz), 34.3, 27.4, 27.1.

$^{19}$F NMR (471 MHz, Chloroform-*d*) δ -69.65 (d, *J* = 8.9 Hz).

HRMS (ESI) (m/z): $[M+H]^+$ Calcd for $C_{23}H_{22}F_3^+$, 355.1668; Found 355.1675.

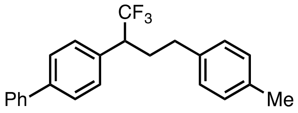

**4-(1,1,1-trifluoro-4-(*p*-tolyl)butan-2-yl)-1,1'-biphenyl (25)**

Prepared following the general procedure A, 2-(diphenylphosphanyl)pyridine (20 mol%) used, instead of triphenylphosphine (20 mol%). Purification by column chromatography (petroleum ether) provided the title compound (28.3 mg, 40% yield), as a colorless oil.

$^1$H NMR (400 MHz, Chloroform-*d*) δ 7.62 (dd, *J* = 8.3, 1.6 Hz, 2H), 7.50 – 7.41 (m, 2H), 7.41 – 7.32 (m, 3H), 7.10 (d, *J* = 7.8 Hz, 2H), 7.00 (d, *J* = 8.0 Hz, 2H), 3.32 – 3.20 (m, 1H), 2.64 – 2.55 (m, 1H), 2.46 – 2.30 (m, 5H), 2.29 – 2.19 (m, 1H).

$^{13}$C NMR (151 MHz, Chloroform-*d*) δ 141.2, 140.6, 137.5, 135.8, 133.6 – 133.4 (m), 129.7, 129.3, 128.9, 128.4, 127.6, 127.5, 127.2, 48.8 (q, *J* = 26.8 Hz), 32.1, 30.3, 21.1.

$^{19}$F NMR (376 MHz, Chloroform-*d*) δ -69.63 (d, *J* = 9.4 Hz).

HRMS (ESI) (m/z): $[M+H]^+$ Calcd for $C_{23}H_{22}F_3^+$, 355.1668; Found 355.1670.

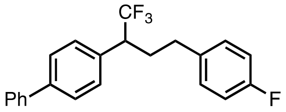


**4-(1,1,1-trifluoro-4-(4-fluorophenyl)butan-2-yl)-1,1'-biphenyl (26)**

Prepared following the general procedure A, 2-(diphenylphosphanyl)pyridine (20 mol%,) used, instead of triphenylphosphine (20 mol%). Purification by column chromatography (petroleum ether) provided the title compound (25.1 mg, 43% yield), as a colorless oil.

$^{1}$H NMR (400 MHz, Chloroform-*d*) δ 7.66 – 7.57 (m, 4H), 7.50 – 7.43 (m, 2H), 7.40 – 7.31 (m, 3H), 7.06 (dd, *J* = 8.6, 5.5 Hz, 2H), 7.02 – 6.92 (m, 2H), 3.34 – 3.15 (m, 1H), 2.69 – 2.55 (m, 1H), 2.49 – 2.17 (m, 3H).

$^{13}$C NMR (151 MHz, Chloroform-*d*) δ 161.5 (d, *J* = 244.2 Hz), 141.3, 140.5, 136.2 (d, *J* = 3.7 Hz), 133.3, 129.9 (d, *J* = 8.3 Hz), 129.6, 128.9, 127.6, 127.6, 127.2, 115.4 (d, *J* = 21.5 Hz), 48.8 (q, *J* = 26.9 Hz), 31.8, 30.3.

$^{19}$F NMR (376 MHz, Chloroform-*d*) δ -69.64 (d, *J* = 9.8 Hz), -116.98.

HRMS (ESI) (m/z): $[M+H]^+$ Calcd for $C_{22}H_{19}F_4^+$, 359.1417; Found 359.1425.

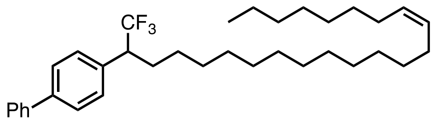


**(*Z*)-4-(1,1,1-trifluorotricos-15-en-2-yl)-1,1'-biphenyl (27)**

Prepared following the general procedure A, 2-(diphenylphosphanyl)pyridine (20 mol%) used, instead of triphenylphosphine (20 mol%). Purification by column chromatography (petroleum ether) provided the title compound (59.1 mg, 56% yield), as a white solid.

$^{1}$H NMR (400 MHz, Chloroform-*d*) δ 7.67 – 7.58 (m, 4H), 7.54 – 7.43 (m, 2H), 7.43 – 7.35 (m, 3H), 5.38 (t, *J* = 5.0 Hz, 2H), 3.39 – 3.16 (m, 1H), 2.09 – 1.98 (m, 5H), 1.37 – 1.20 (m, 31H), 0.92 (t, *J* = 6.8 Hz, 3H).

$^{13}C$ NMR (126 MHz, Chloroform-*d*) δ 139.7, 139.3, 128.7 (d, *J* = 3.2 Hz), 128.2, 127.6, 126.2, 126.1, 125.9, 48.6 (q, *J* = 26.2 Hz), 30.7, 28.6, 28.4 (3C), 28.3, 28.1 (2C), 28.0, 27.4, 26.0, 25.6, 21.5, 12.9.

$^{19}F$ NMR (376 MHz, Chloroform-*d*) δ -69.68 (d, *J* = 9.4 Hz).

HRMS (ESI) (m/z): $[M+H]^+$ Calcd for $C_{35}H_{52}F_3^+$, 529.4016; Found 529.4019.

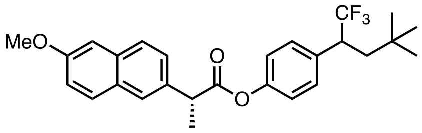


**4-(1,1,1-trifluoro-4,4-dimethylpentan-2-yl)phenyl (2*R*)-2-(6-methoxynaphthalen-2-yl)propanoate (28)**

Prepared following the general procedure A. Purification by column chromatography (Eluent: petroleum ether/ethyl acetate = 10/1) provided the title compound (77.9 mg, 85% yield), as a colorless oil.

$^{1}H$ NMR (500 MHz, Chloroform-*d*) δ 7.84 – 7.57 (m, 3H), 7.49 (dd, *J* = 8.4, 1.9 Hz, 1H), 7.27 (d, *J* = 8.4 Hz, 2H), 7.13 (s, 2H), 6.98 (d, *J* = 8.6 Hz, 2H), 4.09 (q, *J* = 7.1 Hz, 1H), 3.91 (s, 3H), 3.29 (qt, *J* = 9.8, 4.9 Hz, 1H), 2.04 – 1.78 (m, 2H), 1.68 (d, *J* = 7.1 Hz, 3H), 0.76 (s, 9H).

$^{13}C$ NMR (101 MHz, Chloroform-*d*) δ 173.0, 157.7, 150.5, 135.0, 134.2, 133.8, 130.2, 129.3, 129.0, 127.4, 126.1 (d, *J* = 6.0 Hz), 121.4, 119.1, 105.6, 55.3, 46.4 (q, *J* = 26.4 Hz), 45.6, 30.8, 29.7, 18.4.

$^{19}F$ NMR (471 MHz, Chloroform-*d*) δ -70.39 (d, *J* = 8.7 Hz).

HRMS (ESI) (m/z): $[M+H]^+$ Calcd for $C_{27}H_{30}F_3O_3^+$, 459.2142; Found 459.2144.

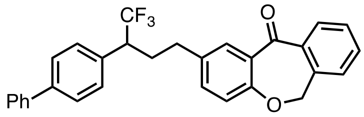


**2-(3-([1,1'-biphenyl]-4-yl)-4,4,4-trifluorobutyl)dibenzo[*b,e*]oxepin-11(6*H*)-one (29)**

Prepared following the general procedure A, 2-(diphenylphosphanyl)pyridine (20 mol%) used, instead of triphenylphosphine (20 mol%). Purification by column

chromatography (Eluent: petroleum ether/ethyl acetate = 10/1) provided the title compound (66.1 mg, 70% yield), as a colorless oil.

$^{1}$H NMR (600 MHz, Chloroform-*d*) δ 7.97 (d, *J* = 2.4 Hz, 1H), 7.89 (dd, *J* = 7.8, 1.4 Hz, 1H), 7.66 – 7.57 (m, 4H), 7.60 – 7.50 (m, 1H), 7.51 – 7.41 (m, 3H), 7.42 – 7.31 (m, 4H), 7.23 (dd, *J* = 8.4, 2.4 Hz, 1H), 6.98 (d, *J* = 8.4 Hz, 1H), 5.17 (s, 2H), 3.35 – 3.22 (m, 1H), 2.67 – 2.57 (m, 1H), 2.55 – 2.45 (m, 1H), 2.41 – 2.31 (m, 1H), 2.30 – 2.17 (m, 1H).

$^{13}$C NMR (151 MHz, Chloroform-*d*) δ 191.2, 160.0, 141.3, 140.6, 135.8, 135.7, 134.4, 132.9, 131.3, 129.6 (2C), 129.3, 128.9, 127.9, 127.6 (2C), 127.2, 125.2, 121.0, 73.7, 49.1 (q, *J* = 27.0 Hz), 31.8, 30.3.

$^{19}$F NMR (565 MHz, Chloroform-*d*) δ -69.46 (d, *J* = 10.0 Hz).

HRMS (ESI) (m/z): $[M+H]^+$ Calcd for $C_{30}H_{24}F_3O_2^+$, 473.1723; Found 473.1721.

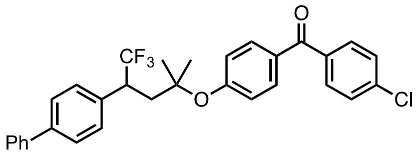


**(4-((4-([1,1'-biphenyl]-4-yl)-5,5,5-trifluoro-2-methylpentan-2-yl)oxy)phenyl)(4-chlorophenyl)methanone (30)**

Prepared following the general procedure A. Purification by column chromatography (petroleum ether) provided the title compound (63.7 mg, 61% yield), as a white solid.

$^{1}$H NMR (400 MHz, Chloroform-*d*) δ 7.68 – 7.61 (m, 4H), 7.54 (ddd, *J* = 6.4, 3.0, 1.7 Hz, 4H), 7.42 – 7.36 (m, 6H), 7.35 – 7.27 (m, 1H), 6.88 – 6.80 (m, 2H), 3.96 – 3.54 (m, 1H), 2.50 – 2.36 (m, 2H), 1.34 (s, 3H), 1.16 (s, 3H).

$^{13}$C NMR (151 MHz, Chloroform-*d*) δ 194.5, 159.6, 141.1, 140.4, 138.6, 136.3, 135.1, 131.7, 131.5, 131.3, 129.9, 128.9, 128.6, 127.6, 127.4, 127.1, 121.7, 80.4, 46.0 (q, *J* = 27.5 Hz), 41.6, 27.6, 27.1.

$^{19}$F NMR (376 MHz, Chloroform-*d*) δ -69.88 (d, *J* = 10.0 Hz).

HRMS (ESI) (m/z): $[M+H]^+$ Calcd for $C_{31}H_{27}ClF_3O_2^+$, 523.1646; Found 523.1645.

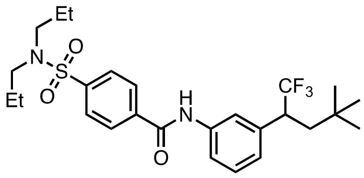


**4-(*N*,*N*-dipropylsulfamoyl)-*N*-(3-(1,1,1-trifluoro-4,4-dimethylpentan-2-yl)phenyl)benzamide (31)**

Prepared following the general procedure A. Purification by column chromatography (Eluent: petroleum ether/ethyl acetate = 3/1) provided the title compound (79.9 mg, 78% yield), as a colorless oil.

$^1$H NMR (400 MHz, Chloroform-*d*) δ 8.68 (s, 1H), 7.97 (d, *J* = 8.0 Hz, 2H), 7.84 – 7.69 (m, 4H), 7.40 (t, *J* = 8.2 Hz, 1H), 7.19 (d, *J* = 7.7 Hz, 1H), 3.40 (m, 1H), 3.18 – 3.03 (m, 4H), 2.05 – 1.86 (m, 2H), 1.58 (q, *J* = 7.5 Hz, 4H), 1.01 – 0.79 (m, 15H).

$^{13}$C NMR (101 MHz, Chloroform-*d*) δ 163.7, 141.2, 137.7, 137.0, 136.6 (d, *J* = 1.8 Hz), 128.0, 126.9, 125.9, 124.5, 120.0, 118.5, 48.7, 45.8 (q, *J* = 26.5 Hz), 40.8, 29.6, 28.5, 20.7, 9.9.

$^{19}$F NMR (376 MHz, Chloroform-*d*) δ -70.14 (d, *J* = 9.8 Hz).

HRMS (ESI) (m/z): $[M+H]^+$ Calcd for $C_{26}H_{36}F_3N_2O_3S^+$, 513.2393; Found 513.2395.

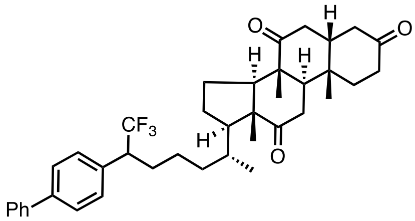


**(5*S*,8*R*,9*R*,10*S*,13*R*,14*R*,17*R*)-17-((2*R*)-6-([1,1'-biphenyl]-4-yl)-7,7,7-trifluoroheptan-2-yl)-8,10,13-trimethyldodecahydro-3*H*-cyclopenta[*a*]phenanthrene-3,7,12(2*H*,4*H*)-trione (32)**

Prepared following the general procedure A, 2-(diphenylphosphanyl)pyridine (20 mol%) used, instead of triphenylphosphine (20 mol%). Purification by column chromatography (ethyl acetate) provided the title compound (83.1 mg, 67% yield), as a white solid.

$^{1}$H NMR (400 MHz, Chloroform-*d*) δ 7.56 – 7.49 (m, 4H), 7.38 (t, *J* = 7.6 Hz, 2H), 7.32 – 7.25 (m, 3H), 3.27 – 3.13 (m, 1H), 2.89 – 2.71 (m, 4H), 2.32 – 2.12 (m, 8H), 2.09 – 2.01 (m, 2H), 1.98 – 1.85 (m, 7H), 1.32 (s, 3H), 1.25 – 1.14 (m, 3H), 1.11 – 1.04 (m, 2H), 0.96 (d, *J* = 2.9 Hz, 3H), 0.78 (dd, *J* = 16.8, 6.8 Hz, 2H), 0.68 (dd, *J* = 10.9, 6.1 Hz, 3H).

$^{13}$C NMR (126 MHz, Chloroform-*d*) δ 212.1, 209.2, 208.8, 140.9, 140.5, 129.4 (2C), 128.8, 127.4, 127.3, 127.1, 57.4 – 56.3 (m), 51.8 (t, *J* = 4.1 Hz), 49.0, 46.9, 45.8 (q, *J* = 28.5 Hz), 45.6, 45.0, 42.8, 38.6, 37.4, 36.5, 36.0, 35.8 (2C), 35.3, 35.1, 34.9, 28.0 – 27.8 (m), 25.2, 23.8, 21.9, 19.0, 18.8.

$^{19}$F NMR (376 MHz, Chloroform-*d*) δ -69.67 (dd, *J* = 40.8, 9.2 Hz).

HRMS (ESI) (m/z): $[M+H]^+$ Calcd for $C_{39}H_{48}F_3O_3^+$, 621.3550; Found 621.3553.

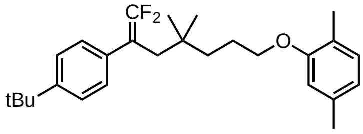


**2-((6-(4-(tert-butyl)phenyl)-7,7-difluoro-4,4-dimethylhept-6-en-1-yl)oxy)-1,4-dimethylbenzene (33)**

Prepared following the general procedure B. Purification by column chromatography (petroleum ether/ethyl acetate = 20:1) provided the title compound (76.1 mg, 92% yield), as a colorless oil.

$^{1}$H NMR (400 MHz, Chloroform-*d*) δ 7.36 – 7.29 (m, 2H), 7.23 (dt, *J* = 8.7, 2.0 Hz, 2H), 6.98 (d, *J* = 7.4 Hz, 1H), 6.63 (d, *J* = 7.1 Hz, 1H), 6.53 (s, 1H), 3.65 (t, *J* = 6.5 Hz, 2H), 2.36 (t, *J* = 4.5, 2.3 Hz, 2H), 2.28 (s, 3H), 2.15 (s, 3H), 1.69 – 1.57 (m, 2H), 1.28 (s, 9H), 1.29 – 1.20 (m, 2H), 0.81 (s, 6H).

$^{13}$C NMR (101 MHz, Chloroform-*d*) δ 157.1, 154.4 (d, *J* = 2.6 Hz), 150.0, 136.4, 132.4 (dd, *J* = 4.5, 2.6 Hz), 130.3, 128.1 (t, *J* = 2.7 Hz), 125.2, 123.6, 120.6, 111.9, 90.6 (dd, *J* = 21.3, 13.4 Hz), 68.4, 39.1, 38.2, 35.0 (t, *J* = 2.5 Hz), 34.5, 31.3, 27.4, 24.3, 21.4, 15.8.

$^{19}$F NMR (376 MHz, Chloroform-*d*) δ -89.92 (d, *J* = 41.9 Hz), -92.26 (d, *J* = 41.9 Hz).

HRMS (ESI) (m/z): $[M+H]^+$ Calcd for $C_{27}H_{37}F_2O^+$, 415.2807; Found 415.2811.

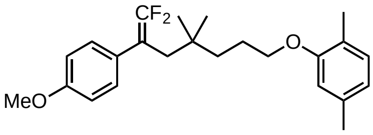


**2-((7,7-difluoro-6-(4-methoxyphenyl)-4,4-dimethylhept-6-en-1-yl)oxy)-1,4-dimethylbenzene (34)**

Prepared following the general procedure B. Purification by column chromatography (petroleum ether/ethyl acetate = 10:1) provided the title compound (65.2 mg, 84% yield), as a colorless oil.

$^1$H NMR (400 MHz, Chloroform-*d*) δ 7.22 (dd, *J* = 8.8, 1.5 Hz, 2H), 7.00 (d, *J* = 7.4 Hz, 1H), 6.86 (d, *J* = 8.7 Hz, 2H), 6.65 (d, *J* = 7.4 Hz, 1H), 6.56 (s, 1H), 3.76 (s, 3H), 3.73 (t, *J* = 6.5 Hz, 2H), 2.33 (t, *J* = 2.3 Hz, 2H), 2.30 (s, 3H), 2.16 (s, 3H), 1.73 – 1.61 (m, 2H), 1.32 – 1.24 (m, 2H), 0.79 (s, 6H).

$^{13}$C NMR (126 MHz, Chloroform-*d*) δ 170.2 – 167.5 (m), 156.9, 156.7, 154.7 (t, *J* = 290.6 Hz), 154.7, 129.9, 129.2 (t, *J* = 3.5 Hz), 127.5 (t, *J* = 3.7 Hz), 123.7, 119.3, 118.6, 86.6 (dd, *J* = 21.8, 17.8 Hz), 79.7, 70.5, 34.2 (2C), 30.4, 28.4.

$^{19}$F NMR (376 MHz, Chloroform-*d*) δ -88.2 (d, *J* = 36.2 Hz), -89.4 (d, *J* = 36.3 Hz).

HRMS (ESI) (m/z): $[M+H]^+$ Calcd for $C_{24}H_{31}F_2O_2^+$, 389.2287; Found 389.2290.

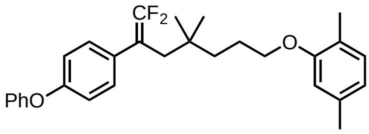


**2-((7,7-difluoro-4,4-dimethyl-6-(4-phenoxyphenyl)hept-6-en-1-yl)oxy)-1,4-dimethylbenzene (35)**

Prepared following the general procedure B. Purification by column chromatography (petroleum ether/ethyl acetate = 20:1) provided the title compound (60.3 mg, 67% yield), as a colorless oil.

$^1$H NMR (400 MHz, Chloroform-*d*) δ 7.35 – 7.23 (m, 4H), 7.14 – 7.05 (m, 1H), 7.03 – 6.90 (m, 5H), 6.65 (d, *J* = 7.5 Hz, 1H), 6.58 (s, 1H), 3.76 (t, *J* = 6.5 Hz, 2H), 2.38 – 2.32 (m, 2H), 2.30 (s, 3H), 2.16 (s, 3H), 1.73 – 1.61 (m, 2H), 1.34 – 1.25 (m, 2H), 0.81 (s, 6H).

$^{13}C$ NMR (101 MHz, Chloroform-*d*) δ 157.0, 156.9, 156.3, 153.6 (dd, *J* = 290.0, 288.1 Hz), 136.5, 130.3, 129.8 (t, *J* = 3.0 Hz), 129.8, 123.6, 123.5, 120.6, 119.1, 118.5, 112.0, 90.2 (dd, *J* = 21.7, 13.5 Hz), 68.4, 39.5, 38.4, 35.0 (t, *J* = 2.5 Hz), 27.3, 24.3, 21.4, 15.8.

$^{19}F$ NMR (376 MHz, Chloroform-*d*) δ -89.80 (d, *J* = 41.6 Hz), -92.14 (d, *J* = 41.6 Hz).

HRMS (ESI) (m/z): $[M+H]^+$ Calcd for $C_{29}H_{33}F_2O_2^+$, 451.2443; Found 451.2449.

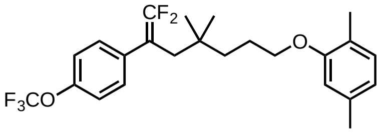


**2-((7,7-difluoro-4,4-dimethyl-6-(4-(trifluoromethoxy)phenyl)hept-6-en-1-yl)oxy)-1,4-dimethylbenzene (36)**

Prepared following the general procedure B. Purification by column chromatography (petroleum ether/ethyl acetate = 20:1) provided the title compound (53.0 mg, 60% yield), as a colorless oil.

$^{1}H$ NMR (400 MHz, Chloroform-*d*) δ 7.39 – 7.29 (m, 3H), 7.18 (d, *J* = 7.7 Hz, 2H), 7.00 (d, *J* = 7.5 Hz, 1H), 6.65 (d, *J* = 7.5 Hz, 1H), 6.57 (s, 1H), 3.75 (t, *J* = 6.4 Hz, 2H), 2.37 (t, *J* = 2.4 Hz, 2H), 2.30 (s, 3H), 2.16 (s, 3H), 1.73 – 1.56 (m, 2H), 1.34 – 1.23 (m, 2H), 0.79 (s, 6H).

$^{13}C$ NMR (101 MHz, Chloroform-*d*) δ 157.0, 154.5 (dd, *J* = 290.6, 288.3 Hz), 148.1 (d, *J* = 2.0 Hz), 136.5, 134.4 – 134.2 (m), 133.9, 133.7, 130.3, 129.9 (t, *J* = 2.9 Hz), 128.7, 128.6, 128.5, 123.9 (q, *J* = 272.1 Hz), 123.5, 120.8, 120.6, 111.9, 89.9 (dd, *J* = 22.4, 13.2 Hz), 68.3, 39.5, 38.5, 35.1 (t, *J* = 2.5 Hz), 27.2, 24.3, 21.4, 15.8.

$^{19}F$ NMR (376 MHz, Chloroform-*d*) δ -57.85, -88.63 (d, *J* = 38.9 Hz), -91.22 (d, *J* = 38.9 Hz).

HRMS (ESI) (m/z): $[M+H]^+$ Calcd for $C_{24}H_{28}F_5O_2^+$,443.2004; Found 443.2008.

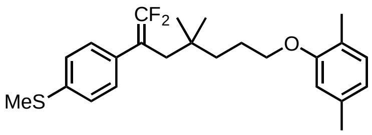


**(4-(7-(2,5-dimethylphenoxy)-1,1-difluoro-4,4-dimethylhept-1-en-2-yl)phenyl)(methyl)sulfane (37)**

Prepared following the general procedure B. Purification by column chromatography (petroleum ether/ethyl acetate = 20:1) provided the title compound (75.1 mg, 93% yield), as a colorless oil.

$^{1}$H NMR (400 MHz, Chloroform-*d*) δ 7.26 – 7.16 (m, 4H), 6.99 (d, *J* = 7.4 Hz, 1H), 6.64 (d, *J* = 7.5 Hz, 1H), 6.57 (s, 1H), 3.73 (t, *J* = 6.5 Hz, 2H), 2.43 (s, 3H), 2.34 (t, 2H), 2.30 (s, 3H), 2.16 (s, 3H), 1.73 – 1.56 (m, 2H), 1.33 – 1.24 (m, 2H), 0.79 (s, 6H).

$^{13}$C NMR (101 MHz, Chloroform-*d*) δ 157.0, 154.4 (dd, *J* = 290.1, 287.9 Hz), 137.3, 136.5, 132.2 (dd, *J* = 4.7, 2.6 Hz), 130.3, 128.9 (t, *J* = 2.8 Hz), 126.3, 123.6, 120.6, 112.0, 90.3 (dd, *J* = 21.6, 13.2 Hz), 68.4, 39.3, 38.4, 35.1 (t, *J* = 2.5 Hz), 27.3, 24.3, 21.4, 15.8, 15.6.

$^{19}$F NMR (376 MHz, Chloroform-*d*) δ -89.35 (d, *J* = 40.6 Hz), -91.70 (d, *J* = 40.7 Hz).

HRMS (ESI) (m/z): $[M+H]^+$ Calcd for $C_{24}H_{31}F_2OS^+$, 405.2058; Found 405.2063.

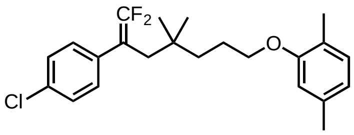


**2-((6-(4-chlorophenyl)-7,7-difluoro-4,4-dimethylhept-6-en-1-yl)oxy)-1,4-dimethylbenzene (38)**

Prepared following the general procedure B. Purification by column chromatography (petroleum ether/ethyl acetate = 20:1) provided the title compound (51.0 mg, 65% yield), as a colorless oil.

$^{1}$H NMR (400 MHz, Chloroform-*d*) δ 7.31 (t, *J* = 4.0 Hz, 2H), 7.27 – 7.20 (m, 2H), 7.00 (d, *J* = 7.4 Hz, 1H), 6.66 (s, 1H), 6.58 (s, 1H), 3.77 (t, *J* = 6.4 Hz, 2H), 2.35 (t, *J* = 2.4 Hz, 2H), 2.30 (s, 3H), 2.16 (s, 3H), 1.86 – 1.62 (m, 2H), 1.35 – 1.21 (m, 2H), 0.78 (s, 6H).

$^{13}$C NMR (126 MHz, Chloroform-*d*) δ 157.0, 156.7 – 152.0 (m), 136.5, 134.1 (dd, *J* = 5.0, 2.8 Hz), 132.9, 130.3, 129.8 (t, *J* = 2.9 Hz), 128.6, 123.6, 120.7, 90.0 (dd, *J* = 22.1, 13.2 Hz), 68.4, 39.4, 38.5, 35.1 (t, *J* = 2.5 Hz), 27.2, 24.3, 21.4, 15.8.

$^{19}$F NMR (376 MHz, Chloroform-*d*) δ -88.73 (d, *J* = 39.4 Hz), -91.16 (d, *J* = 39.0 Hz).

HRMS (ESI) (m/z): $[M+H]^+$ Calcd for $C_{23}H_{28}ClF_2O^+$, 393.1791; Found 393.1799.

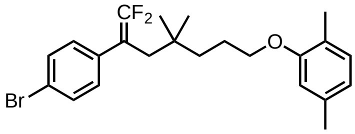


**2-((6-(4-bromophenyl)-7,7-difluoro-4,4-dimethylhept-6-en-1-yl)oxy)-1,4-dimethylbenzene (39)**

Prepared following the general procedure B. Purification by column chromatography (petroleum ether/ethyl acetate = 20:1) provided the title compound (35.4 mg, 81% yield), as a colorless oil.

$^{1}$H NMR (400 MHz, Chloroform-*d*) δ 7.49 – 7.42 (m, 2H), 7.18 (dd, *J* = 8.4, 1.5 Hz, 2H), 7.00 (d, *J* = 7.4 Hz, 1H), 6.66 (dd, *J* = 7.5, 1.5 Hz, 1H), 6.58 (s, 1H), 3.77 (t, *J* = 6.4 Hz, 2H), 2.35 (t, *J* = 2.5 Hz, 2H), 2.31 (s, 3H), 2.16 (s, 3H), 1.72 – 1.64 (m, 2H), 1.32 – 1.26 (m, 2H), 0.78 (s, 6H).

$^{13}$C NMR (126 MHz, Chloroform-*d*) δ 157.0, 136.5, 134.6 (dd, *J* = 4.6, 2.7 Hz), 131.5, 130.3, 130.2 (t, *J* = 2.4 Hz), 123.6, 121.0, 120.7, 112.0, 90.3 – 89.9 (m), 68.4, 39.4, 38.5, 35.1, 27.2, 24.3, 21.5, 15.8.

$^{19}$F NMR (376 MHz, Chloroform-*d*) δ -89.28 (d, *J* = 39.5 Hz), -91.58 (d, *J* = 39.9 Hz).

HRMS (ESI) (m/z): $[M+H]^+$ Calcd for $C_{23}H_{28}BrF_2O^+$, 437.1286; Found 437.1288.

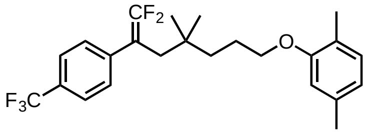

**2-((7,7-difluoro-4,4-dimethyl-6-(4-(trifluoromethyl)phenyl)hept-6-en-1-yl)oxy)-1,4-dimethylbenzene (40)**

Prepared following the general procedure B. Purification by column chromatography (petroleum ether/ethyl acetate = 20:1) provided the title compound (78.4 mg, 92% yield), as a colorless oil.

$^1$H NMR (400 MHz, Chloroform-*d*) δ 7.59 (d, *J* = 8.1 Hz, 2H), 7.43 (d, *J* = 8.1 Hz, 2H), 7.00 (d, *J* = 7.4 Hz, 1H), 6.66 (dd, *J* = 7.4, 1.5 Hz, 1H), 6.57 (d, *J* = 1.5 Hz, 1H), 3.76 (t, *J* = 6.4 Hz, 2H), 2.40 (t, *J* = 2.4 Hz, 2H), 2.30 (s, 3H), 2.15 (s, 3H), 1.75 – 1.61 (m, 2H), 1.36 – 1.26 (m, 2H), 0.79 (s, 6H).

$^{13}$C NMR (126 MHz, Chloroform-*d*) δ 157.0, 154.6 (dd, *J* = 292.0, 289.2 Hz), 139.5, 136.5, 130.3, 128.8 (t, *J* = 2.7 Hz), 125.3 (q, *J* = 3.7 Hz), 123.5, 120.7, 111.9, 90.2 (dd, *J* = 22.6, 12.7 Hz), 68.3, 39.3, 38.5, 35.1 (t, *J* = 2.2 Hz), 29.7, 27.1, 24.3, 21.4, 15.8.

$^{19}$F NMR (376 MHz, Chloroform-*d*) δ -62.53, -87.53 (d, *J* = 36.8 Hz), -90.30 (d, *J* = 36.6 Hz).

HRMS (ESI) (m/z): $[M+H]^+$ Calcd for $C_{24}H_{28}F_5O^+$, 427.2055; Found 427.2057.

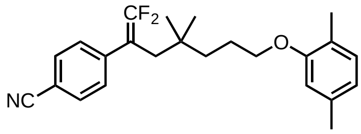

**4-(7-(2,5-dimethylphenoxy)-1,1-difluoro-4,4-dimethylhept-1-en-2-yl)benzonitrile (41)**

Prepared following the general procedure B. Purification by column chromatography (petroleum ether/ethyl acetate = 20:1) provided the title compound (64.3 mg, 84% yield), as a colorless oil.

$^1$H NMR (400 MHz, Chloroform-*d*) δ 7.68 – 7.59 (m, 2H), 7.43 (dd, *J* = 8.4, 1.6 Hz, 2H), 7.01 (d, *J* = 7.4 Hz, 1H), 6.66 (d, *J* = 7.5 Hz, 1H), 6.58 (s, 1H), 3.80 (t, *J* = 6.3 Hz,

2H), 2.43 – 2.37 (m, 2H), 2.31 (s, 3H), 2.15 (s, 3H), 1.75 – 1.63 (m, 2H), 1.35 – 1.23 (m, 2H), 0.78 (s, 6H).

$^{13}$C NMR (101 MHz, Chloroform-*d*) δ 157.0, 156.9 – 151.3 (m), 140.7 (dd, *J* = 5.2, 3.0 Hz), 136.5, 132.2, 130.3, 129.2 (t, *J* = 3.0 Hz), 123.5, 120.7, 118.6, 112.0, 110.8, 90.3 (dd, *J* = 22.8, 12.2 Hz), 68.3, 39.3, 38.6, 35.2 (t, *J* = 2.5 Hz), 27.1, 24.3, 21.4, 15.8.

$^{19}$F NMR (376 MHz, Chloroform-*d*) δ -86.23 (d, *J* = 33.6 Hz), -89.12 (d, *J* = 33.6 Hz).

HRMS (ESI) (m/z): $[M+H]^+$ Calcd for $C_{24}H_{28}F_2NO^+$, 384.2133; Found 384.2137.

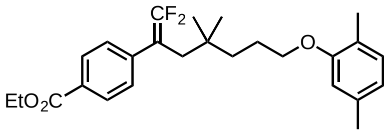


**ethyl 4-(7-(2,5-dimethylphenoxy)-1,1-difluoro-4,4-dimethylhept-1-en-2-yl)benzoate (42)**

Purification by column chromatography ((petroleum ether/ethyl acetate = 10:1) provided the title compound (75.7 mg, 88% yield), as a colorless oil.

$^{1}$H NMR (400 MHz, Chloroform-*d*) δ 8.02 (d, *J* = 8.5 Hz, 2H), 7.40 (dd, *J* = 8.4, 1.6 Hz, 2H), 7.00 (d, *J* = 7.4 Hz, 1H), 6.65 (d, *J* = 7.5 Hz, 1H), 6.57 (s, 1H), 4.37 (q, *J* = 7.1 Hz, 2H), 3.77 (t, *J* = 6.4 Hz, 2H), 2.41 (t, *J* = 2.4 Hz, 25H), 2.30 (s, 3H), 2.16 (s, 3H), 1.75 – 1.64 (m, 2H), 1.39 (t, *J* = 7.1 Hz, 3H), 1.35 – 1.27 (m, 2H), 0.78 (s, 6H).

$^{13}$C NMR (101 MHz, Chloroform-*d*) δ 166.2, 157.0, 154.4 (dd, *J* = 290.6, 288.3 Hz), 140.4 (dd, *J* = 5.0, 2.9 Hz), 136.5, 130.3, 129.6, 129.1, 128.4 (t, *J* = 2.9 Hz), 123.6, 120.6, 112.0, 90.6 (dd, *J* = 22.1, 12.8 Hz), 68.3, 61.0, 39.3, 38.5, 35.1, 29.7, 27.1, 24.3, 21.4, 15.8, 14.3.

$^{19}$F NMR (376 MHz, Chloroform-*d*) δ -87.61 (d, *J* = 36.4 Hz), -90.09 (d, *J* = 36.3 Hz).

HRMS (ESI) (m/z): $[M+H]^+$ Calcd for $C_{26}H_{33}F_2O_3^+$, 431.2392; Found 431.2399.

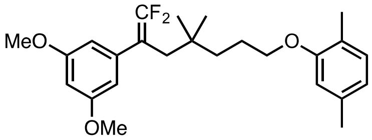


**2-((6-(3,5-dimethoxyphenyl)-7,7-difluoro-4,4-dimethylhept-6-en-1-yl)oxy)-1,4-dimethylbenzene (43)**

Purification by column chromatography ((petroleum ether/ethyl acetate = 10:1) provided the title compound (61.9 mg, 74% yield), as a colorless oil.

$^1$H NMR (400 MHz, Chloroform-*d*) δ 6.99 (d, *J* = 7.5 Hz, 1H), 6.64 (d, *J* = 7.5 Hz, 1H), 6.57 (d, *J* = 1.5 Hz, 1H), 6.46 (dd, *J* = 2.3, 1.3 Hz, 2H), 6.34 (t, *J* = 2.3 Hz, 1H), 3.76 (s, 6H), 2.33 (t, *J* = 2.5 Hz, 2H), 2.30 (s, 3H), 2.16 (s, 3H), 1.73 – 1.62 (m, 2H), 1.41 – 1.24 (m, 2H), 0.82 (s, 6H).

$^{13}$C NMR (101 MHz, Chloroform-*d*) δ 160.5, 157.0, 136.4, 130.2, 123.6, 120.6, 112.0, 107.0 (t, *J* = 2.8 Hz), 98.8, 90.8 (dd, *J* = 21.8, 13.1 Hz), 68.4, 55.3, 39.4, 38.3, 35.0, 27.1, 24.3, 21.4, 15.6.

$^{19}$F NMR (376 MHz, Chloroform-*d*) δ -89.17 (d, *J* = 39.5 Hz), -90.49 (d, *J* = 39.7 Hz).

HRMS (ESI) (m/z): $[M+H]^+$ Calcd for $C_{25}H_{33}F_2O_3^+$, 419.2392; Found 419.2395.

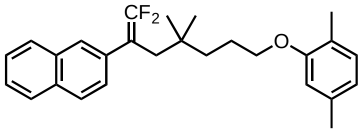


**2-(7-(2,5-dimethylphenoxy)-1,1-difluoro-4,4-dimethylhept-1-en-2-yl)naphthalene (44)**

Prepared following the general procedure B. Purification by column chromatography (petroleum ether/ethyl acetate = 20:1) provided the title compound (73.5 mg, 90% yield), as a colorless oil.

$^1$H NMR (400 MHz, Chloroform-*d*) δ 7.96 – 7.77 (m, 4H), 7.50 (td, *J* = 6.0, 3.2 Hz, 3H), 7.03 (d, *J* = 7.5 Hz, 1H), 6.69 (d, *J* = 7.5 Hz, 1H), 6.52 (s, 1H), 3.71 (t, *J* = 6.5 Hz, 2H), 2.54 (t, *J* = 2.4 Hz, 2H), 2.33 (s, 3H), 2.18 (s, 3H), 1.89 – 1.58 (m, 2H), 1.54 – 1.12 (m, 2H), 0.86 (s, 6H).

$^{13}$C NMR (126 MHz, Chloroform-*d*) δ 157.1, 154.7 (dd, *J* = 290.3, 288.0 Hz), 136.5, 133.3, 133.0 (dd, *J* = 4.8, 2.6 Hz), 132.4, 130.3, 128.0, 127.9, 127.7, 127.5 (t, *J* = 2.9 Hz), 126.5 (t, *J* = 2.7 Hz), 126.3, 126.1, 123.6, 120.6, 112.0, 91.0 (dd, *J* = 21.5, 13.2 Hz), 68.4, 39.5, 38.5, 35.2 (t, *J* = 2.5 Hz), 27.3, 24.3, 21.5, 15.8.

$^{19}$F NMR (376 MHz, Chloroform-*d*) δ -88.98 (d, *J* = 40.0 Hz), -91.55 (d, *J* = 39.7 Hz).

HRMS (ESI) (m/z): $[M+H]^+$ Calcd for $C_{27}H_{31}F_2O^+$, 409.2337; Found 409.2339.

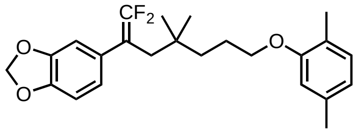


**5-(7-(2,5-dimethylphenoxy)-1,1-difluoro-4,4-dimethylhept-1-en-2-yl)benzo[*d*][1,3]dioxole (45)**

Prepared following the general procedure B. Purification by column chromatography (petroleum ether/ethyl acetate = 20:1) provided the title compound (71.6 mg, 89% yield), as a colorless oil.

$^{1}$H NMR (400 MHz, Chloroform-*d*) δ 6.92 (d, *J* = 7.4 Hz, 1H), 6.71 (d, *J* = 1.3 Hz, 1H), 6.69 (s, 2H), 6.57 (d, *J* = 7.2 Hz, 1H), 6.50 (s, 1H), 5.81 (s, 2H), 3.68 (t, *J* = 6.5 Hz, 2H), 2.22 (s, 5H), 2.08 (s, 3H), 1.66 – 1.52 (m, 2H), 1.26 – 1.16 (m, 2H), 0.73 (s, 6H).

$^{13}$C NMR (101 MHz, Chloroform-*d*) δ 157.1, 154.4 (dd, *J* = 289.1, 287.3 Hz), 147.6, 146.6, 136.5, 130.3, 129.7 – 128.7 (m), 123.6, 122.0 (t, *J* = 2.9 Hz), 120.6, 112.0, 109.0 (t, *J* = 2.9 Hz), 108.2, 101.1, 90.5 (dd, *J* = 21.9, 13.5 Hz), 68.5, 39.7, 38.4, 35.0 (t, *J* = 2.5 Hz), 27.2, 24.3, 21.4, 15.8.

$^{19}$F NMR (376 MHz, Chloroform-*d*) δ -90.00 (d, *J* = 41.8 Hz), -91.94 (d, *J* = 42.0 Hz).

HRMS (ESI) (m/z): $[M+H]^+$ Calcd for $C_{24}H_{29}F_2O_3^+$ 403.2079; Found 403.2078.

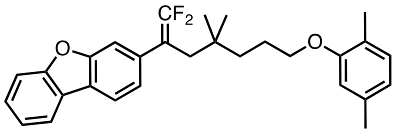


**3-(7-(2,5-dimethylphenoxy)-1,1-difluoro-4,4-dimethylhept-1-en-2-yl)dibenzo[*b*,*d*]furan (46)**

Prepared following the general procedure B. Purification by column chromatography (petroleum ether/ethyl acetate = 20:1) provided the title compound (84.4 mg, 94% yield), as a white solid.

$^{1}$H NMR (500 MHz, Chloroform-*d*) δ 7.88 – 7.75 (m, 2H), 7.50 – 7.38 (m, 2H), 7.34 (ddd, *J* = 8.4, 7.2, 1.3 Hz, 1H), 7.27 – 7.16 (m, 2H), 6.88 (d, *J* = 7.4 Hz, 1H), 6.53 (dd, *J* = 7.5, 1.5 Hz, 1H), 6.40 (d, *J* = 1.6 Hz, 1H), 3.63 (t, *J* = 6.5 Hz, 2H), 2.37 (t, *J* = 2.4 Hz, 2H), 2.15 (s, 3H), 2.05 (s, 3H), 1.69 – 1.52 (m, 2H), 1.32 – 1.17 (m, 2H), 0.72 (s, 6H).

$^{13}$C NMR (126 MHz, Chloroform-*d*) δ 157.0, 156.6, 156.3, 156.1 (t, *J* = 290.9 Hz), 136.4, 135.4 – 134.1 (m), 130.3, 127.3, 124.0, 123.6, 123.4 (t, *J* = 2.7 Hz), 123.3, 122.9, 120.6 (d, *J* = 1.9 Hz), 120.4, 112.0, 111.8 (q, *J* = 2.8 Hz), 111.7, 91.1 (dd, *J* = 22.0, 13.4 Hz), 68.4, 39.8, 38.5, 35.2 (d, *J* = 2.7 Hz), 27.2, 24.3, 21.4, 15.8.

$^{19}$F NMR (471 MHz, Chloroform-*d*) δ -88.78 (d, *J* = 38.6 Hz), -91.14 (d, *J* = 38.6 Hz).

HRMS (ESI) (m/z): $[M+H]^+$ Calcd for $C_{29}H_{31}F_2O_2^+$ 449.2287; Found 449.2290.

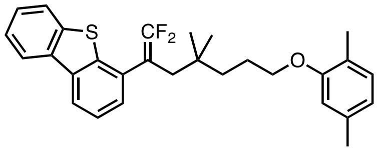


**4-(7-(2,5-dimethylphenoxy)-1,1-difluoro-4,4-dimethylhept-1-en-2-yl)dibenzo[*b*,*d*]thiophene (47)**

Prepared following the general procedure B. Purification by column chromatography (petroleum ether/ethyl acetate = 20:1) provided the title compound (46.4 mg, 50% yield), as a white solid.

$^{1}$H NMR (400 MHz, Chloroform-*d*) δ 8.14 – 8.09 (m, 1H), 8.06 – 8.04 (m, 1H), 7.87 – 7.82 (m, 1H), 7.47 – 7.42 (m, 3H), 7.38 (dd, *J* = 7.5, 1.2 Hz, 1H), 6.97 (d, *J* = 7.4 Hz,

1H), 6.64 (dd, $J$ = 7.5, 1.5 Hz, 1H), 6.48 (d, $J$ = 1.5 Hz, 1H), 3.62 (t, $J$ = 6.5 Hz, 2H), 2.55 (s, 2H), 2.28 (s, 3H), 2.10 (s, 3H), 1.60 (m, 2H), 1.43 – 1.28 (m, 2H), 0.84 (s, 6H).

$^{13}$C NMR (151 MHz, Chloroform-*d*) δ 157.1, 154.0 (t, $J$ = 290.9 Hz), 139.4, 139.3, 136.4, 136.1, 135.8, 130.3, 127.5 (d, $J$ = 4.3 Hz), 127.0, 124.7, 124.5, 123.6, 122.8, 121.8, 120.8, 120.6, 112.1, 89.9 (dd, $J$ = 23.8, 14.7 Hz), 68.4, 39.9, 38.2, 35.2, 27.1, 24.4, 21.5, 15.8.

$^{19}$F NMR (376 MHz, Chloroform-*d*) δ -84.52 (d, $J$ = 33.4 Hz), -88.21 (d, $J$ = 33.5 Hz).

HRMS (ESI) (m/z): [M+H]$^+$ Calcd for $C_{29}H_{31}F_2OS^+$ 465.2058; Found 465.2059.

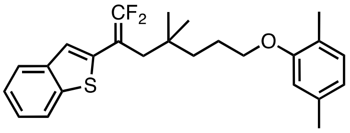


**2-(7-(2,5-dimethylphenoxy)-1,1-difluoro-4,4-dimethylhept-1-en-2-yl)benzo[*b*]thiophene (48)**

Prepared following the general procedure B. Purification by column chromatography (petroleum ether/ethyl acetate = 20:1) provided the title compound (62.9 mg, 76% yield), as a colorless oil.

$^1$H NMR (500 MHz, Chloroform-*d*) δ 7.79 – 7.65 (m, 2H), 7.38 – 7.22 (m, 3H), 6.99 (d, $J$ = 7.4 Hz, 1H), 6.65 (d, $J$ = 7.5 Hz, 1H), 6.56 (s, 1H), 3.83 (t, $J$ = 6.4 Hz, 2H), 2.43 (t, $J$ = 2.5 Hz, 2H), 2.29 (s, 3H), 2.15 (s, 3H), 1.84 – 1.70 (m, 2H), 1.48 – 1.35 (m, 2H), 0.90 (s, 6H).

$^{13}$C NMR (126 MHz, Chloroform-*d*) δ 157.1, 154.8 (dd, $J$ = 296.4, 290.1 Hz), 139.7, 139.2 (d, $J$ = 3.5 Hz), 138.2 (t, $J$ = 5.5 Hz), 136.5, 130.3, 124.4, 124.3, 123.6, 123.4, 122.5 (t, $J$ = 4.6 Hz), 122.0, 120.7, 112.1, 86.7 (dd, $J$ = 26.5, 12.0 Hz), 68.5, 40.0, 38.8, 35.3, 26.9, 24.4, 21.5, 15.8.

$^{19}$F NMR (471 MHz, Chloroform-*d*) δ -83.99 (d, $J$ = 28.8 Hz), -84.85 (d, $J$ = 27.8 Hz).

HRMS (ESI) (m/z): [M+H]$^+$ Calcd for $C_{25}H_{29}F_2OS^+$ 415.1902; Found 415.1909.

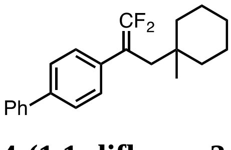


**4-(1,1-difluoro-3-(1-methylcyclohexyl)prop-1-en-2-yl)-1,1'-biphenyl (49)**

Prepared following the general procedure B. Purification by column chromatography (petroleum ether) provided the title compound (56.1 mg, 86% yield), as a colorless oil.

$^{1}$H NMR (400 MHz, Chloroform-*d*) δ 7.71 – 7.62 (m, 2H), 7.62 – 7.57 (m, 2H), 7.50 – 7.43 (m, 2H), 7.43 – 7.39 (m, 2H), 7.39 – 7.34 (m, 1H), 2.42 (t, *J* = 2.4 Hz, 2H), 1.57 – 1.31 (m, 5H), 1.22 (t, *J* = 5.9 Hz, 5H), 0.82 (s, 3H).

$^{13}$C NMR (101 MHz, Chloroform-*d*) δ 154.4 (dd, *J* = 290.1, 287.6 Hz), 140.6, 139.7, 134.9 (dd, *J* = 4.9, 2.8 Hz), 128.9 (t, *J* = 2.9 Hz), 128.8, 127.3, 127.0, 126.9, 90.3 (dd, *J* = 21.8, 13.0 Hz), 40.2, 38.1, 35.3 (t, *J* = 2.5 Hz), 26.3, 24.7, 22.0.

$^{19}$F NMR (376 MHz, Chloroform-*d*) δ -88.96 (d, *J* = 40.6 Hz), -91.71 (d, *J* = 40.6 Hz).

HRMS (ESI) (m/z): $[M+Na]^+$ Calcd for $C_{24}H_{27}F_2NNaO_4^+$, 454.1800; Found, 454.1805.

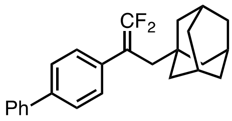


**(3*r*,5*r*,7*r*)-1-(2-([1,1'-biphenyl]-4-yl)-3,3-difluoroallyl)adamantane (50)[4]**

Prepared following the general procedure B. Purification by column chromatography (petroleum ether) provided the title compound (54.6 mg, 75% yield), as a colorless oil.

$^{1}$H NMR (400 MHz, Chloroform-*d*) δ 7.65 – 7.53 (m, 4H), 7.47 – 7.28 (m, 5H), 2.24 (t, *J* = 2.6 Hz, 2H), 1.91 – 1.84 (m, 3H), 1.68 – 1.50 (m, 7H), 1.41 (d, *J* = 2.9 Hz, 6H).

$^{13}$C NMR (101 MHz, Chloroform-*d*) δ 154.4 (dd, *J* = 290.8, 287.3 Hz), 140.6, 139.5, 134.9 (dd, *J* = 4.8, 3.1 Hz), 128.8, 128.7 (t, *J* = 3.0 Hz), 127.3, 126.9 (d, *J* = 9.4 Hz), 89.5 (dd, *J* = 22.0, 12.2 Hz), 42.7, 41.8, 36.9, 34.7, 28.6.

$^{19}$F NMR (376 MHz, Chloroform-*d*) δ -88.44 (d, *J* = 40.4 Hz), -91.62 (d, *J* = 40.4 Hz).

The spectroscopic data were consistent with those previously reported in the literature[4].

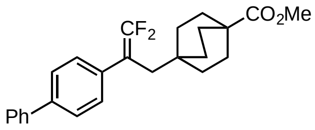

**methyl 4-(2-([1,1'-biphenyl]-4-yl)-3,3-difluoroallyl)bicyclo[2.2.2]octane-1-carboxylate (51)**

Prepared following the general procedure B. Purification by column chromatography (petroleum ether) provided the title compound (73.7 mg, 93% yield), as a colorless oil.

$^{1}$H NMR (400 MHz, Chloroform-*d*) δ 7.60 (dd, *J* = 8.1, 1.4 Hz, 2H), 7.58 – 7.55 (m, 2H), 7.43 (t, *J* = 7.6 Hz, 2H), 7.39 – 7.30 (m, 3H), 3.59 (s, 3H), 2.29 (t, *J* = 2.5 Hz, 2H), 1.80 – 1.54 (m, 6H), 1.42 – 1.15 (m, 6H).

$^{13}$C NMR (126 MHz, Chloroform-*d*) δ 178.4, 154.4 (dd, *J* = 291.0, 287.7 Hz), 140.5, 139.8, 134.3 (dd, *J* = 4.5, 3.1 Hz), 128.8, 128.7 (t, *J* = 3.0 Hz), 127.4, 127.0, 89.9 (dd, *J* = 21.7, 12.8 Hz), 51.6, 38.7, 32.9 (t, *J* = 2.6 Hz), 30.7, 28.5.

$^{19}$F NMR (376 MHz, Chloroform-*d*) δ -88.53 (d, *J* = 39.6 Hz), -91.43 (d, *J* = 39.7 Hz).

HRMS (ESI) (m/z): $[M+Na]^+$ Calcd for $C_{25}H_{26}F_2NaO_2^+$, 419.1793; Found 419.1795.

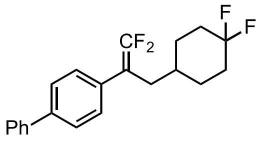

***tert*-butyl 4-((3-(dibenzo[*b*,*d*]thiophen-4-yl)-4,4-difluorobut-3-enoyl)oxy)piperidine-1-carboxylate (53)[5]**

Prepared following the general procedure B, using 1-Hexanaminium (50 mol%, 0.1 mmol) instead of NaI (50 mol%, 0.1 mmol). Purification by column chromatography (petroleum ether) provided the title compound (38.3 mg, 55% yield), as a colorless oil.

$^{1}$H NMR (400 MHz, Chloroform-*d*) δ 7.62 (dd, *J* = 8.0, 2.5 Hz,4H), 7.47 (t, *J* = 7.5 Hz, 2H), 7.38 (dd, *J* = 13.0, 7.3 Hz, 3H), 2.41 (dt, *J* = 6.9, 2.4 Hz, 2H), 2.12 – 2.00 (m, 2H), 1.78 (d, *J* = 13.2 Hz, 2H), 1.74 – 1.53 (m, 2H), 1.47 – 1.25 (m, 3H).

$^{13}C$ NMR (126 MHz, $CDCl_3$) δ 171.5 – 166.5 (m), 154.7, 154.5 (t, *J* = 292.0 Hz), 139.6, 139.0, 136.2, 135.6, 127.7, 127.1, 124.8, 124.6, 122.8, 121.8, 121.5, 86.4 – 85.1 (m), 79.7, 70.8, 33.8 (2C), 30.4, 28.4.

$^{19}F$ NMR (376 MHz, Chloroform-*d*) δ -90.04 (d, *J* = 41.5 Hz), -90.52 (d, *J* = 41.6 Hz), -92.05 (d, *J* = 235.0 Hz), -101.86 (d, *J* = 234.7 Hz).

The spectroscopic data were consistent with those previously reported in the literature[5].

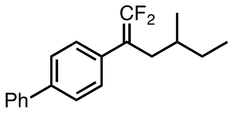


**4-(1,1-difluoro-4-methylhex-1-en-2-yl)-1,1'-biphenyl (53)[6]**

Prepared following the general procedure B, using 1-Hexanaminium (50 mol%, 0.1 mmol) instead of NaI (50 mol%, 0.1 mmol). Purification by column chromatography (petroleum ether) provided the title compound (25.2 mg, 44% yield), as a colorless oil.

$^{1}H$ NMR (400 MHz, Chloroform-*d*) δ 7.59 (m, 4H), 7.46 – 7.41 (m, 2H), 7.40 – 7.37 (m, 2H), 7.36 – 7.32 (m, 1H), 2.43 (m, 1H), 2.23 (m, 1H), 1.46 – 1.33 (m, 2H), 1.25 – 1.09 (m, 1H), 0.90 – 0.82 (m, 6H).

$^{13}C$ NMR (126 MHz, Chloroform-*d*) δ 154.1 (dd, *J* = 290.4, 286.4 Hz), 140.6, 139.9, 133.0 (t, *J* = 3.9 Hz), 128.8, 128.7 (t, *J* = 3.3 Hz), 127.4, 127.1, 127.0, 91.3 (dd, *J* = 21.9, 12.6 Hz), 34.5, 32.7 (t, *J* = 2.3 Hz), 29.1, 18.6, 11.3.

$^{19}F$ NMR (376 MHz, Chloroform-*d*) δ -91.02 (d, *J* = 46.7 Hz), -91.34 (d, *J* = 46.5 Hz).

The spectroscopic data were consistent with those previously reported in the literature[6].

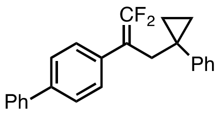


**4-(1,1-difluoro-3-(1-phenylcyclopropyl)prop-1-en-2-yl)-1,1'-biphenyl (54)**

Prepared following the general procedure B. Purification by column chromatography (petroleum ether) provided the title compound (58.1 mg, 84% yield), as a colorless oil.

$^{1}H$ NMR (400 MHz, Chloroform-*d*) δ 7.60 (dt, *J* = 7.0, 2.6, 1.5 Hz, 2H), 7.52 (dd, 2H), 7.44 (td, *J* = 8.4, 6.9 Hz, 2H), 7.39 – 7.30 (m, 1H), 7.27 – 7.21 (m, 6H), 7.18 – 7.12 (m, 1H), 2.76 (t, *J* = 2.3 Hz, 2H), 0.70 (t, 2H), 0.63 (t, 2H).

$^{13}$C NMR (101 MHz, Chloroform-*d*) δ 157.9 – 149.5 (m), 144.4, 140.6, 139.8, 133.3 (dd, *J* = 4.5, 2.9 Hz), 129.4, 129.0 (t, *J* = 3.0 Hz), 128.8, 128.0, 127.4, 127.0, 126.8, 126.2, 90.6 (dd, *J* = 21.5, 13.6 Hz), 38.0 (d, *J* = 1.9 Hz), 25.0 (dd, *J* = 3.3, 2.3 Hz), 12.1.

$^{19}$F NMR (376 MHz, Chloroform-*d*) δ -90.25 (d, *J* = 39.8 Hz), -91.64 (d, *J* = 40.2 Hz).

HRMS (ESI) (m/z): $[M+H]^+$ Calcd for $C_{24}H_{21}F_2^+$, 347.1606; Found 347.1609.

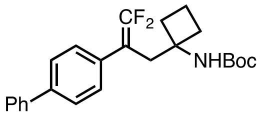


***tert*-butyl (1-(2-([1,1'-biphenyl]-4-yl)-3,3-difluoroallyl)cyclobutyl)carbamate (55)**

Prepared following the general procedure B. Purification by column chromatography (petroleum ether/ethyl acetate = 3:1) provided the title compound (58.3 mg, 73% yield), as a colorless oil.

$^{1}$H NMR (400 MHz, Chloroform-*d*) δ 7.62 – 7.53 (m, 4H), 7.43 (t, *J* = 7.6 Hz, 2H), 7.40 – 7.30 (m, 3H), 4.42 (s, 1H), 2.99 (s, 2H), 2.08 – 1.83 (m, 6H), 1.30 (s, 9H).

$^{13}$C NMR (101 MHz, Chloroform-*d*) δ 153.8, 154.3 (dd, *J* = 290.3, 288.0 Hz), 140.6, 139.9, 133.6, 128.8, 128.7 (t, *J* = 2.8 Hz), 127.4, 127.2, 127.0, 89.8 (dd, *J* = 21.7, 14.1 Hz), 78.8, 56.9 (t, *J* = 2.7 Hz), 34.7, 32.7, 28.3, 15.1.

$^{19}$F NMR (376 MHz, Chloroform-*d*) δ -90.12 (d, *J* = 39.9 Hz), -90.74 (d, *J* = 40.2 Hz).

HRMS (ESI) (m/z): $[M+Na]^+$ Calcd for $C_{22}H_{28}F_2N_2NaO_5^+$, 461.1858; Found 461.1854.

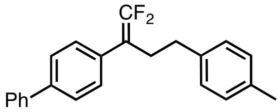


**4-(1,1-difluoro-4-(*p*-tolyl)but-1-en-2-yl)-1,1'-biphenyl (56)**

Prepared following the general procedure B. 2-(diphenylphosphanyl)pyridine (0.2 mmol) used, instead of triphenylphosphine (0.2 mmol). Purification by column chromatography (petroleum ether) provided the title compound (26.7 mg, 40% yield), as a colorless oil.

$^{1}$H NMR (500 MHz, Chloroform-*d*) δ 7.64 – 7.58 (m, 4H), 7.49 – 7.43 (m, 2H), 7.42 – 7.33 (m, 3H), 7.15 – 7.02 (m, 4H), 2.76 – 2.62 (m, 4H), 2.32 (s, 3H).

$^{13}$C NMR (101 MHz, Chloroform-*d*) δ 153.85 (dd, *J* = 291.3, 287.4 Hz), 140.59, 140.10, 137.95, 135.59, 132.65 – 132.32 (m), 129.08, 128.83, 128.64 (t, *J* = 3.3 Hz), 128.30, 127.42, 127.19, 127.04, 91.65 (dd, *J* = 21.8, 13.1 Hz), 33.69 (t, *J* = 2.6 Hz), 29.72, 21.03.

$^{19}$F NMR (471 MHz, Chloroform-*d*) δ -90.49 (d, *J* = 41.6 Hz), -90.97 (d, *J* = 41.7 Hz).

HRMS (ESI) (m/z): $[M+Na]^+$ Calcd for $C_{23}H_{20}F_2Na^+$, 357.1425; Found 357.1423.

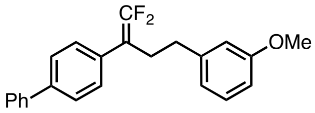


**4-(1,1-difluoro-4-(3-methoxyphenyl)but-1-en-2-yl)-1,1'-biphenyl (57)**

Prepared following the general procedure B. 2-(diphenylphosphanyl)pyridine (0.2 mmol) used, instead of triphenylphosphine (0.2 mmol). Purification by column chromatography (petroleum ether) provided the title compound (29.4 mg, 42% yield), as a colorless oil.

$^{1}$H NMR (500 MHz, Chloroform-*d*) δ 7.64 – 7.57 (m, 4H), 7.48 – 7.43 (m, 2H), 7.41 – 7.34 (m, 3H), 7.20 (t, *J* = 7.8 Hz, 1H), 6.77 – 6.73 (m, 2H), 6.70 (t, *J* = 2.0 Hz, 1H), 3.79 (s, 3H), 2.81 – 2.62 (m, 4H).

$^{13}$C NMR (101 MHz, Chloroform-*d*) δ 159.7, 153.9 (dd, *J* = 291.4, 287.5 Hz), 142.6, 140.6, 140.1, 132.4 (t, *J* = 3.8 Hz), 129.4, 128.8, 128.6 (t, *J* = 3.3 Hz), 127.4, 127.2, 127.0, 120.8, 114.2, 111.4, 91.6 (dd, *J* = 22.0, 13.2 Hz), 55.2, 34.1, 29.4.

$^{19}$F NMR (471 MHz, Chloroform-*d*) δ -90.38 (d, *J* = 41.6 Hz), -90.86 (d, *J* = 41.7 Hz).

HRMS (ESI) (m/z): $[M+Na]^+$ Calcd for $C_{23}H_{20}F_2NaO^+$, 373.1374; Found 373.1379.

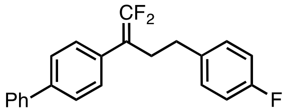


**4-(1,1-difluoro-4-(4-fluorophenyl)but-1-en-2-yl)-1,1'-biphenyl (58)**

Prepared following the general procedure B. 2-(diphenylphosphanyl)pyridine (0.2 mmol) used, instead of triphenylphosphine (0.2 mmol). Purification by column chromatography (petroleum ether) provided the title compound (22.3 mg, 33% yield), as a colorless oil.

$^{1}H$ NMR (500 MHz, Chloroform-*d*) δ 7.63 – 7.58 (m, 4H), 7.45 (dd, *J* = 8.4, 6.9 Hz, 2H), 7.41 – 7.33 (m, 3H), 7.12 – 7.06 (m, 2H), 7.00 – 6.93 (m, 2H), 2.75 – 2.65 (m, 4H).

$^{13}C$ NMR (101 MHz, Chloroform-*d*) δ 161.4 (d, *J* = 244.1 Hz), 154.0 (dd, *J* = 287.7, 283.9 Hz, ), 140.5, 140.2, 136.5 (d, *J* = 3.4 Hz), 132.3 (t, *J* = 4.0 Hz), 129.8 (d, *J* = 7.8 Hz), 128.8, 128.6 (t, *J* = 3.5 Hz), 127.5, 127.2, 127.0, 115.2, 115.0, 91.3 (dd, *J* = 21.7, 13.1 Hz), 33.2, 29.6.

$^{19}F$ NMR (471 MHz, Chloroform-*d*) δ -90.39 (d, *J* = 41.6 Hz), -90.80 (d, *J* = 41.5 Hz), -117.25.

HRMS (ESI) (m/z): $[M+Na]^+$ Calcd for $C_{22}H_{17}F_3Na^+$, 361.1175; Found 361.1178.

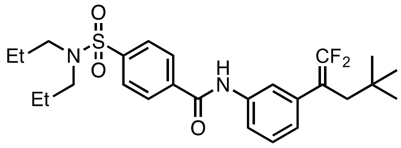


**N-(3-(1,1-difluoro-4,4-dimethylpent-1-en-2-yl)phenyl)-4-(N,N-dipropylsulfamoyl)benzamide (59)**

Prepared following the general procedure B. Purification by column chromatography (petroleum ether/ethyl acetate = 3:1) provided the title compound (62.0 mg, 63% yield), as a colorless oil.

$^{1}H$ NMR (500 MHz, Chloroform-*d*) δ 8.36 (s, 1H), 7.94 (d, *J* = 8.1 Hz, 2H), 7.82 – 7.72 (m, 2H), 7.72 – 7.60 (m, 2H), 7.35 (t, *J* = 7.9 Hz, 1H), 7.18 – 7.10 (m, 1H), 3.18 – 2.95 (m, 4H), 2.36 (t, *J* = 2.4 Hz, 2H), 1.54 (h, *J* = 7.5 Hz, 4H), 0.96 – 0.72 (m, 15H).

$^{13}C$ NMR (126 MHz, Chloroform-*d*) δ 164.8, 157.0 – 151.6 (m), 142.7, 138.8, 137.9, 136.7 (dd, *J* = 4.8, 2.4 Hz), 129.0, 128.0, 127.3, 125.0, 120.2, 118.9, 90.9 (dd, *J* = 22.2, 12.4 Hz), 50.0, 41.2, 32.8, 29.7, 21.9, 11.2.

$^{19}F$ NMR (471 MHz, Chloroform-*d*) δ -89.19 (d, *J* = 39.7 Hz), -91.51 (d, *J* = 39.7 Hz).

HRMS (ESI) (m/z): $[M+Na]^+$ Calcd for $C_{26}H_{34}F_2N_2NaO_3S^+$, 515.2150; Found 515.2159.

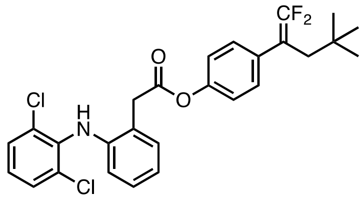


**4-(1,1-difluoro-4,4-dimethylpent-1-en-2-yl)phenyl 2-(2-((2,6-dichlorophenyl)amino)phenyl)acetate (60)**

Prepared following the general procedure B. Purification by column chromatography (petroleum ether/ethyl acetate = 5:1) provided the title compound (44.3 mg, 44% yield), as a colorless oil.

$^{1}$H NMR (400 MHz, Chloroform-*d*) δ 7.53 (s, 1H), 7.44 – 7.38 (m, 2H), 7.36 (s, 1H), 7.34 (s, 1H), 7.29 – 7.22 (m, 2H), 7.20 – 7.13 (m, 1H), 7.08 – 6.95 (m, 3H), 6.54 (dd, *J* = 8.1, 1.2 Hz, 1H), 3.85 (s, 2H), 2.46 – 2.17 (m, 2H), 0.78 (s, 9H).

$^{13}$C NMR (101 MHz, Chloroform-*d*) δ 169.4, 142.8, 137.7, 137.3, 136.6, 131.2, 130.3, 129.0 (2C), 128.6, 124.9, 123.7, 122.0, 120.2 (t, *J* = 2.7 Hz), 118.9, 117.6, 91.0 (dd, *J* = 22.0, 12.7 Hz), 42.1, 41.2, 32.9 (t, *J* = 2.4 Hz), 29.8.

$^{19}$F NMR (376 MHz, Chloroform-*d*) δ -89.25 (d, *J* = 39.6 Hz), -91.58 (d, *J* = 39.5 Hz).

HRMS (ESI) (m/z): $[M+Na]^+$ Calcd for $C_{27}H_{25}Cl_2F_2NNaO_2^+$, 526.1123; Found 526.1127.

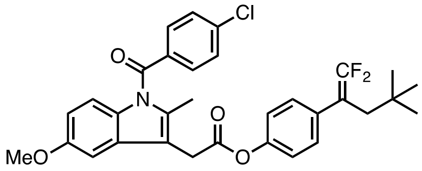


**4-(1,1-difluoro-4,4-dimethylpent-1-en-2-yl)phenyl 2-(1-(4-chlorobenzoyl)-5-methoxy-2-methyl-1*H*-indol-3-yl)acetate (61)**

Prepared following the general procedure B. Purification by column chromatography (petroleum ether/ethyl acetate = 5:1) provided the title compound (45.2 mg, 40% yield), as a colorless oil.

$^{1}$H NMR (500 MHz, Chloroform-*d*) δ 7.66 (d, *J* = 8.3 Hz, 2H), 7.53 – 7.46 (m, 2H), 7.40 (s, 1H), 7.32 (s, 1H), 7.24 (d, *J* = 6.4 Hz, 2H), 7.08 – 7.01 (m, 1H), 6.95 (d, *J* = 2.5 Hz, 1H), 6.88 (d, *J* = 9.0 Hz, 1H), 6.72 (dd, *J* = 9.0, 2.5 Hz, 1H), 3.81 (s, 5H), 2.45 (s, 3H), 2.29 (d, *J* = 2.5 Hz, 2H), 0.78 (s, 9H).

$^{13}$C NMR (126 MHz, Chloroform-*d*) δ 168.4, 168.2, 156.4, 139.7, 137.4, 136.7, 133.5, 131.2, 131.0, 130.2, 129.3, 128.8, 124.9 – 124.8 (m), 120.1 (d, *J* = 2.8 Hz), 118.8, 115.3, 112.5, 112.3, 100.7, 91.0 (dd, *J* = 22.4, 12.5 Hz), 55.8, 41.1, 33.4, 32.8, 29.7, 13.4.

$^{19}$F NMR (471 MHz, Chloroform-*d*) δ -89.21 (d, *J* = 39.7 Hz), -91.53 (d, *J* = 39.8 Hz).

HRMS (ESI) (m/z): $[M+Na]^+$ Calcd for $C_{32}H_{31}ClF_2NO_4^+$, 566.1904; Found 566.1910.

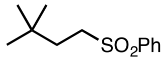


**((3,3-dimethylbutyl)sulfonyl)benzene (62)**

Prepared following the general procedure A. TBAI (40 mol%), $PPh_3$ (40 mol%). Purification by column chromatography (petroleum ether/ethyl acetate = 5:1) provided the title compound (18.8 mg, 83% yield), as a colorless oil.

$^1$H NMR (400 MHz, Chloroform-*d*) δ 7.94 – 7.87 (m, 2H), 7.70 – 7.63 (m, 1H), 7.61 – 7.54 (m, 2H), 3.13 – 2.97 (m, 2H), 1.75 – 1.44 (m, 2H), 0.86 (s, 9H).

$^{13}$C NMR (126 MHz, $CDCl_3$) δ 141.2, 135.7, 131.3, 130.1, 55.0, 37.6, 32.1, 31.0.

HRMS (ESI) (m/z): $[M+Na]^+$ Calcd for $C_{12}H_{18}NaO_2S^+$,249.0920; Found 249.0929.

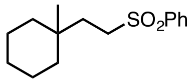


**((2-(1-methylcyclohexyl)ethyl)sulfonyl)benzene (63)**

Prepared following the general procedure A. TBAI (40 mol%), $PPh_3$ (40 mol%). Purification by column chromatography (petroleum ether/ethyl acetate = 5:1) provided the title compound (22.1 mg, 83% yield), as a colorless oil.

$^1$H NMR (400 MHz, Chloroform-*d*) δ 7.96 – 7.86 (m, 2H), 7.71 – 7.62 (m, 1H), 7.62 – 7.53 (m, 2H), 3.10 – 2.99 (m, 2H), 1.67 – 1.57 (m, 2H), 1.45 – 1.33 (m, 5H), 1.30 – 1.11 (m, 5H), 0.80 (s, 3H).

$^{13}$C NMR (126 MHz, $CDCl_3$) δ 141.2, 135.7, 135.6, 131.3, 131.3, 130.3, 130.0, 54.0, 39.4, 34.3, 28.1, 23.8.

HRMS (ESI) (m/z): $[M+Na]^+$ Calcd for $C_{15}H_{22}NaO_2S^+$,289.1233; Found 289.1239.

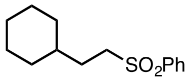

**((2-cyclohexylethyl)sulfonyl)benzene (64)**

Prepared following the general procedure A. TBAI (40 mol%), $PPh_3$ (40 mol%). Purification by column chromatography (petroleum ether/ethyl acetate = 5:1) provided the title compound (20.7 mg, 82% yield), as a colorless oil.

$^1$H NMR (500 MHz, Chloroform-*d*) δ 7.93 – 7.85 (m, 2H), 7.68 – 7.62 (m, 1H), 7.57 (dd, *J* = 8.5, 7.1 Hz, 2H), 3.25 – 2.97 (m, 2H), 1.75 – 1.52 (m, 7H), 1.35 – 1.01 (m, 4H), 0.93 – 0.77 (m, 2H).

$^{13}$C NMR (126 MHz, $CDCl_3$) δ 141.2, 135.6, 131.3, 130.1, 56.4, 38.7, 34.8, 31.6, 28.3, 28.0.

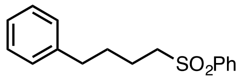

**((4-cyclohexylbutyl)sulfonyl)benzene (65)**

Prepared following the general procedure A. TBAI (40 mol%), $PPh_3$ (40 mol%). Purification by column chromatography (petroleum ether/ethyl acetate = 5:1) provided the title compound (26.0 mg, 50% yield), as a colorless oil.

$^1$H NMR (500 MHz, Chloroform-*d*) δ 7.89 (dd, *J* = 8.4, 1.3 Hz, 2H), 7.70 – 7.63 (m, 1H), 7.60 – 7.53 (m, 2H), 7.29 – 7.23 (m, 2H), 7.21 – 7.14 (m, 1H), 7.14 – 7.03 (m, 2H), 3.27 – 2.89 (m, 2H), 2.59 (t, *J* = 7.4 Hz, 2H), 1.83 – 1.58 (m, 4H).

$^{13}$C NMR (126 MHz, $CDCl_3$) δ 141.2, 139.1, 133.7, 129.3, 128.4, 128.3, 128.1, 126.3, 56.1, 35.3, 30.0, 22.3.

HRMS (ESI) (m/z): $[M+Na]^+$ Calcd for $C_{16}H_{18}NaO_2S^+$,297.0920; Found 297.0923.

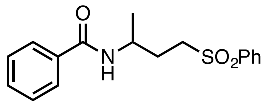

**N-(4-(phenylsulfonyl)butan-2-yl)benzamide (66)**

Prepared following the general procedure A. TBAI (40 mol%), $PPh_3$ (40 mol%). Purification by column chromatography (petroleum ether/ethyl acetate = 5:1) provided the title compound (26.0 mg, 85% yield), as a colorless oil.

$^1$H NMR (500 MHz, Chloroform-*d*) δ 7.90 – 7.84 (m, 2H), 7.79 – 7.74 (m, 2H), 7.65 (td, *J* = 7.2, 1.3 Hz, 1H), 7.55 (t, *J* = 7.8 Hz, 2H), 7.52 – 7.46 (m, 1H), 7.41 (dd, *J* = 8.3, 6.9 Hz, 2H), 6.38 (d, *J* = 8.7 Hz, 1H), 4.38 – 4.21 (m, 1H), 3.35 – 3.05 (m, 2H), 2.18 – 1.84 (m, 2H), 1.28 (d, *J* = 6.6 Hz, 3H).

$^{13}$C NMR (126 MHz, $CDCl_3$) δ 167.2, 139.0, 134.1, 133.9, 131.7, 129.4, 128.6, 128.0, 127.0, 53.6, 44.5, 29.6, 21.3.

HRMS (ESI) (m/z): $[M+Na]^+$ Calcd for $C_{17}H_{19}NNaO_3S^+$,340.0978; Found 340.0991.

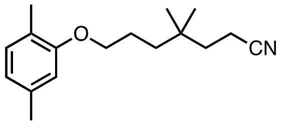


**7-(2,5-dimethylphenoxy)-4,4-dimethylheptanenitrile (67)**

Prepared following the general procedure A. TBAI (40 mol%), $PPh_3$ (40 mol%). Purification by column chromatography (petroleum ether/ethyl acetate = 5:1) provided the title compound (15.5 mg, 60% yield), as a colorless oil.

$^1$H NMR (500 MHz, Chloroform-*d*) δ 7.01 (d, *J* = 7.5 Hz, 1H), 6.79 – 6.49 (m, 2H), 3.92 (t, *J* = 6.2 Hz, 2H), 2.31 (s, 2H), 2.17 (s, 3H), 1.79 – 1.69 (m, 2H), 1.71 – 1.59 (m, 2H), 1.46 – 1.30 (m, 2H), 0.94 (s, 6H).

$^{13}$C NMR (126 MHz, $CDCl_3$) δ 156.9, 136.5, 130.4, 123.5, 120.8, 120.5, 111.9, 68.0, 37.5, 37.1, 32.6, 26.4, 24.2, 21.4, 15.8, 12.3.

HRMS (ESI) (m/z): $[M+H]^+$ Calcd for $C_{17}H_{26}NO^+$,260.2009; Found 260.2019.

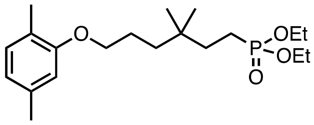


**diethyl (6-(2,5-dimethylphenoxy)-3,3-dimethylhexyl)phosphonate (68)**

Prepared following the general procedure A. TBAI (40 mol%), $PPh_3$ (40 mol%). Purification by column chromatography (petroleum ether/ethyl acetate = 5:1) provided the title compound (20.0 mg, 54% yield), as a colorless oil.

$^1$H NMR (500 MHz, Chloroform-*d*) δ 6.99 (d, *J* = 7.4 Hz, 1H), 6.67 – 6.57 (m, 2H), 4.16 – 4.00 (m, 4H), 3.89 (t, *J* = 6.3 Hz, 2H), 2.29 (s, 3H), 2.16 (s, 3H), 1.76 – 1.60 (m, 4H), 1.59 – 1.44 (m, 2H), 1.38 – 1.33 (m, 2H), 1.31 (t, *J* = 7.0 Hz, 6H), 0.89 (s, 6H).

$^{13}$C NMR (126 MHz, $CDCl_3$) δ 157.0, 136.5, 130.3, 123.5, 120.6, 111.9, 68.3, 61.6, 61.5, 37.5, 33.6, 33.6, 32.6, 32.5, 26.6, 24.2, 21.4, 16.5, 16.5, 15.8.

$^{31}$P NMR (202 MHz, Chloroform-*d*) δ 33.78.

HRMS (ESI) (m/z): $[M+Na]^+$ Calcd for $C_{20}H_{35}NaO_4P^+$,393.2165; Found 393.2172.

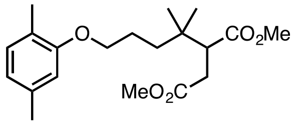


**dimethyl 2-(5-(2,5-dimethylphenoxy)-2-methylpentan-2-yl)succinate (69)**

Prepared following the general procedure A. TBAI (40 mol%), $PPh_3$ (40 mol%). Purification by column chromatography (petroleum ether/ethyl acetate = 5:1) provided the title compound (21.4 mg, 61% yield), as a colorless oil.

$^1$H NMR (500 MHz, Chloroform-*d*) δ 6.79 (d, *J* = 7.4 Hz, 1H), 6.50 – 6.33 (m, 2H), 3.75 – 3.65 (m, 2H), 3.46 (d, *J* = 17.1 Hz, 6H), 2.68 – 2.53 (m, 2H), 2.09 (s, 3H), 1.96 (s, 3H), 1.74 – 1.57 (m, 1H), 1.57 – 1.47 (m, 1H), 1.24 (dd, *J* = 9.5, 7.4 Hz, 2H), 0.76 (d, *J* = 3.6 Hz, 6H).

$^{13}$C NMR (126 MHz, $CDCl_3$) δ 174.7, 173.2, 156.9, 136.5, 130.3, 123.5, 120.7, 111.9, 68.1, 51.8, 51.5, 49.4, 37.1, 35.0, 32.2, 25.1, 24.9, 24.0, 21.4, 15.8.

HRMS (ESI) (m/z): $[M+Na]^+$ Calcd for $C_{20}H_{30}NaO_5^+$,373.1985; Found 373.1983.

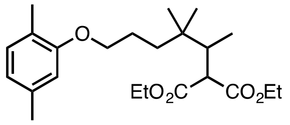


**diethyl 2-(6-(2,5-dimethylphenoxy)-3,3-dimethylhexan-2-yl)malonate (70)**

Prepared following the general procedure A. TBAI (40 mol%), $PPh_3$ (40 mol%). Purification by column chromatography (petroleum ether/ethyl acetate = 5:1) provided the title compound (35.3 mg, 45% yield), as a colorless oil.

$^1$H NMR (500 MHz, Chloroform-*d*) δ 6.99 (d, *J* = 7.4 Hz, 1H), 6.75 – 6.54 (m, 2H), 4.24 – 4.08 (m, 4H), 3.90 (t, *J* = 6.3 Hz, 2H), 3.55 (d, *J* = 4.9 Hz, 1H), 2.44 – 2.33 (m, 1H), 2.29 (s, 3H), 2.16 (s, 3H), 1.79 – 1.69 (m, 2H), 1.53 – 1.44 (m, 1H), 1.44 – 1.33 (m, 1H), 1.25 (q, *J* = 7.2 Hz, 6H), 1.02 (d, *J* = 7.2 Hz, 3H), 0.88 (d, *J* = 2.4 Hz, 6H).

$^{13}C$ NMR (101 MHz, Chloroform-*d*) δ 170.1, 156.1, 135.4, 129.2, 122.6, 119.5, 111.0, 67.1, 59.3, 34.3, 26.8, 26.3, 21.6, 20.4, 20.0, 14.7, 13.2.

HRMS (ESI) (m/z): $[M+Na]^+$ Calcd for $C_{23}H_{36}NaO_5^+$,415.2455; Found 415.2454.

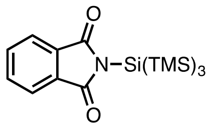


**2-(1,1,1,3,3,3-hexamethyl-2-(trimethylsilyl)trisilan-2-yl)isoindoline-1,3-dione (75)**

$^1H$ NMR (400 MHz, Chloroform-*d*) δ 7.78 – 7.69 (m, 2H), 7.65 – 7.57 (m, 2H), 0.20 (s, 6H).

$^{13}C$ NMR (101 MHz, $CDCl_3$) δ 172.5, 133.2, 132.3, 121.5, 0.0.

HRMS (ESI) (m/z): $[M+H]^+$ Calcd for $C_{11}H_{14}NO_2Si^+$,220.0788; Found 220.0793.

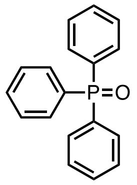


**triphenylphosphine oxide (76)[7]**

$^1H$ NMR (400 MHz, Chloroform-*d*) δ 7.77 – 7.60 (m, 6H), 7.59 – 7.34 (m, 9H).

$^{13}C$ NMR (151 MHz, Chloroform-*d*) δ 132.2, 132.1 (3C), 128.7, 128.6.

$^{31}P$ NMR (162 MHz, Chloroform-*d*) δ 29.52.

The spectroscopic data were consistent with those previously reported in the literature[7].

## 5. General Procedures for Scaling Up

### A. Synthesis of 4-(1,1,1-trifluoro-4,4-dimethylpentan-2-yl)-1,1'-biphenyl

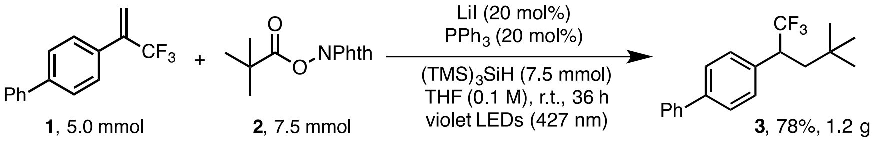


4-(3,3,3-trifluoroprop-1-en-2-yl)-1,1'-biphenyl (1.0 equiv., 5.0 mmol), 1,3-dioxoisoindolin-2-yl pivalate (1.5 equiv., 7.5 mmol), LiI (20 mol%), $PPh_3$ (20 mol%) were placed in a 100 mL transparent Schlenk tube equipped with a stirring bar. The test tube was evacuated and backfilled with argon (three times). To these solids, $(TMS)_3SiH$ (1.5 equiv., 7.5 mmol) and THF (0.1 M) were added via a gastight syringe under argon atmosphere. The reaction mixture was stirred under irradiation with 427 nm violet LEDs, maintained at approximately room temperature in the air-conditioned room of 25 $^oC$. After 36 h, the mixture was quenched with 30 mL water, the extracted with ethyl acetate (3 x 50 mL).The organic layers were combined and concentrated on rotary evaporator. The residue was purified via flash column chromatography on silica gel to give the product (white solid, 1.2 g, yield of 78%, petroleum ether).

### B. Synthesis of 4-(1,1-difluoro-4,4-dimethylpent-1-en-2-yl)-1,1'-biphenyl

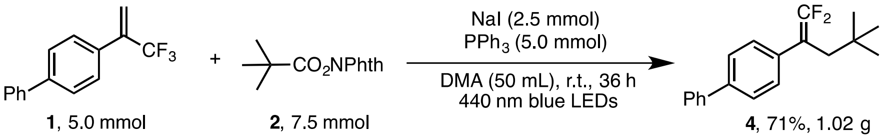


4-(3,3,3-trifluoroprop-1-en-2-yl)-1,1'-biphenyl (1.0 equiv., 5.0 mmol), 1,3-dioxoisoindolin-2-yl pivalate (1.5 equiv., 7.5 mmol), NaI (50 mol%, 2.5 mmol), $PPh_3$ (1.0 equiv., 5.0 mmol). were placed in a 100 mL transparent Schlenk tube equipped with a stirring bar. The test tube was evacuated and backfilled with argon (three times). To these solids, then DMA (0.1 M) was added via a gastight syringe under argon atmosphere. The reaction mixture was stirred under irradiation with 440 nm purple LEDs, maintained at approximately room temperature in the air-conditioned room of 25 $^oC$. After 36 h, ethyl acetate (30 mL) was added to the reation mixture. The resulting solution was washed with brine (3 x 50 mL) and dried over anhydrous $Na_2SO_4$. The organic layers were combined and concentrated on rotary evaporator. The residue was purified via flash column chromatography on silica gel to give the product (white solid, 1.02 g, yield of 71%, petroleum ether).

## 6. General Procedure of Continuous-Flow Reactions

### 6.1 Synthesis of 4-(1,1,1-trifluoro-4,4-dimethylpentan-2-yl)-1,1'-biphenyl

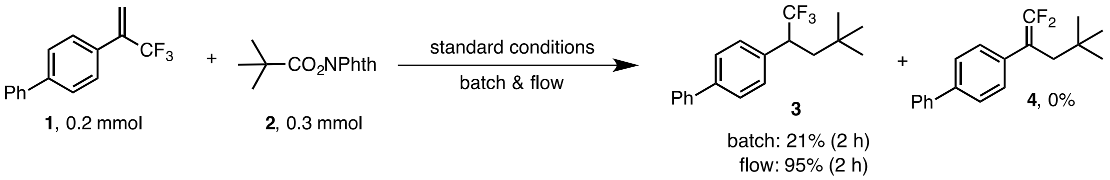


A 25 mL round bottom flask equipped with a magnetic stir bar was charged with 4-(3,3,3-trifluoroprop-1-en-2-yl)-1,1'-biphenyl (1.0 equiv., 0.8 mmol), 1,3-dioxoisoindolin-2-yl pivalate (1.5 equiv., 1.2 mmol), LiI (20 mol%), $PPh_3$ (20 mol%) and $(TMS)_3SiH$ (1.5 equiv., 1.2 mmol). The reagents were dissolved in anhydrous THF and the total volume of the solution was adjusted to 8 mL. The resulting mixture bubbled with an argon balloon for 20 min. After that, the reaction solution was introduced to the flow apparatus (Supplementary Figure S2). The flow apparatus was purged with degassed argon to remove the air first. The syringe pump was then connected to the reaction mixture and the 3.6 mL PFA microreactor coil (internal diameter of 1.0 mm) with a 5 psi back-pressure regulator (BPR). The reaction was placed under 427 nm violet LEDs. The flow apparatus itself was set up with residence time (TR) = 2 h, flow rate = 1.8 mL/h. When the syringe was fully empty, a crude sample (2 mL) was taken from the collected solution and analyzed by $^{19}F$-NMR spectroscopy using benzotrifluoride as an internal standard. The yield of product **3** was determined to be 95%.

### 6.2 Synthesis of 4-(1,1-difluoro-4,4-dimethylpent-1-en-2-yl)-1,1'-biphenyl

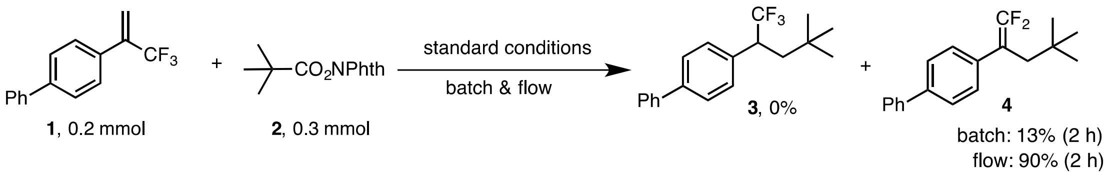


A 25 mL round bottom flask equipped with a magnetic stir bar was charged with 4-(3,3,3-trifluoroprop-1-en-2-yl)-1,1'-biphenyl (1.0 equiv., 0.8 mmol), 1,3-dioxoisoindolin-2-yl pivalate (1.5 equiv., 1.2 mmol), NaI (0.1 mmol) and $PPh_3$ (0.2 mmol). The reagents were dissolved in anhydrous DMA and the total volume of the solution was adjusted to 8 mL. The resulting mixture bubbled with an argon balloon for 20 min. After that, the reaction solution was introduced to the flow apparatus (Supplementary Figure S2). The flow apparatus was purged with degassed argon to remove the air first. The syringe pump was then connected to the reaction mixture and

the 3.6 mL PFA microreactor coil (internal diameter of 1.0 mm) with a 5 psi back-pressure regulator (BPR). The reaction was placed under 440 nm blue LEDs. The flow apparatus itself was set up with residence time (TR) = 2 h, flow rate = 1.8 mL/h. When the syringe was fully empty, a crude sample (2 mL) was taken from the collected solution and analyzed by $^{19}$F-NMR spectroscopy using benzotrifluoride as an internal standard. The yield of product **4** was determined to be 90%.

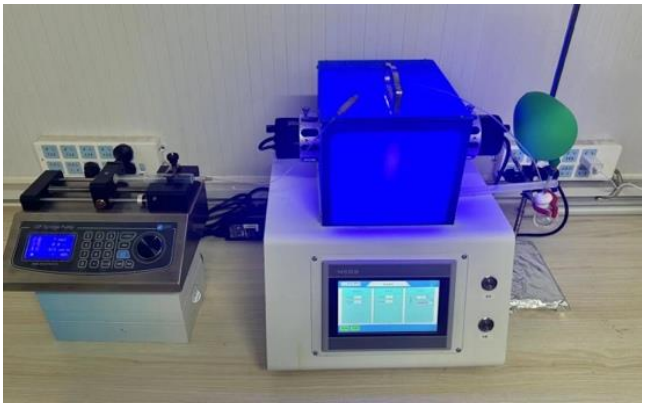

**Figure S2. Flow set-up with light irradiation**

## 7. Preliminary Mechanistic Studies

### 7.1. Radical Trap Experiment

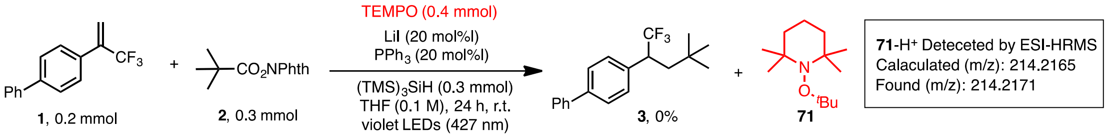


To a 10 mL transparent Schlenk tube equipped with a magnetic stirrer was added 4-(3,3,3 trifluoroprop-1-en-2-yl)-1,1'-biphenyl (1.0 equiv., 0.2 mmol), 1,3-dioxoisoindolin-2-yl pivalate (1.5 equiv., 0.3 mmol), LiI (20 mol%), $PPh_3$ (20 mol%), TEMPO (2.0 equiv., 0.4 mmol). The test tube was evacuated and backfilled with argon for three times, then $(TMS)_3SiH$ (1.5 equiv., 0.3 mmol) and THF (0.1 M) were added. The reaction was irradiated using 34 W Kessil PR160L 427 nm violet LEDs for 24 hours. The reaction mixture was sent for HRMS analysis, the compounds **71** were detected by HRMS.

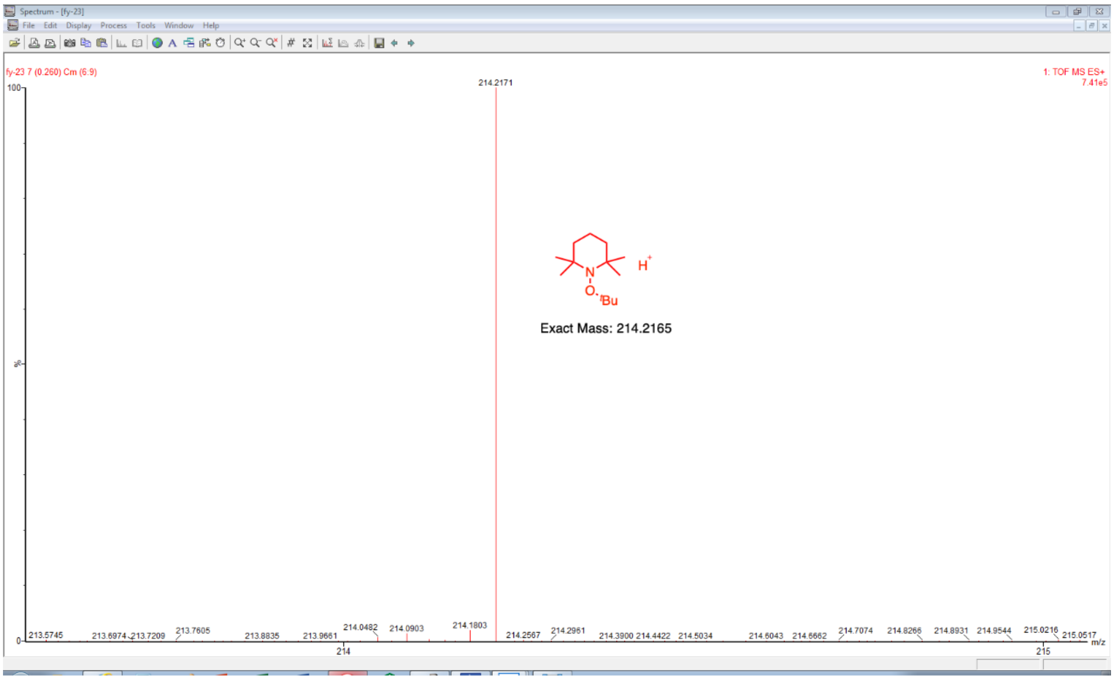


**Figure S3.** HRMS Spectra after TEMPO Trapping Experiment

## 7.2. Radical Clock Experiment

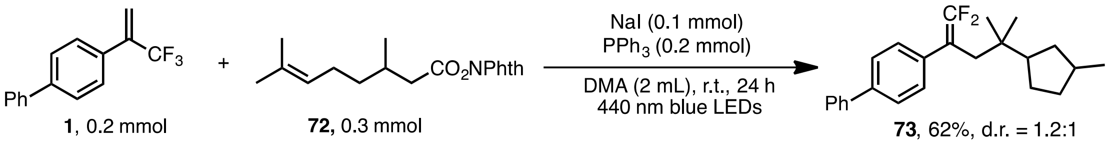


In the air, a 100 mL transparent Schlenk tube equipped with a magnetic stirrer was charged with 4-(3,3,3-trifluoroprop-1-en-2-yl)-1,1'-biphenyl (1.0 equiv., 0.2 mmol), 1,3-dioxoisoindolin-2-yl 3,7-dimethyloct-6-enoate (1.5 equiv., 0.3 mmol), NaI (50 mol%, 0.1 mmol), $PPh_3$ (1.0 equiv., 0.2 mmol). The test tube was evacuated and backfilled with argon for three times, then DMA (2 mL) were added. The reaction mixture was stirred under irradiation with 440 nm blue LEDs, maintained at approximately room temperature in the air-conditioned room of 25 $^{o}$C. After 24 h, ethyl acetate (5 mL) was added to the reaction mixture. The resulting solution was washed with brine (3 x 10 mL) and dried over anhydrous $Na_2SO_4$. The organic layers were combined and concentrated on rotary evaporator. The residue was purified via flash column chromatography on silica gel to give the product.

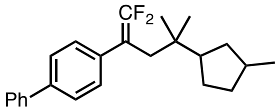


**4-(1,1-difluoro-4-methyl-4-(3-methylcyclopentyl)pent-1-en-2-yl)-1,1'-biphenyl (73)**

$^{1}$H NMR (400 MHz, Chloroform-*d*) δ 7.67 – 7.52 (m, 4H), 7.47 – 7.40 (m, 2H), 7.40 – 7.28 (m, 3H), 2.37 (q, *J* = 2.2 Hz, 2H), 1.91 – 1.63 (m, 3.74H), 1.60 – 1.45 (m, 2H), 1.30 – 1.17 (m, 1H), 1.11 – 1.00 (m, 1.26H), 0.95 (d, *J* = 6.5 Hz, 1.3H), 0.88 (d, *J* = 6.6 Hz, 1.7H), 0.70 (s, 6H).

$^{13}$C NMR (101 MHz, Chloroform-*d*) δ 158.2 – 150.6 (m), 140.6, 140.6, 139.7, 139.6, 135.0 – 134.7 (m), 128.9, 128.8, 128.8, 127.3, 127.0, 126.9, 90.6 (dd, *J* = 21.7, 13.0 Hz), 50.4, 48.6, 38.5, 38.4, 37.3, 37.1, 36.8, 35.3, 34.9, 34.6, 34.1, 34.0, 27.7, 26.0, 24.6, 24.6, 24.3, 24.2, 21.1, 20.6.

$^{19}$F NMR (376 MHz, Chloroform-*d*) δ -89.31 (d, *J* = 40.5 Hz), -90.28 – -95.79 (m).

HRMS (ESI) (m/z): $[M+Na]^+$ Calcd for $C_{24}H_{28}F_2Na^+$, 377.2051; Found 377.2056.

## 7.2. D-Labelling Experiments

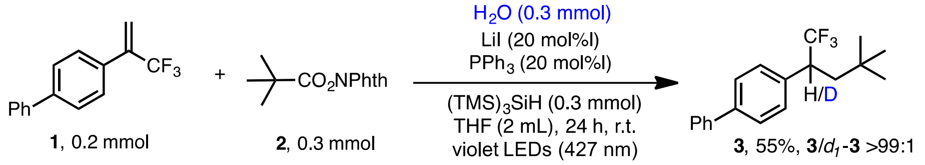


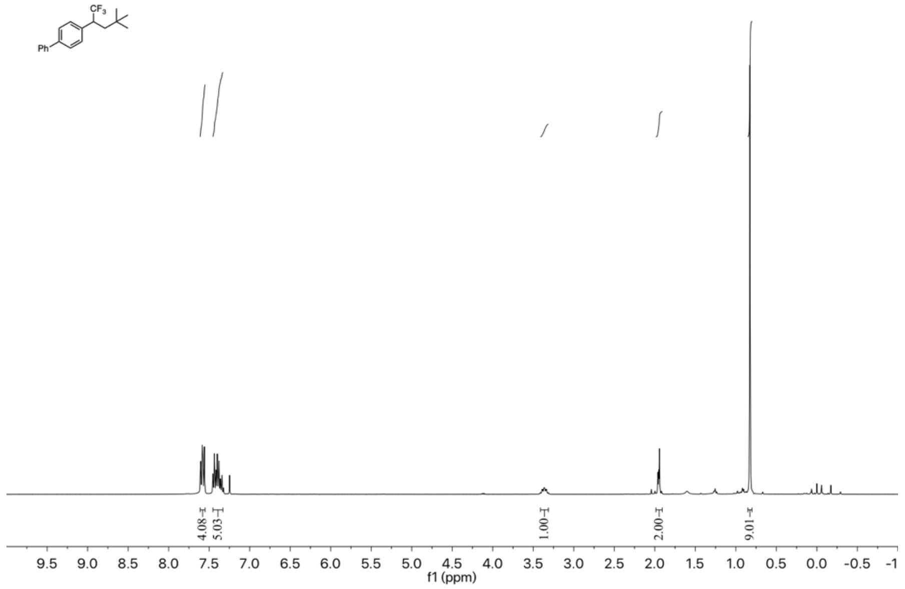


**Figure S4.** $^{1}$H (400 MHz, $CDCl_3$) spectrum of **3** and *$d_1$*-**3**

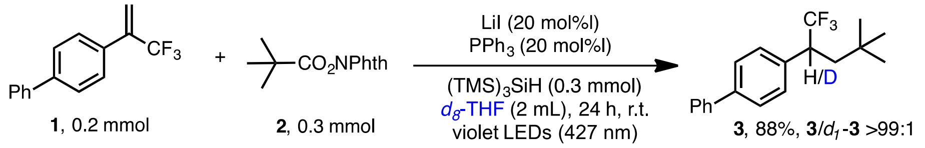


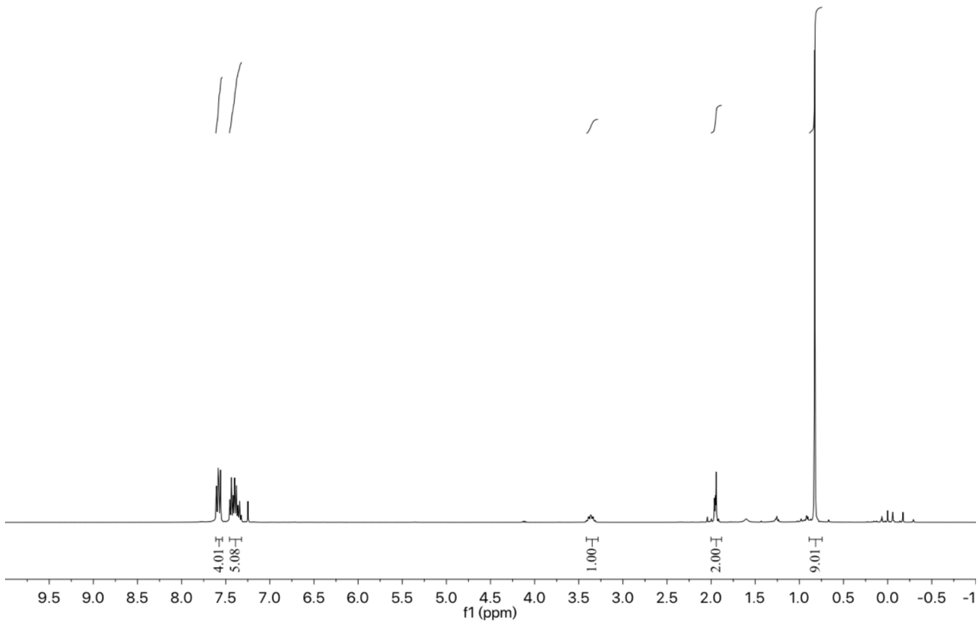


**Figure S5.** $^{1}$H (400 MHz, $CDCl_3$) spectrum of **3** and *$d_1$*-**3**

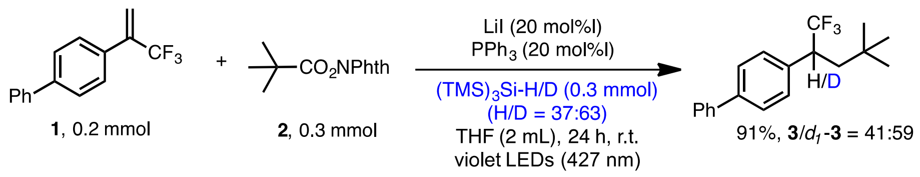


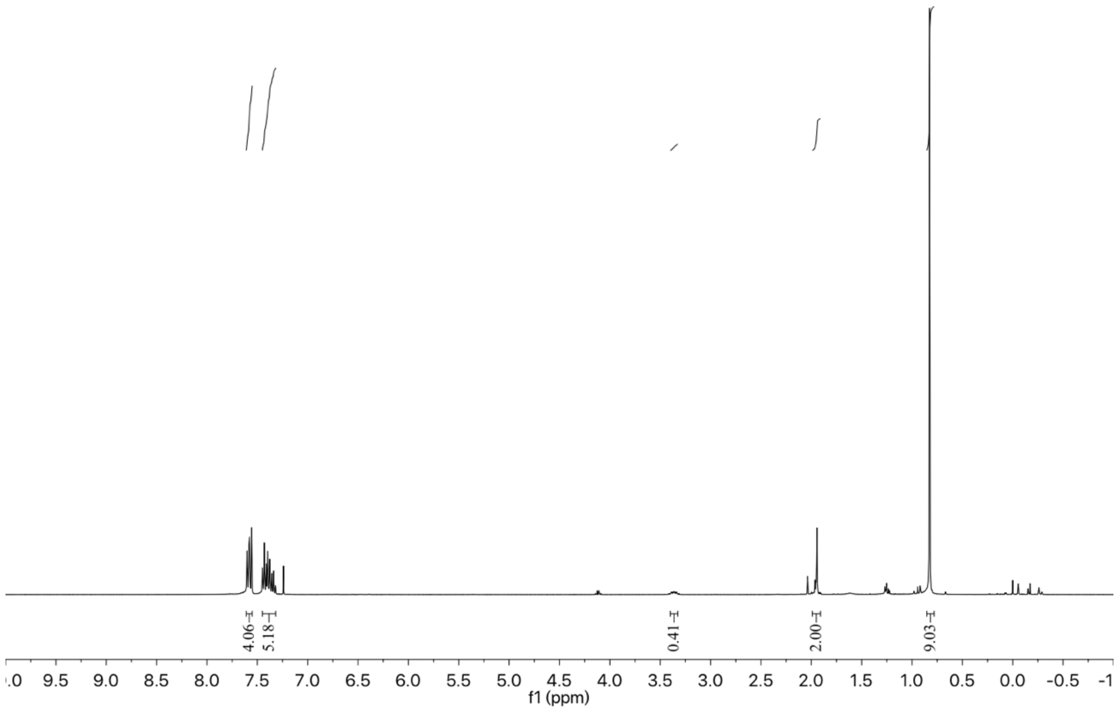


**Figure S6**. $^{1}$H (400 MHz, $CDCl_3$) spectrum of **3** and *$d_1$*-**3**

## 7.4 Light On-Off Experiments

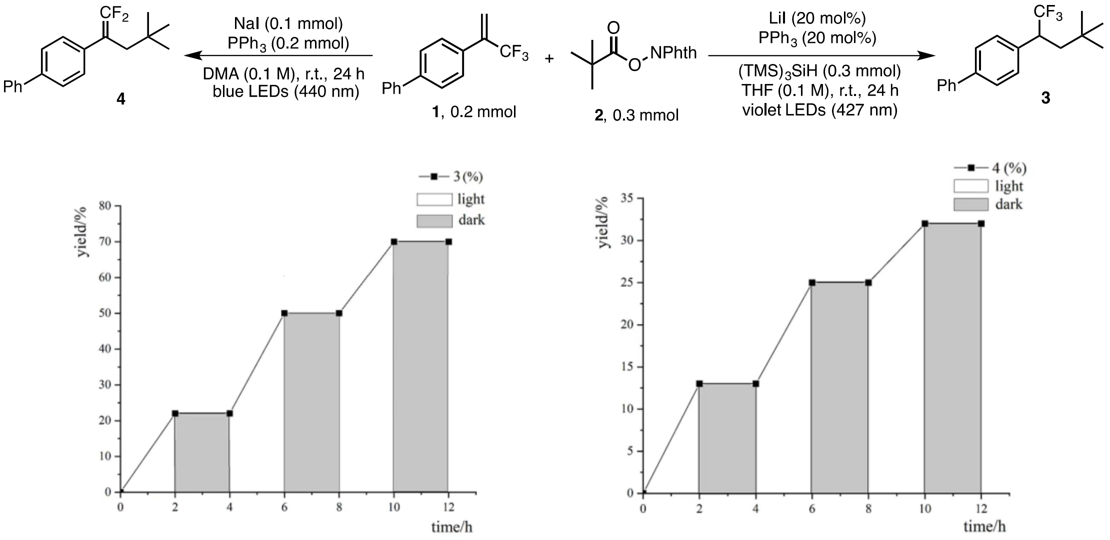


**Figure S7**. Light on-off experiments

To examine the impact of light, we conducted experiments under alternating periods of irradiation and darkness. These resulted in a total interruption of the reaction progress in the absence of light and recuperation of reactivity on further illumination, which allows precise temporal control over the entire reaction period. These results demonstrated that light is a necessary component of the reaction.

## 8. References


1. J. Cornella, J. T. Edwards, T. Qin, S. Kawamura, J. Wang, C.-M. Pan, R. Gianatassio, M. Schmidt, M. D. Eastgate, P. S. Baran, *J. Am. Chem. Soc.* **2016**, *138*, 2174.
2. F. Chen, X. Xu, Y. He, G. Huang, S. Zhu, *Angew. Chem., Int. Ed.* **2020**, *59*, 5398.
3. Y.-Q. Guo, Y. Wu, R. Wang, H. Song, Y. Liu, Q. Wang, *Org. Lett.* **2021**, *23*, 2353.
4. G. Zhang, L. Wang, L. Cui, P. Gao, F. Chen, *Org. Biomol. Chem.* **2023**, *21*, 294.
5. J.-X. Wang, W. Ge, M.-C. Fu, Y. Fu, *Org. Lett.* **2022**, *24,* 1471.
6. W. Chen, S. Ni, Y. Wang, Y. Pan, *Org. Lett.* **2022**, *24*, 3647.
7. W.-C. Fu, T. F. Jamison, *Angew. Chem., Int. Ed.* **2020**, *59*, 13885.

## 9. NMR Spectra

**$^{1}$H NMR (400 MHz, $CDCl_3$)** spectrum of **4-(1,1,1-trifluoro-4,4-dimethylpentan-2-yl)-1,1'-biphenyl (3)**

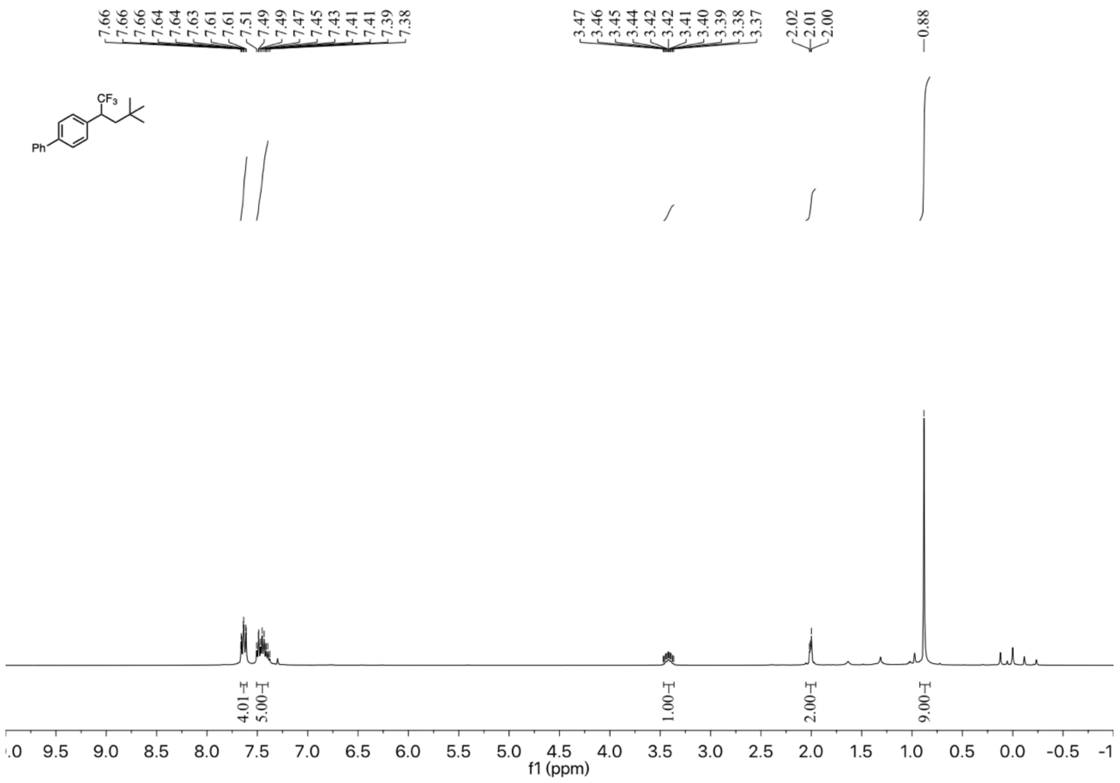


**$^{13}$C NMR (126 MHz, $CDCl_3$)** spectrum of **4-(1,1,1-trifluoro-4,4-dimethylpentan-2-yl)-1,1'-biphenyl (3)**

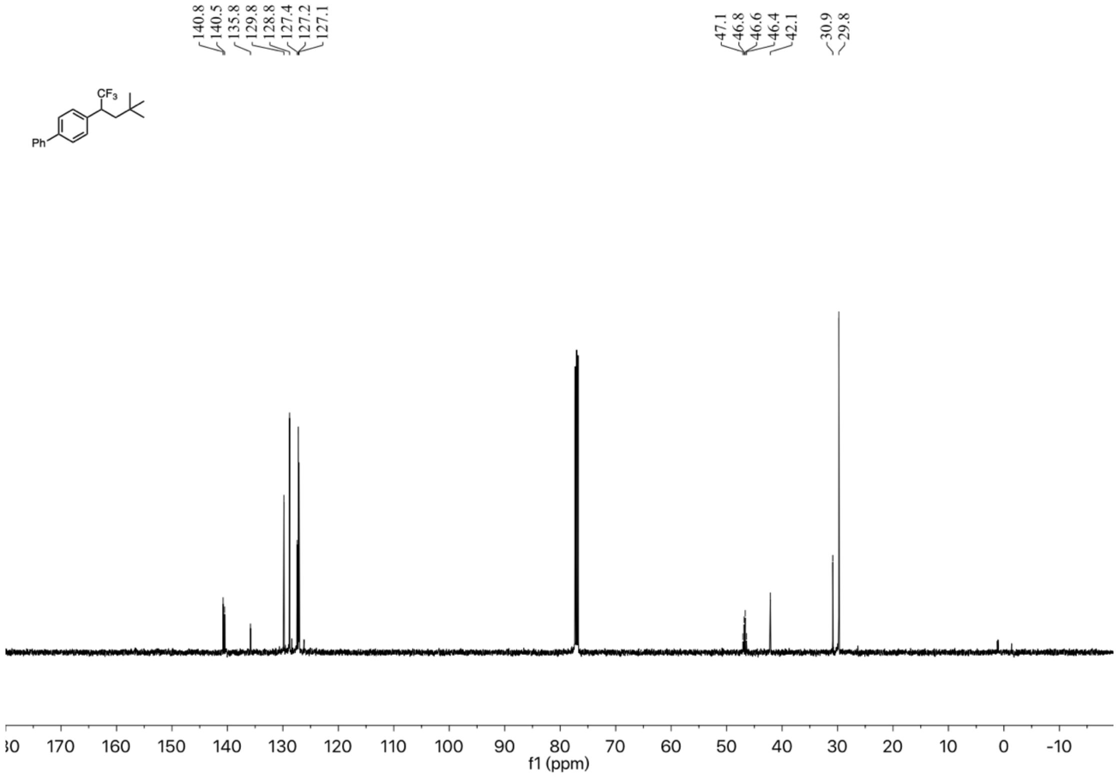

**$^{19}$F NMR (376 MHz, $CDCl_3$)** spectrum of **4-(1,1,1-trifluoro-4,4-dimethylpentan-2-yl)-1,1'-biphenyl (3)**

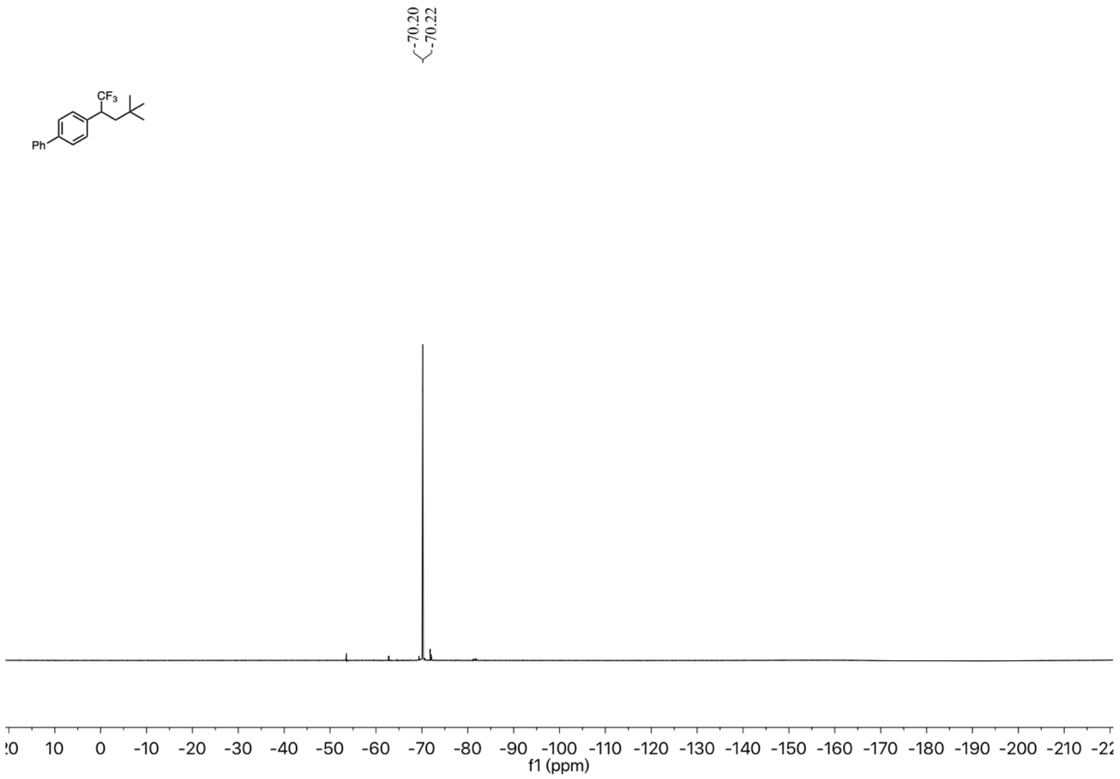


**$^{1}$H NMR (400 MHz, $CDCl_3$)** spectrum of **4-(1,1-difluoro-4,4-dimethylpent-1-en-2-yl)-1,1'-biphenyl (4)**

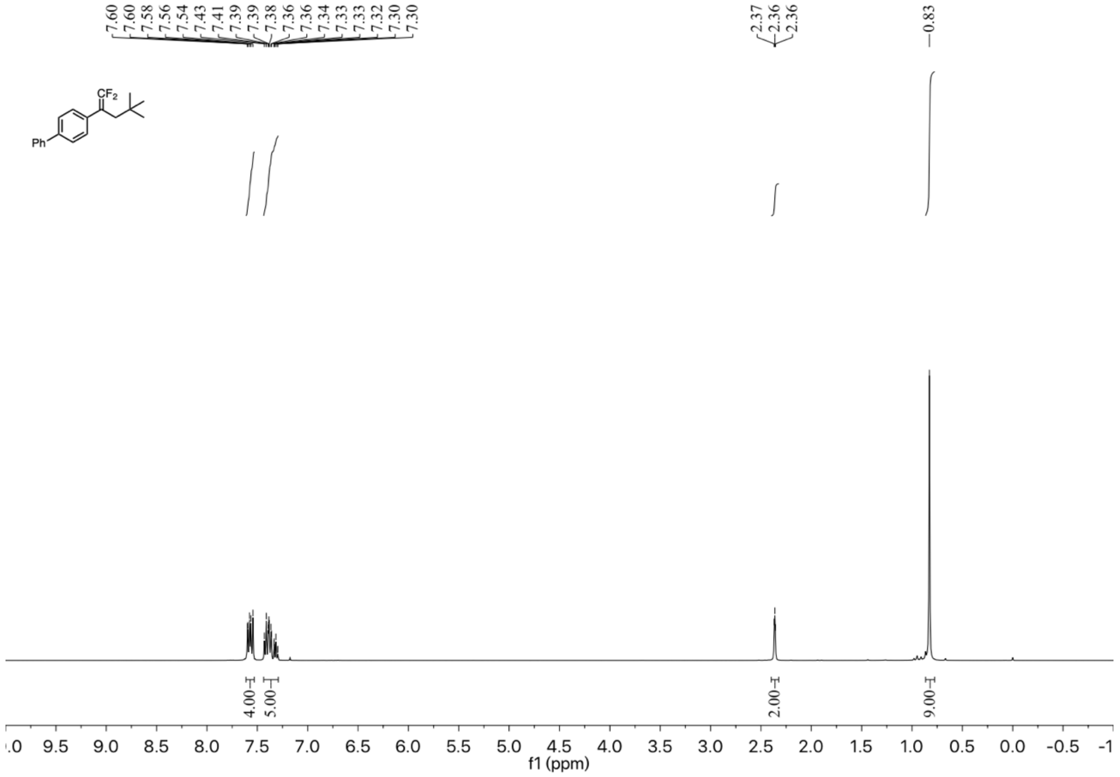

**$^{13}$C NMR (126 MHz, $CDCl_3$)** spectrum of **4-(1,1-difluoro-4,4-dimethylpent-1-en-2-yl)-1,1'-biphenyl (4)**

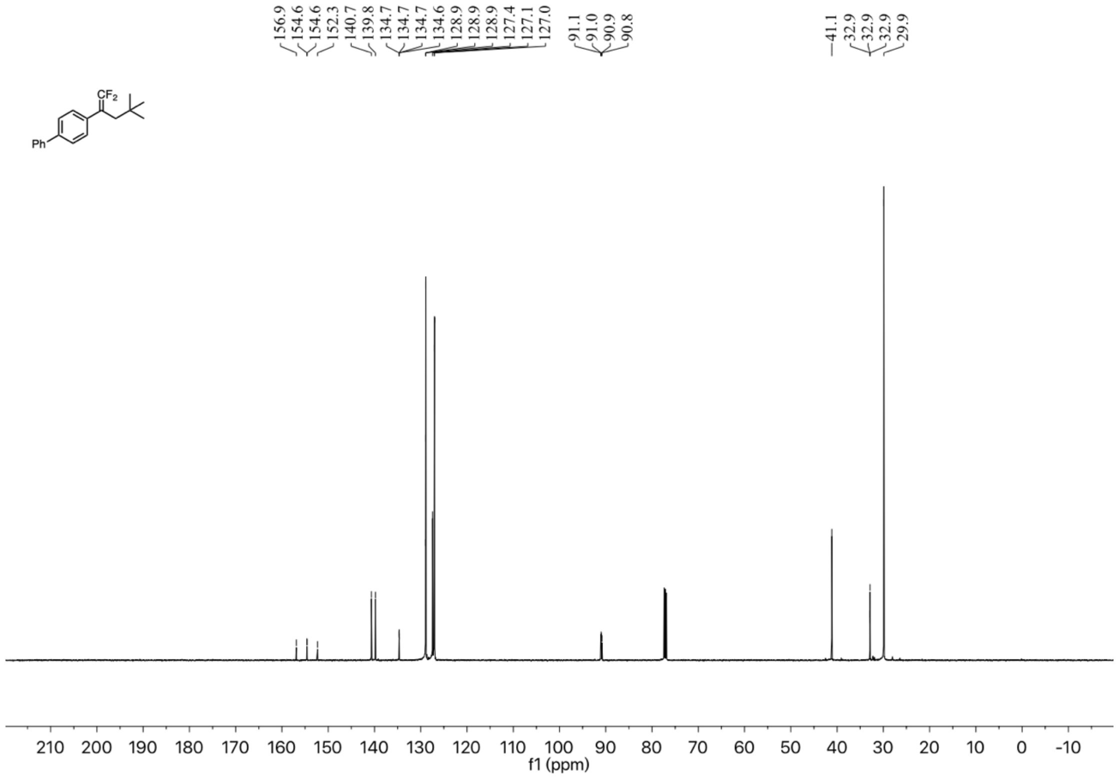


**$^{19}$F NMR (376 MHz, $CDCl_3$)** spectrum of **4-(1,1-difluoro-4,4-dimethylpent-1-en-2-yl)-1,1'-biphenyl (4)**

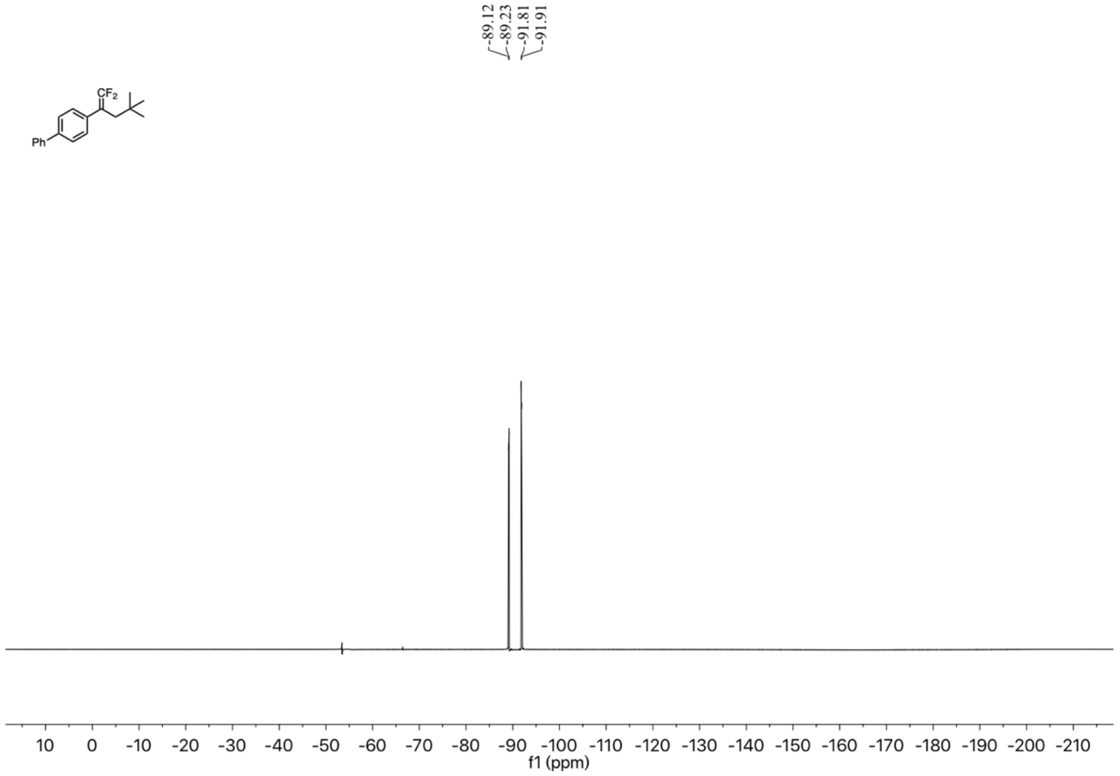

**$^{1}$H NMR (400 MHz, CDCl$_3$)** spectrum of **4-(7-(2,5-dimethylphenoxy)-1,1,1-trifluoro-4,4-dimethylheptan-2-yl)-1,1'-biphenyl (5)**

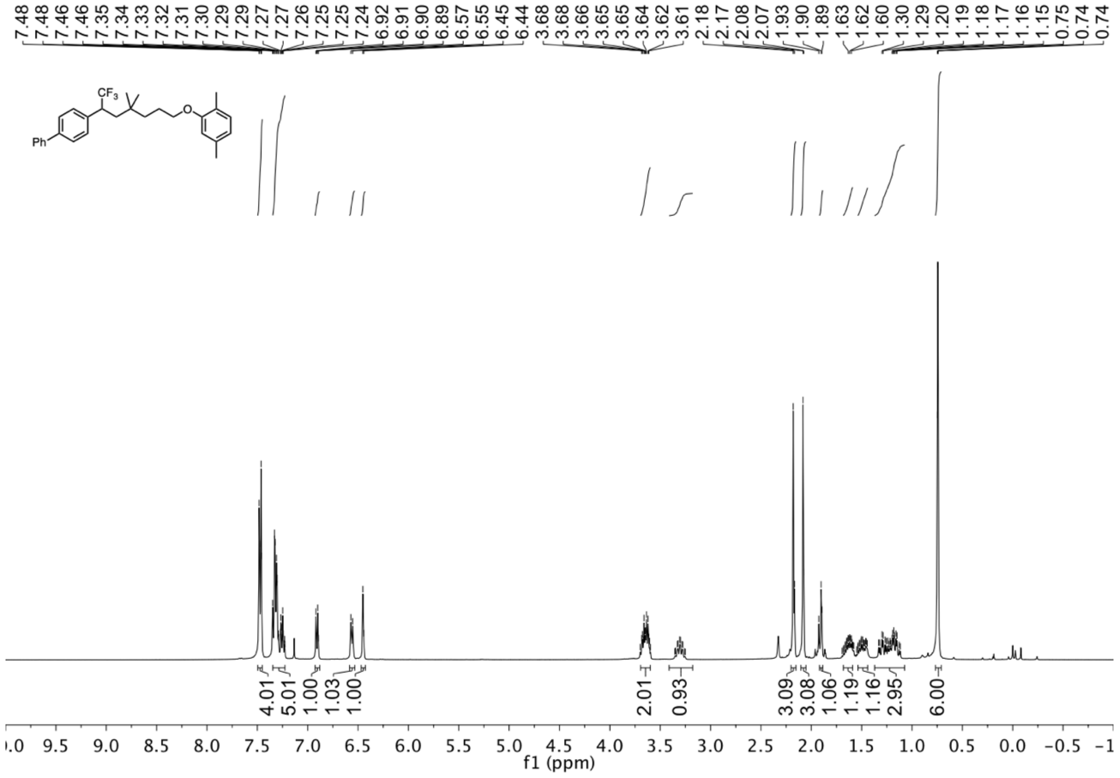


**$^{13}$C NMR (101 MHz, CDCl$_3$)** spectrum of **4-(7-(2,5-dimethylphenoxy)-1,1,1-trifluoro-4,4-dimethylheptan-2-yl)-1,1'-biphenyl (5)**

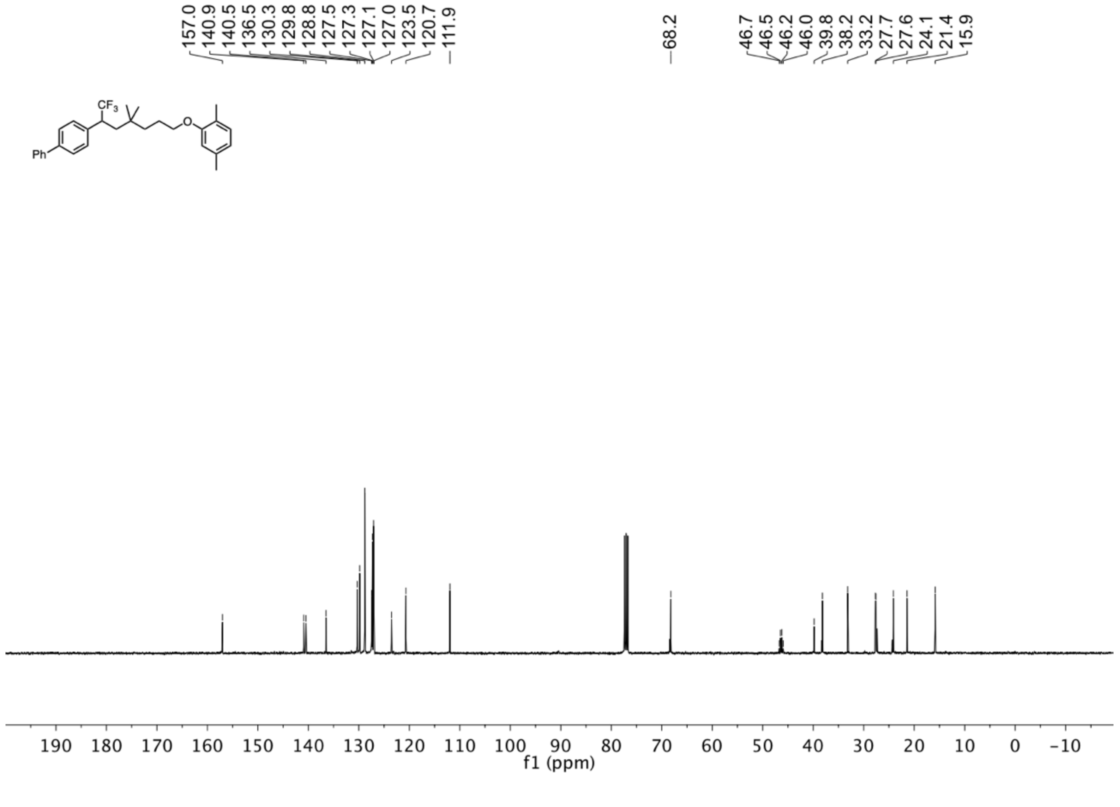

**$^{19}$F NMR (376 MHz, CDCl$_3$)** spectrum of **4-(7-(2,5-dimethylphenoxy)-1,1,1-trifluoro-4,4-dimethylheptan-2-yl)-1,1'-biphenyl (5)**

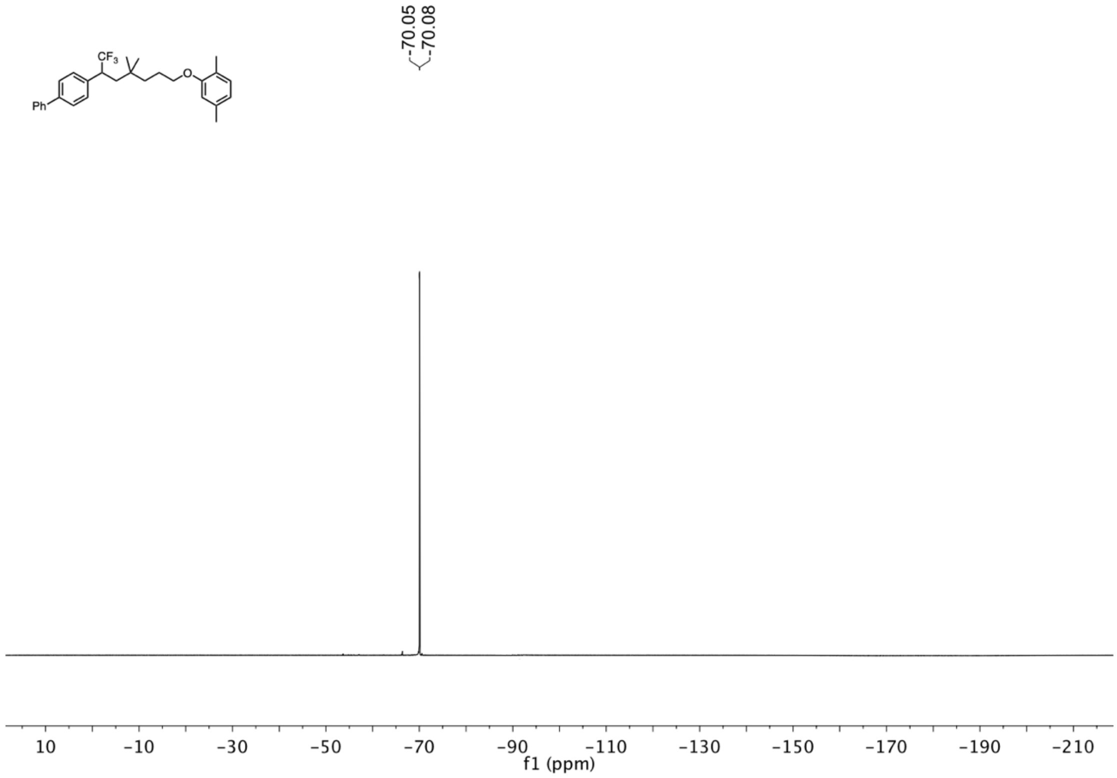


**$^{1}$H NMR (400 MHz, CDCl$_3$)** spectrum of **2-((6-(4-(*tert*-butyl)phenyl)-7,7,7-trifluoro-4,4-dimethylheptyl)oxy)-1,4-dimethylbenzene (6)**

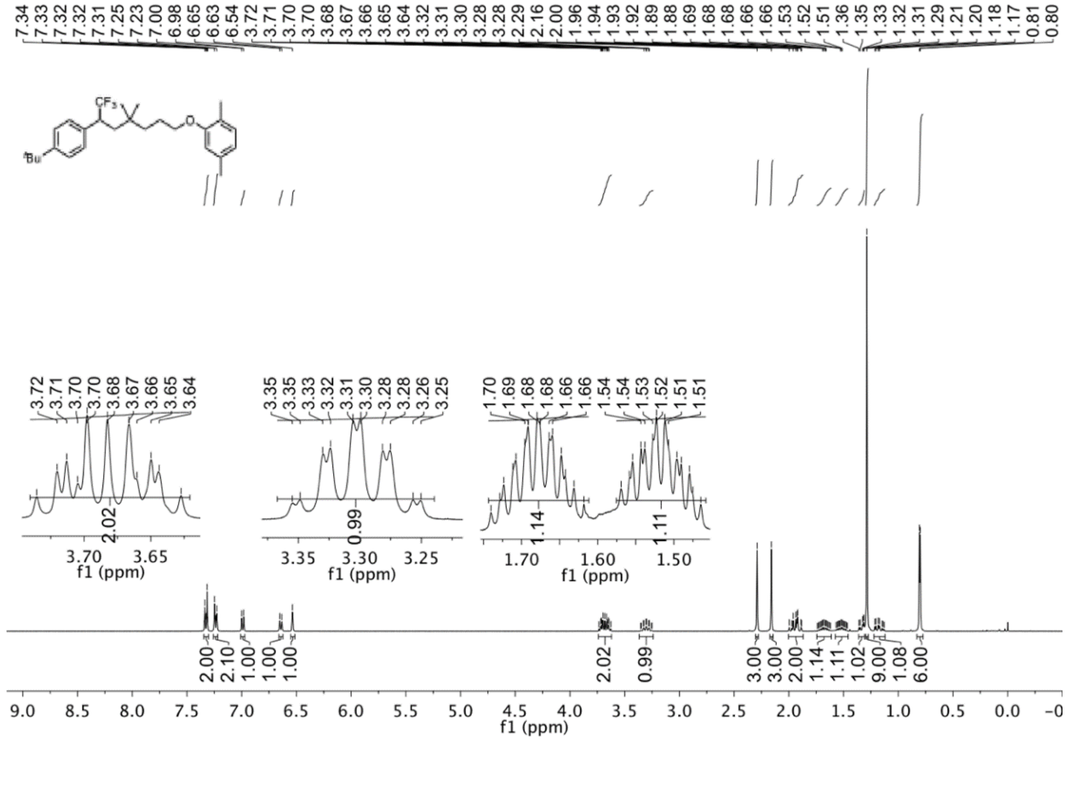

**$^{13}$C NMR (126 MHz, CDCl$_3$)** spectrum of **2-((6-(4-(*tert*-butyl)phenyl)-7,7,7-trifluoro-4,4-dimethylheptyl)oxy)-1,4-dimethylbenzene (6)**

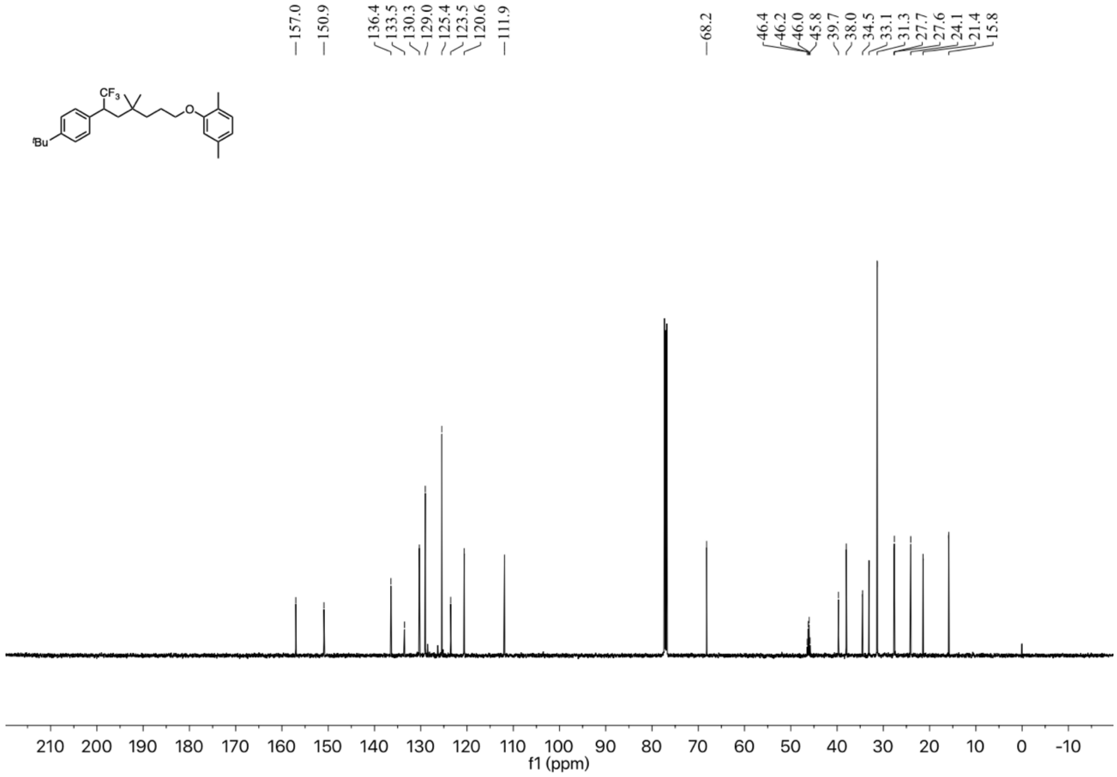


**$^{19}$F NMR (376 MHz, CDCl$_3$)** spectrum of **2-((6-(4-(*tert*-butyl)phenyl)-7,7,7-trifluoro-4,4-dimethylheptyl)oxy)-1,4-dimethylbenzene (6)**

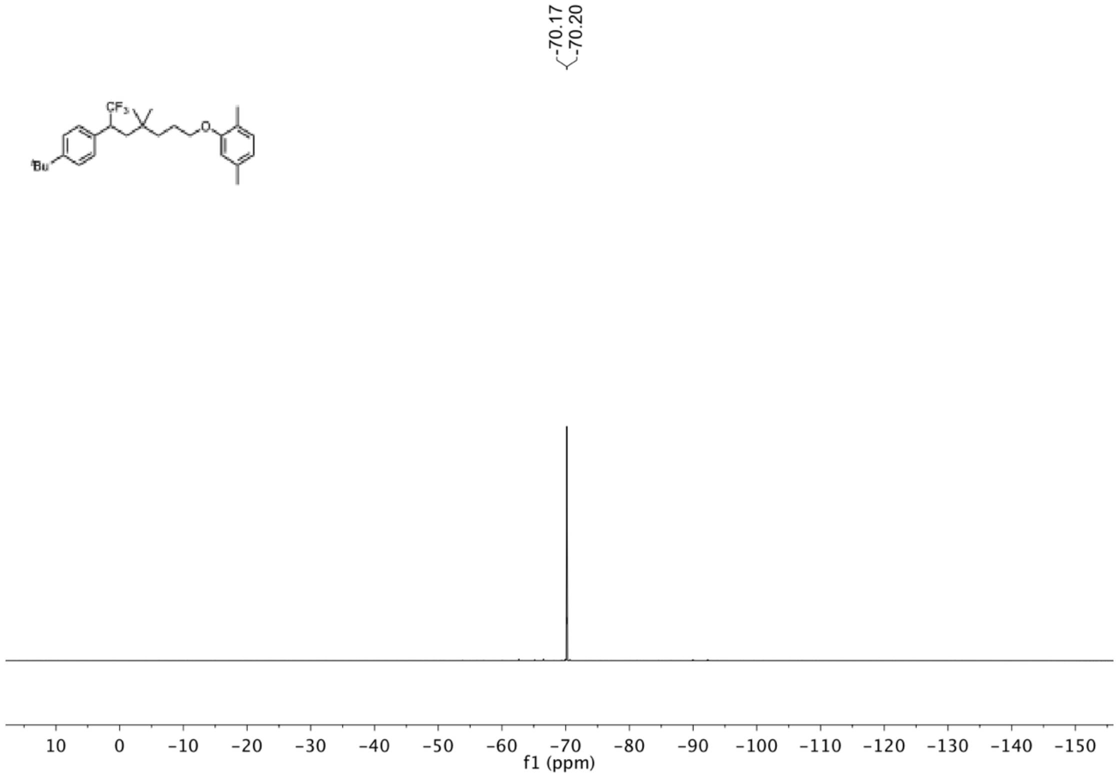

**$^{1}$H NMR (400 MHz, CDCl$_3$)** spectrum of **1,4-dimethyl-2-((7,7,7-trifluoro-6-(4-methoxyphenyl)-4,4-dimethylheptyl)oxy)benzene (7)**

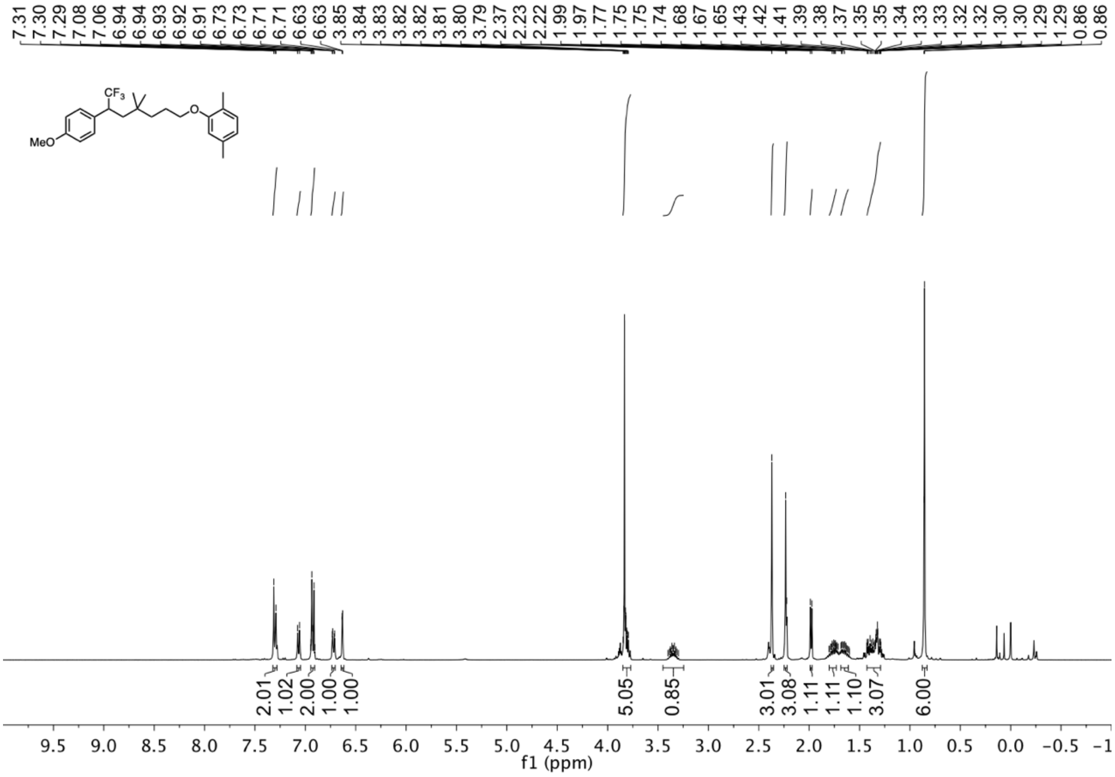

**$^{13}$C NMR (101 MHz, CDCl$_3$)** spectrum of **1,4-dimethyl-2-((7,7,7-trifluoro-6-(4-methoxyphenyl)-4,4-dimethylheptyl)oxy)benzene (7)**

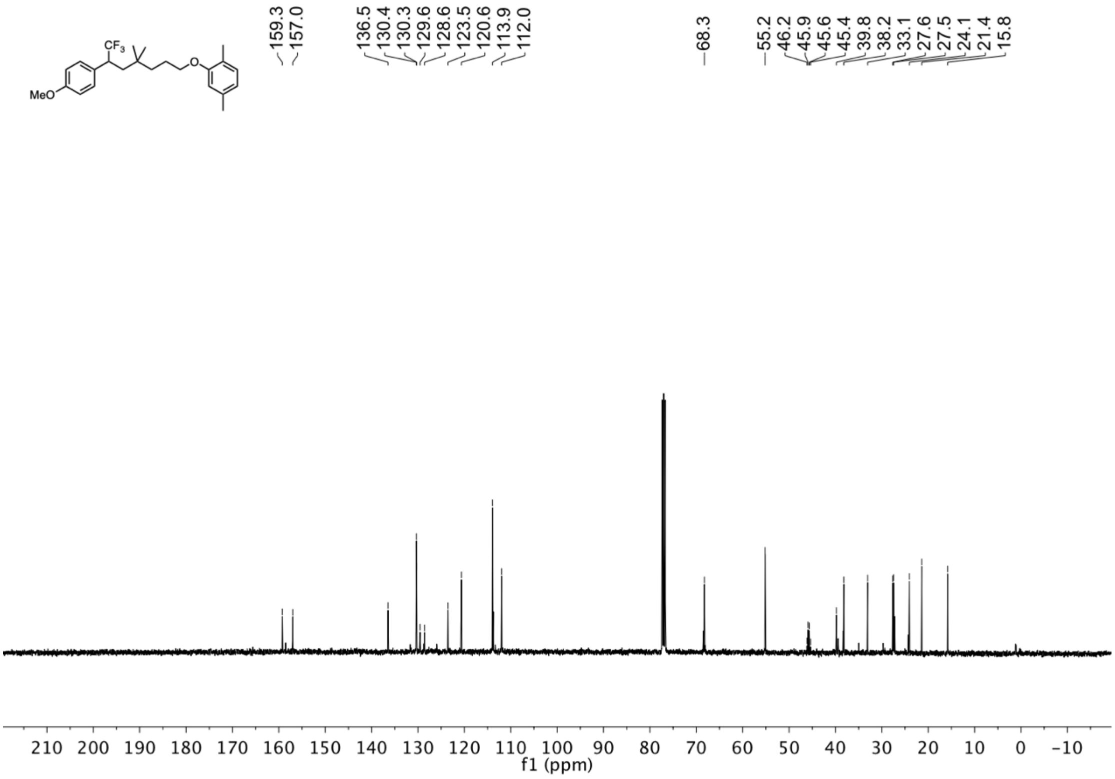

**$^{19}$F NMR (376 MHz, CDCl3)** spectrum of **1,4-dimethyl-2-((7,7,7-trifluoro-6-(4-methoxyphenyl)-4,4-dimethylheptyl)oxy)benzene (7)**

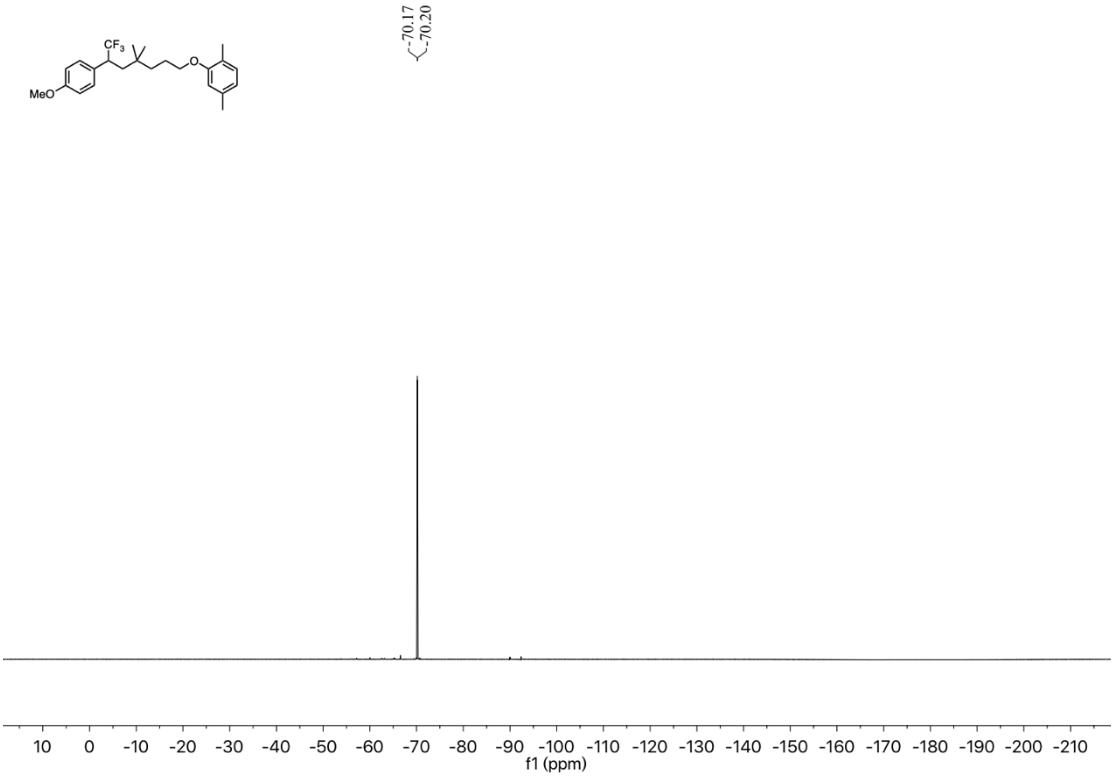


**$^{1}$H NMR (400 MHz, CDCl3)** spectrum of **(4-(7-(2,5-dimethylphenoxy)-1,1,1-trifluoro-4,4-dimethylheptan-2-yl)phenyl)(methyl)sulfane (8)**

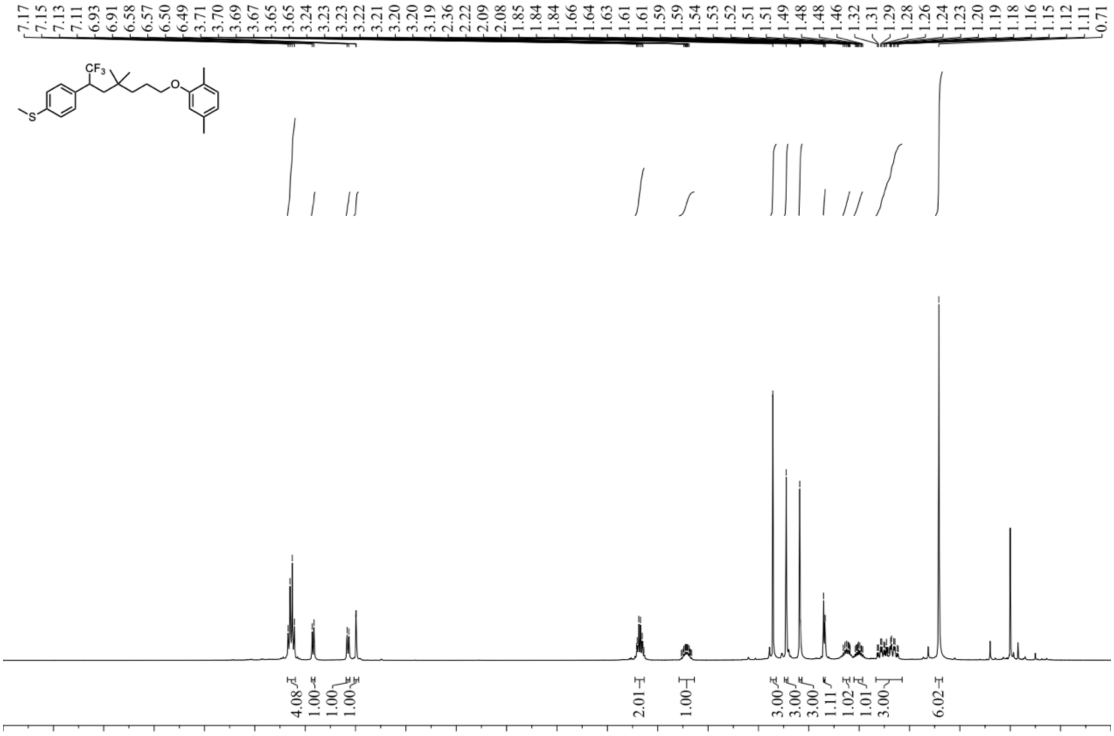

**$^{13}$C NMR (101 MHz, CDCl$_3$)** spectrum of **(4-(7-(2,5-dimethylphenoxy)-1,1,1-trifluoro-4,4-dimethylheptan-2-yl)phenyl)(methyl)sulfane (8)**

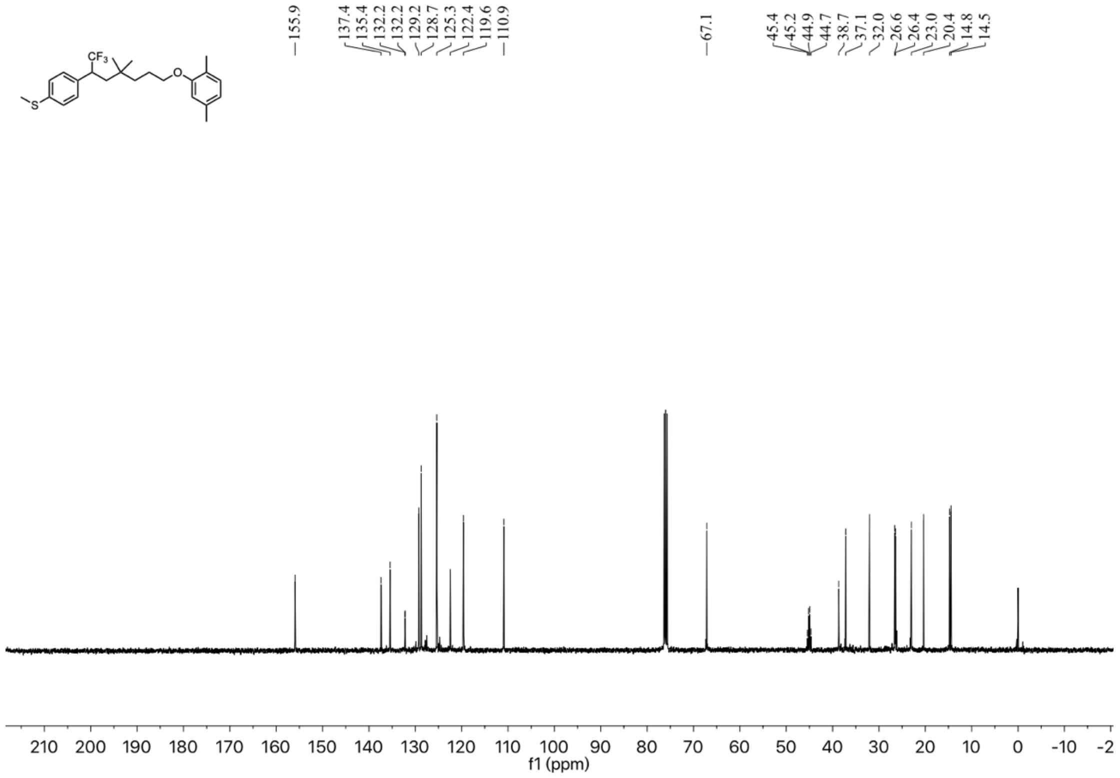


**$^{19}$F NMR (376 MHz, CDCl$_3$)** spectrum of **(4-(7-(2,5-dimethylphenoxy)-1,1,1-trifluoro-4,4-dimethylheptan-2-yl)phenyl)(methyl)sulfane (8)**

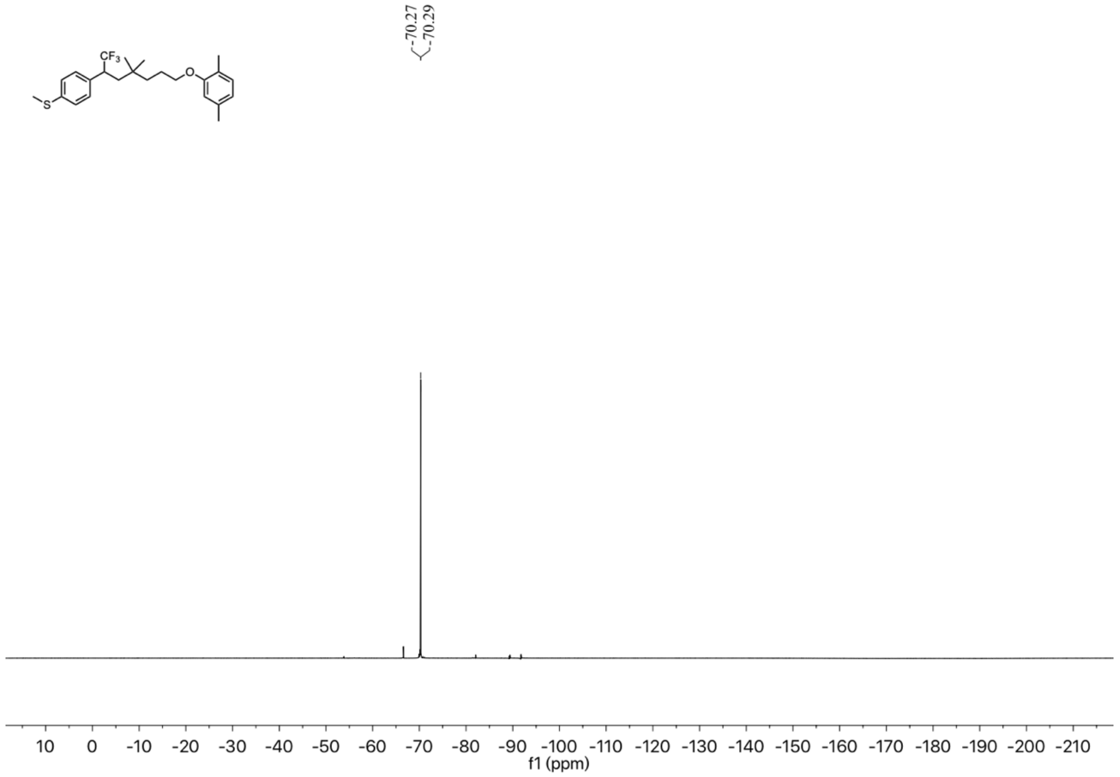

**$^{1}$H NMR (400 MHz, CDCl$_3$)** spectrum of **(4-(7-(2,5-dimethylphenoxy)-1,1,1-trifluoro-4,4-dimethylheptan-2-yl)phenyl)trimethylsilane (9)**

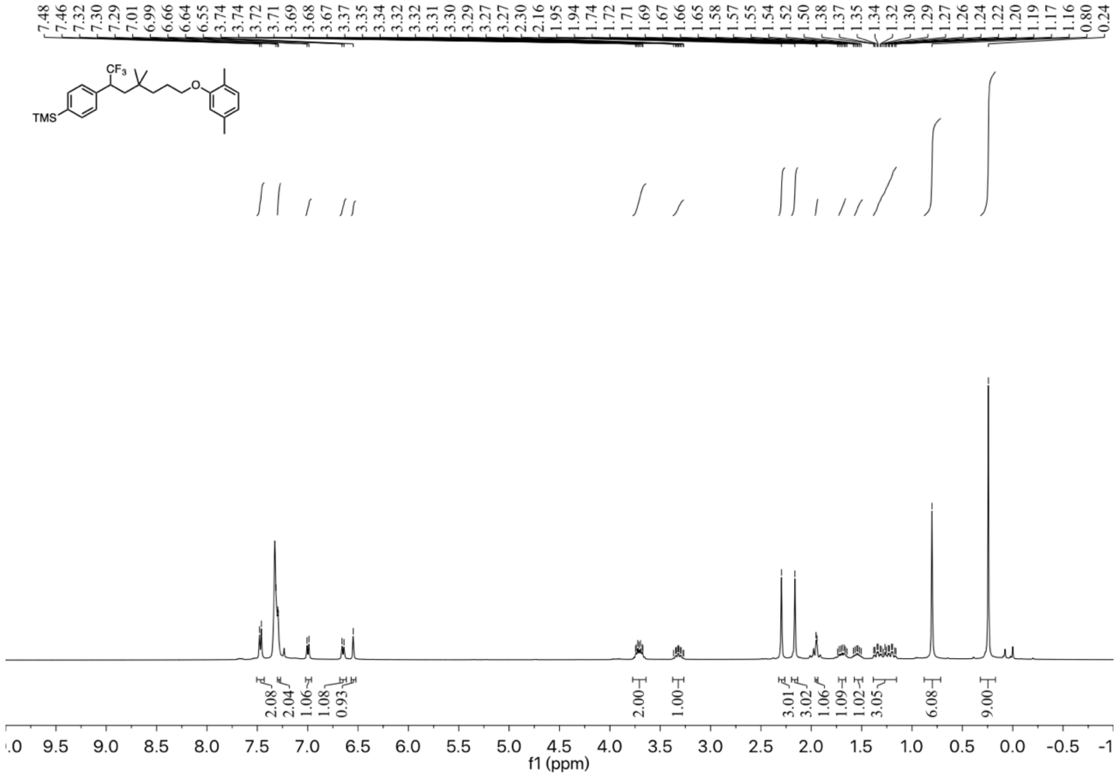

**$^{13}$C NMR (126 MHz, CDCl$_3$)** spectrum of **(4-(7-(2,5-dimethylphenoxy)-1,1,1-trifluoro-4,4-dimethylheptan-2-yl)phenyl)trimethylsilane (9)**

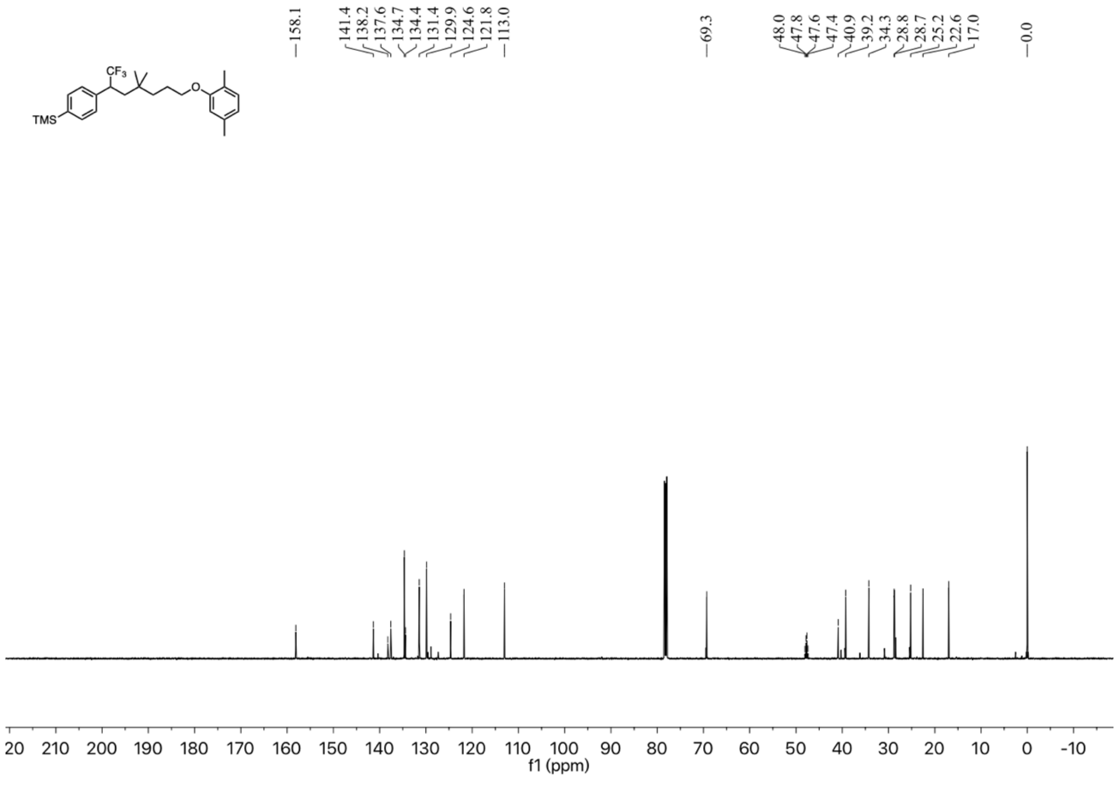

**$^{19}$F NMR (376 MHz, $CDCl_3$)** spectrum of **(4-(7-(2,5-dimethylphenoxy)-1,1,1-trifluoro-4,4-dimethylheptan-2-yl)phenyl)trimethylsilane (9)**

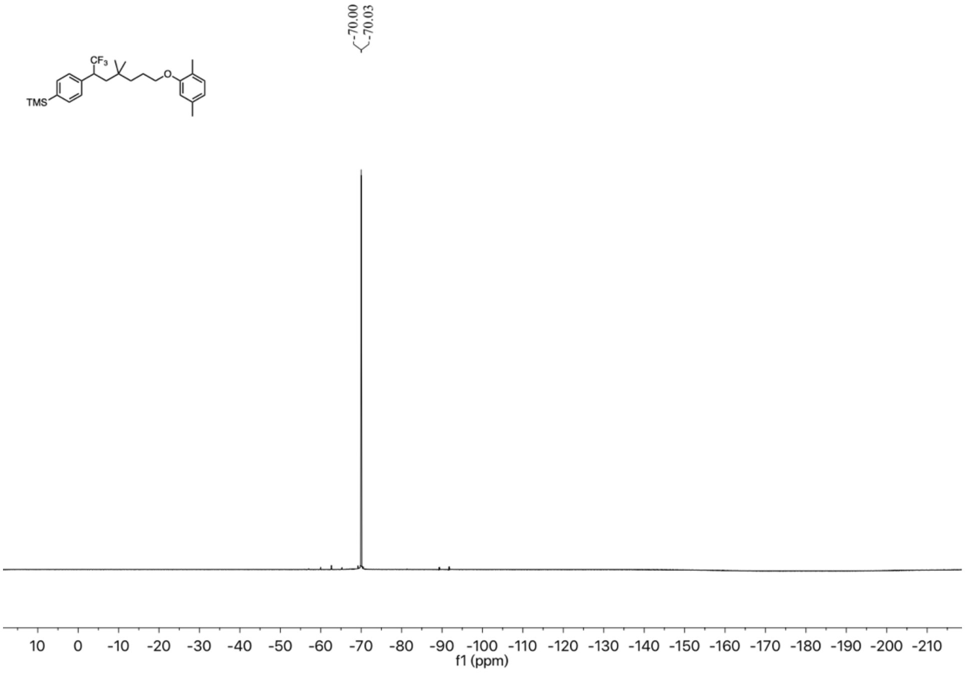

**$^{1}$H NMR (400 MHz, $CDCl_3$)** spectrum of **2-((6-(4-chlorophenyl)-7,7,7-trifluoro-4,4-dimethylheptyl)oxy)-1,4-dimethylbenzene (10)**

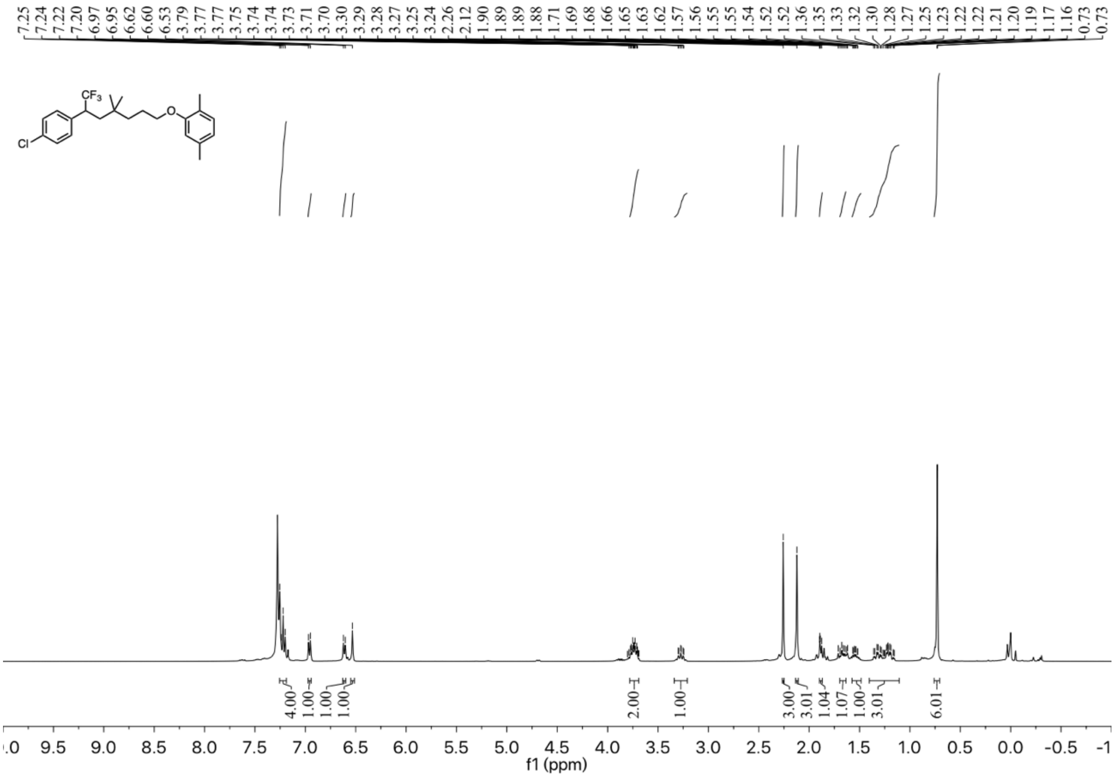

**$^{13}$C NMR (151 MHz, CDCl$_3$)** spectrum of **2-((6-(4-chlorophenyl)-7,7,7-trifluoro-4,4-dimethylheptyl)oxy)-1,4-dimethylbenzene (10)**

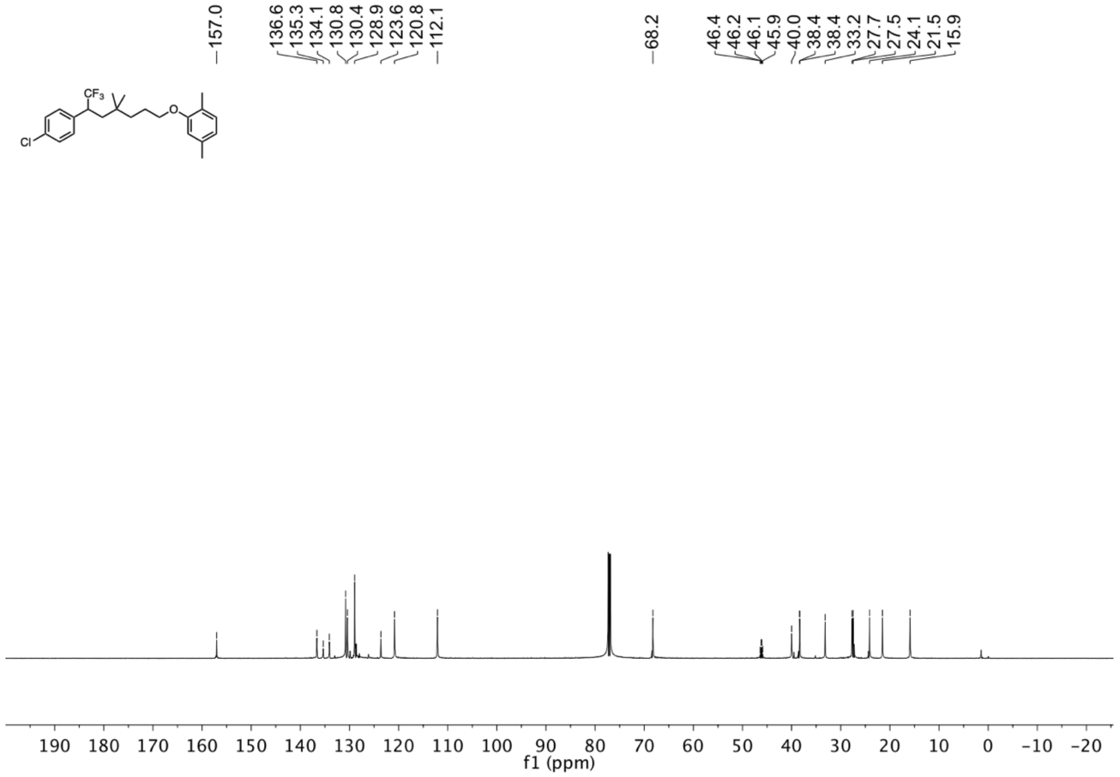

**$^{19}$F NMR (376 MHz, CDCl$_3$)** spectrum of **2-((6-(4-chlorophenyl)-7,7,7-trifluoro-4,4-dimethylheptyl)oxy)-1,4-dimethylbenzene (10)**

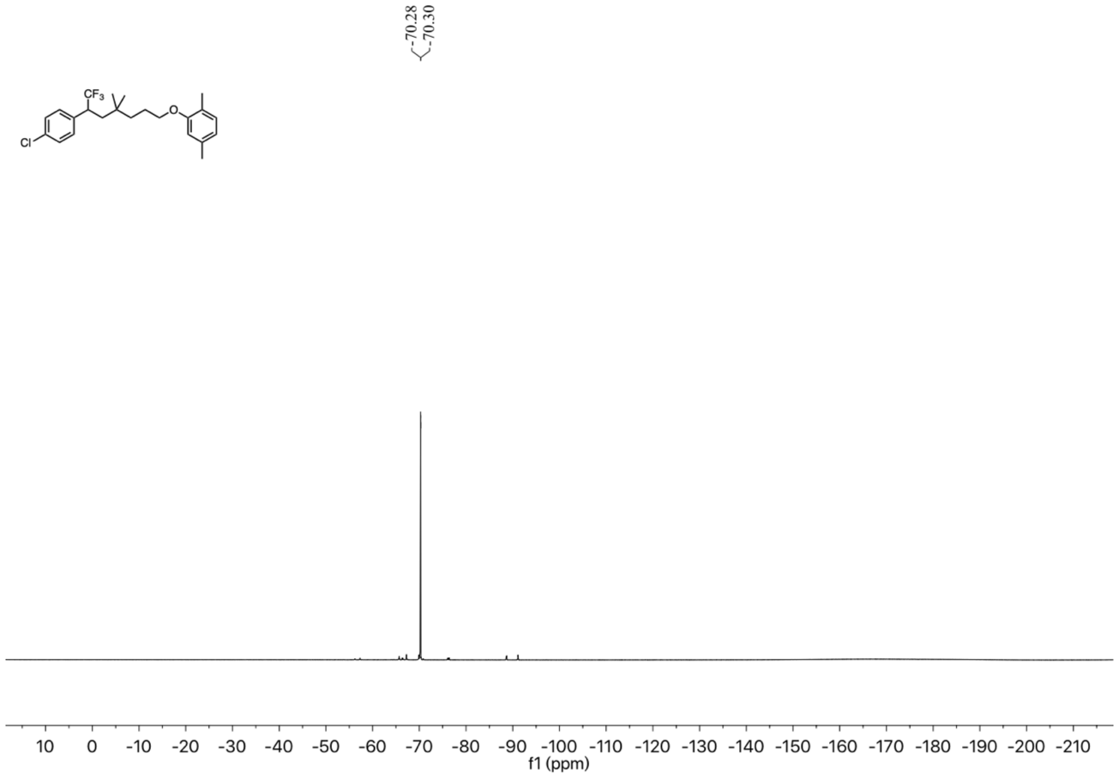

**$^1$H NMR (400 MHz, CDCl$_3$)** spectrum of **4-(7-(2,5-dimethylphenoxy)-1,1,1-trifluoro-4,4-dimethylheptan-2-yl)benzonitrile (11)**

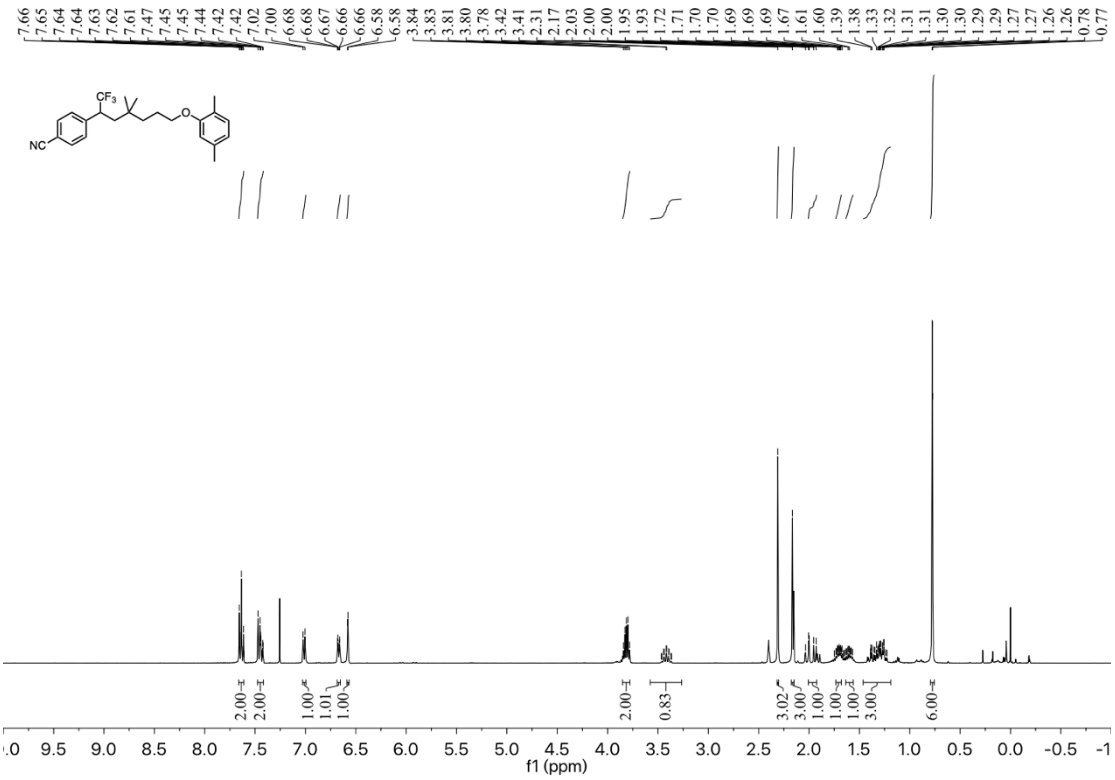

**$^{13}$C NMR (126 MHz, CDCl$_3$)** spectrum of **4-(7-(2,5-dimethylphenoxy)-1,1,1-trifluoro-4,4-dimethylheptan-2-yl)benzonitrile (11)**

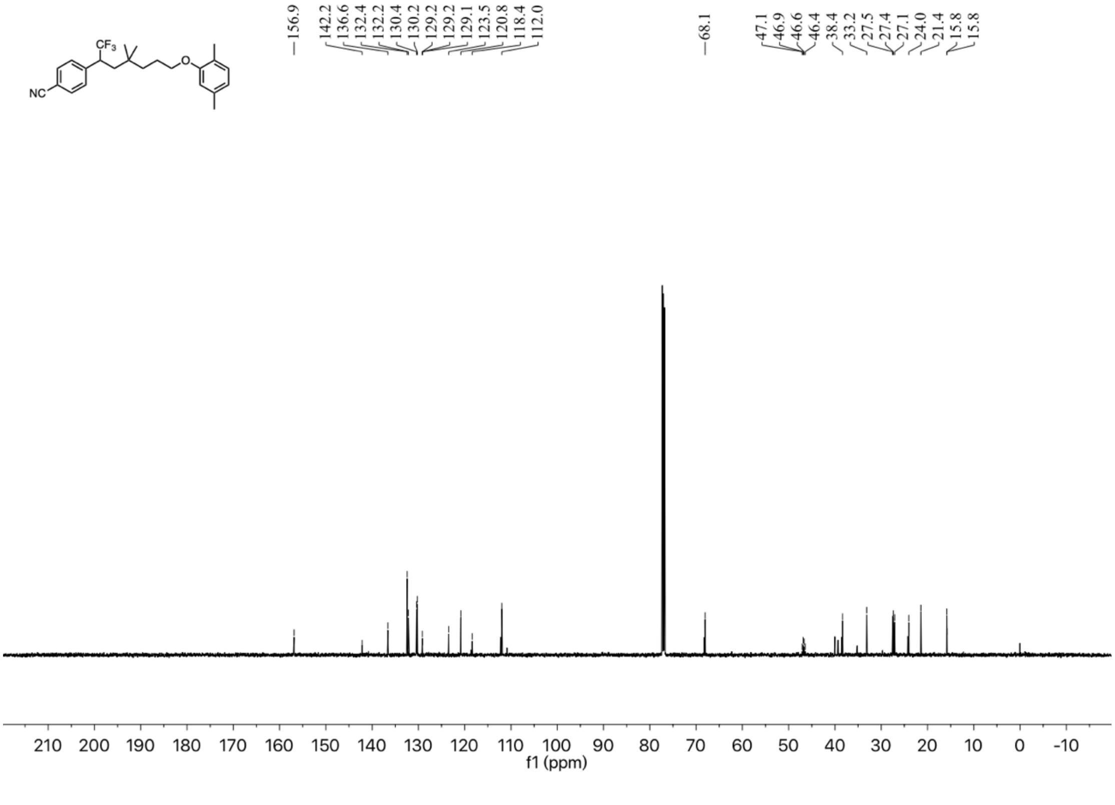

**$^{19}$F NMR (376 MHz, CDCl$_3$)** spectrum of spectrum of **4-(7-(2,5-dimethylphenoxy)-1,1,1-trifluoro-4,4-dimethylheptan-2-yl)benzonitrile (11)**

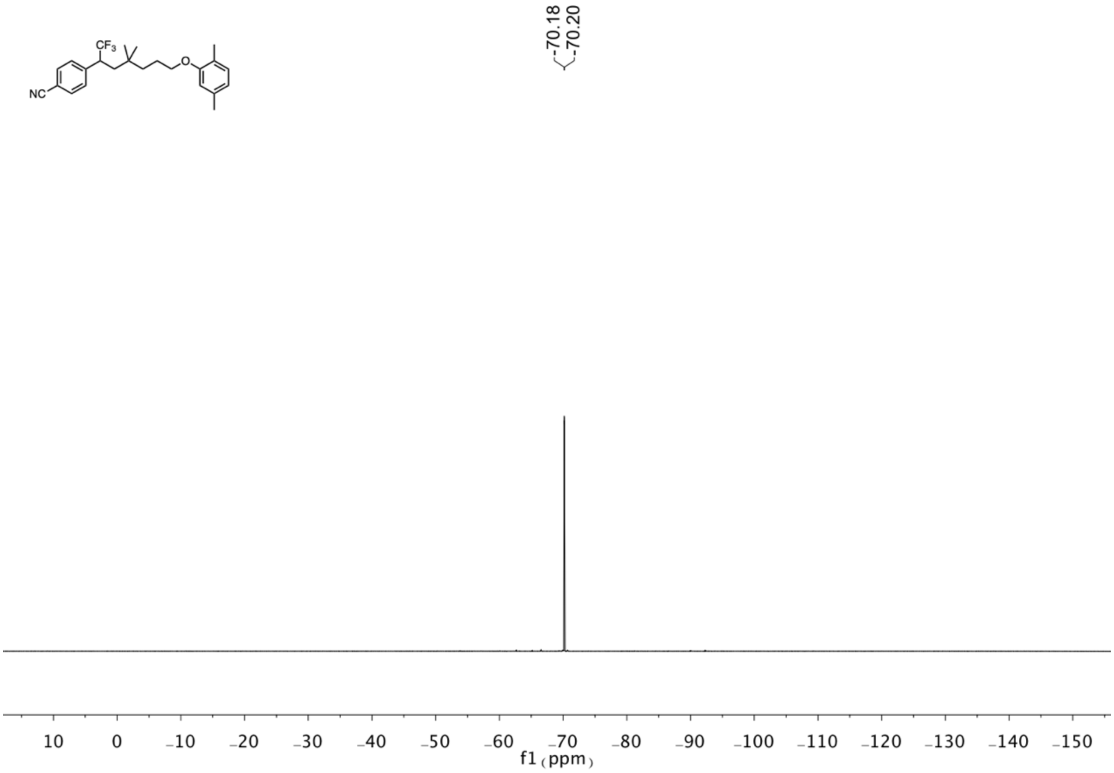


**$^{1}$H NMR (400 MHz, CDCl$_3$)** spectrum of **1,4-dimethyl-2-((7,7,7-trifluoro-4,4-dimethyl-6-(4-(trifluoromethyl)phenyl)heptyl)oxy)benzene (12)**

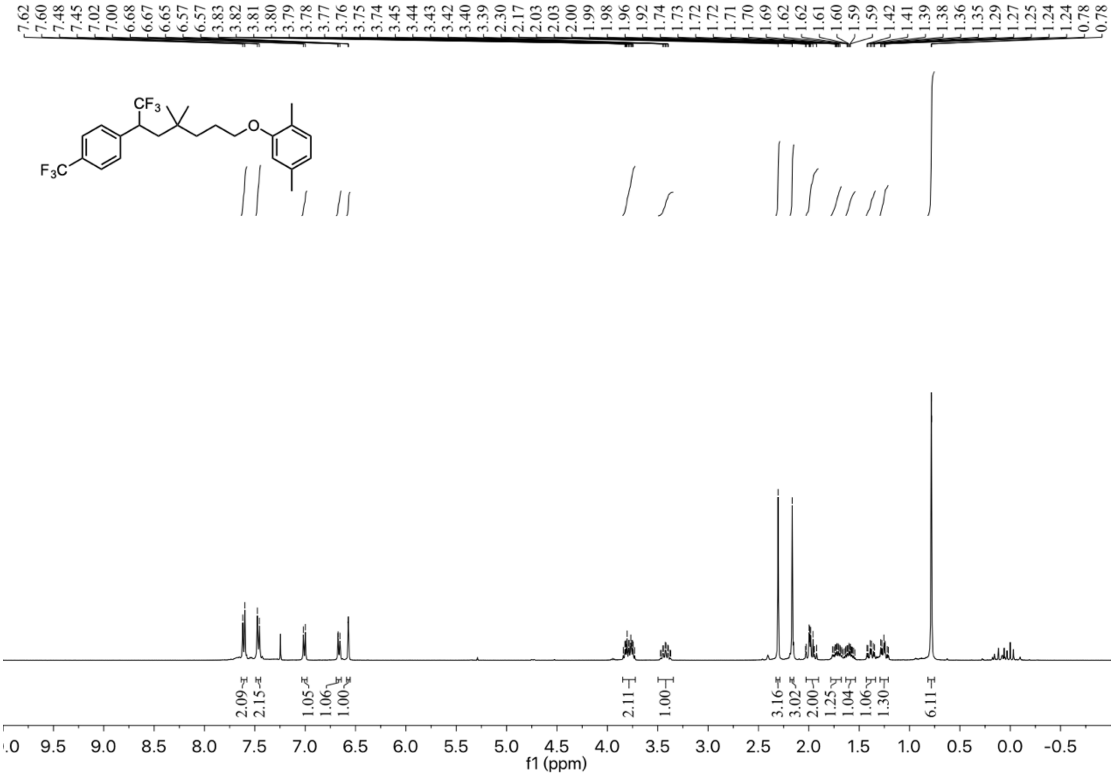

$^{13}$C NMR (126 MHz, CDCl$_3$)** spectrum of **1,4-dimethyl-2-((7,7,7-trifluoro-4,4-dimethyl-6-(4-(trifluoromethyl)phenyl)heptyl)oxy)benzene (12)**

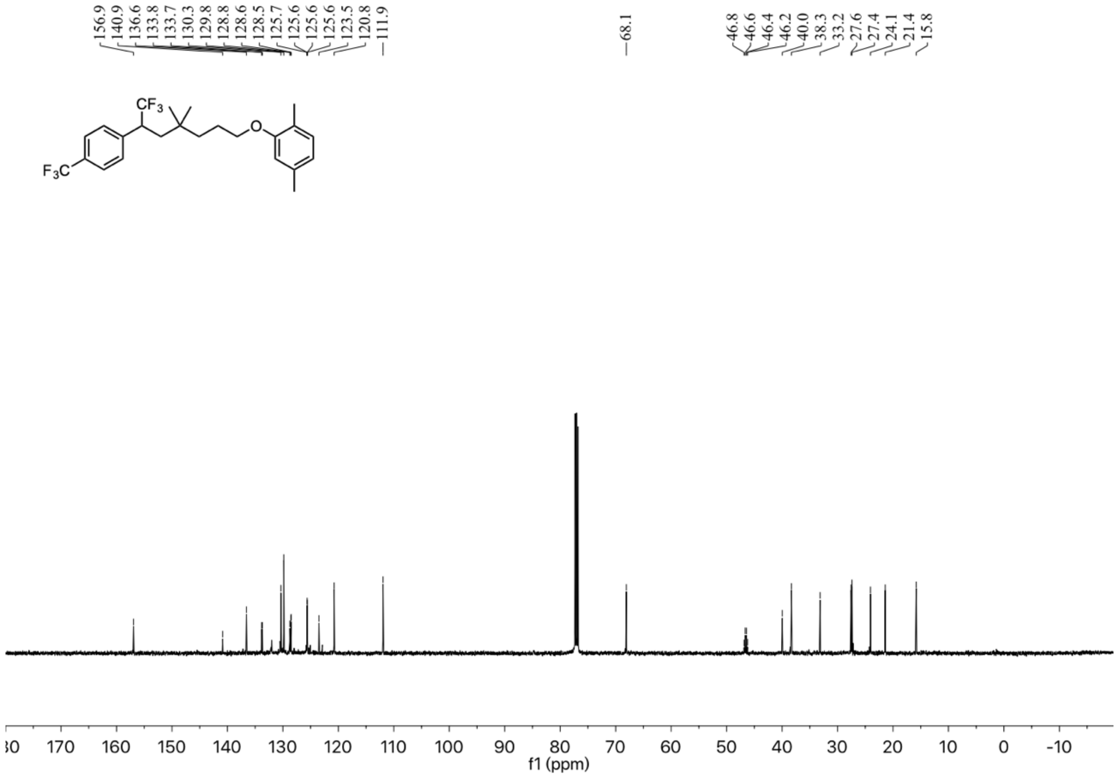


$^{19}$F NMR (376 MHz, CDCl$_3$)** spectrum of **1,4-dimethyl-2-((7,7,7-trifluoro-4,4-dimethyl-6-(4-(trifluoromethyl)phenyl)heptyl)oxy)benzene (12)**

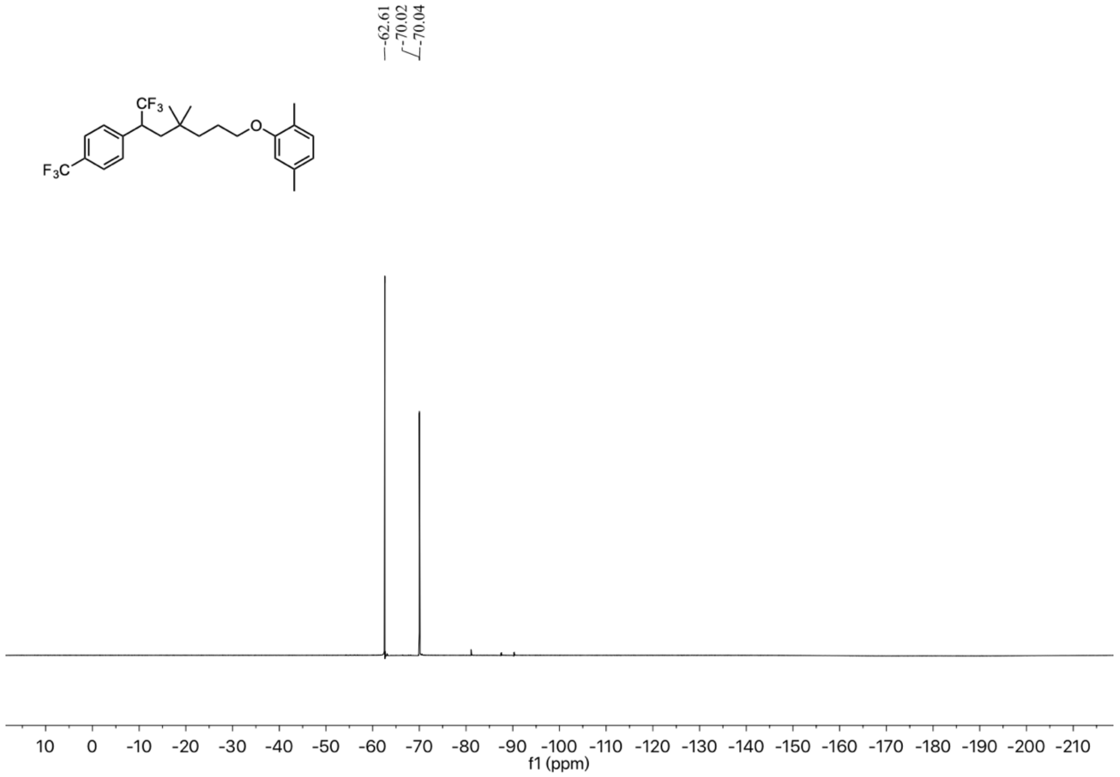

$^{1}H$ **NMR (500 MHz, $CDCl_3$)** spectrum of **ethyl 4-(7-(2,5-dimethylphenoxy)-1,1,1-trifluoro-4,4-dimethylheptan-2-yl)benzoate (13)**

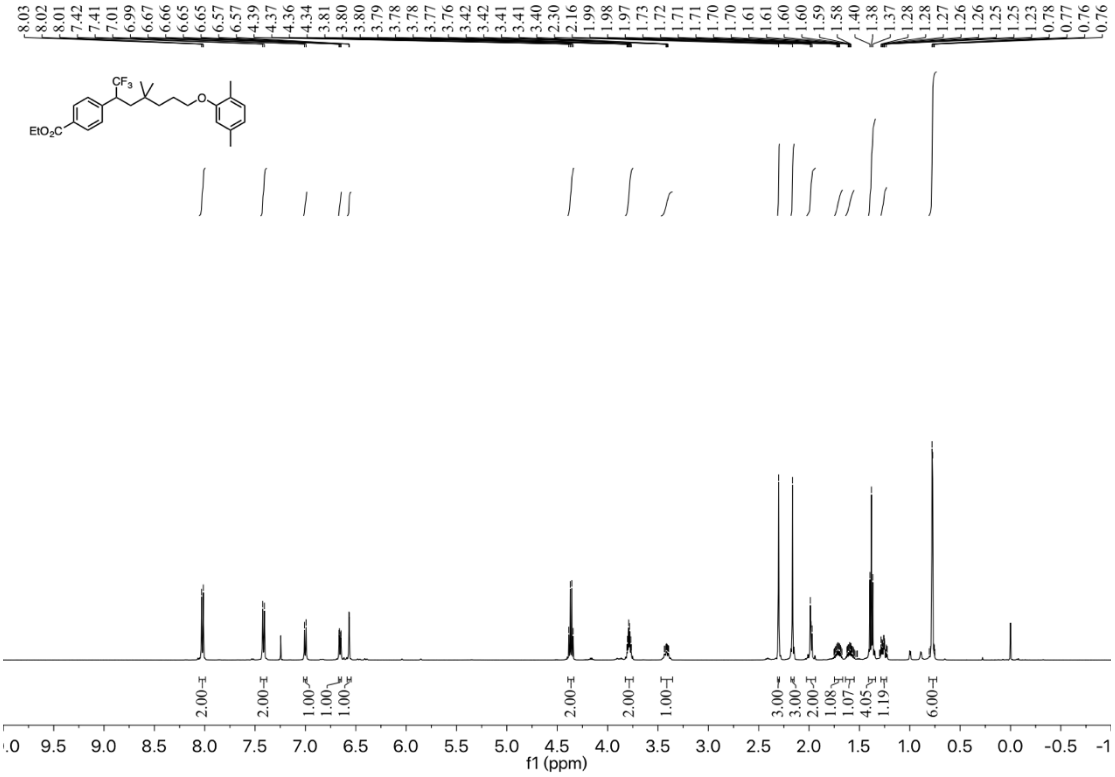


$^{13}C$ **NMR (126 MHz, $CDCl_3$)** spectrum of **ethyl 4-(7-(2,5-dimethylphenoxy)-1,1,1-trifluoro-4,4-dimethylheptan-2-yl)benzoate (13)**

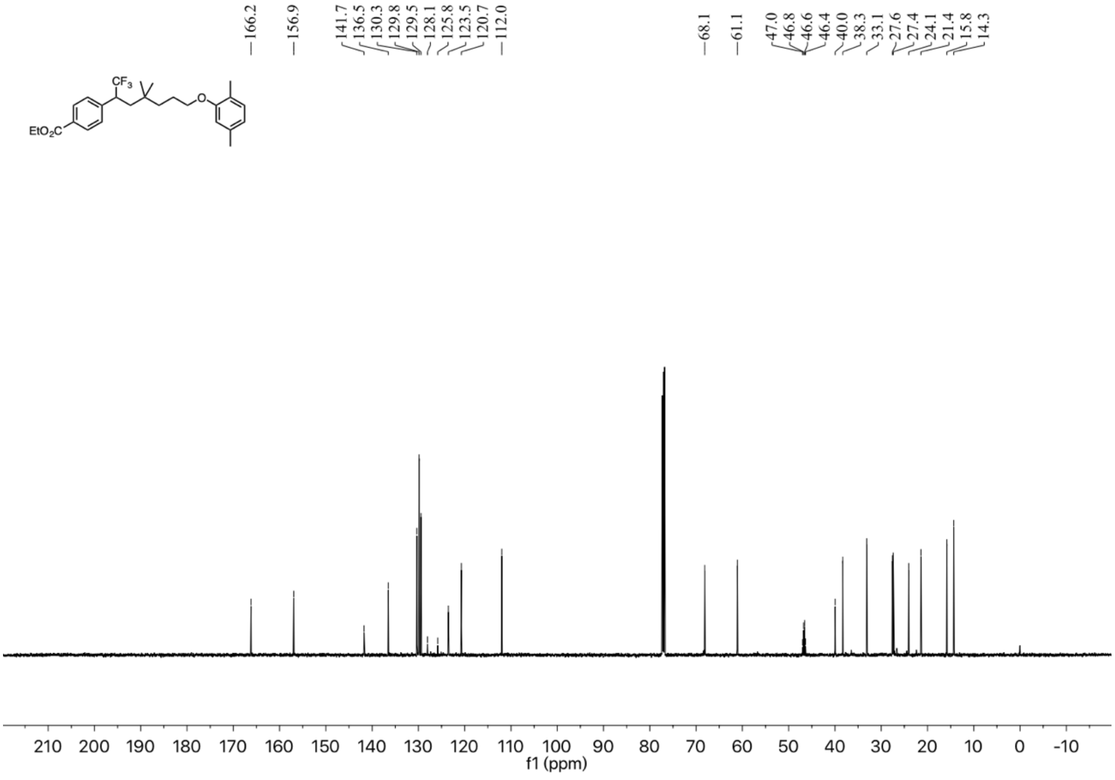

**$^{19}F$ NMR (471 MHz, $CDCl_3$)** spectrum of **ethyl 4-(7-(2,5-dimethylphenoxy)-1,1,1-trifluoro-4,4-dimethylheptan-2-yl)benzoate (13)**

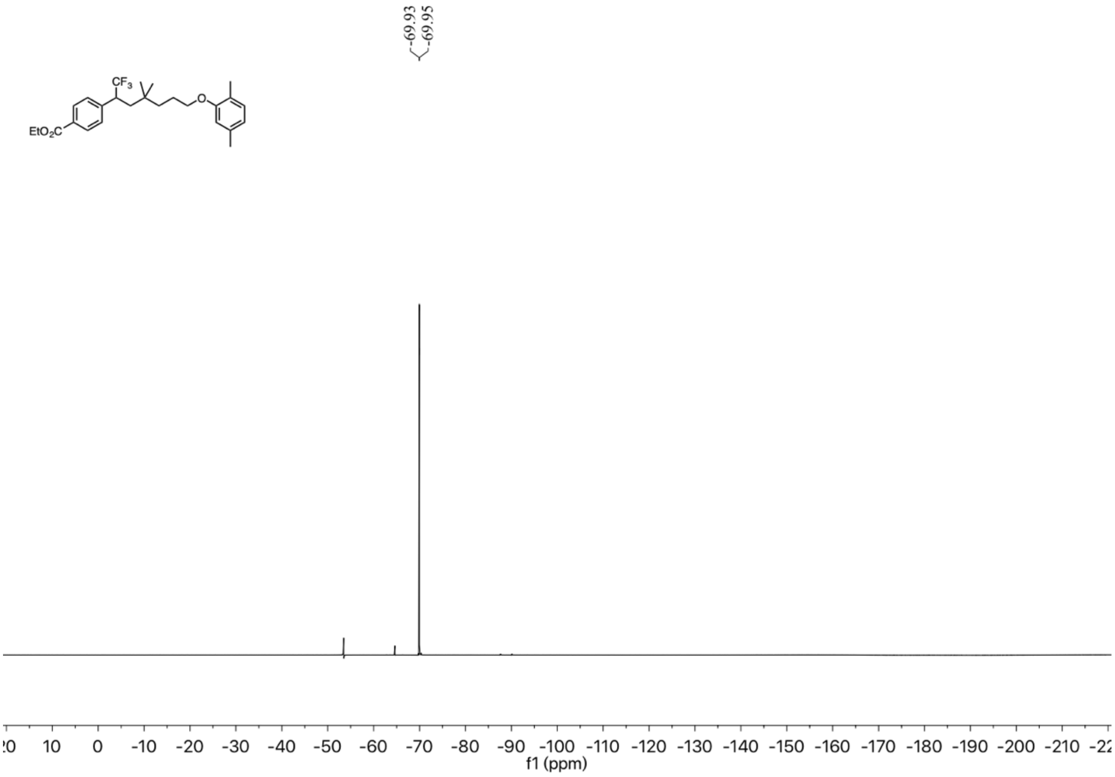


**$^{1}H$ NMR (500 MHz, $CDCl_3$)** spectrum of ***N*-(3-(7-(2,5-dimethylphenoxy)-1,1,1-trifluoro-4,4-dimethylheptan-2-yl)phenyl)acetamide (14)**

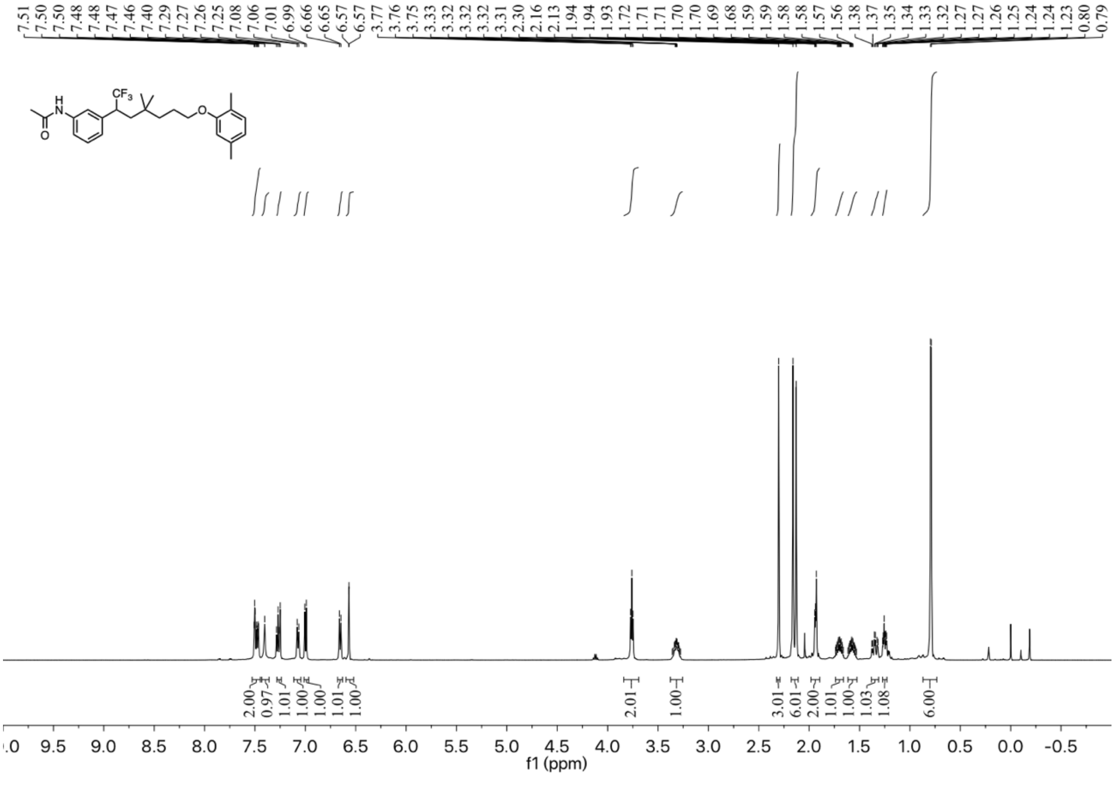

**$^{13}$C NMR (126 MHz, CDCl$_3$)** spectrum of ***N*-(3-(7-(2,5-dimethylphenoxy)-1,1,1-trifluoro-4,4-dimethylheptan-2-yl)phenyl)acetamide (14)**

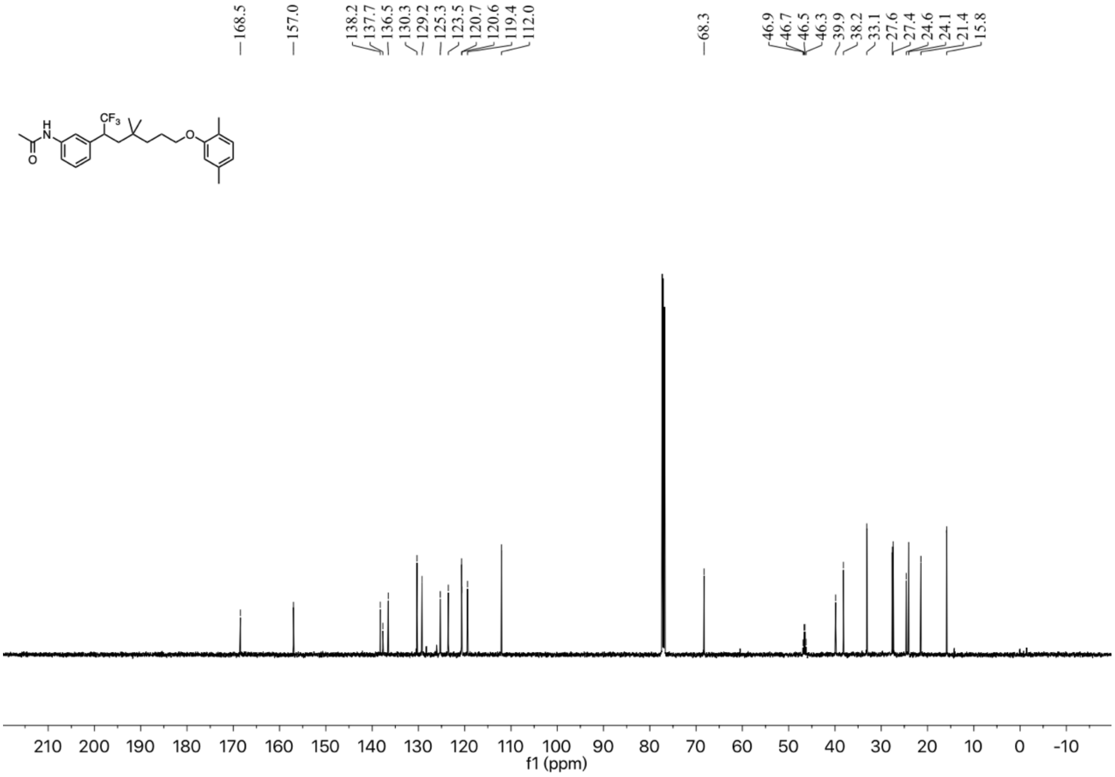


**$^{19}$F NMR (471 MHz, CDCl$_3$)** spectrum of ***N*-(3-(7-(2,5-dimethylphenoxy)-1,1,1-trifluoro-4,4-dimethylheptan-2-yl)phenyl)acetamide (14)**

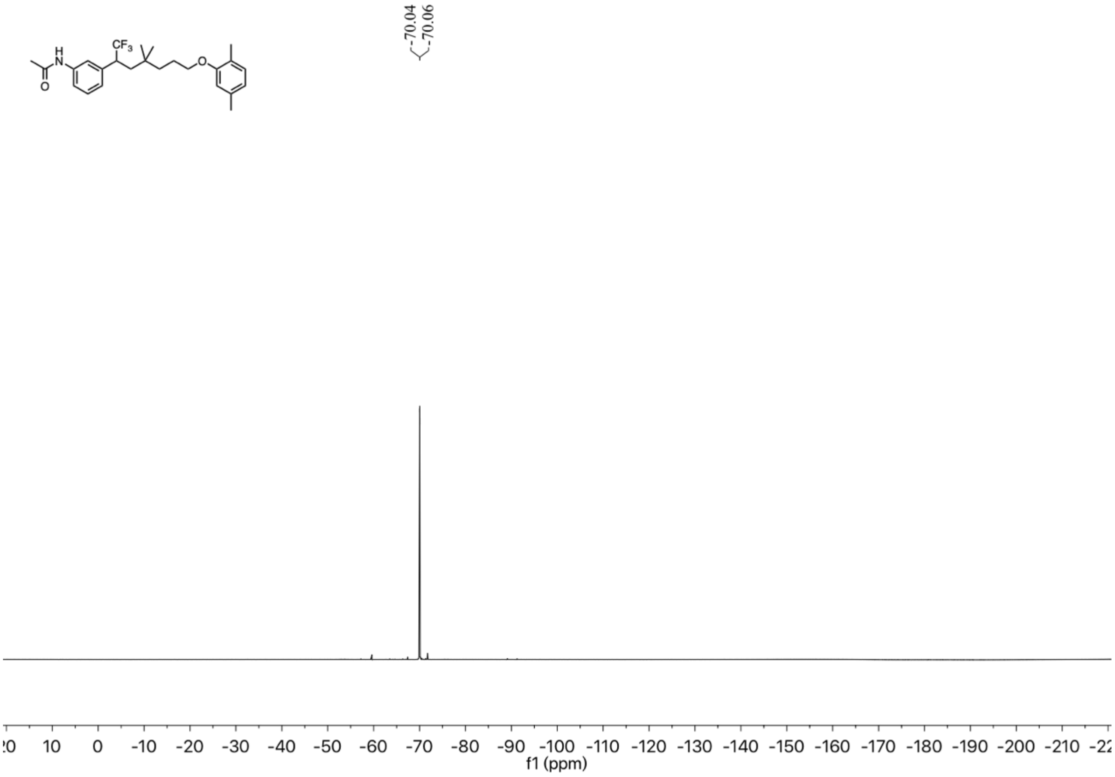

**$^{1}H$ NMR (400 MHz, $CDCl_3$)** spectrum of **5-(7-(2,5-dimethylphenoxy)-1,1,1-trifluoro-4,4-dimethylheptan-2-yl)benzo[*d*][1,3]dioxole (15)**

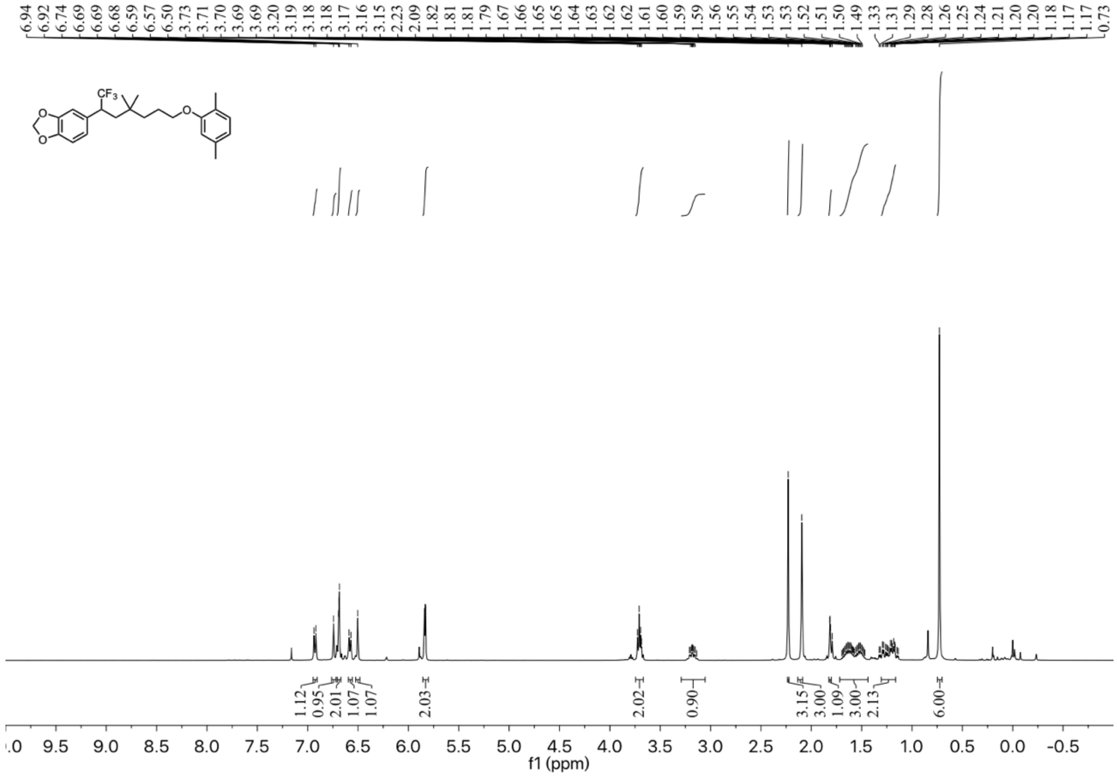


**$^{13}C$ NMR (151 MHz, $CDCl_3$)** spectrum of **5-(7-(2,5-dimethylphenoxy)-1,1,1-trifluoro-4,4-dimethylheptan-2-yl)benzo[*d*][1,3]dioxole (15)**

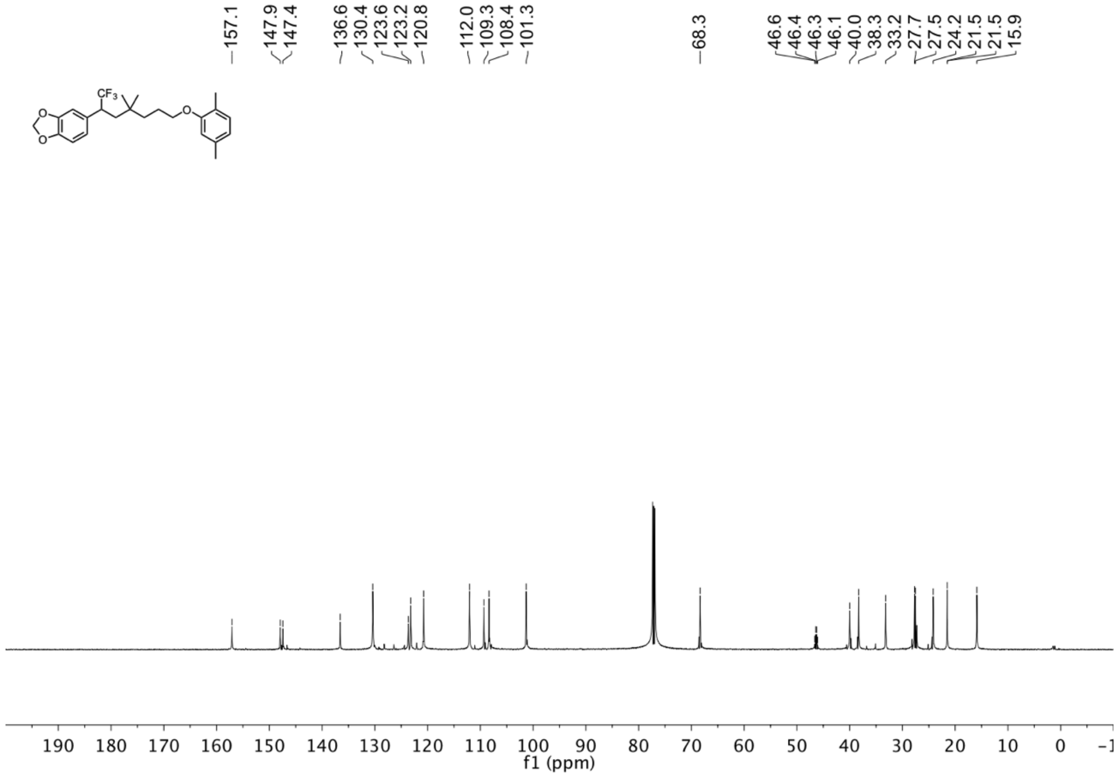

**$^{19}$F NMR (376 MHz, CDCl$_3$)** spectrum of **5-(7-(2,5-dimethylphenoxy)-1,1,1-trifluoro-4,4-dimethylheptan-2-yl)benzo[*d*][1,3]dioxole (15)**

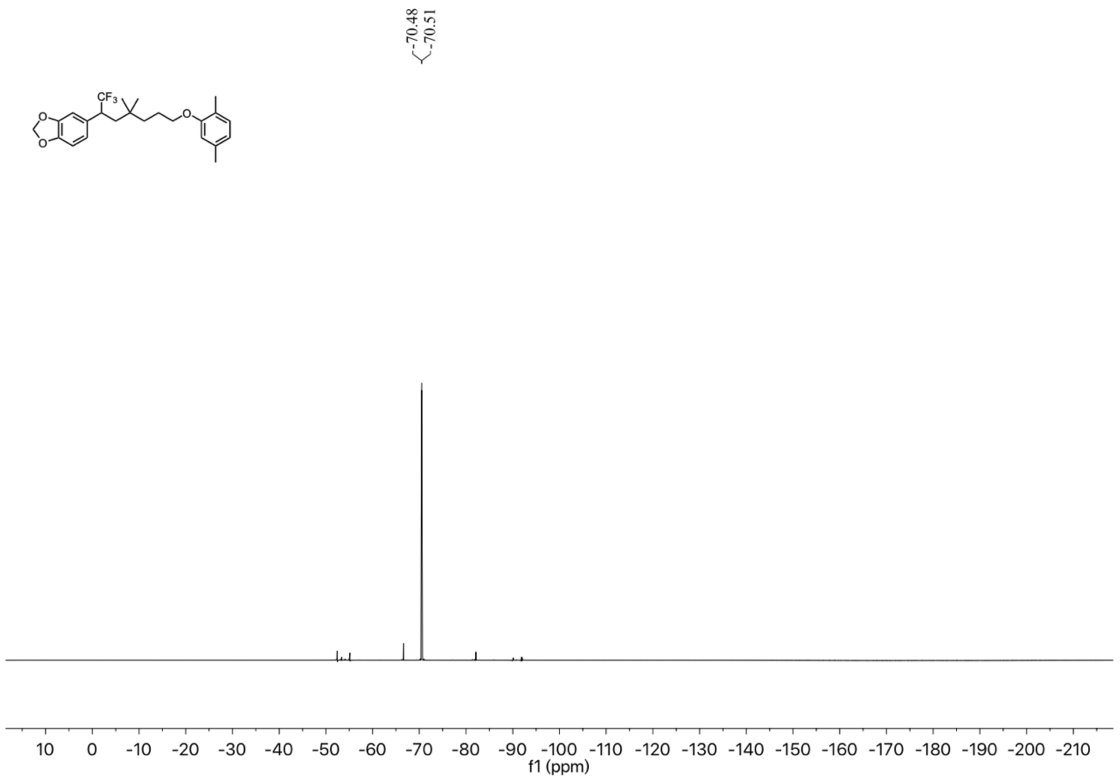

**$^{1}$H NMR (500 MHz, CDCl$_3$)** spectrum of **3-(7-(2,5-dimethylphenoxy)-1,1,1-trifluoro-4,4-dimethylheptan-2-yl)dibenzo[*b*,*d*]furan (16)**

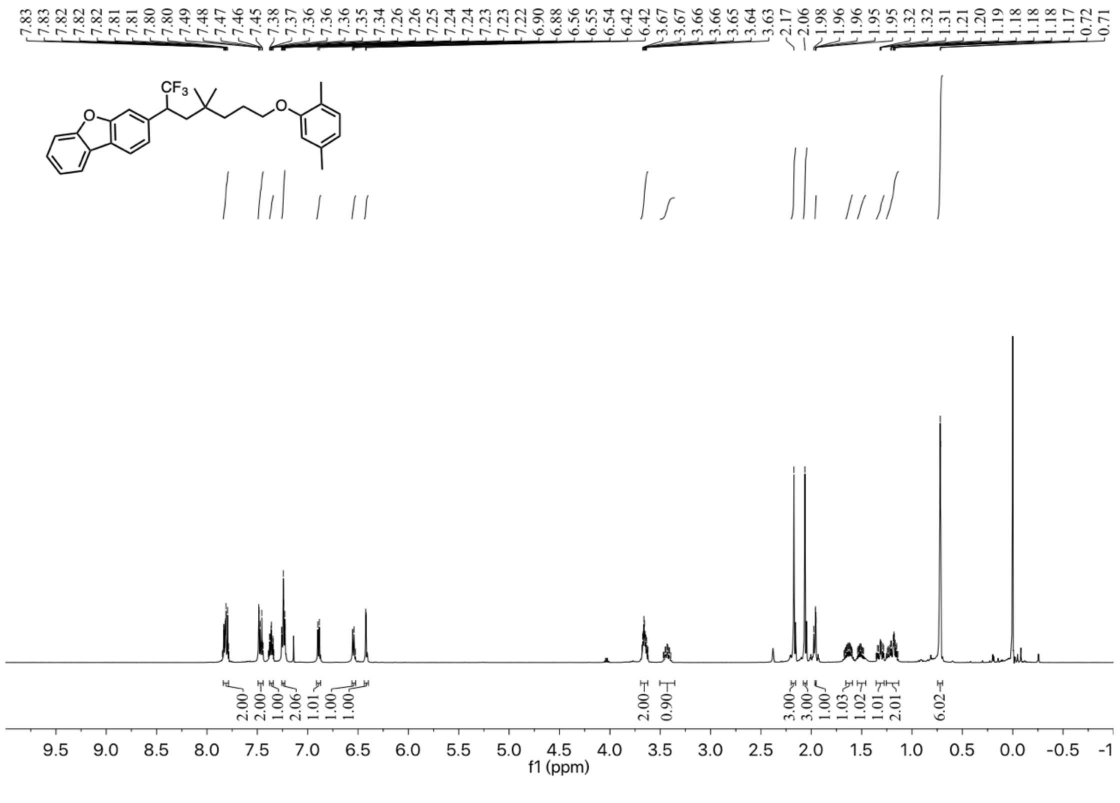

**$^{13}$C NMR (126 MHz, CDCl$_3$)** spectrum of **3-(7-(2,5-dimethylphenoxy)-1,1,1-trifluoro-4,4-dimethylheptan-2-yl)dibenzo[*b*,*d*]furan (16)**

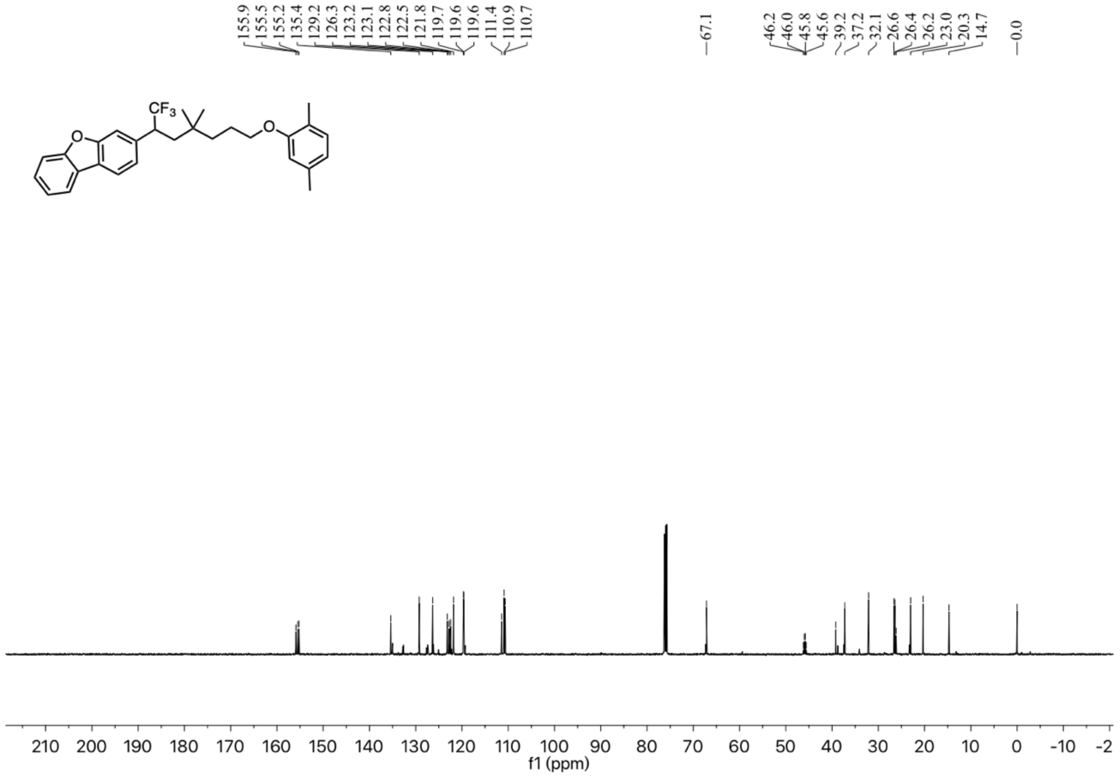


**$^{19}$F NMR (471 MHz, CDCl$_3$)** spectrum of **3-(7-(2,5-dimethylphenoxy)-1,1,1-trifluoro-4,4-dimethylheptan-2-yl)dibenzo[*b*,*d*]furan (16)**

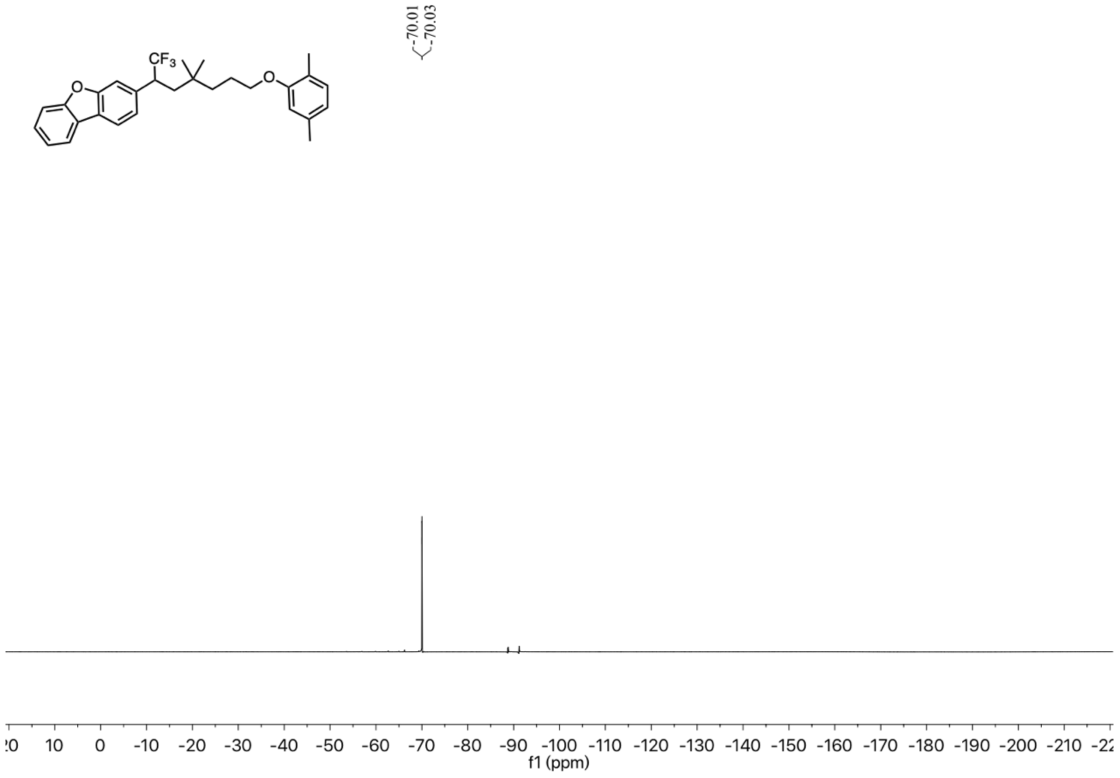

**$^{1}$H NMR (500 MHz, CDCl$_3$)** spectrum of **5-(7-(2,5-dimethylphenoxy)-1,1,1-trifluoro-4,4-dimethylheptan-2-yl)-1-methyl-1*H*-indole (17)**

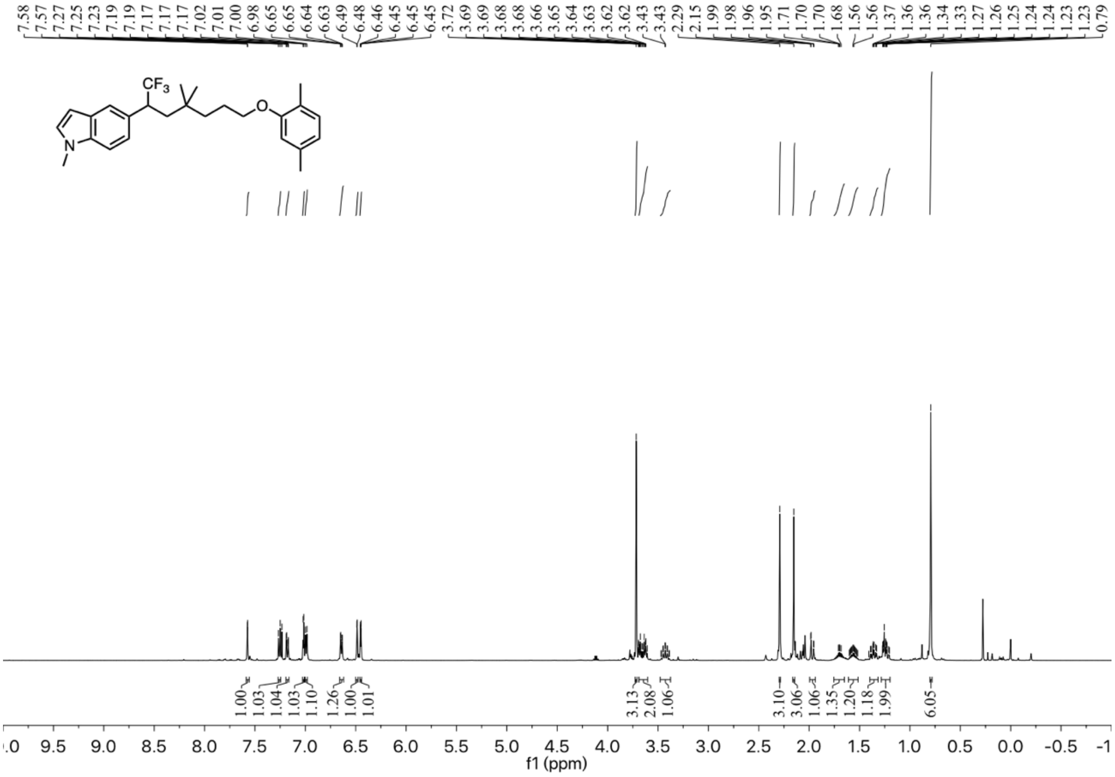

**$^{13}$C NMR (126 MHz, CDCl$_3$)** spectrum of **5-(7-(2,5-dimethylphenoxy)-1,1,1-trifluoro-4,4-dimethylheptan-2-yl)-1-methyl-1*H*-indole (17)**

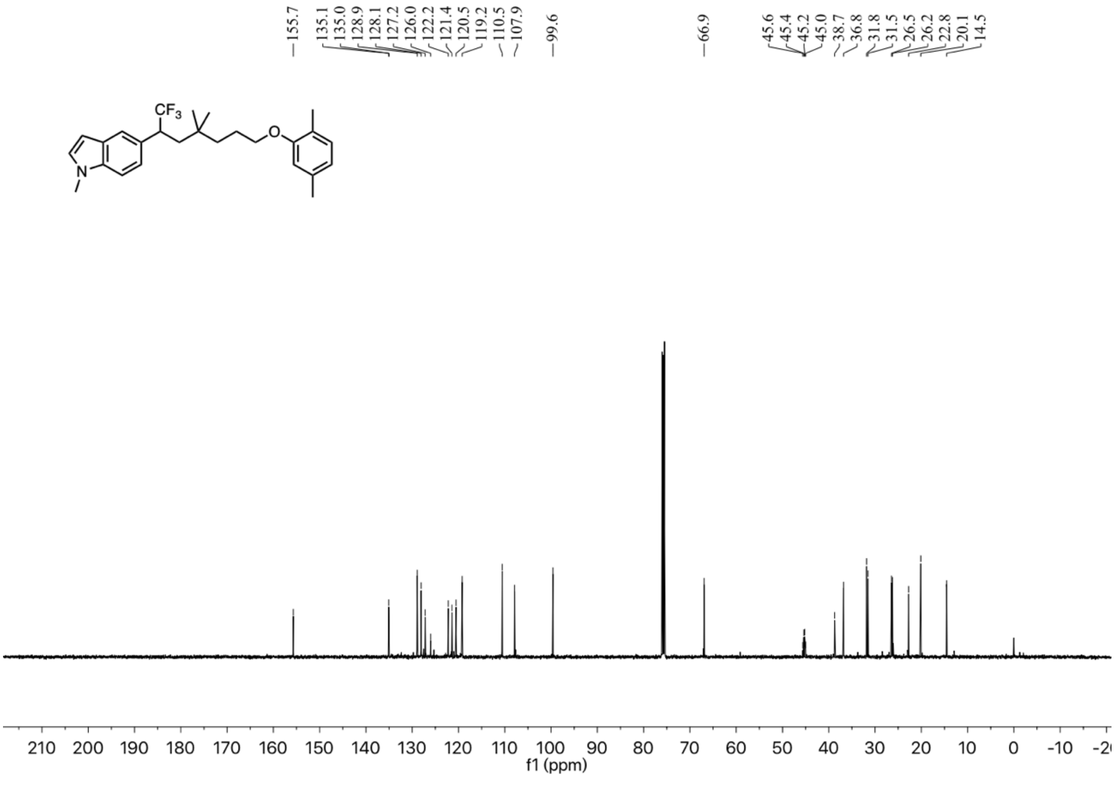

**$^{19}$F NMR (471 MHz, CDCl$_3$)** spectrum of **5-(7-(2,5-dimethylphenoxy)-1,1,1-trifluoro-4,4-dimethylheptan-2-yl)-1-methyl-1*H*-indole (17)**

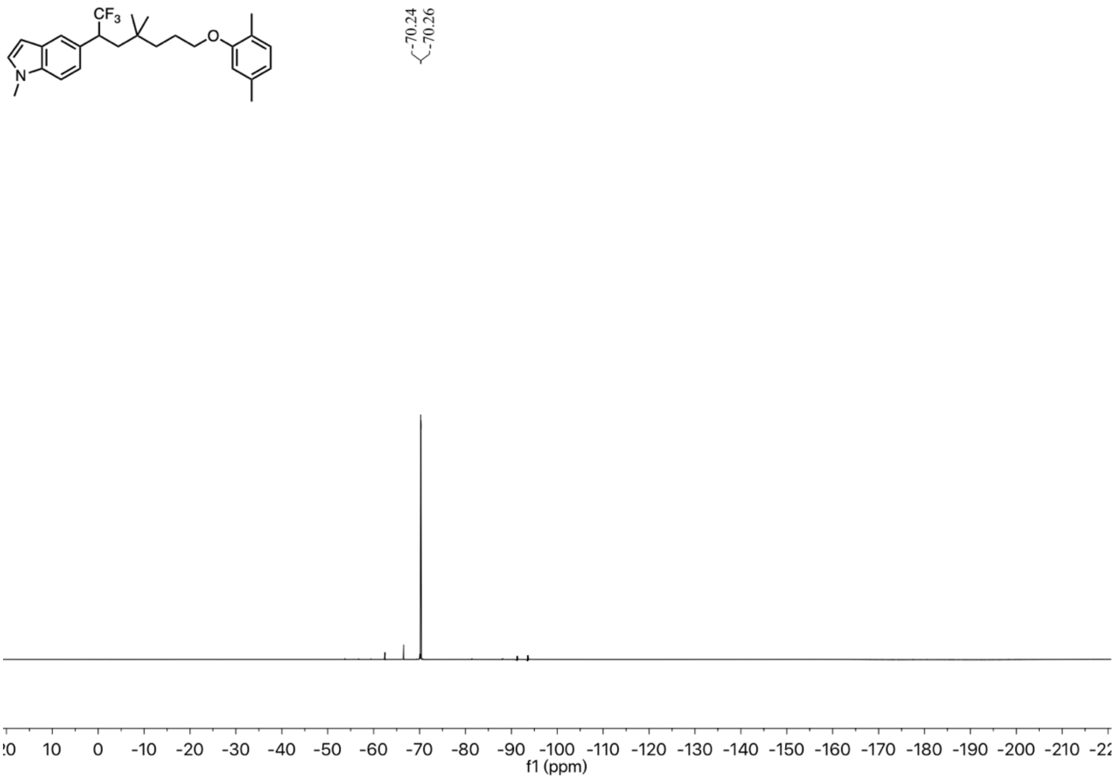


**$^{1}$H NMR (500 MHz, CDCl$_3$)** spectrum of **3-(7-(2,5-dimethylphenoxy)-1,1,1-trifluoro-4,4-dimethylheptan-2-yl)quinoline (18)**

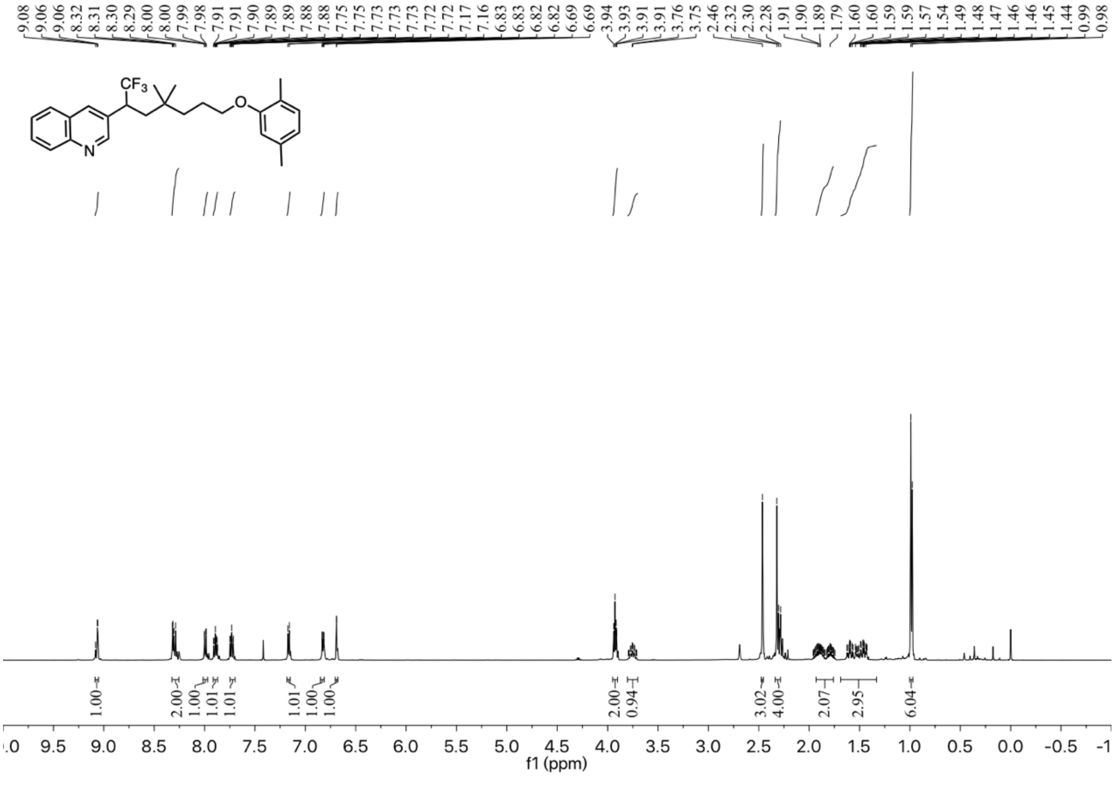

**$^{13}C$ NMR (126 MHz, $CDCl_3$)** spectrum of **3-(7-(2,5-dimethylphenoxy)-1,1,1-trifluoro-4,4-dimethylheptan-2-yl)quinoline (18)**

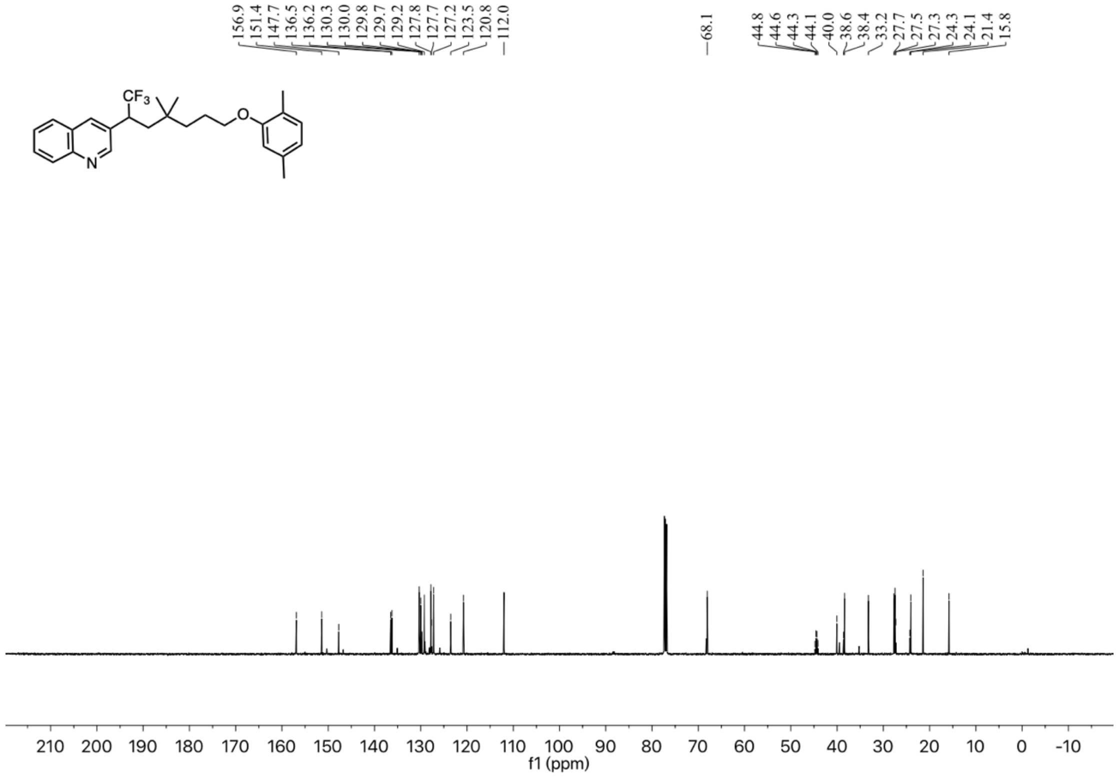


**$^{19}F$ NMR (471 MHz, $CDCl_3$)** spectrum of **3-(7-(2,5-dimethylphenoxy)-1,1,1-trifluoro-4,4-dimethylheptan-2-yl)quinoline (18)**

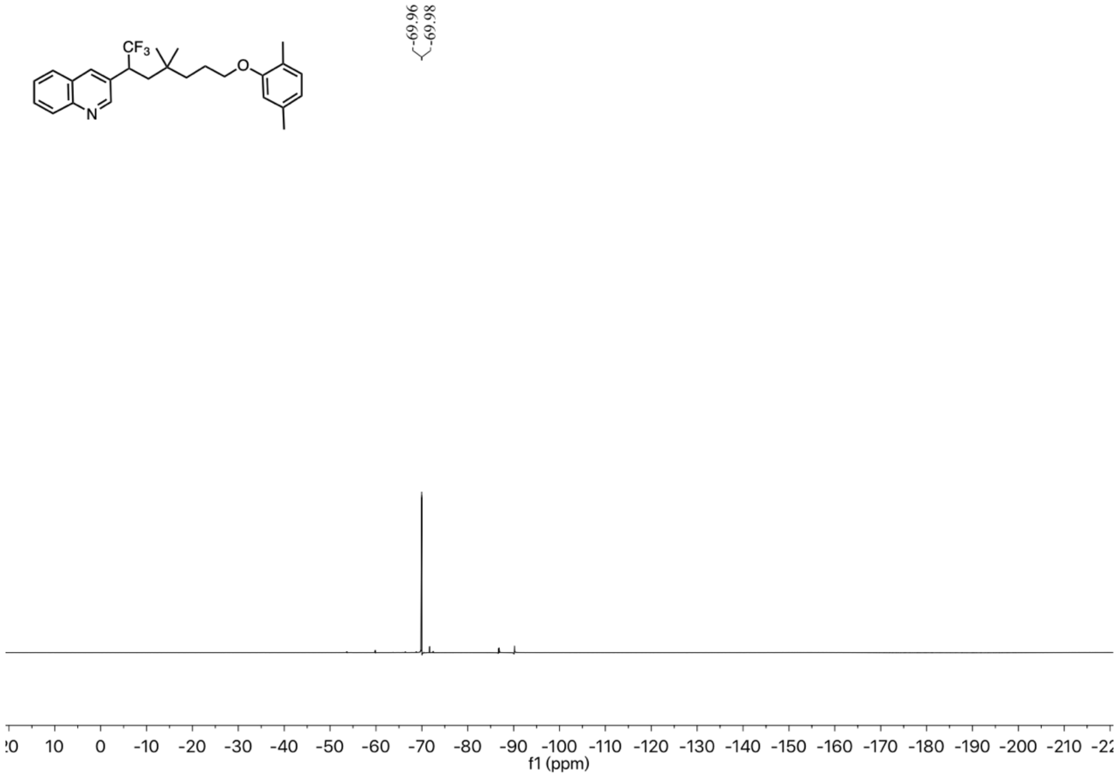

**$^{1}$H NMR (400 MHz, $CDCl_3$)** spectrum of **3-(7-(2,5-dimethylphenoxy)-1,1,1-trifluoro-4,4-dimethylheptan-2-yl)benzamide (19)**

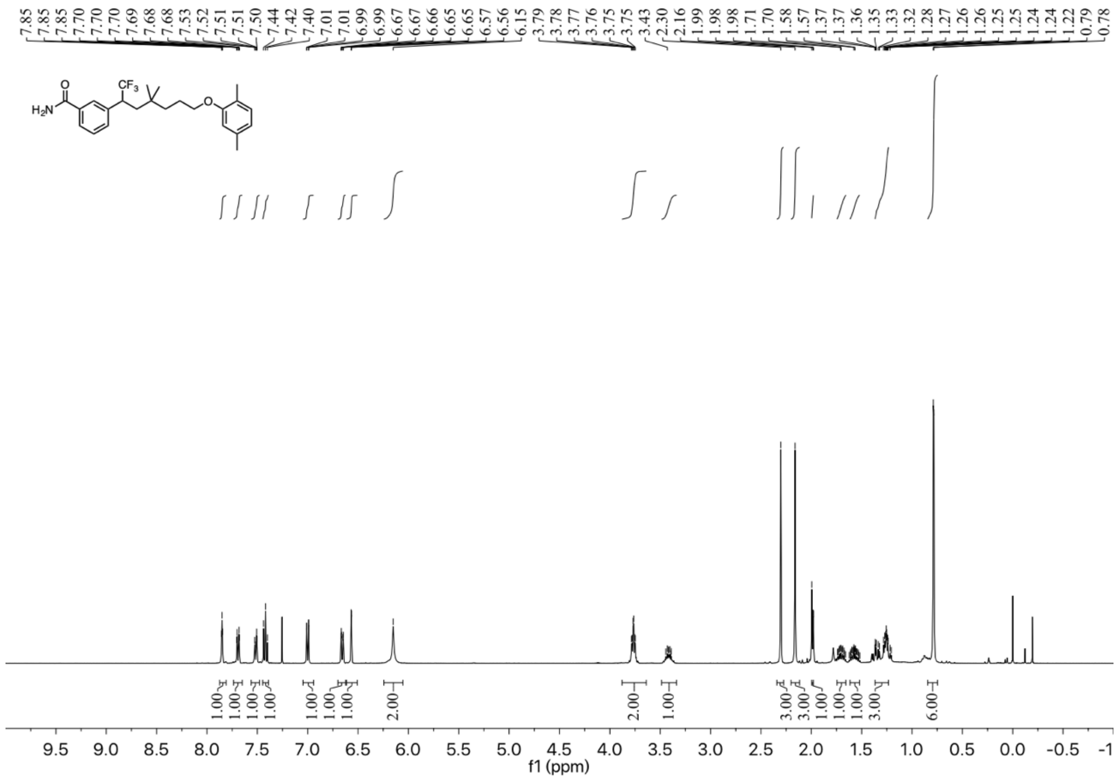

**$^{13}$C NMR (101 MHz, $CDCl_3$)** spectrum of **3-(7-(2,5-dimethylphenoxy)-1,1,1-trifluoro-4,4-dimethylheptan-2-yl)benzamide (19)**

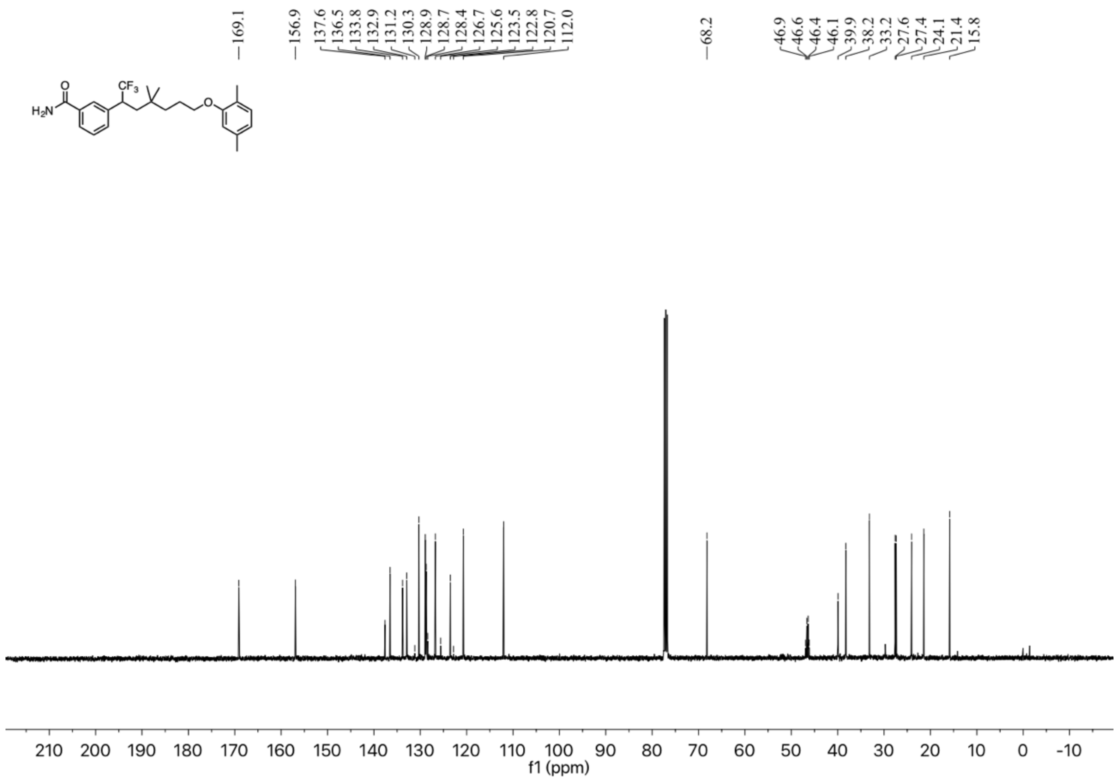

**$^{19}$F NMR (376 MHz, CDCl$_3$)** spectrum of **3-(7-(2,5-dimethylphenoxy)-1,1,1-trifluoro-4,4-dimethylheptan-2-yl)benzamide (19)**

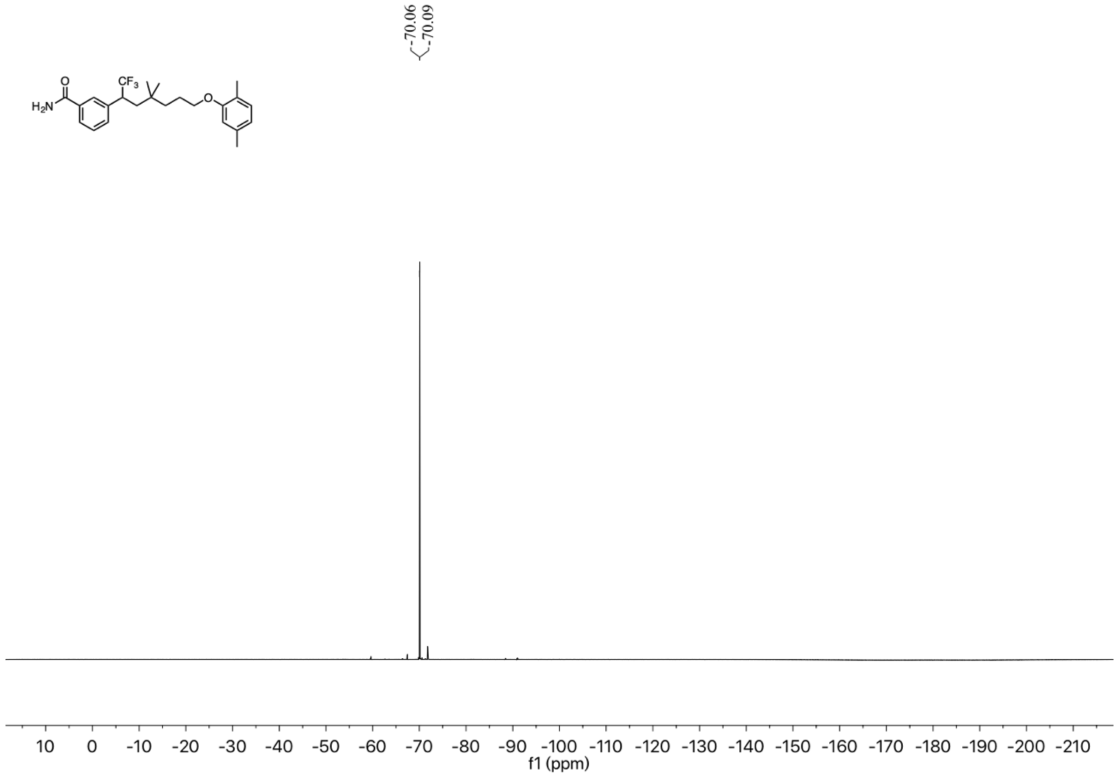


**$^{1}$H NMR (500 MHz, CDCl$_3$)** spectrum of **4-(7-(2,5-dimethylphenoxy)-1,1,1-trifluoro-4,4-dimethylheptan-2-yl)benzoic acid (20)**

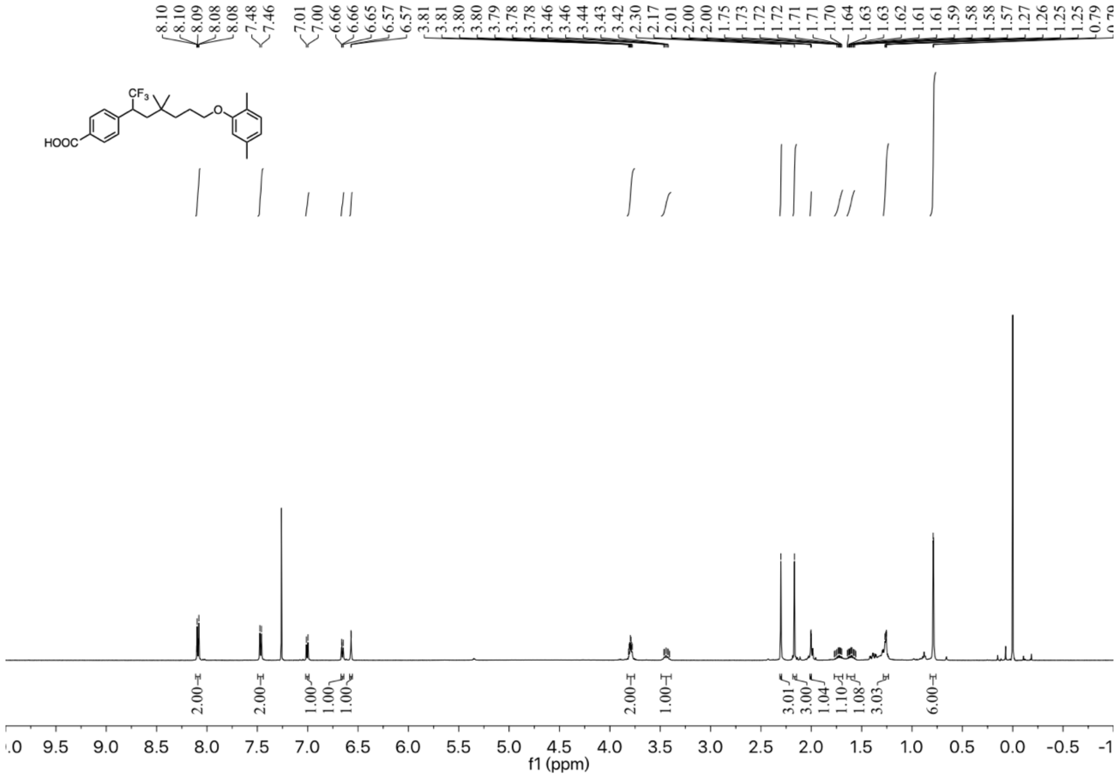

**$^{13}$C NMR (126 MHz, CDCl$_3$)** spectrum of **4-(7-(2,5-dimethylphenoxy)-1,1,1-trifluoro-4,4-dimethylheptan-2-yl)benzoic acid (20)**

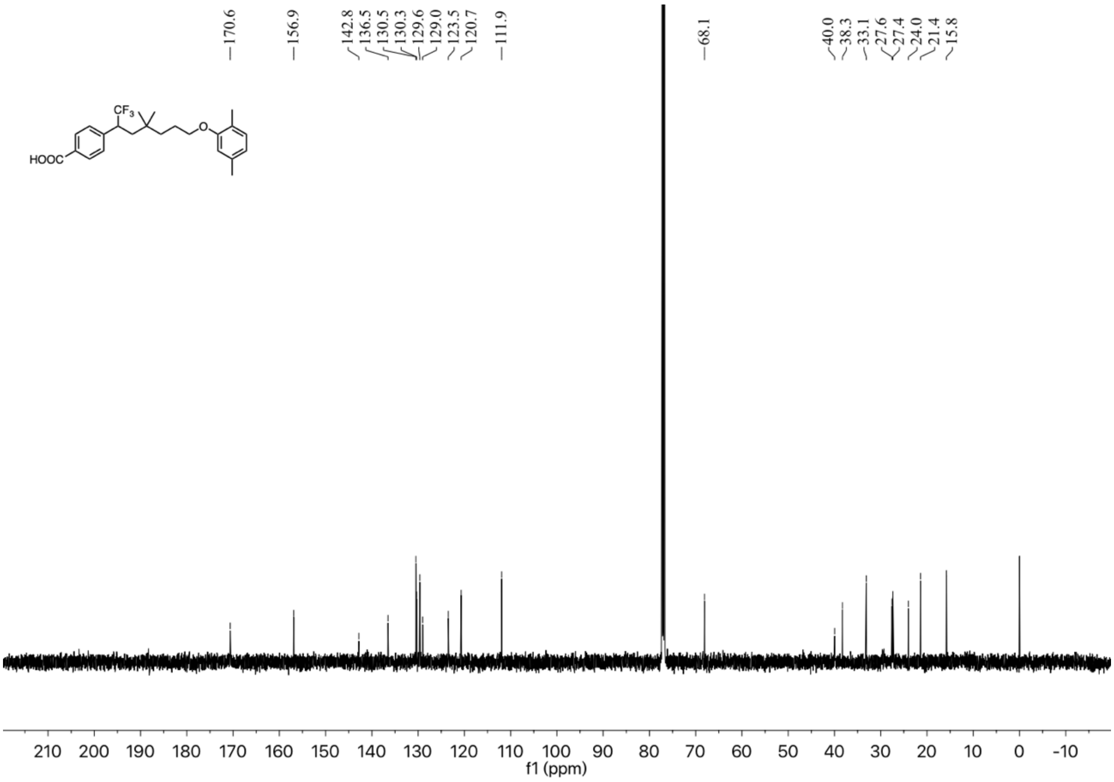


**$^{19}$F NMR (471 MHz, CDCl$_3$)** spectrum of **4-(7-(2,5-dimethylphenoxy)-1,1,1-trifluoro-4,4-dimethylheptan-2-yl)benzoic acid (20)**

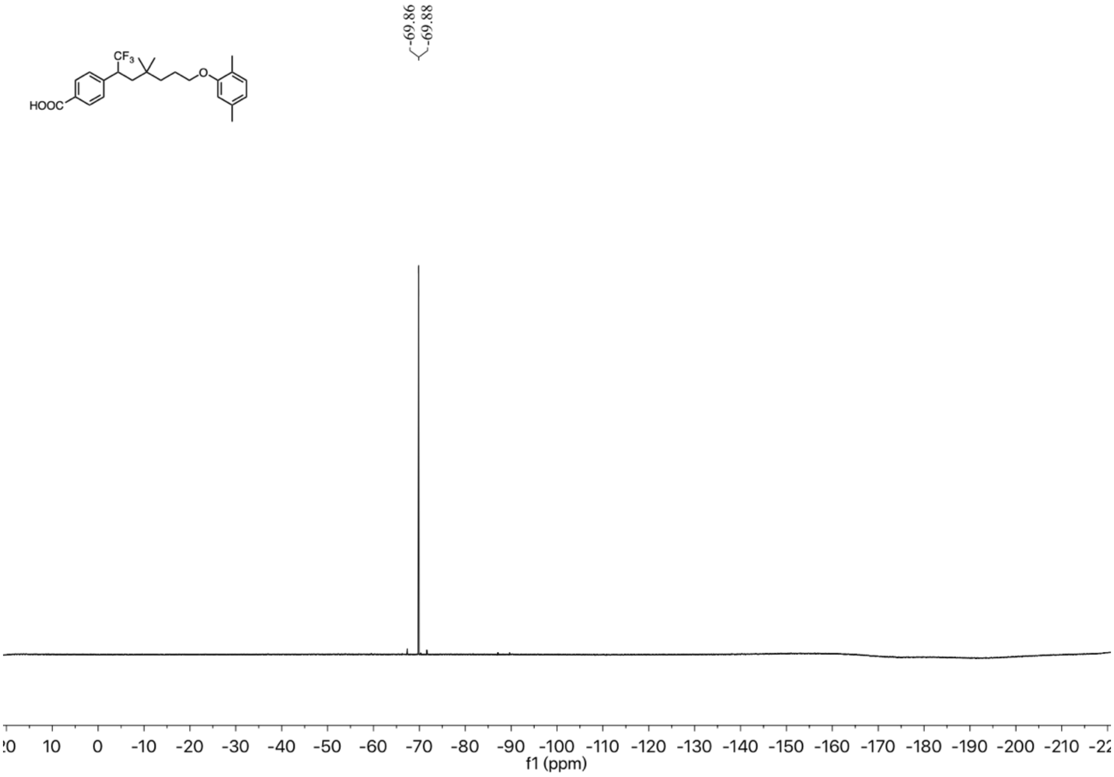

**$^{1}$H NMR (400 MHz, CDCl$_3$)** spectrum of **4-(1,1,1-trifluoro-3-(1-methylcyclohexyl)propan-2-yl)-1,1'-biphenyl (21)**

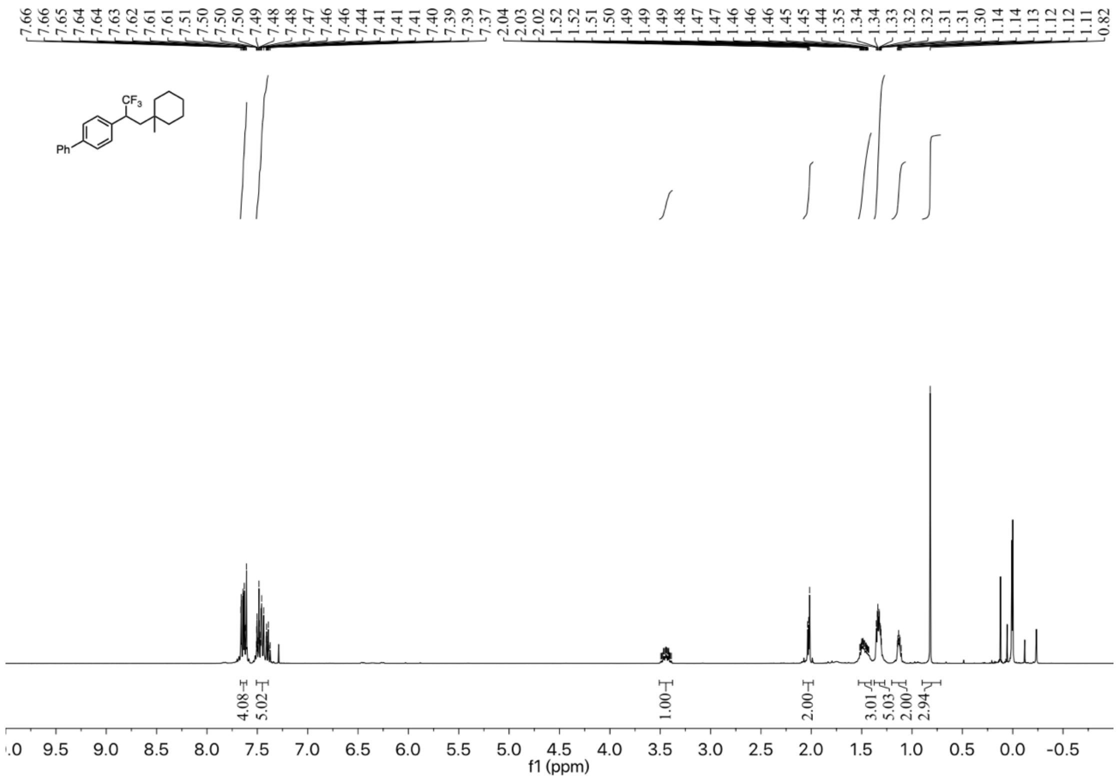

**$^{13}$C NMR (101 MHz, CDCl$_3$)** spectrum of **4-(1,1,1-trifluoro-3-(1-methylcyclohexyl)propan-2-yl)-1,1'-biphenyl (21)**

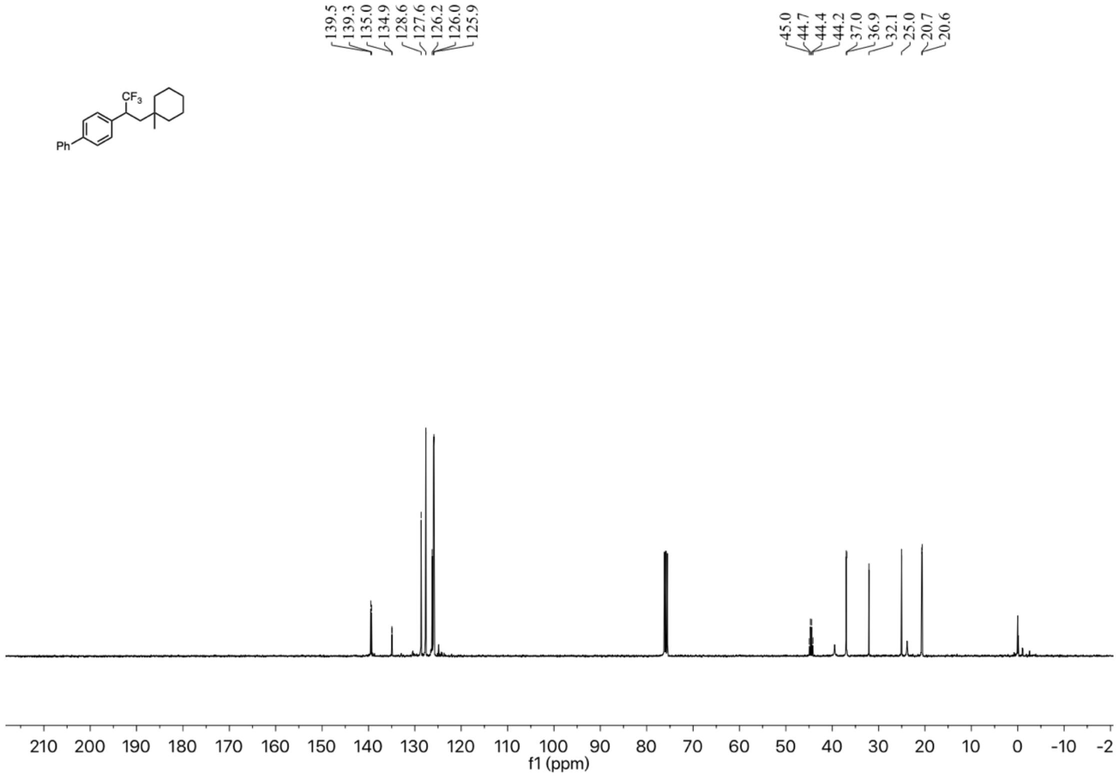

**$^{19}$F NMR (376 MHz, $CDCl_3$)** spectrum of **4-(1,1,1-trifluoro-3-(1-methylcyclohexyl)propan-2-yl)-1,1'-biphenyl (21)**

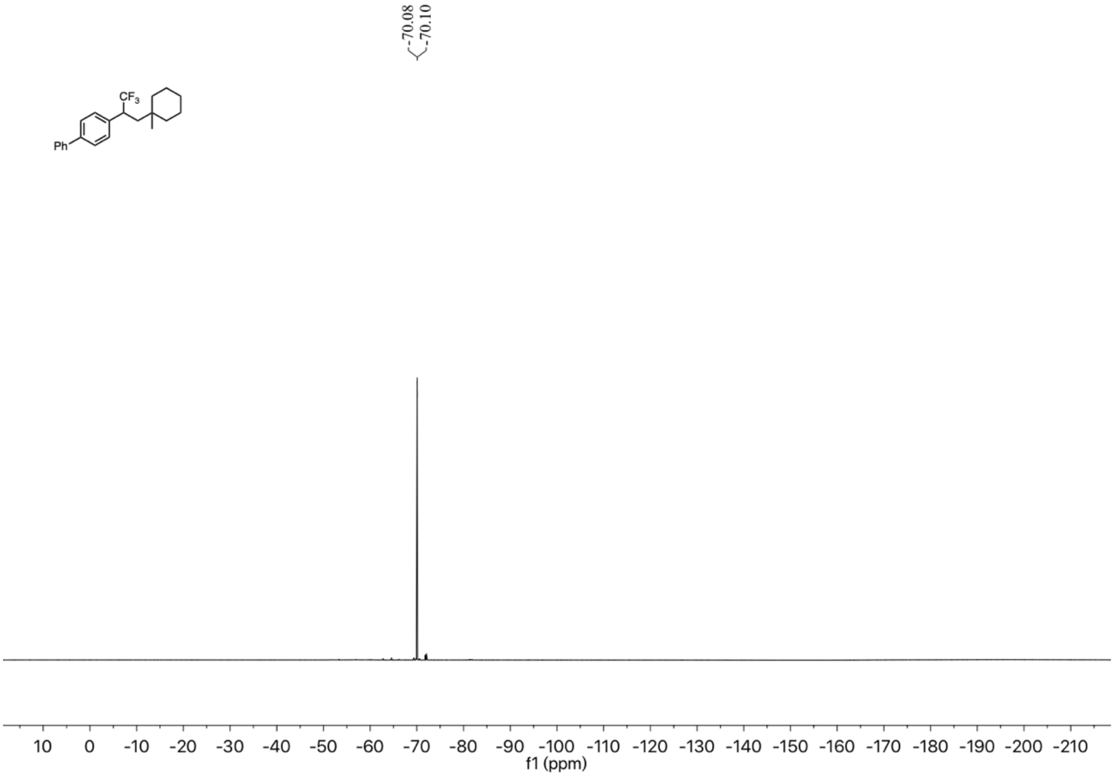

**$^{1}$H NMR (400 MHz, $CDCl_3$)** spectrum of **(3*r*,5*r*,7*r*)-1-(2-([1,1'-biphenyl]-4-yl)-3,3,3-trifluoropropyl)adamantane (22)**

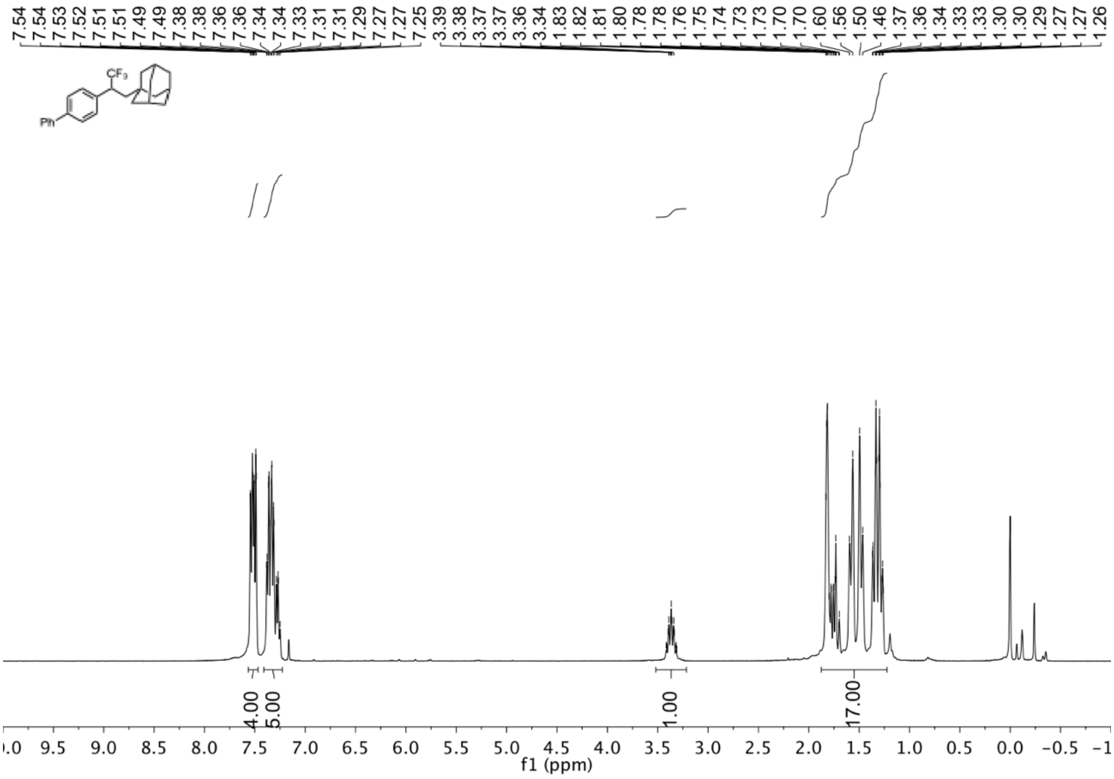

**$^{13}$C NMR (101 MHz, CDCl$_3$)** spectrum of **(3*r*,5*r*,7*r*)-1-(2-([1,1'-biphenyl]-4-yl)-3,3,3-trifluoropropyl)adamantane (22)**

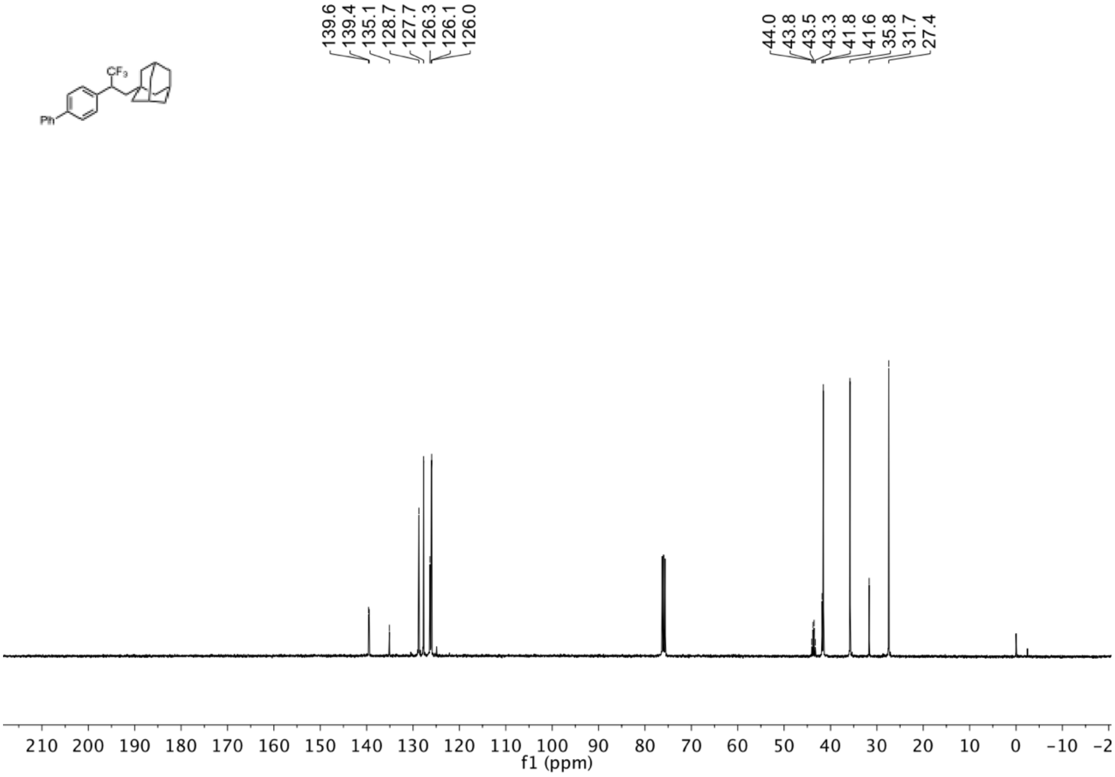


**$^{19}$F NMR (376 MHz, CDCl$_3$)** spectrum of **(3*r*,5*r*,7*r*)-1-(2-([1,1'-biphenyl]-4-yl)-3,3,3-trifluoropropyl)adamantane (22)**

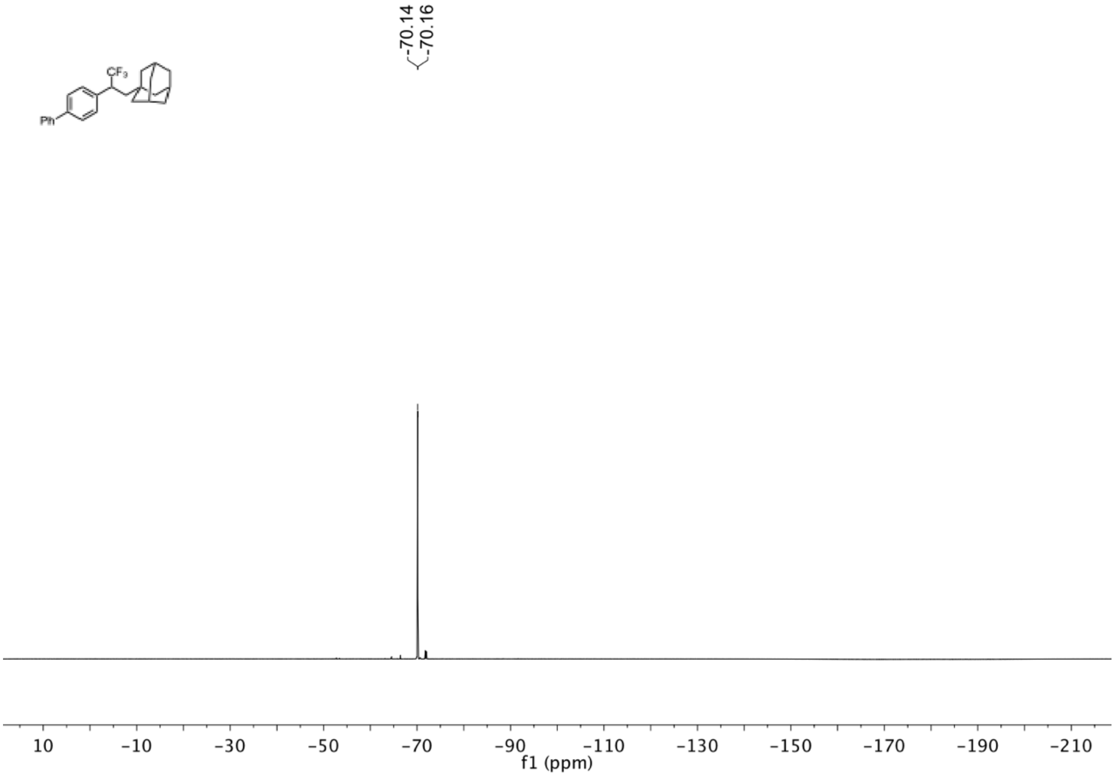

**$^{1}$H NMR (600 MHz, CDCl3)** spectrum of **4-(3-cyclohexyl-1,1,1-trifluoropropan-2-yl)-1,1'-biphenyl (23)**

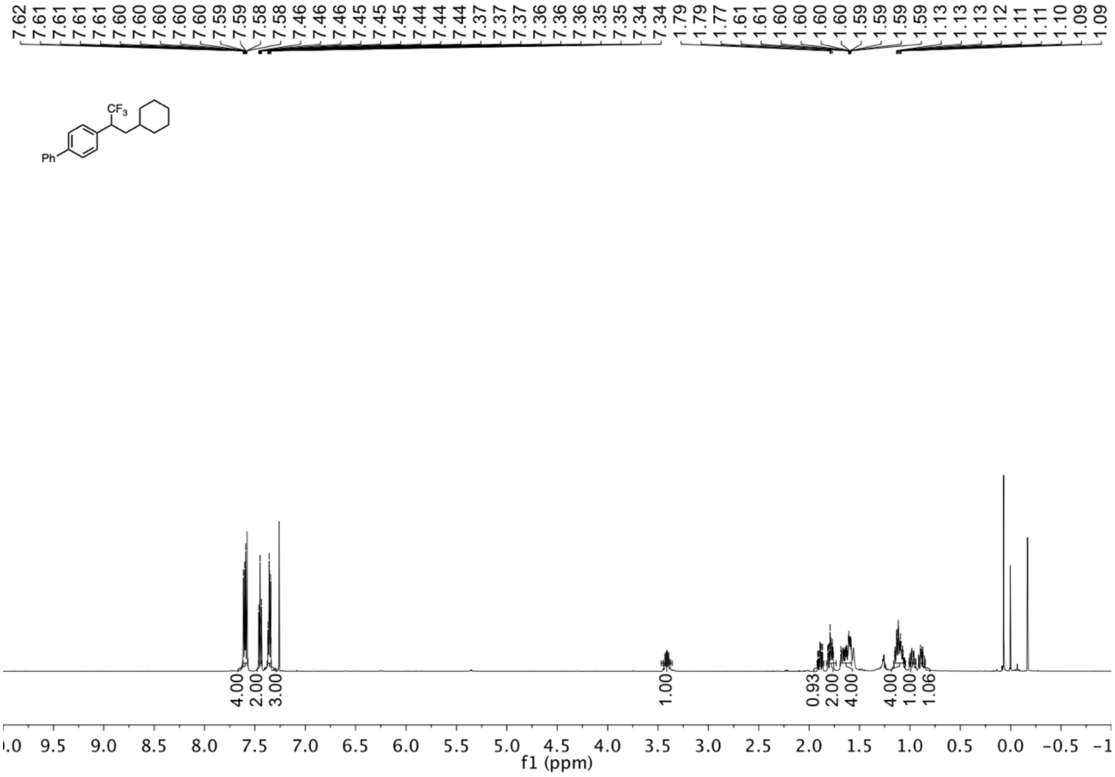

**$^{13}$C NMR (126 MHz, CDCl3)** spectrum of **4-(3-cyclohexyl-1,1,1-trifluoropropan-2-yl)-1,1'-biphenyl (23)**

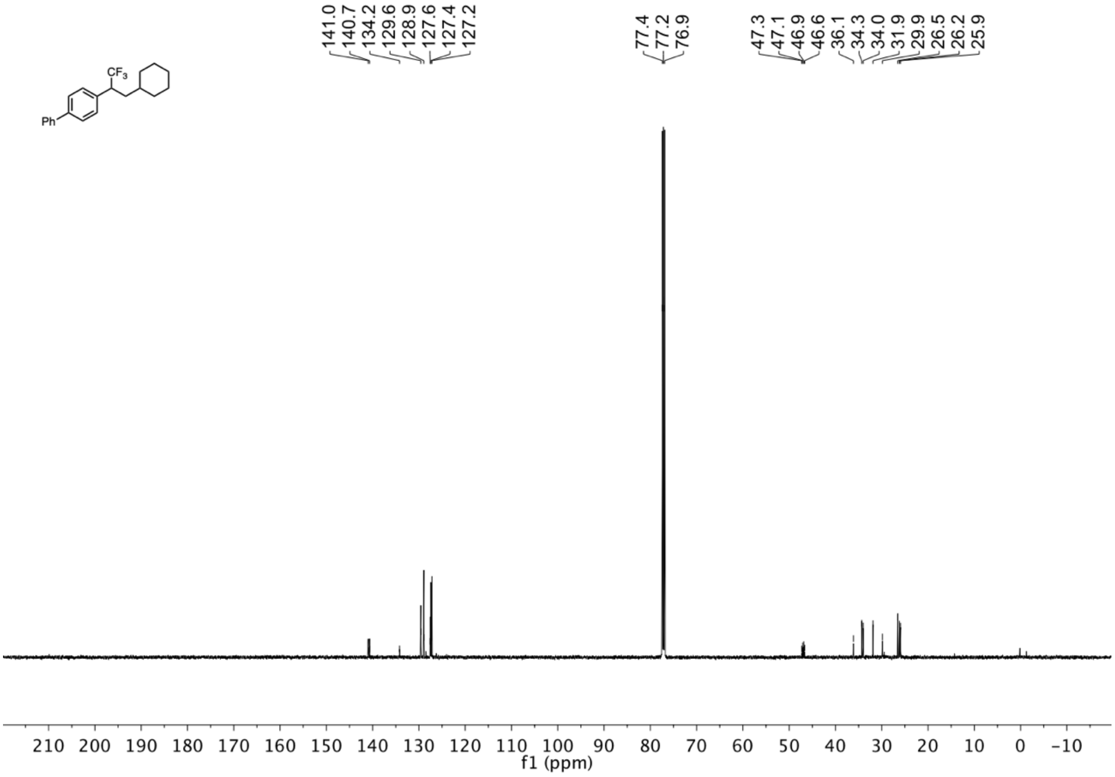

**$^{19}$F NMR (565 MHz, CDCl$_3$)** spectrum of **4-(3-cyclohexyl-1,1,1-trifluoropropan-2-yl)-1,1'-biphenyl (23)**

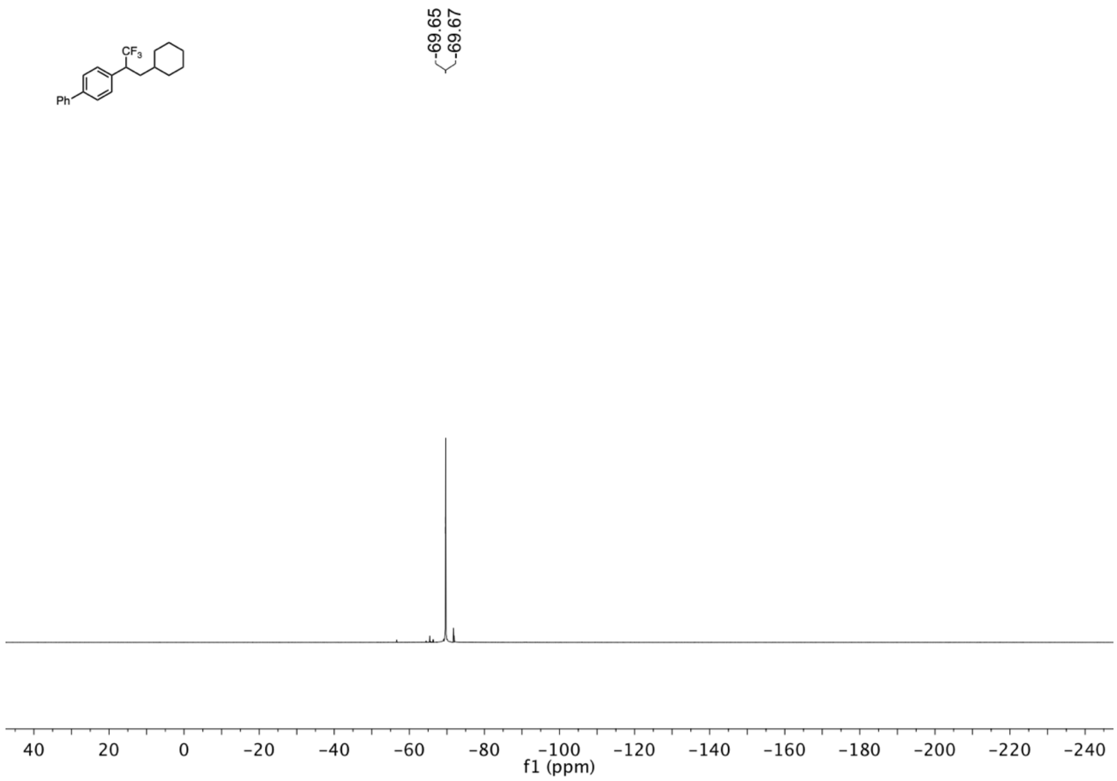

**$^{1}$H NMR (400 MHz, CDCl$_3$)** spectrum of **4-(1,1,1-trifluoro-5-phenylpentan-2-yl)-1,1'-biphenyl (24)**

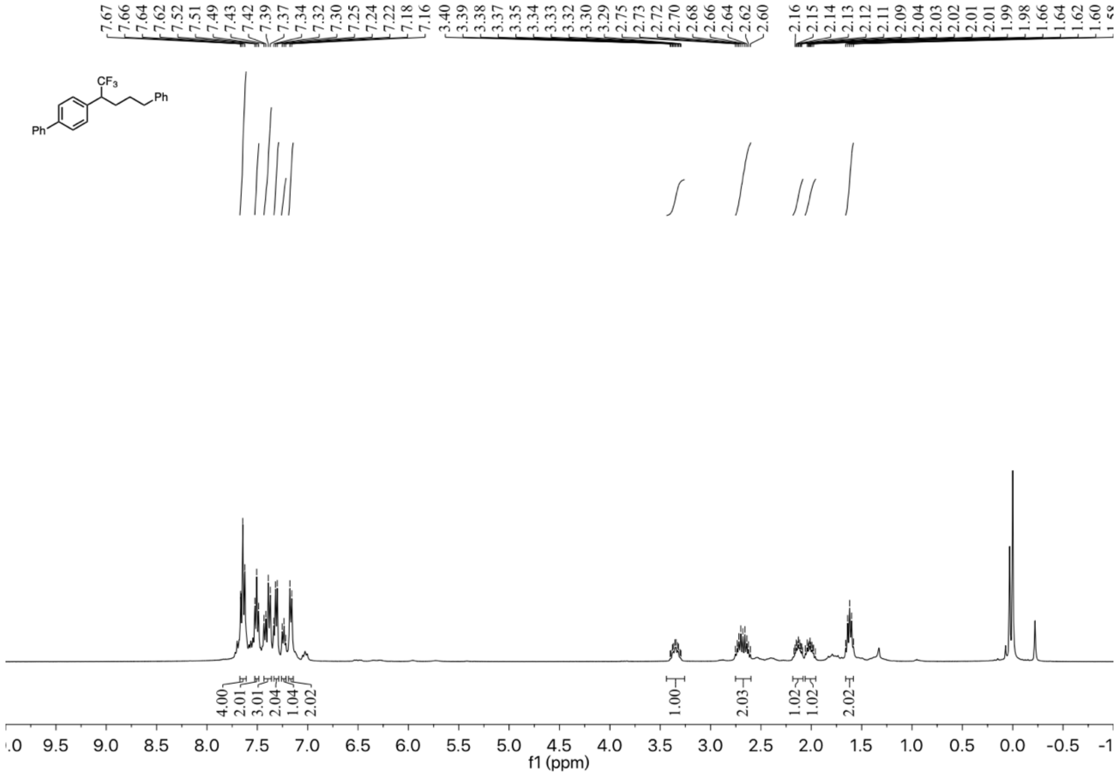

**$^{13}$C NMR (126 MHz, CDCl$_3$)** spectrum of **4-(1,1,1-trifluoro-5-phenylpentan-2-yl)-1,1'-biphenyl (24)**

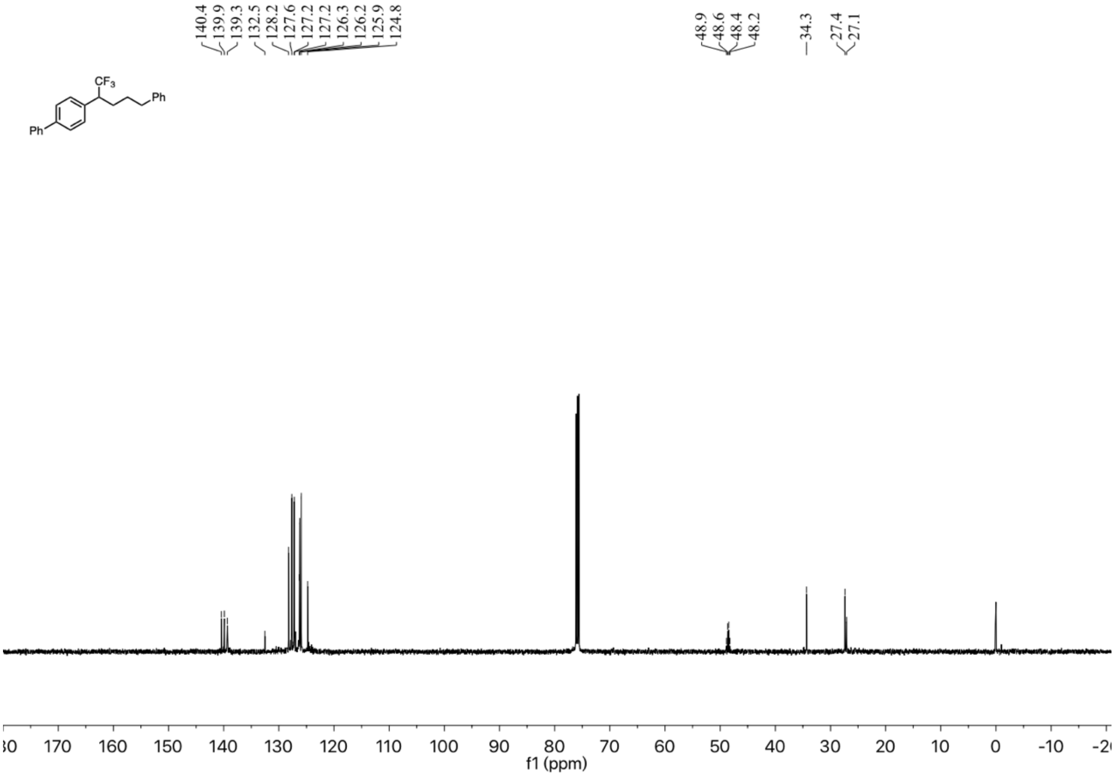


**$^{19}$F NMR (471 MHz, CDCl$_3$)** spectrum of **4-(1,1,1-trifluoro-5-phenylpentan-2-yl)-1,1'-biphenyl (24)**

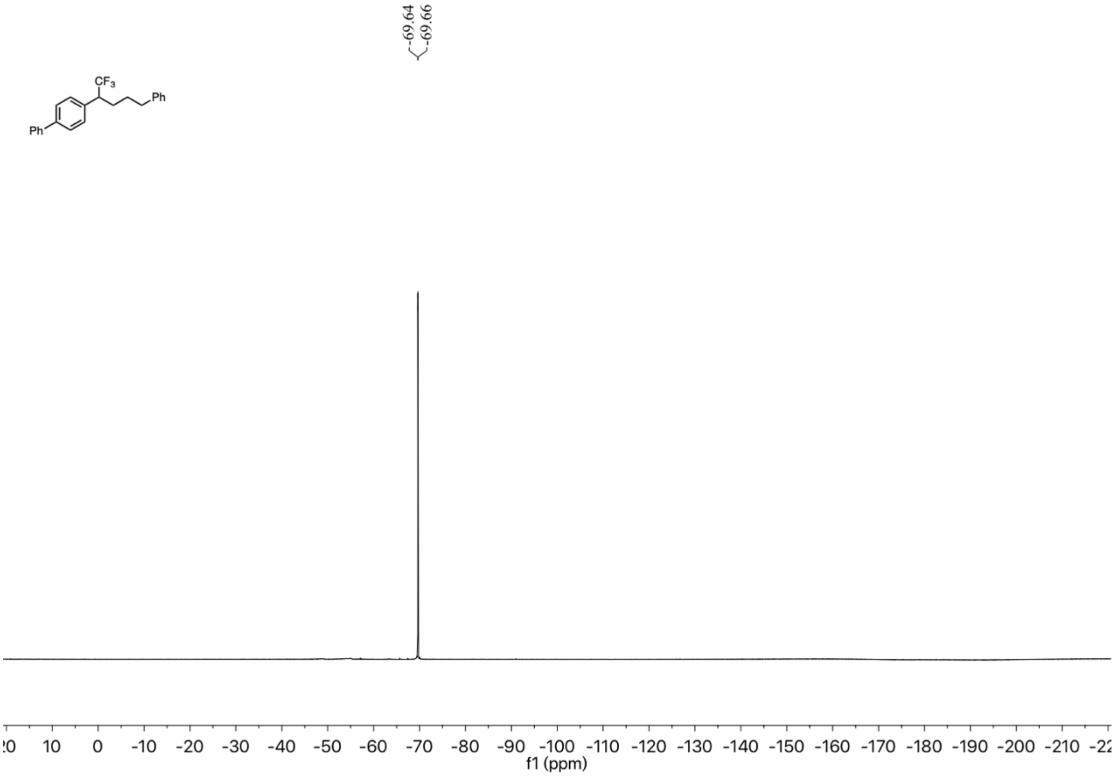

**$^1$H NMR (400 MHz, CDCl$_3$)** spectrum of **4-(1,1,1-trifluoro-4-(*p*-tolyl)butan-2-yl)-1,1'-biphenyl (25)**

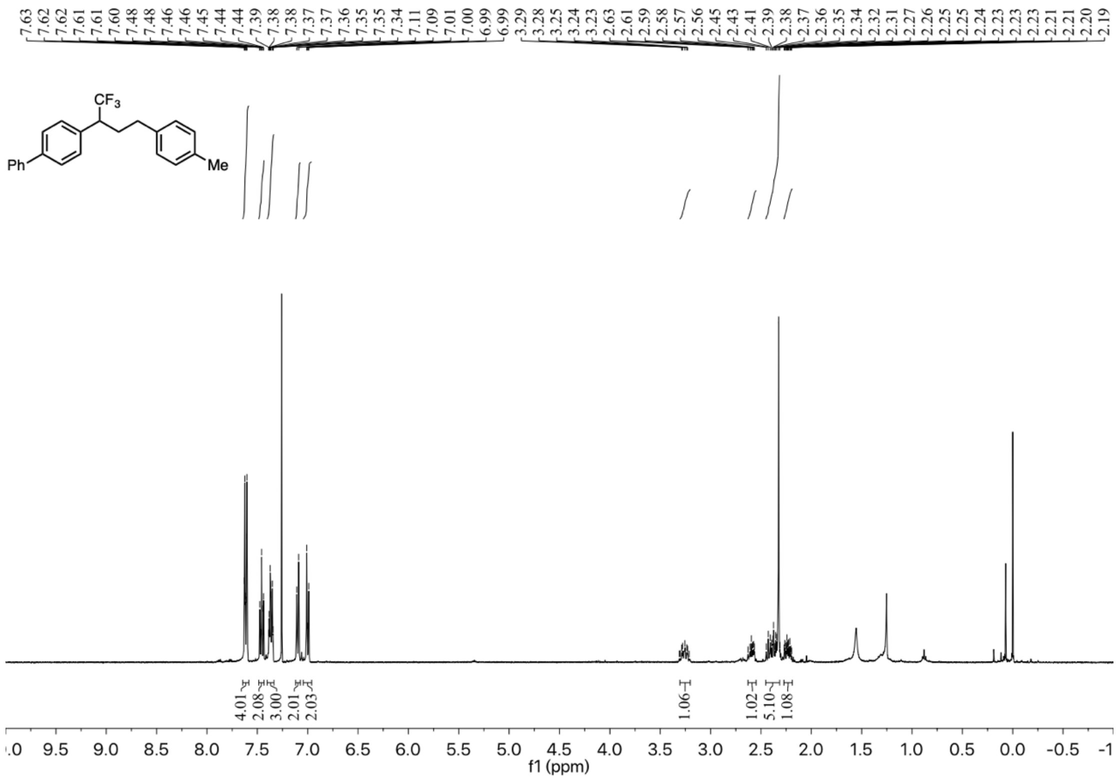

**$^{13}$C NMR (101 MHz, CDCl$_3$)** spectrum of **4-(1,1,1-trifluoro-4-(*p*-tolyl)butan-2-yl)-1,1'-biphenyl (25)**

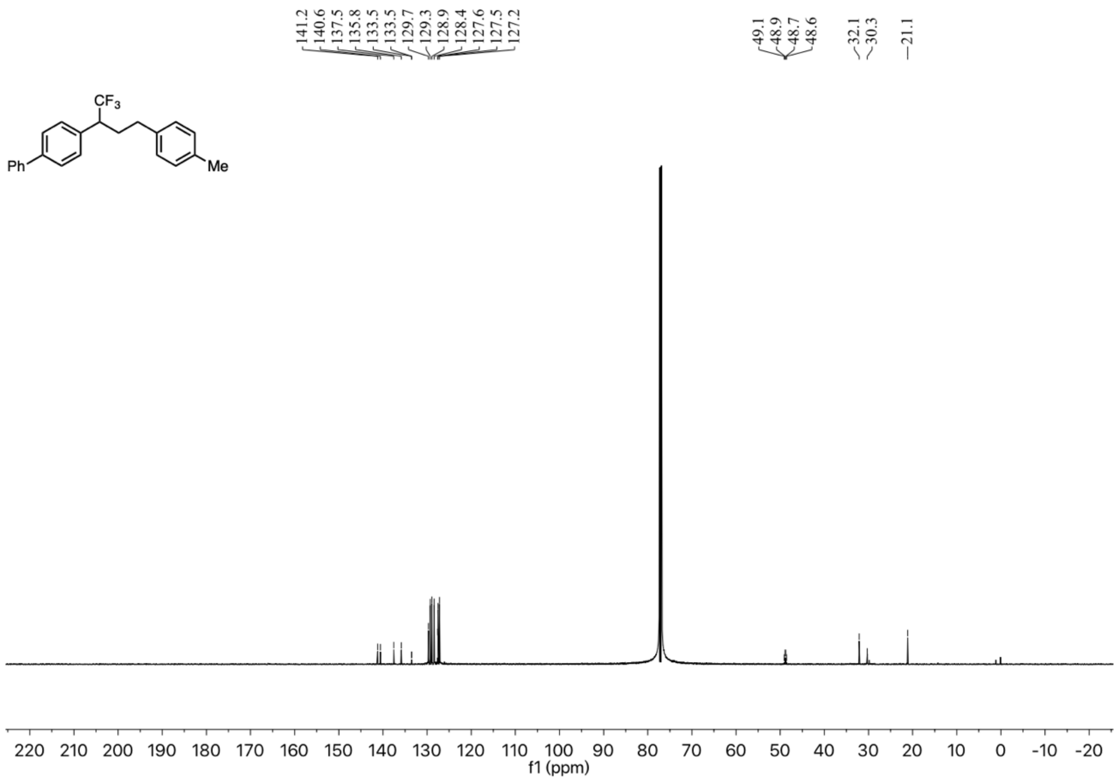

**$^{19}$F NMR (376 MHz, CDCl$_3$)** spectrum of **4-(1,1,1-trifluoro-4-(*p*-tolyl)butan-2-yl)-1,1'-biphenyl (25)**

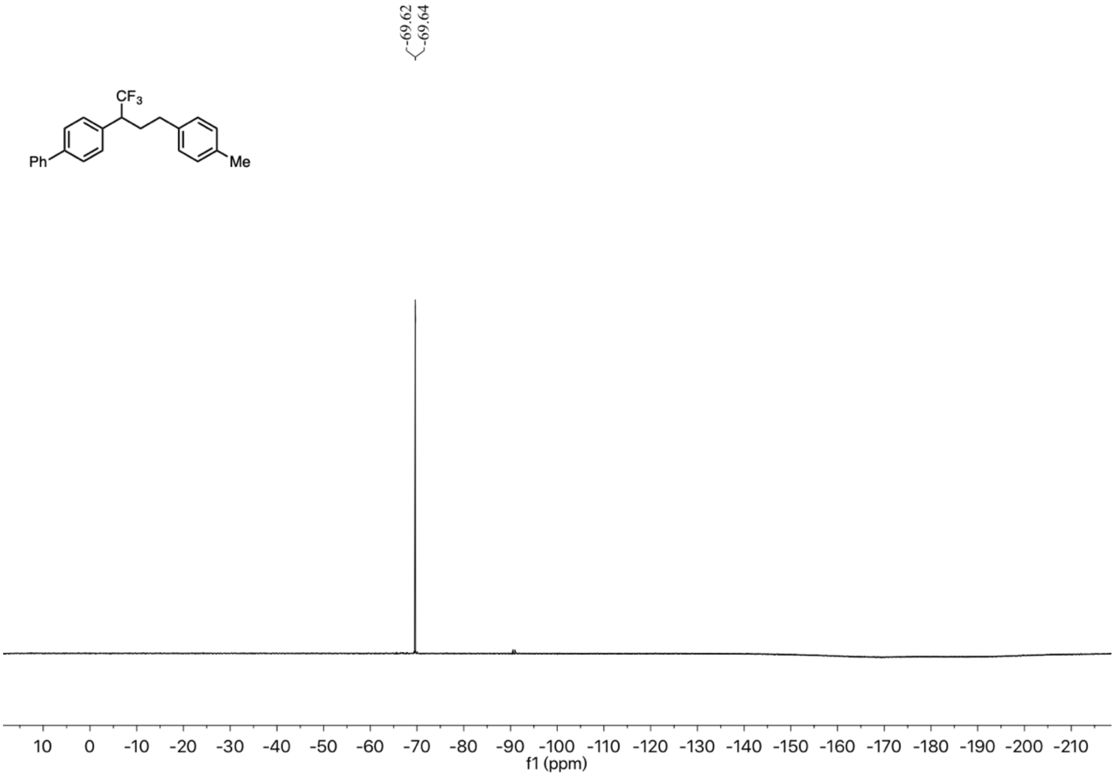


**$^{1}$H NMR (400 MHz, CDCl$_3$)** spectrum of **4-(1,1,1-trifluoro-4-(4-fluorophenyl)butan-2-yl)-1,1'-biphenyl (26)**

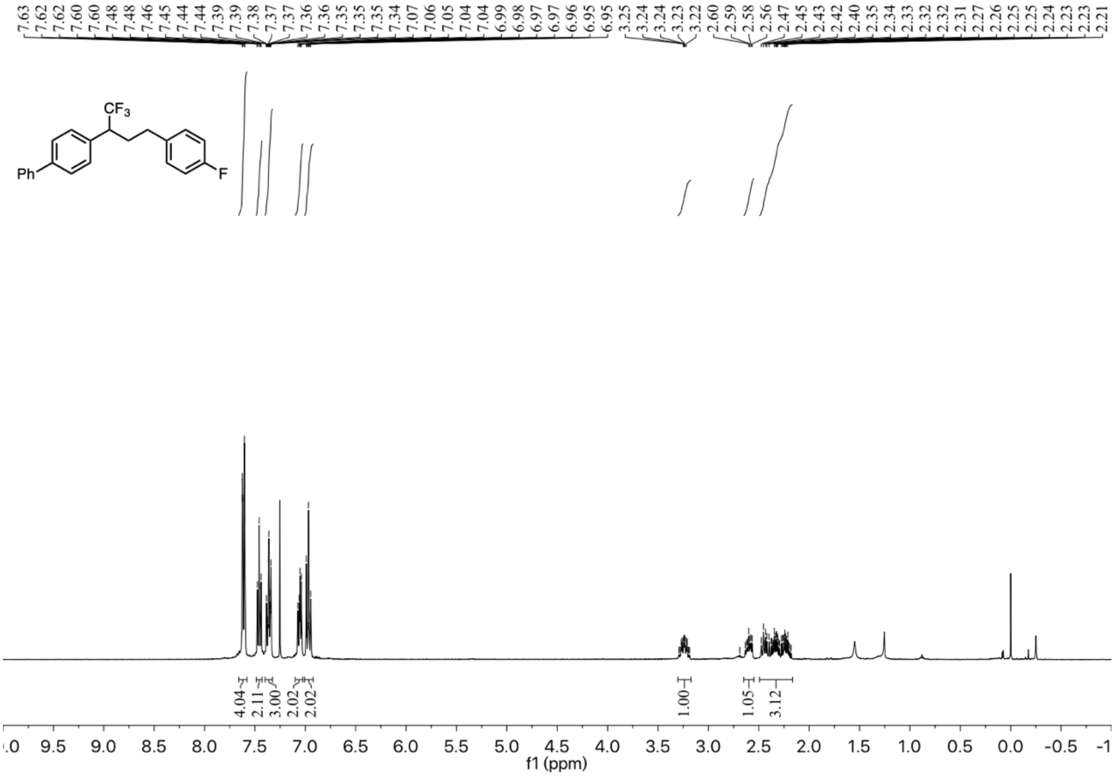

**$^{13}C$ NMR (101 MHz, $CDCl_3$)** spectrum of **4-(1,1,1-trifluoro-4-(4-fluorophenyl)butan-2-yl)-1,1'-biphenyl (26)**

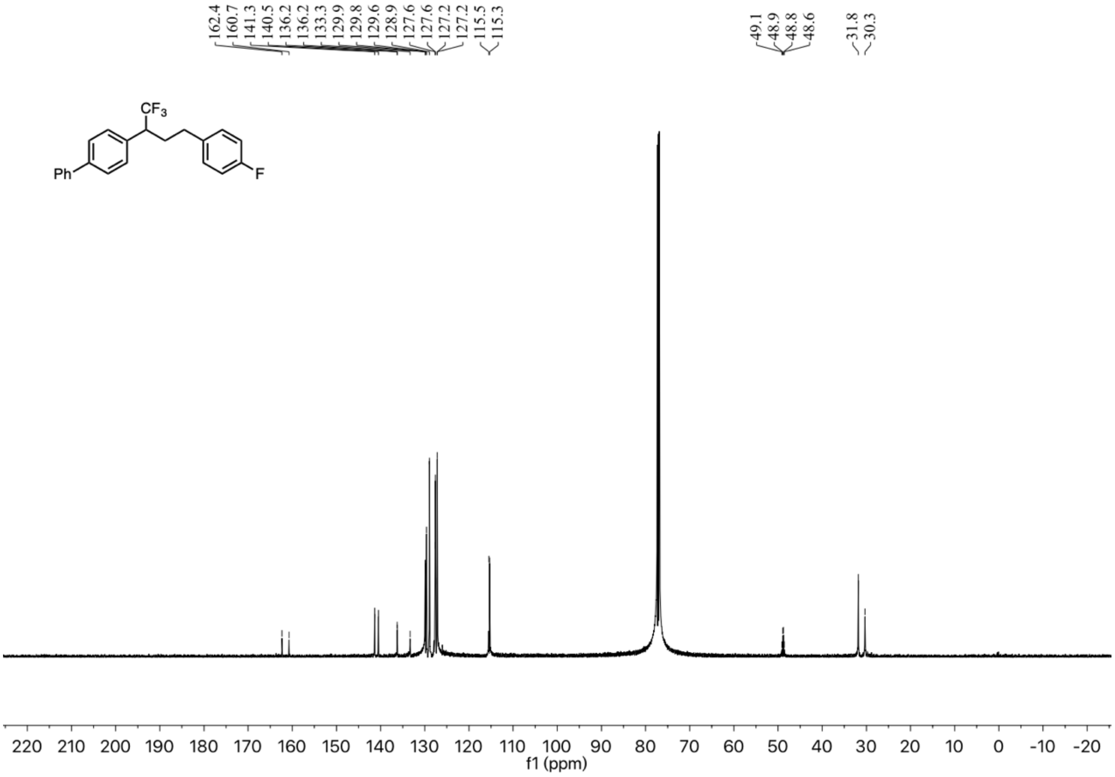


**$^{19}F$ NMR (376 MHz, $CDCl_3$)** spectrum of **4-(1,1,1-trifluoro-4-(4-fluorophenyl)butan-2-yl)-1,1'-biphenyl (26)**

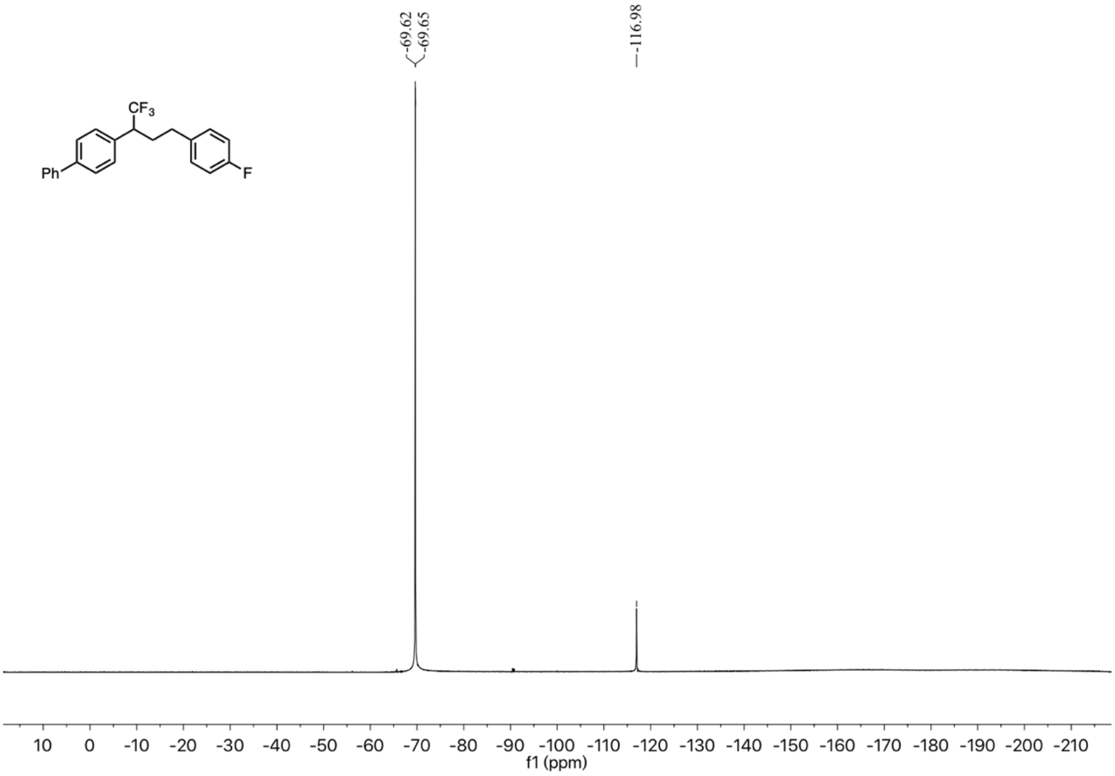

**$^{1}$H NMR (400 MHz, CDCl3)** spectrum of **(*Z*)-4-(1,1,1-trifluorotricos-15-en-2-yl)-1,1'-biphenyl (27)**

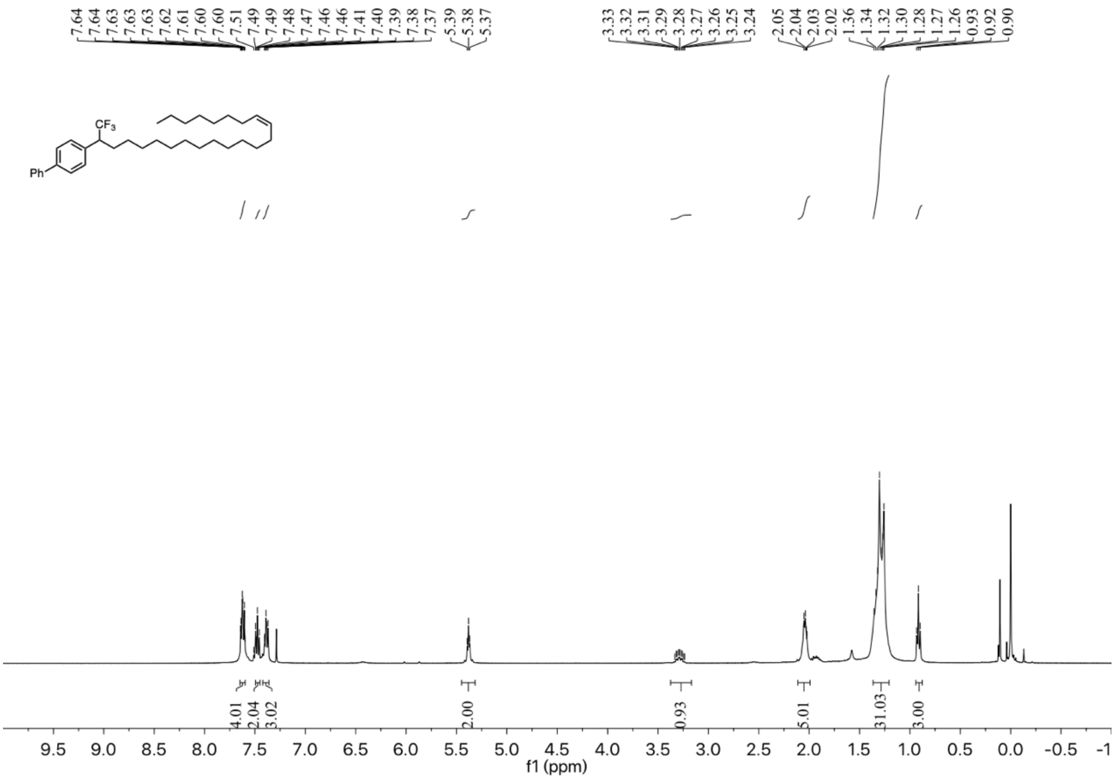

**$^{13}$C NMR (126 MHz, CDCl3)** spectrum of **(*Z*)-4-(1,1,1-trifluorotricos-15-en-2-yl)-1,1'-biphenyl (27)**

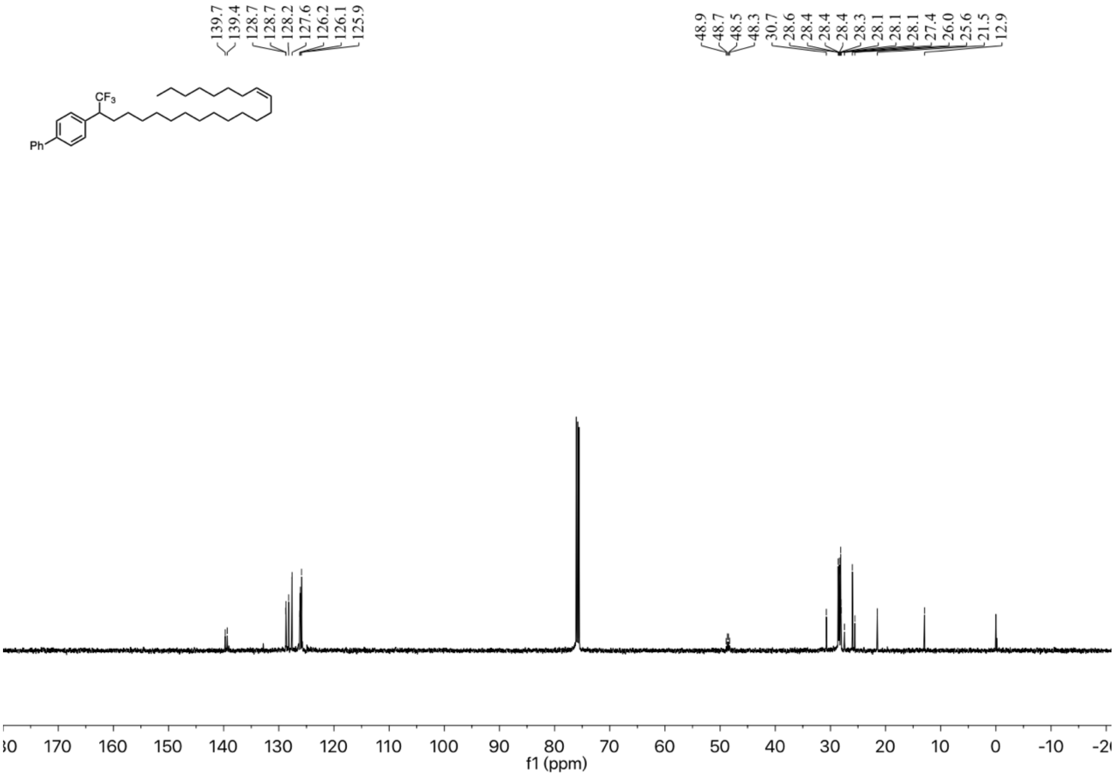

**$^{19}$F NMR (376 MHz, $CDCl_3$)** spectrum of**(*Z*)-4-(1,1,1-trifluorotricos-15-en-2-yl)-1,1'-biphenyl (27)**

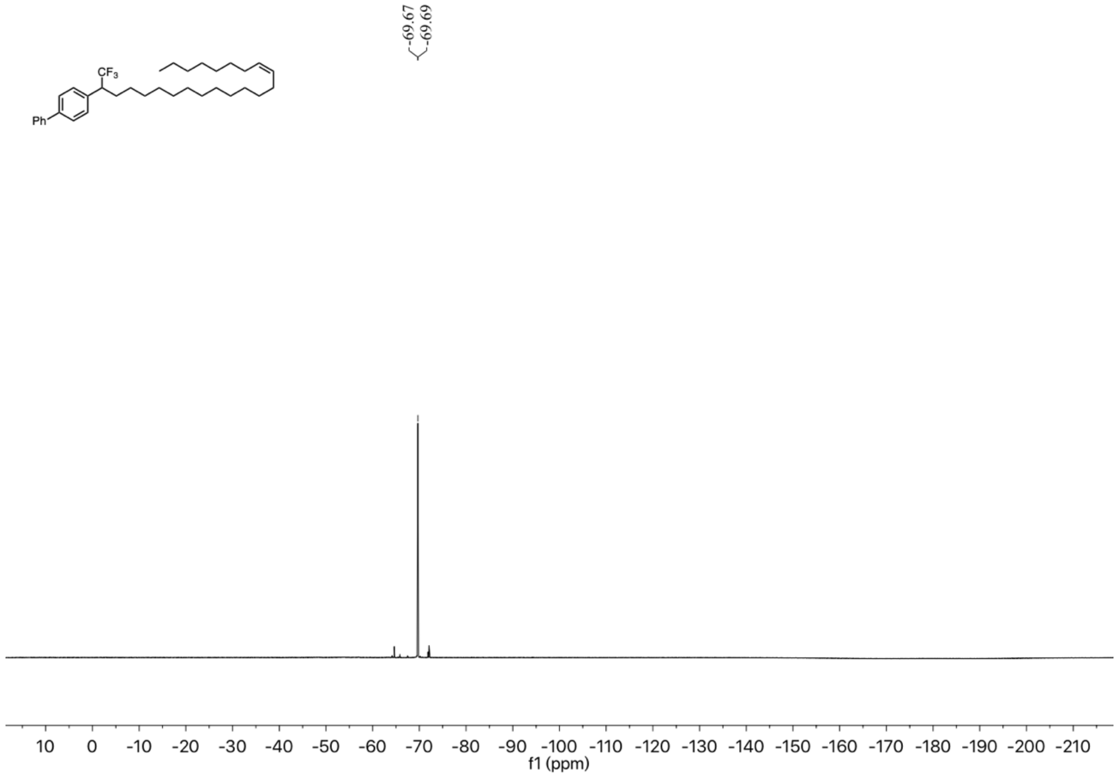

**$^{1}$H NMR (500 MHz, $CDCl_3$)** spectrum of **4-(1,1,1-trifluoro-4,4-dimethylpentan-2-yl)phenyl (2*R*)-2-(6-methoxynaphthalen-2-yl)propanoate (28)**

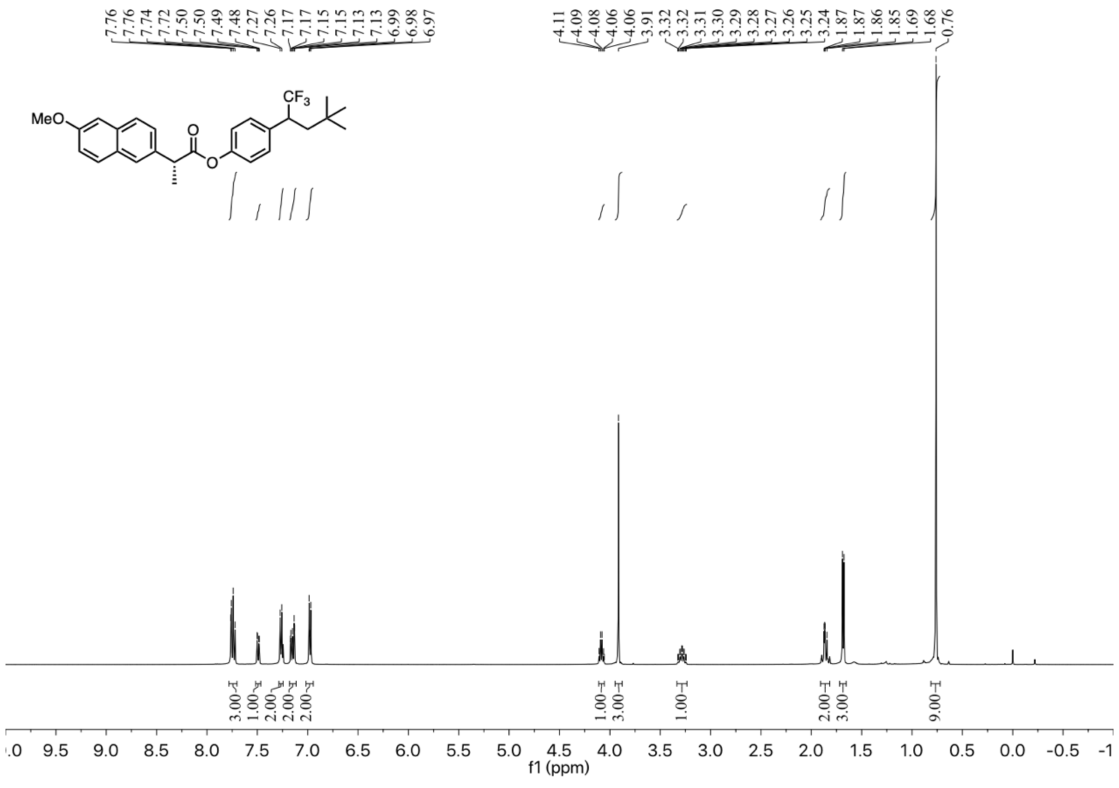

**$^{13}$C NMR (101 MHz, CDCl$_3$)** spectrum of **4-(1,1,1-trifluoro-4,4-dimethylpentan-2-yl)phenyl (2*R*)-2-(6-methoxynaphthalen-2-yl)propanoate (28)**

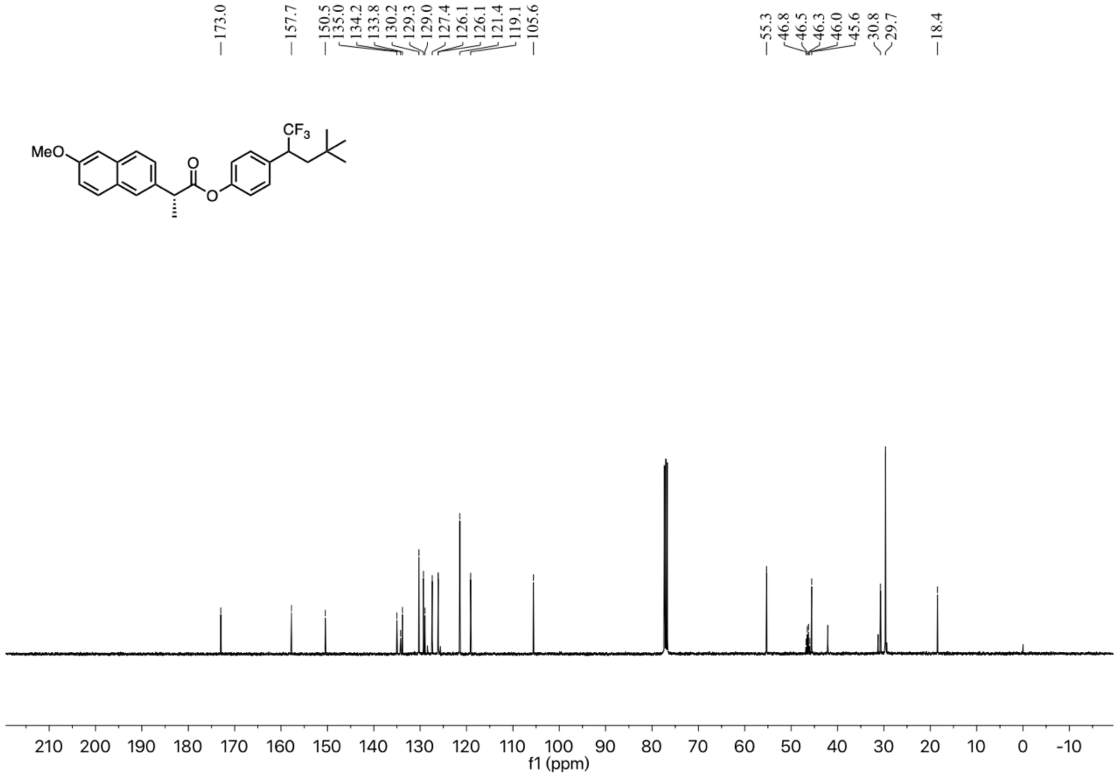


**$^{19}$F NMR (471 MHz, CDCl$_3$)** spectrum of **4-(1,1,1-trifluoro-4,4-dimethylpentan-2-yl)phenyl (2*R*)-2-(6-methoxynaphthalen-2-yl)propanoate (28)**

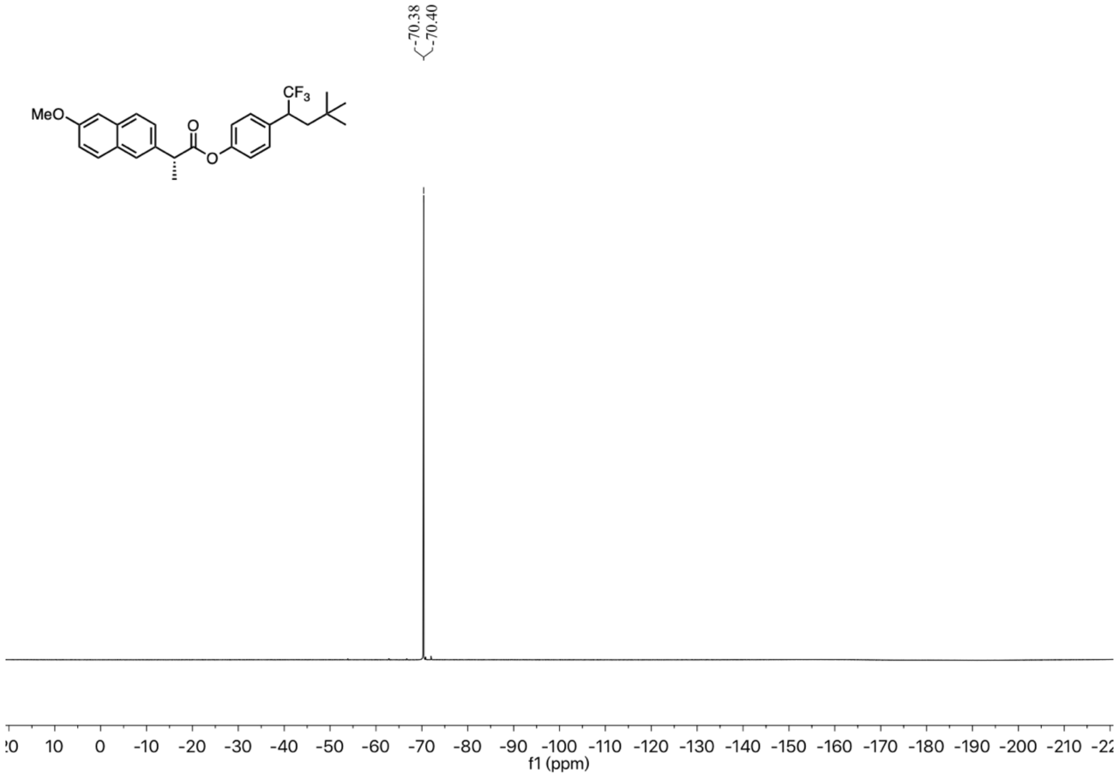

**$^{1}$H NMR (600 MHz, $CDCl_3$)** spectrum of **2-(3-([1,1'-biphenyl]-4-yl)-4,4,4-trifluorobutyl)dibenzo[*b*,*e*]oxepin-11(6*H*)-one (29)**

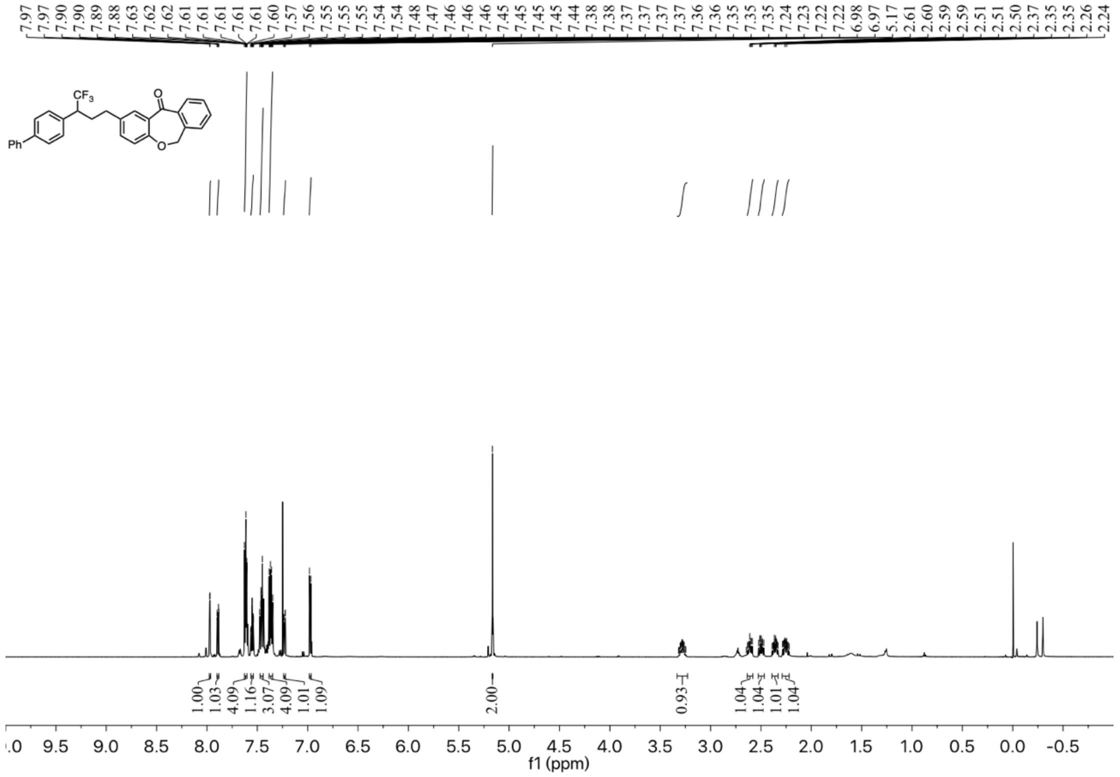

**$^{13}$C NMR (151 MHz, $CDCl_3$)** spectrum of **2-(3-([1,1'-biphenyl]-4-yl)-4,4,4-trifluorobutyl)dibenzo[*b*,*e*]oxepin-11(6*H*)-one (29)**

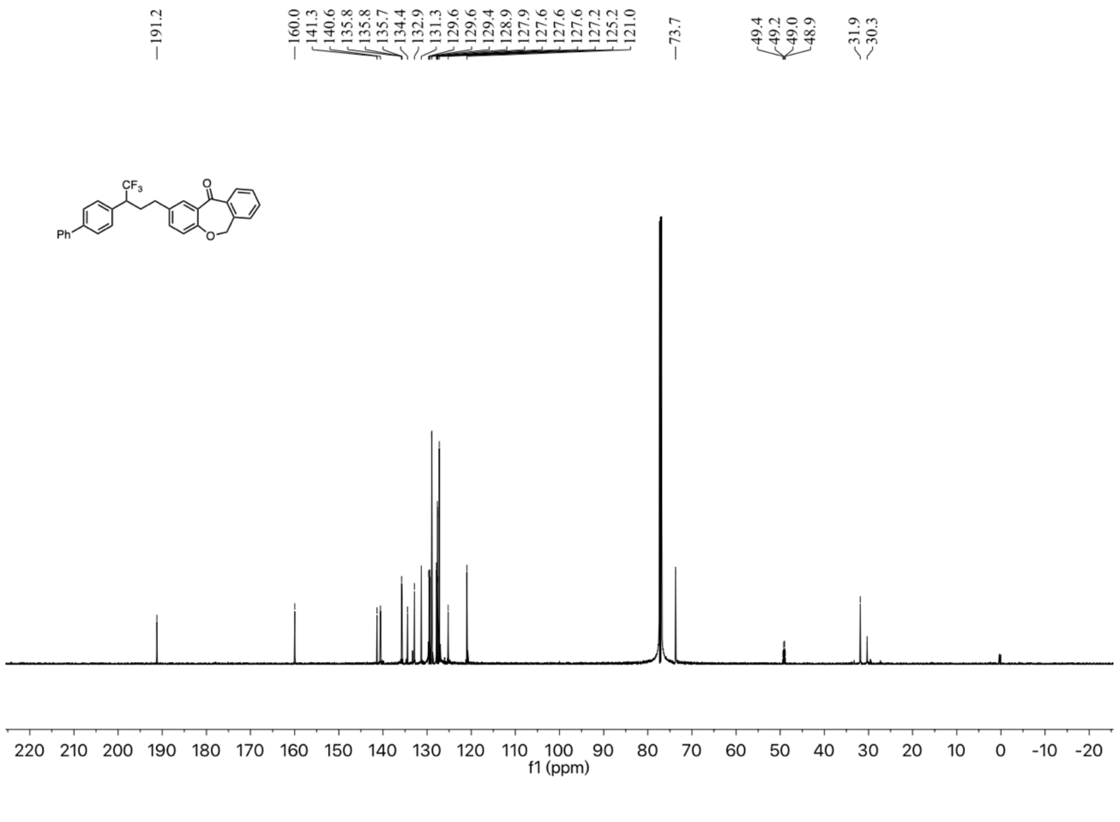

**$^{19}$F NMR (565 MHz, CDCl3)** spectrum of**2-(3-([1,1'-biphenyl]-4-yl)-4,4,4-trifluorobutyl)dibenzo[*b*,*e*]oxepin-11(6*H*)-one (29)**

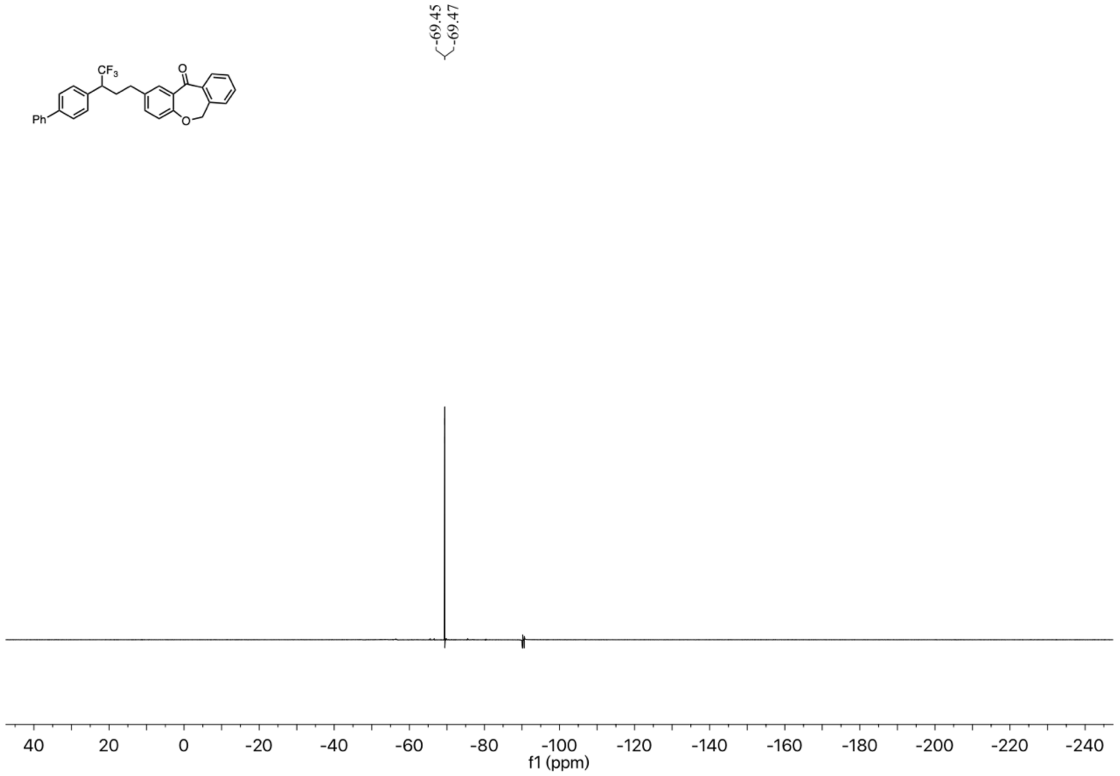


**$^{1}$H NMR (400 MHz, CDCl3)** spectrum of **(4-((4-([1,1'-biphenyl]-4-yl)-5,5,5-trifluoro-2-methylpentan-2-yl)oxy)phenyl)(4-chlorophenyl)methanone (30)**

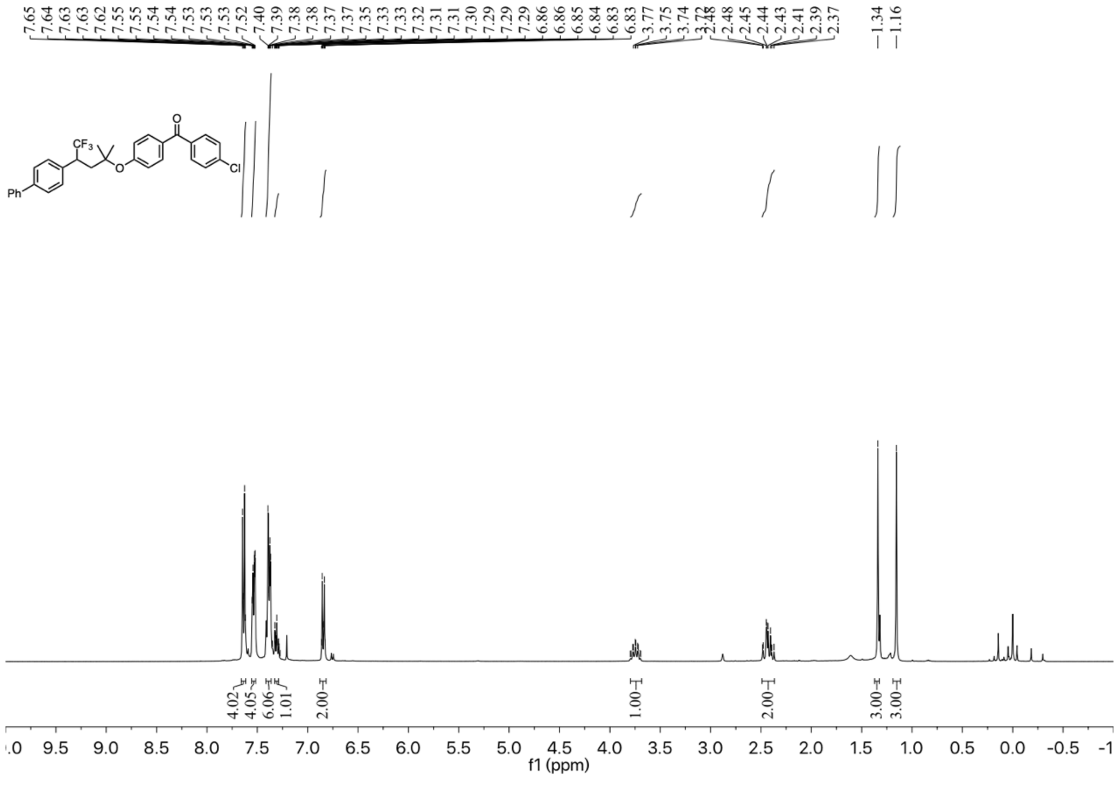

**$^{13}$C NMR (151 MHz, CDCl$_3$)** spectrum of **(4-((4-([1,1'-biphenyl]-4-yl)-5,5,5-trifluoro-2-methylpentan-2-yl)oxy)phenyl)(4-chlorophenyl)methanone (30)**

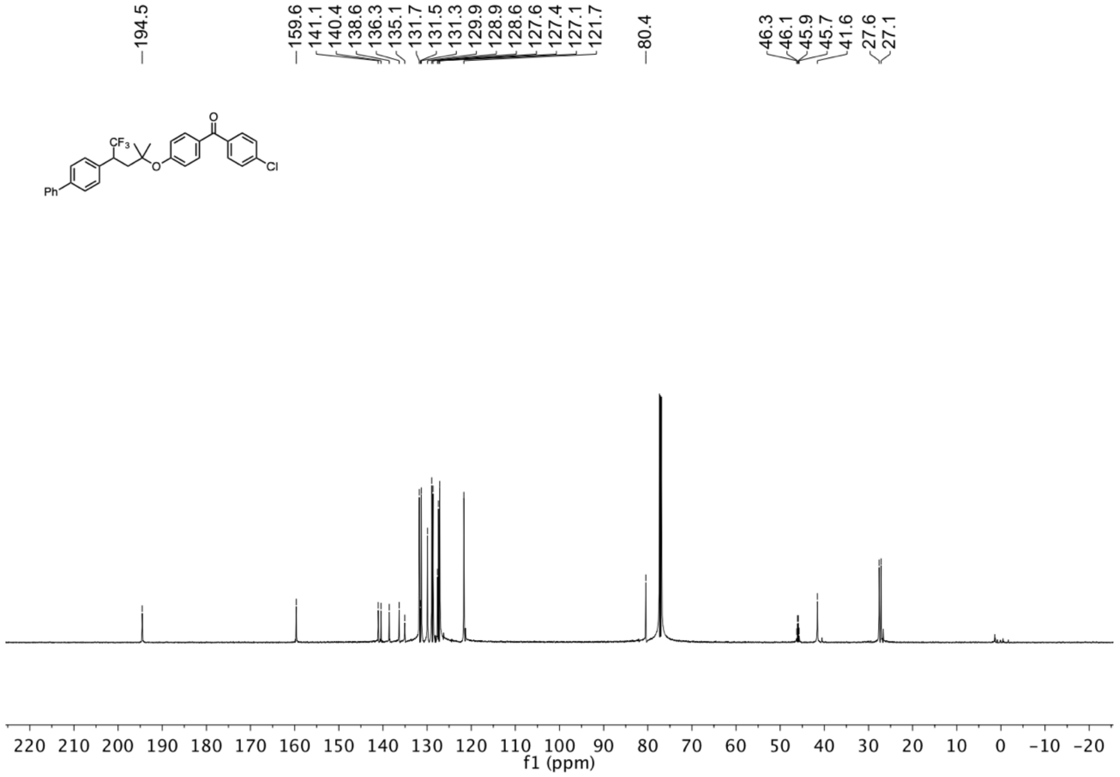

**$^{19}$F NMR (376 MHz, CDCl$_3$)** spectrum of **(4-((4-([1,1'-biphenyl]-4-yl)-5,5,5-trifluoro-2-methylpentan-2-yl)oxy)phenyl)(4-chlorophenyl)methanone (30)**

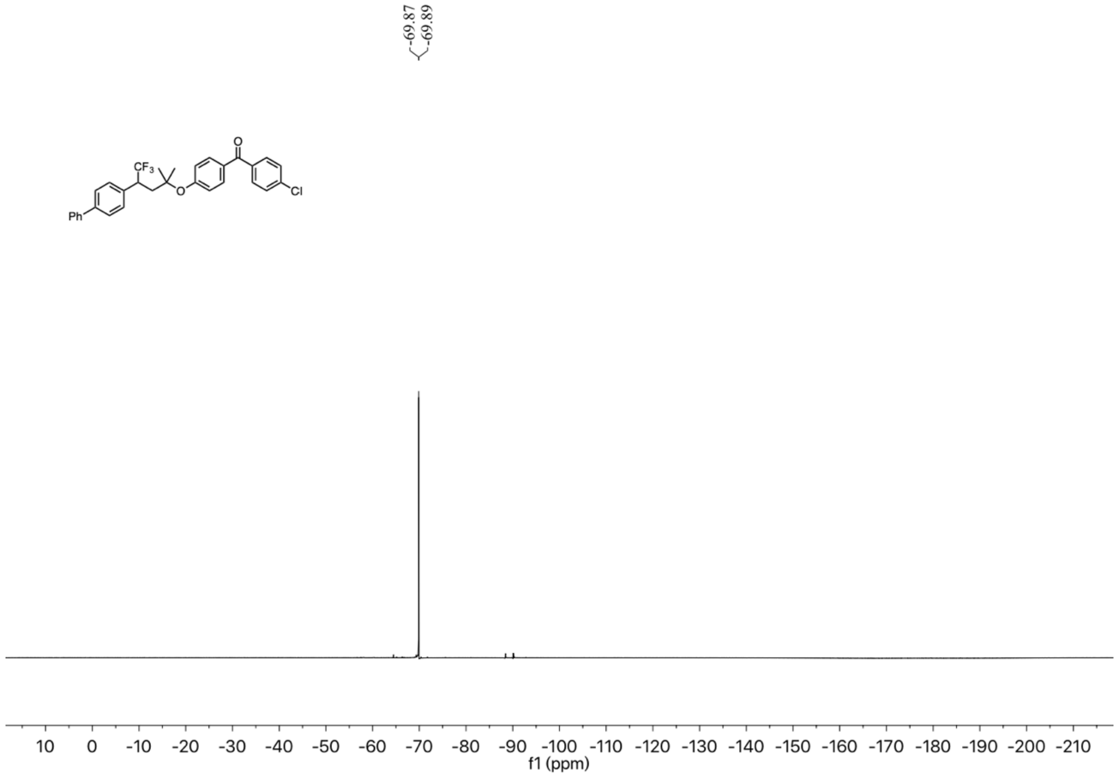

**$^{1}$H NMR (400 MHz, CDCl3)** spectrum of **4-(*N*,*N*-dipropylsulfamoyl)-*N*-(3-(1,1,1-trifluoro-4,4-dimethylpentan-2-yl)phenyl)benzamide (31)**

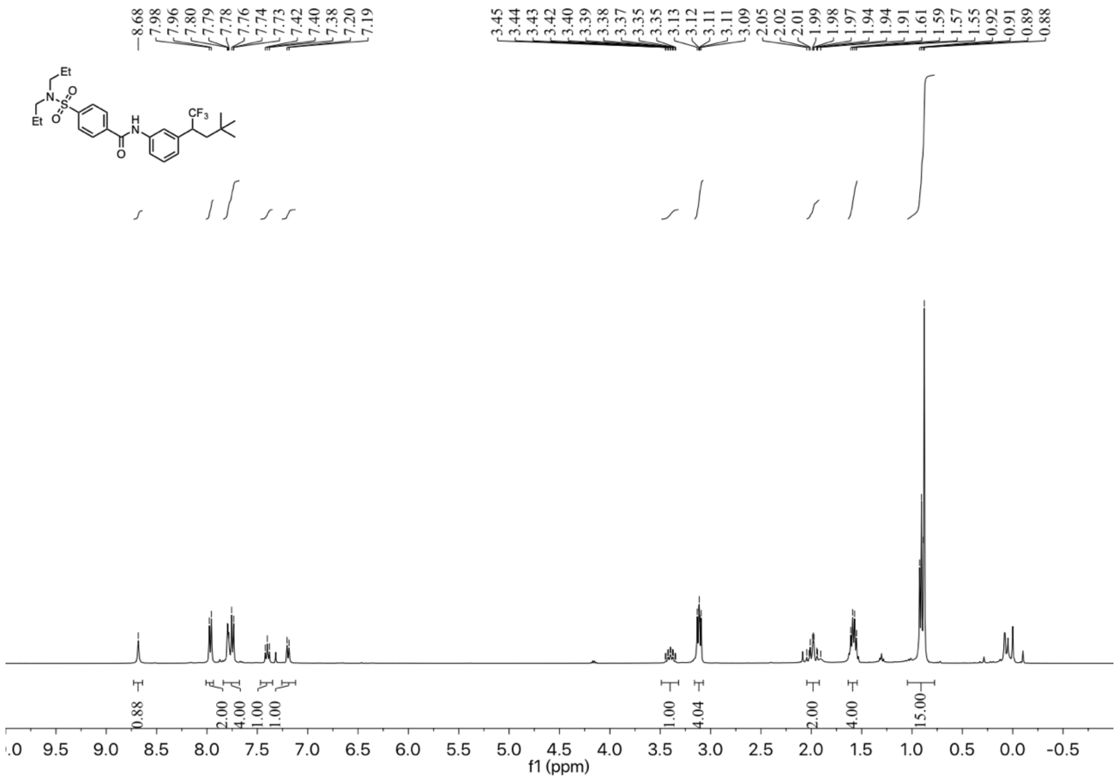

**$^{13}$C NMR (101 MHz, CDCl3)** spectrum of **4-(*N*,*N*-dipropylsulfamoyl)-*N*-(3-(1,1,1-trifluoro-4,4-dimethylpentan-2-yl)phenyl)benzamide (31)**

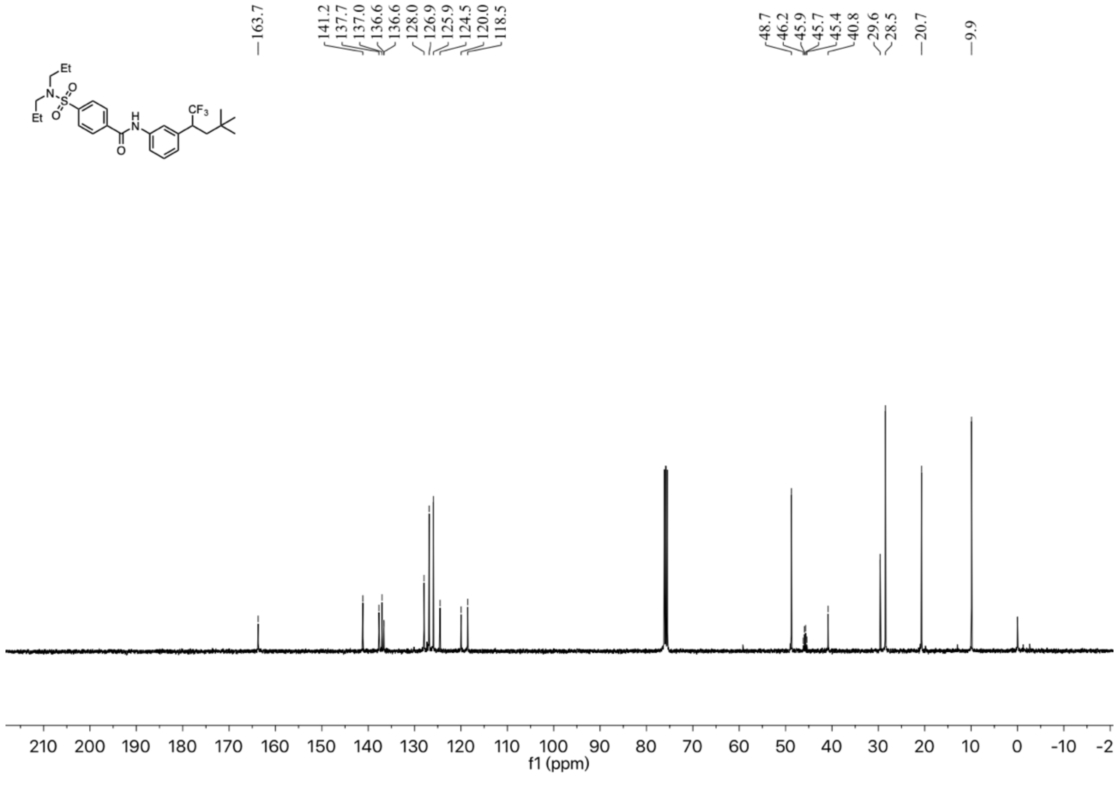

**$^{19}$F NMR (376 MHz, CDCl$_3$)** spectrum of **4-(*N*,*N*-dipropylsulfamoyl)-*N*-(3-(1,1,1-trifluoro-4,4-dimethylpentan-2-yl)phenyl)benzamide (31)**

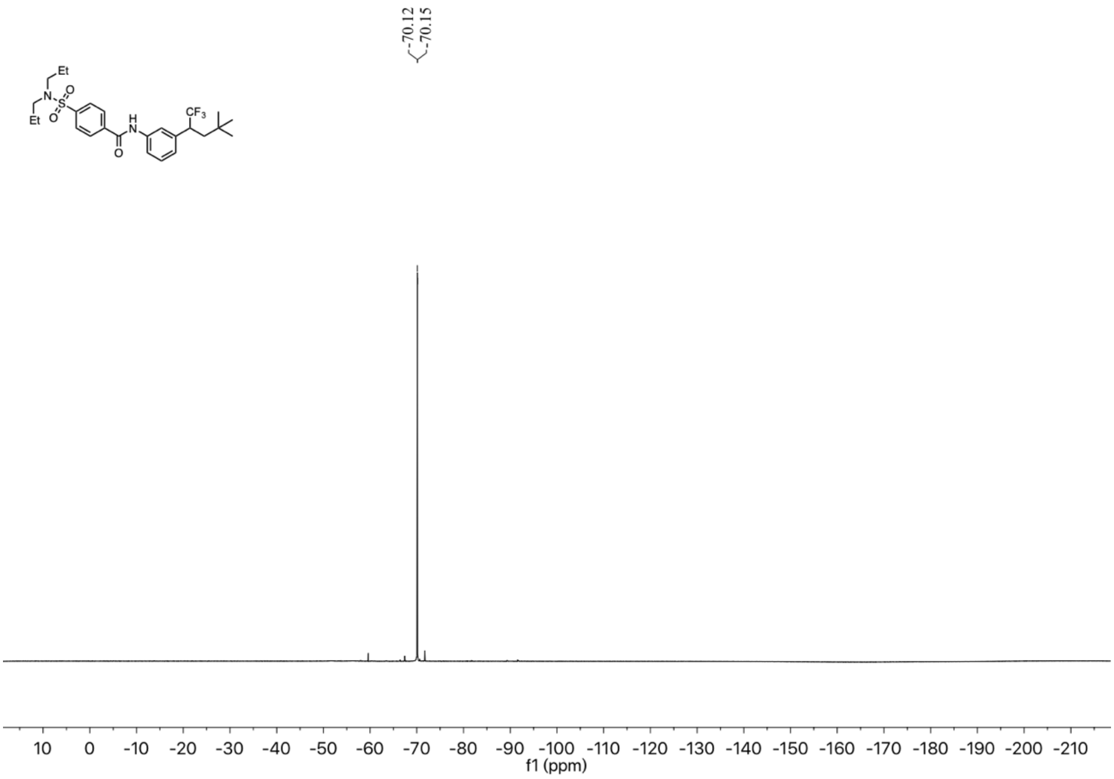


**$^{1}$H NMR (400 MHz, CDCl$_3$)** spectrum of **(5*S*,8*R*,9*R*,10*S*,13*R*,14*R*,17*R*)-17-((2*R*)-6-([1,1'-biphenyl]-4-yl)-7,7,7-trifluoroheptan-2-yl)-8,10,13-trimethyldodecahydro-3*H*-cyclopenta[*a*]phenanthrene-3,7,12(2*H*,4*H*)-trione (32)**

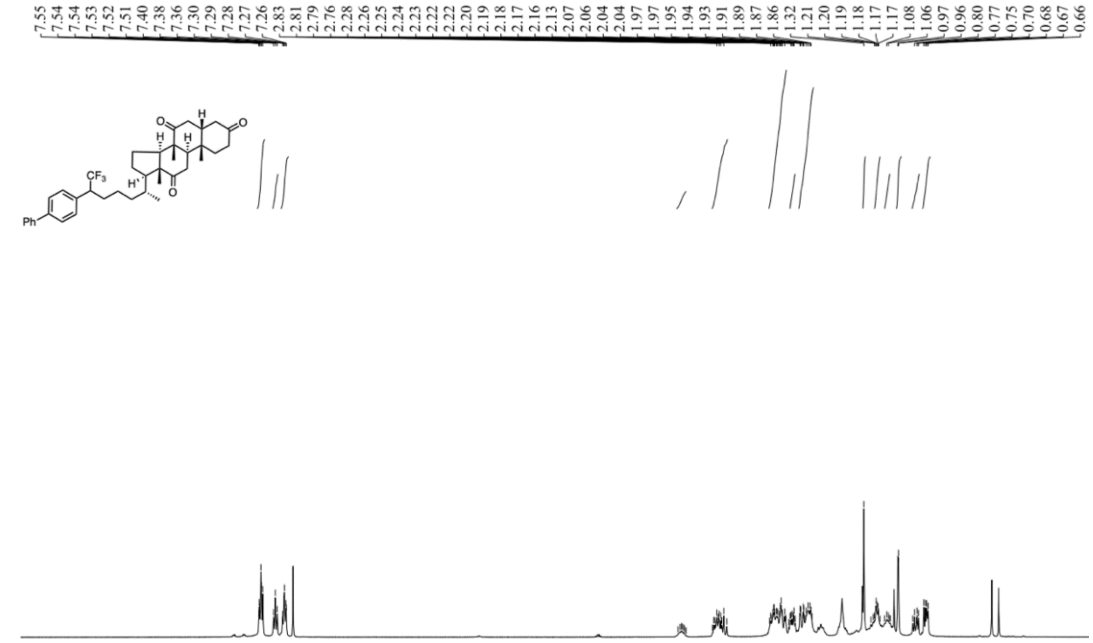

**$^{13}$C NMR (126 MHz, CDCl$_3$)** spectrum of **(5*S*,8*R*,9*R*,10*S*,13*R*,14*R*,17*R*)-17-((2*R*)-6-([1,1'-biphenyl]-4-yl)-7,7,7-trifluoroheptan-2-yl)-8,10,13-trimethyldodecahydro-3*H*-cyclopenta[*a*]phenanthrene-3,7,12(2*H*,4*H*)-trione (32)**

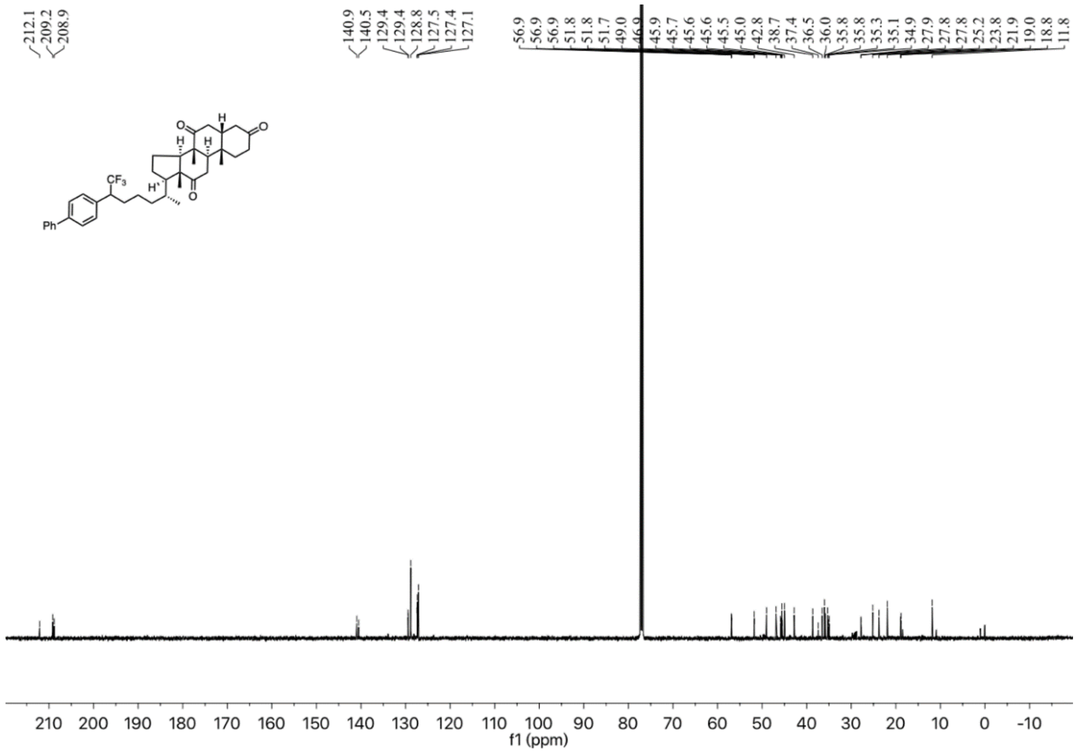


**$^{19}$F NMR (376 MHz, CDCl$_3$)** spectrum of **(5*S*,8*R*,9*R*,10*S*,13*R*,14*R*,17*R*)-17-((2*R*)-6-([1,1'-biphenyl]-4-yl)-7,7,7-trifluoroheptan-2-yl)-8,10,13-trimethyldodecahydro-3*H*-cyclopenta[*a*]phenanthrene-3,7,12(2*H*,4*H*)-trione (32)**

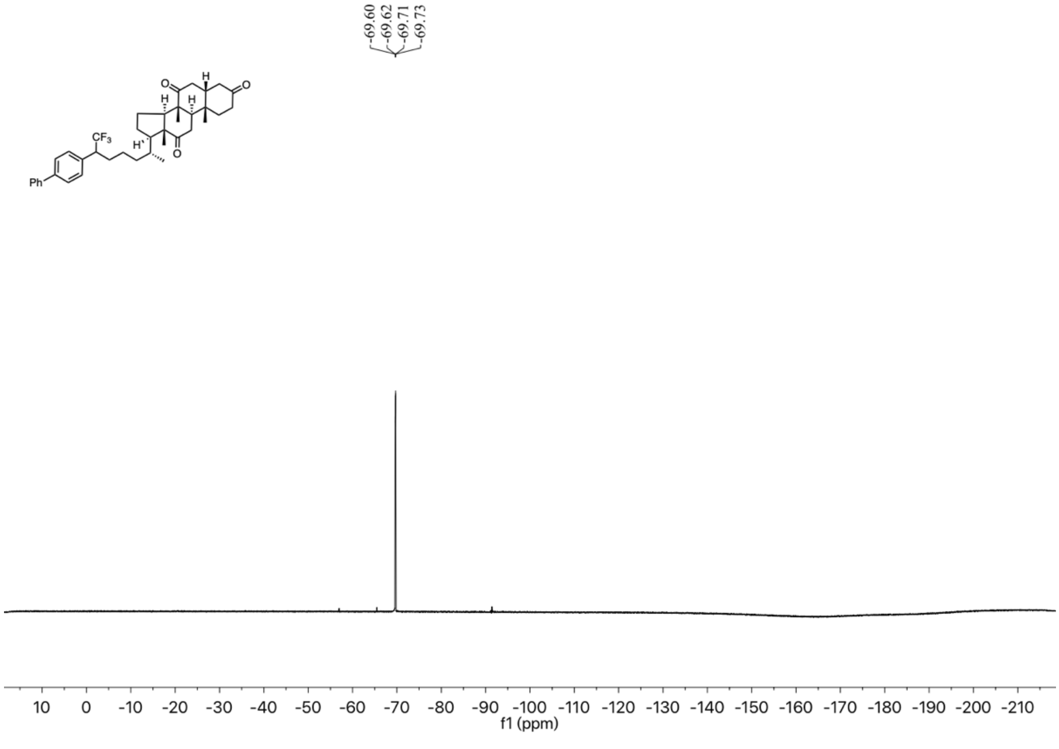

**$^{1}$H NMR (400 MHz, CDCl3)** spectrum of **2-((6-(4-(tert-butyl)phenyl)-7,7-difluoro-4,4-dimethylhept-6-en-1-yl)oxy)-1,4-dimethylbenzene (33)**

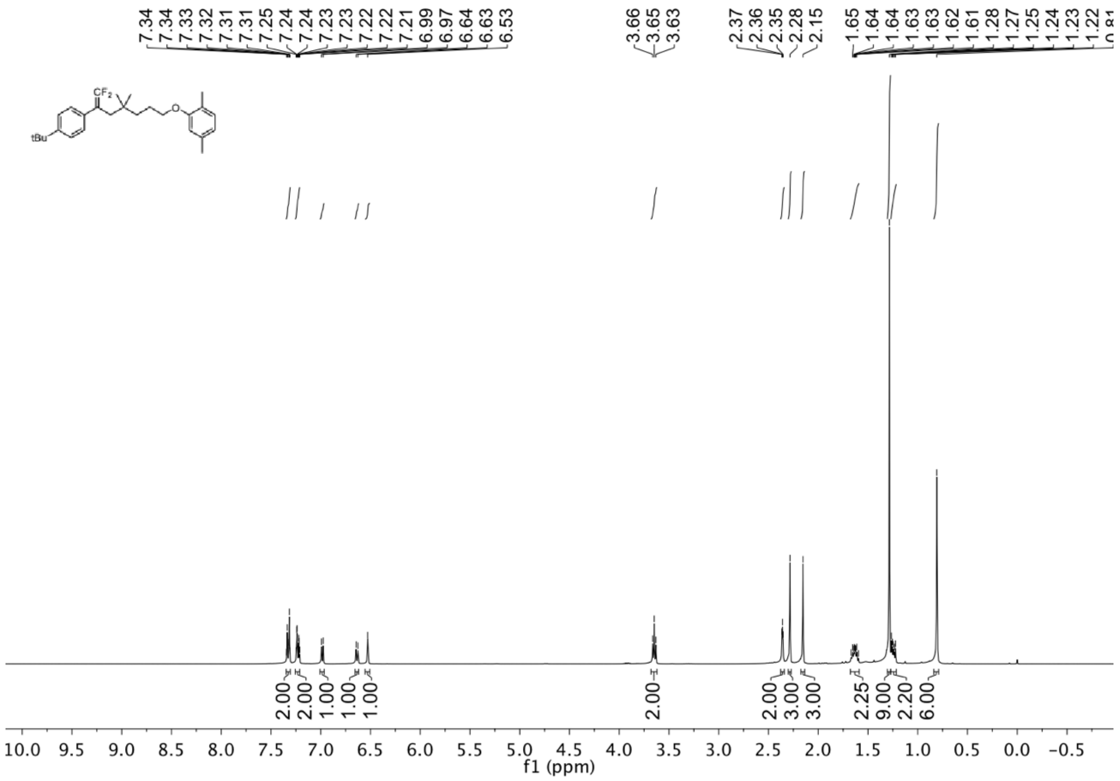

**$^{13}$C NMR (101 MHz, CDCl3)** spectrum of **2-((6-(4-(tert-butyl)phenyl)-7,7-difluoro-4,4-dimethylhept-6-en-1-yl)oxy)-1,4-dimethylbenzene (33)**

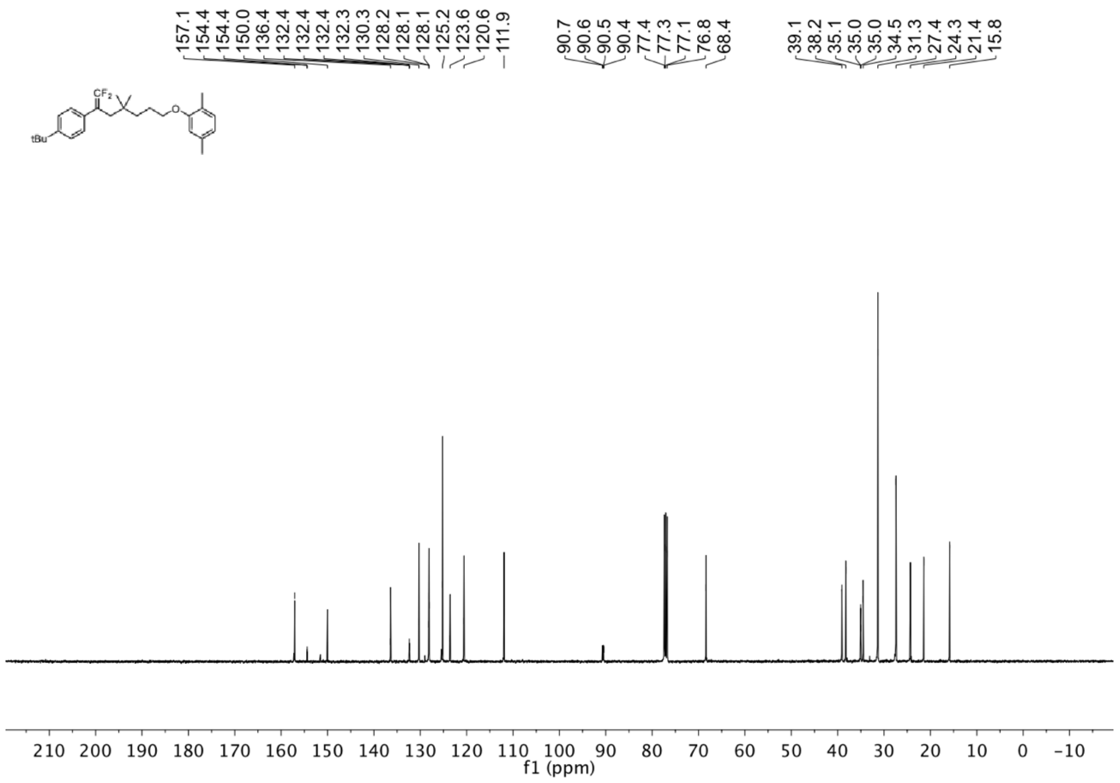

**$^{19}$F NMR (376 MHz, CDCl$_3$)** spectrum of **2-((6-(4-(tert-butyl)phenyl)-7,7-difluoro-4,4-dimethylhept-6-en-1-yl)oxy)-1,4-dimethylbenzene (33)**

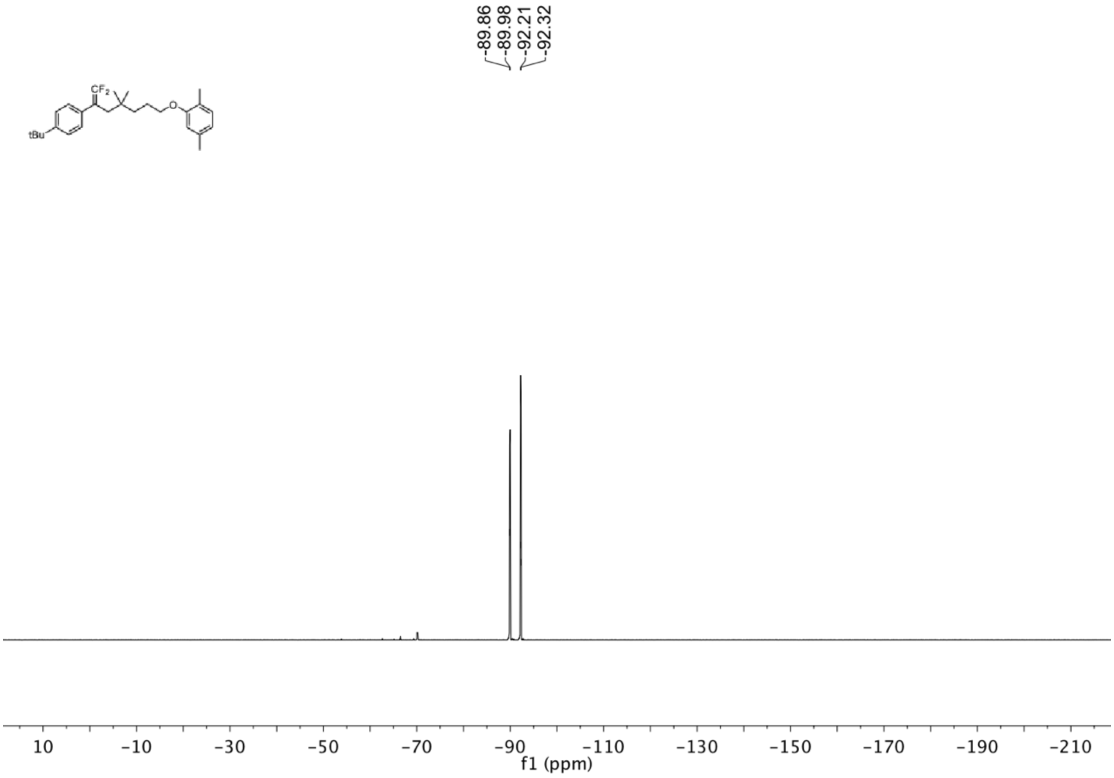


**$^{1}$H NMR (400 MHz, CDCl$_3$)** spectrum of **2-((7,7-difluoro-6-(4-methoxyphenyl)-4,4-dimethylhept-6-en-1-yl)oxy)-1,4-dimethylbenzene (34)**

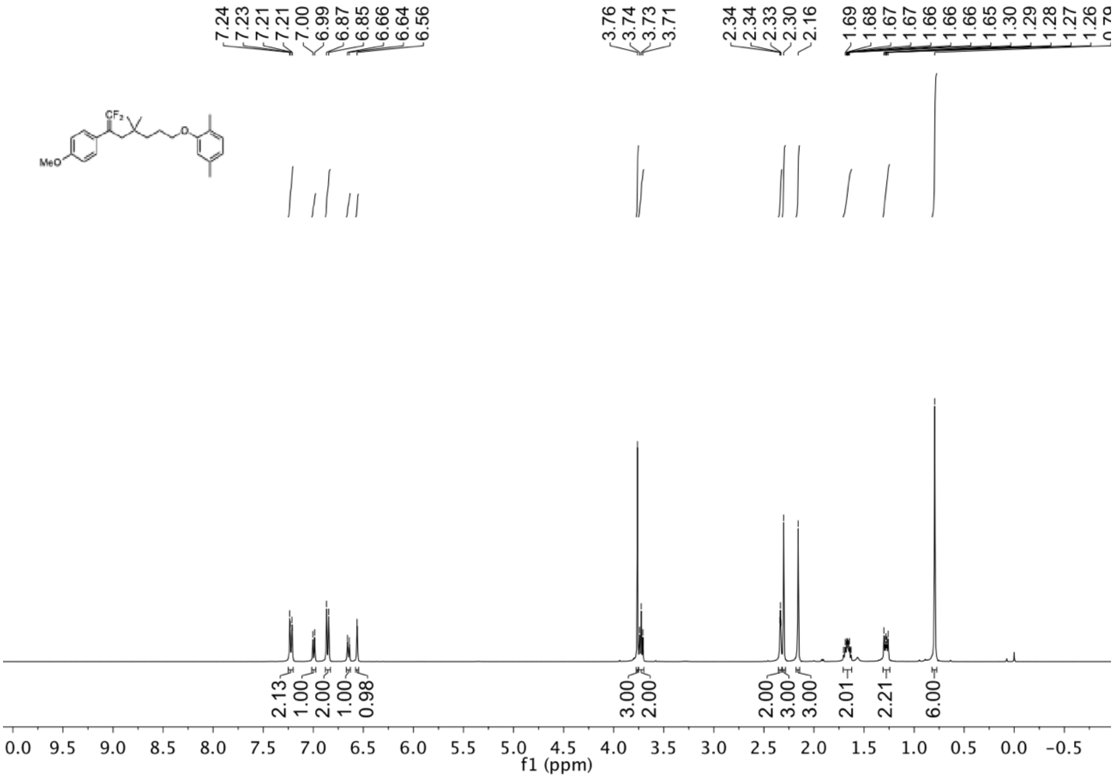

$^{13}$C NMR (126 MHz, CDCl$_3$) spectrum of **2-((7,7-difluoro-6-(4-methoxyphenyl)-4,4-dimethylhept-6-en-1-yl)oxy)-1,4-dimethylbenzene (34)**

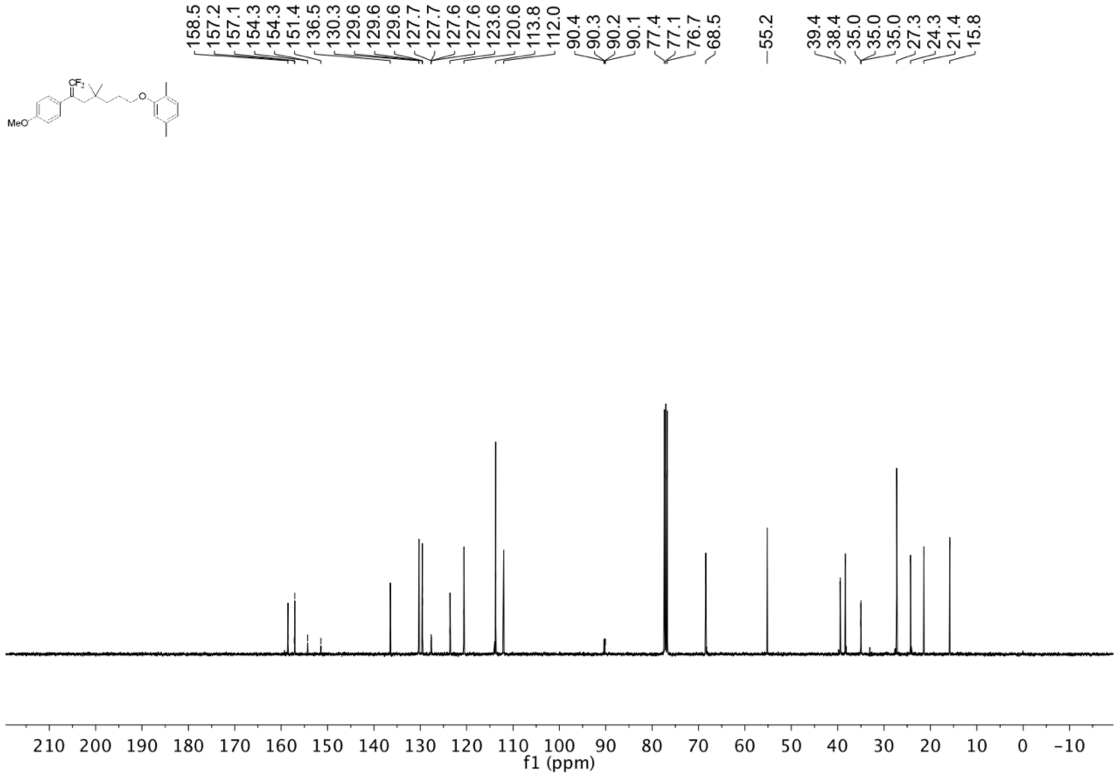


$^{19}$F NMR (376 MHz, CDCl$_3$) spectrum of **2-((7,7-difluoro-6-(4-methoxyphenyl)-4,4-dimethylhept-6-en-1-yl)oxy)-1,4-dimethylbenzene (34)**

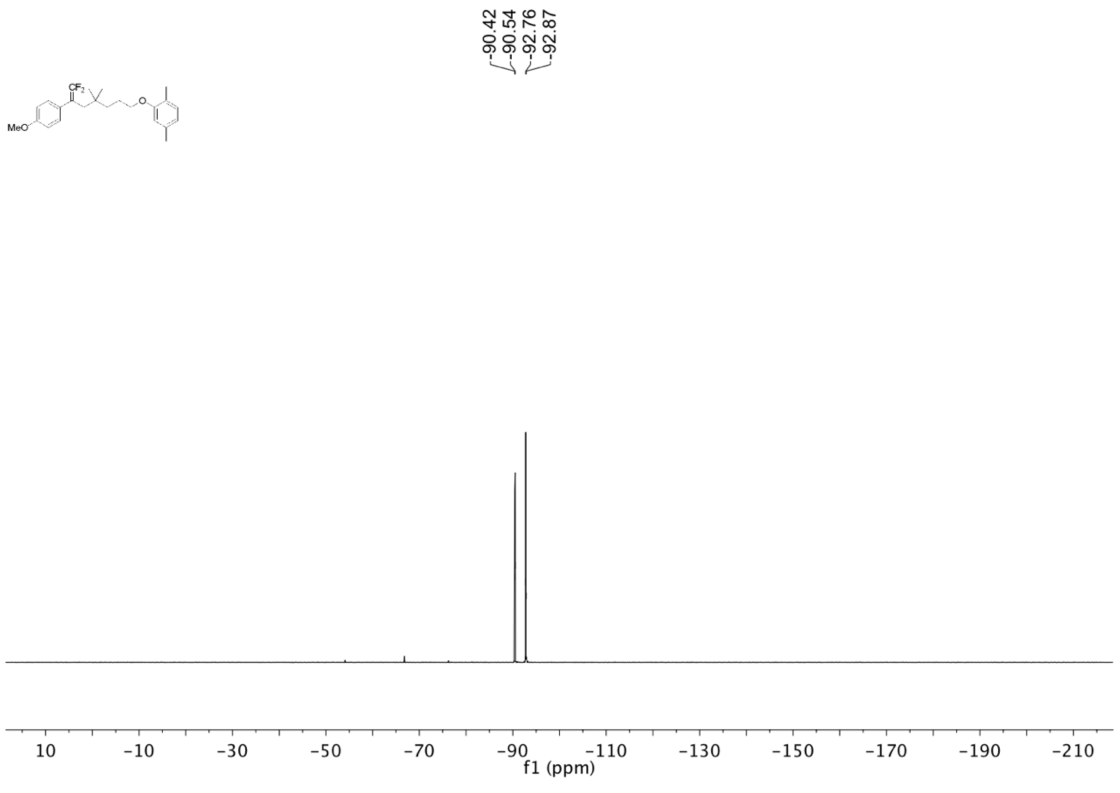

**$^{1}$H NMR (400 MHz, $CDCl_3$)** spectrum of **2-((7,7-difluoro-4,4-dimethyl-6-(4-phenoxyphenyl)hept-6-en-1-yl)oxy)-1,4-dimethylbenzene (35)**

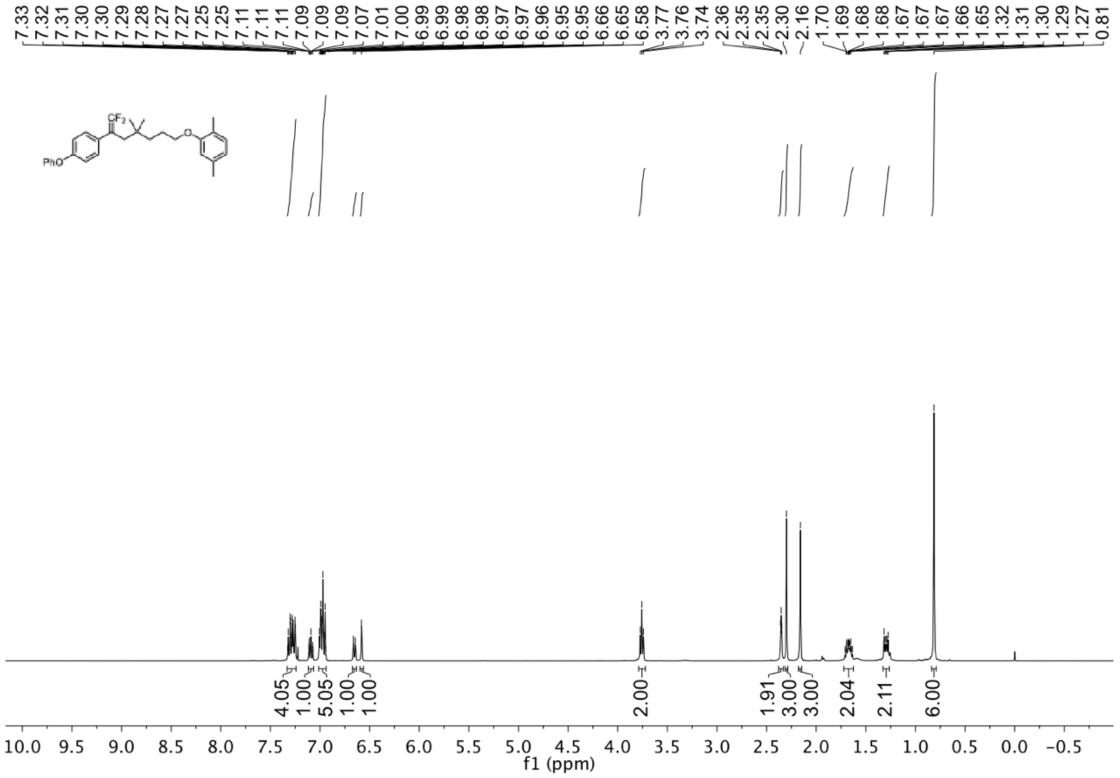


**$^{13}$C NMR (101 MHz, $CDCl_3$)** spectrum of **2-((7,7-difluoro-4,4-dimethyl-6-(4-phenoxyphenyl)hept-6-en-1-yl)oxy)-1,4-dimethylbenzene (35)**

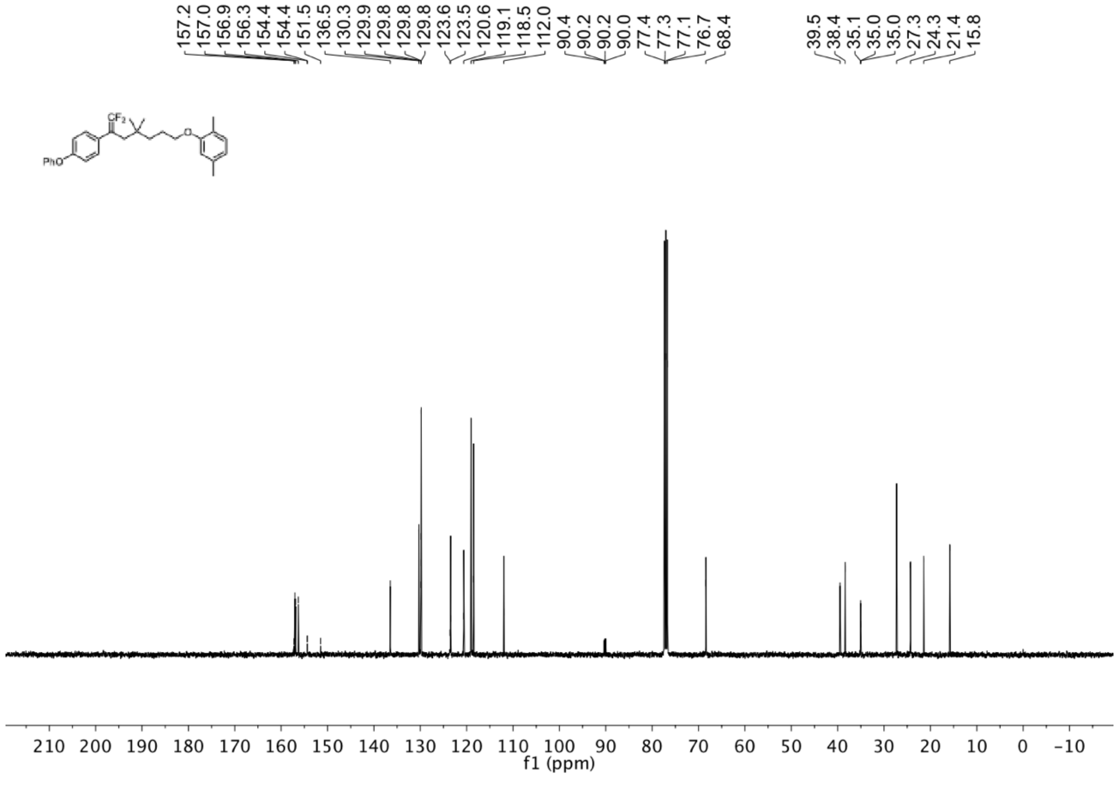

**$^{19}$F NMR (376 MHz, $CDCl_3$)** spectrum of **2-((7,7-difluoro-4,4-dimethyl-6-(4-phenoxyphenyl)hept-6-en-1-yl)oxy)-1,4-dimethylbenzene (35)**

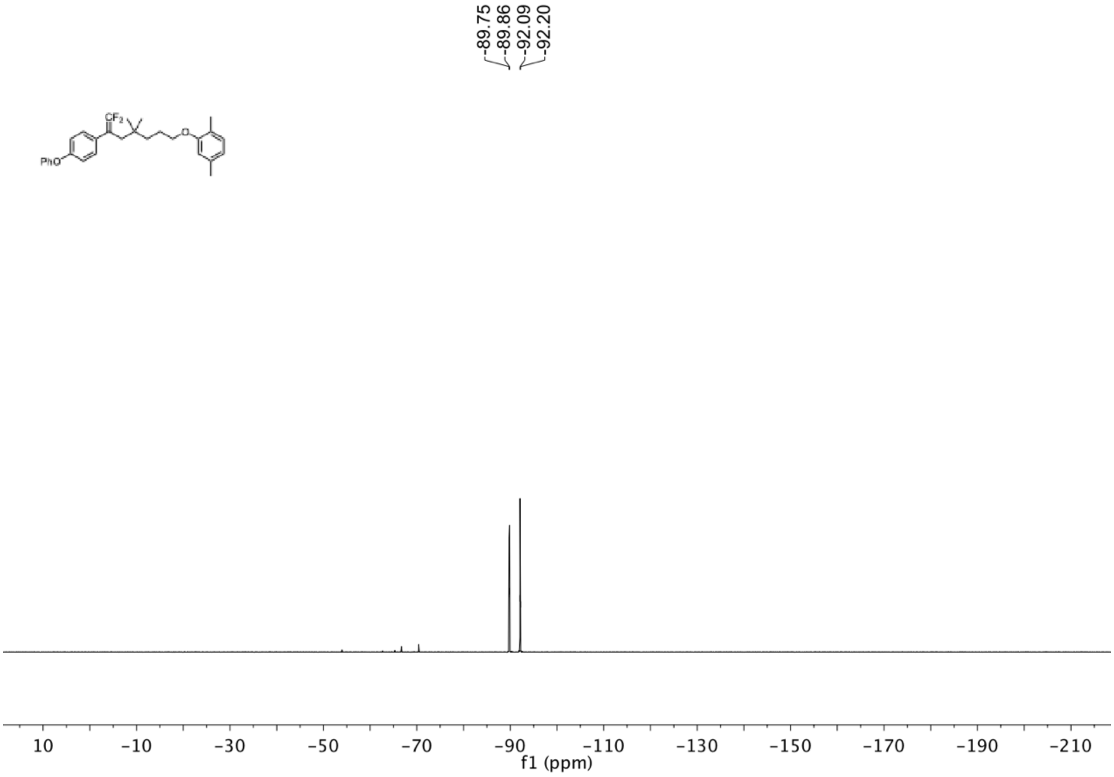


**$^{1}$H NMR (400 MHz, $CDCl_3$)** spectrum of **2-((7,7-difluoro-4,4-dimethyl-6-(4-(trifluoromethoxy)phenyl)hept-6-en-1-yl)oxy)-1,4-dimethylbenzene (36)**

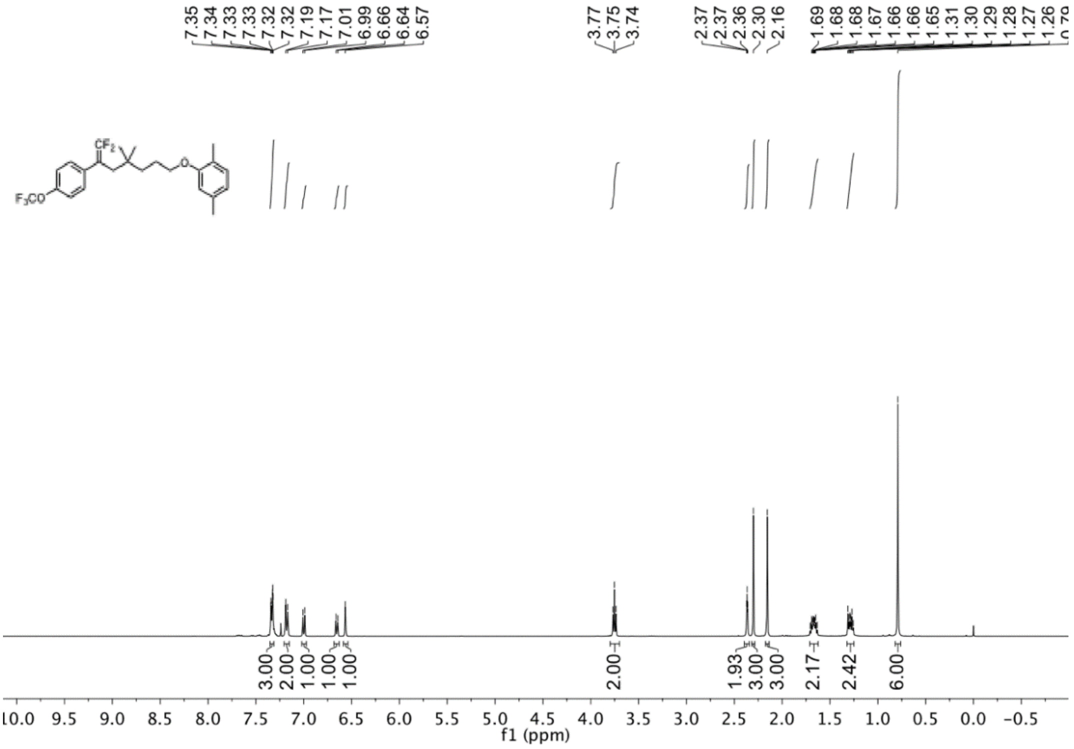

**$^{13}$C NMR (101 MHz, CDCl$_3$)** spectrum of **2-((7,7-difluoro-4,4-dimethyl-6-(4-(trifluoromethoxy)phenyl)hept-6-en-1-yl)oxy)-1,4-dimethylbenzene (36)**

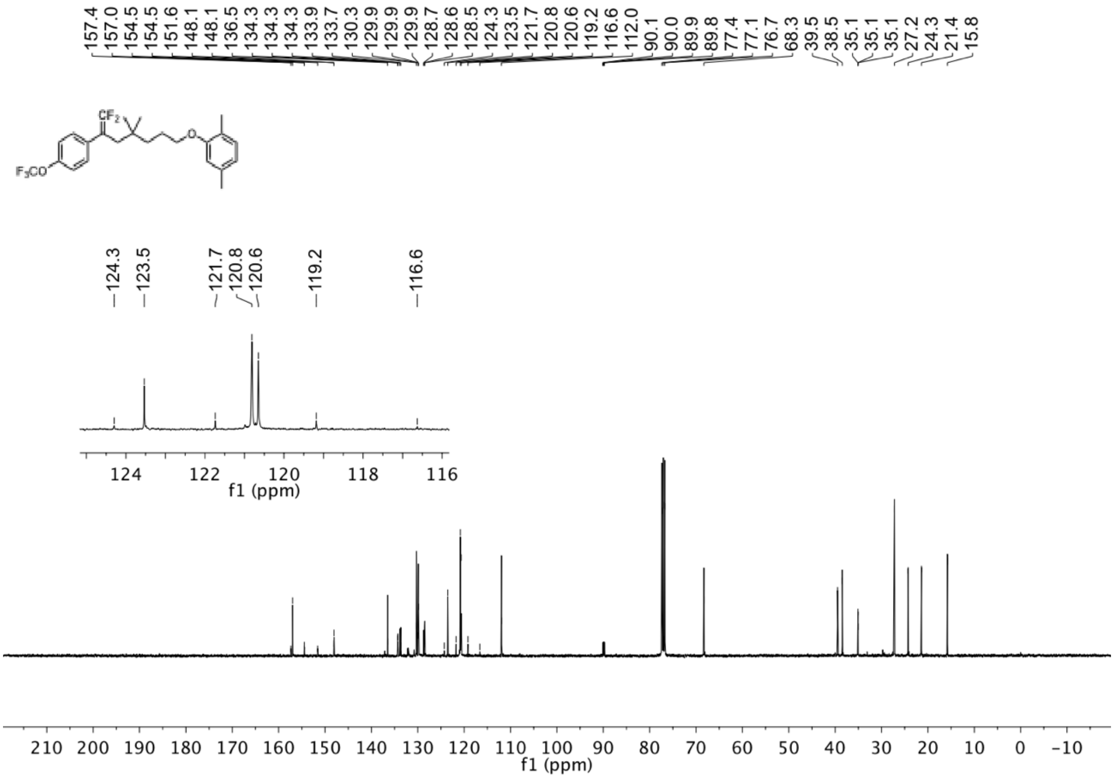


**$^{19}$F NMR (376 MHz, CDCl$_3$)** spectrum of **2-((7,7-difluoro-4,4-dimethyl-6-(4-(trifluoromethoxy)phenyl)hept-6-en-1-yl)oxy)-1,4-dimethylbenzene (36)**

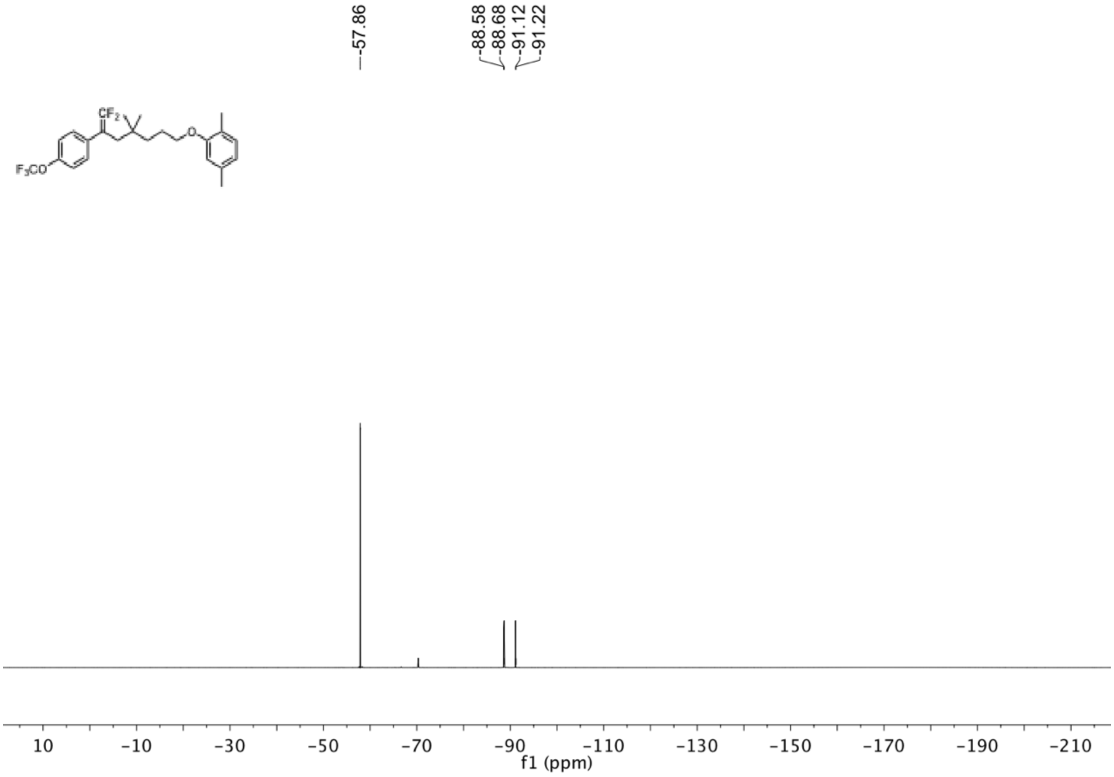

**$^{1}$H NMR (400 MHz, CDCl$_3$)** spectrum of **(4-(7-(2,5-dimethylphenoxy)-1,1-difluoro-4,4-dimethylhept-1-en-2-yl)phenyl)(methyl)sulfane (37)**

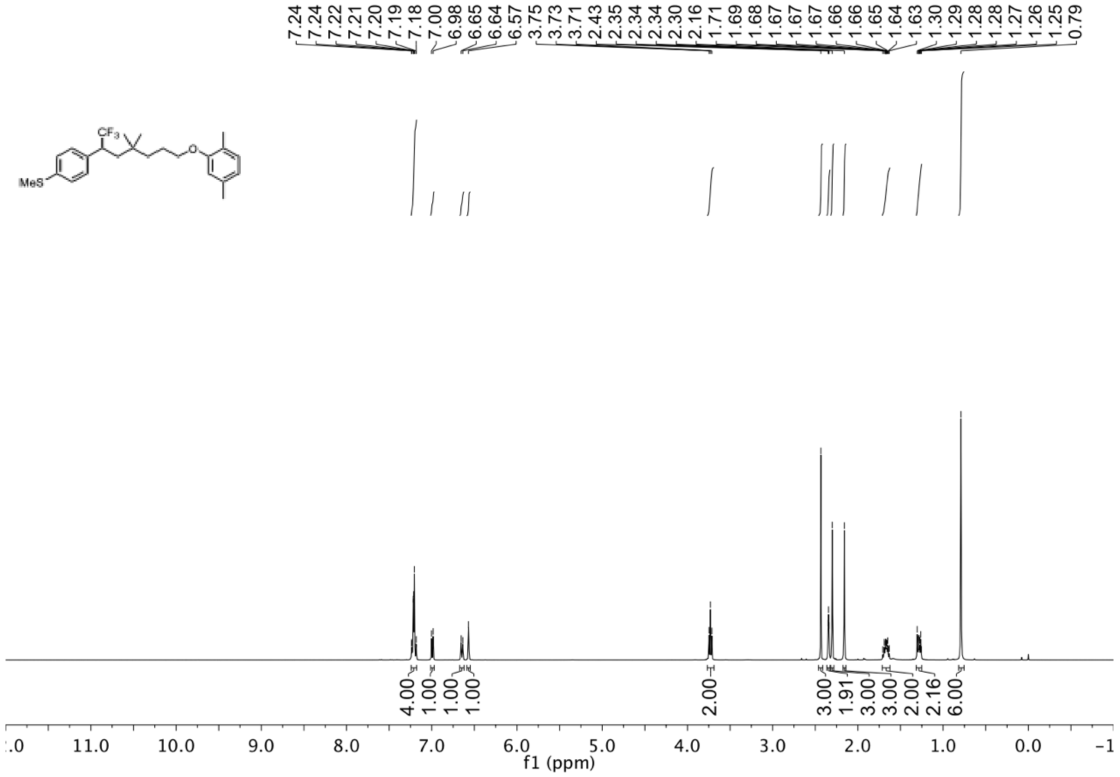


**$^{13}$C NMR (101 MHz, CDCl$_3$)** spectrum of **(4-(7-(2,5-dimethylphenoxy)-1,1-difluoro-4,4-dimethylhept-1-en-2-yl)phenyl)(methyl)sulfane (37)**

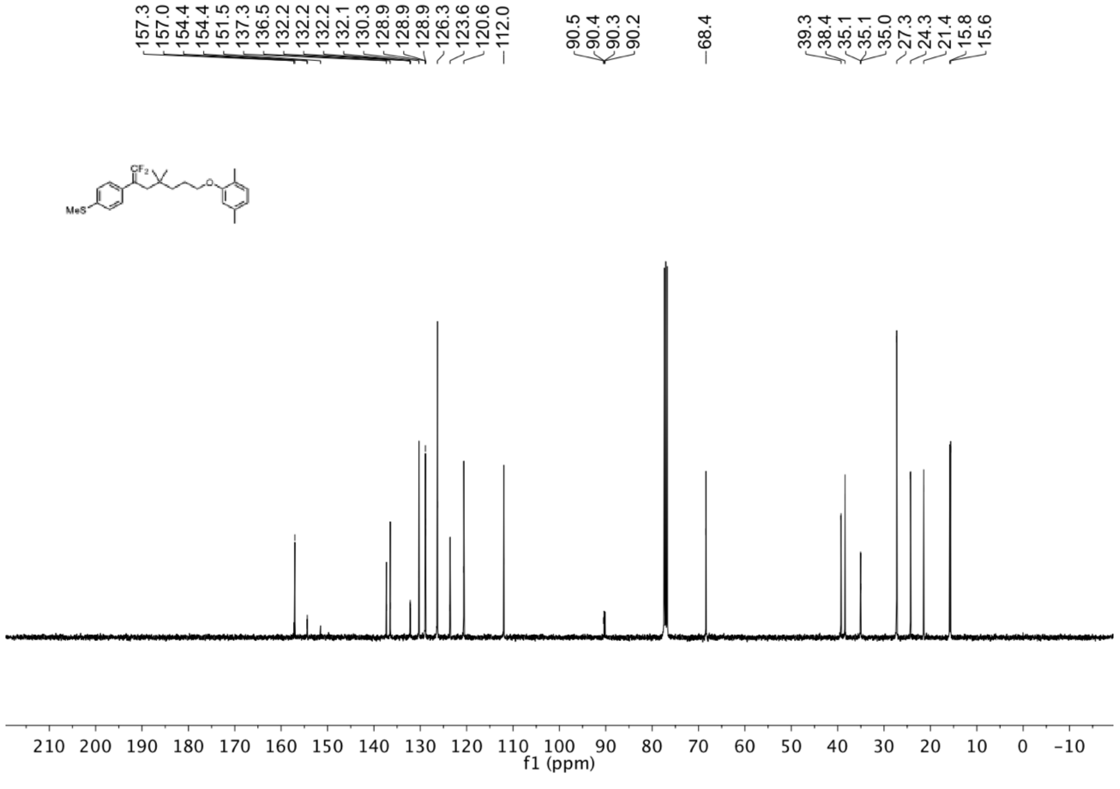

**$^{19}$F NMR (376 MHz, $CDCl_3$)** spectrum of **(4-(7-(2,5-dimethylphenoxy)-1,1-difluoro-4,4-dimethylhept-1-en-2-yl)phenyl)(methyl)sulfane (37)**

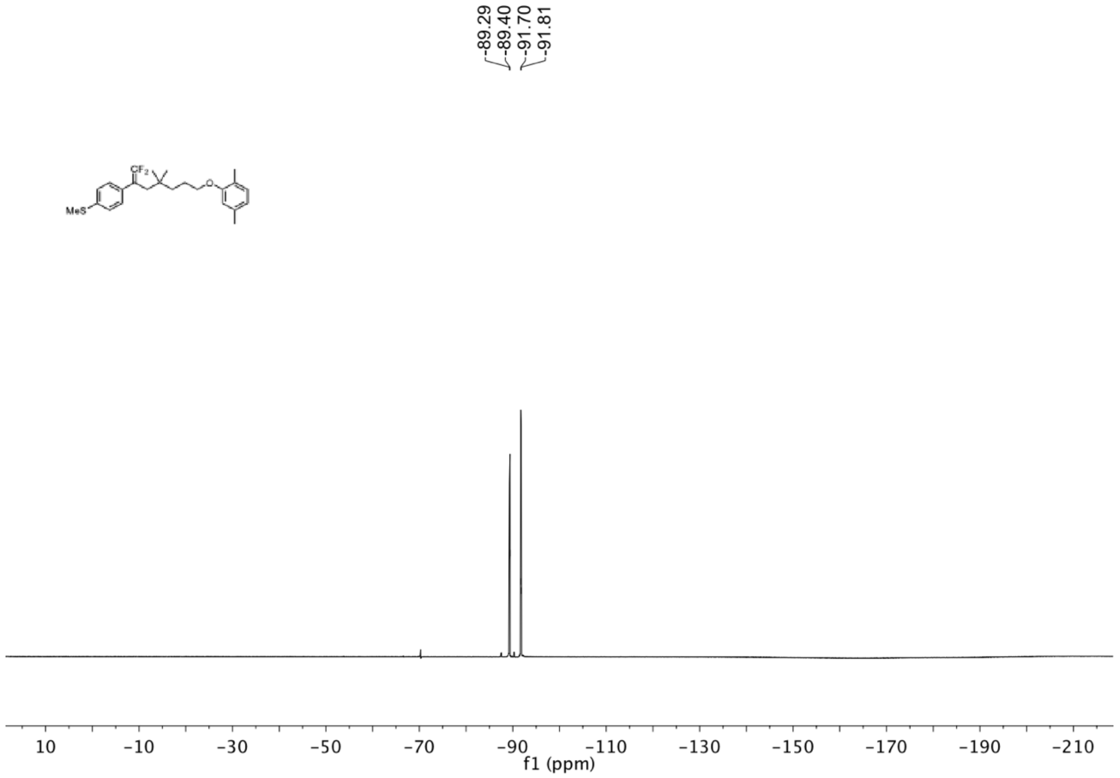


**$^{1}$H NMR (400 MHz, $CDCl_3$)** spectrum of **2-((6-(4-chlorophenyl)-7,7-difluoro-4,4-dimethylhept-6-en-1-yl)oxy)-1,4-dimethylbenzene (38)**

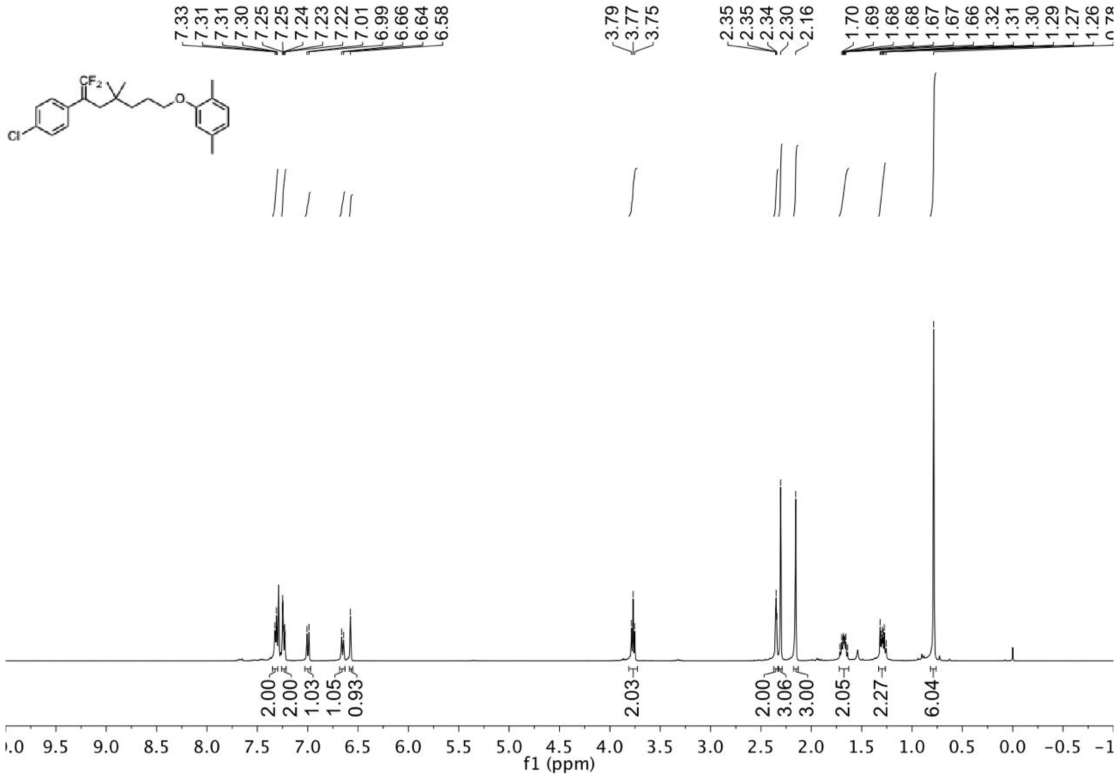

**$^{13}$C NMR (126 MHz, CDCl$_3$)** spectrum of **2-((6-(4-chlorophenyl)-7,7-difluoro-4,4-dimethylhept-6-en-1-yl)oxy)-1,4-dimethylbenzene (38)**

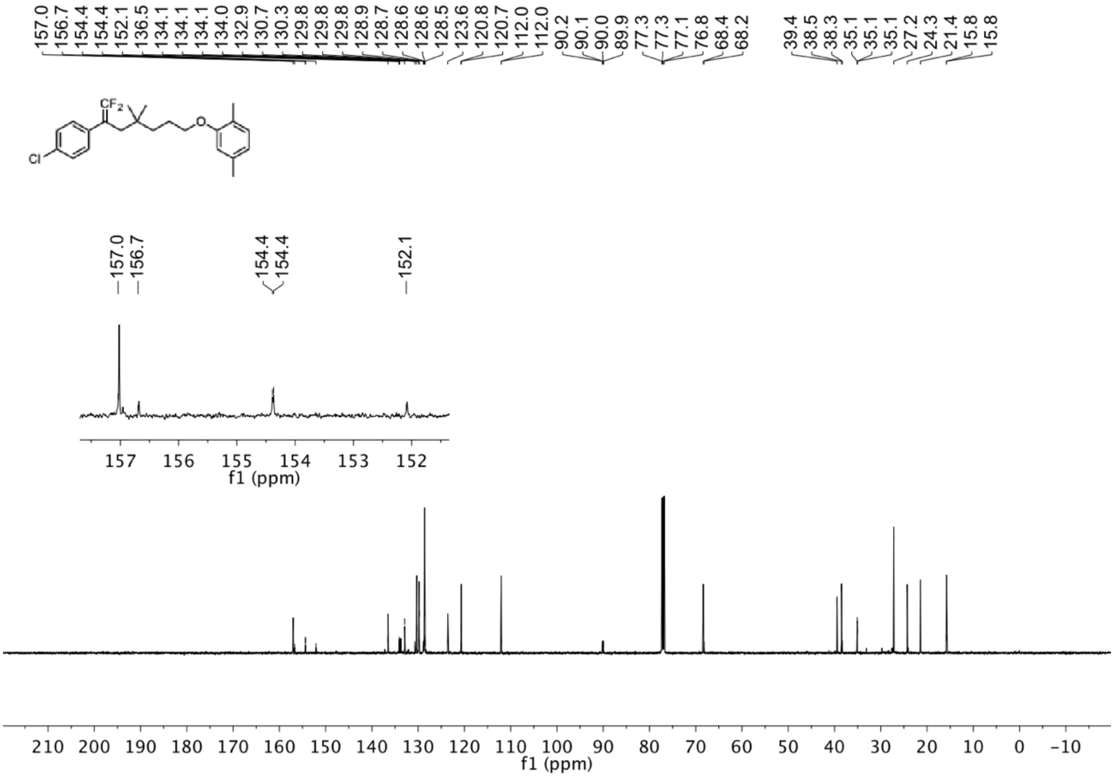


**$^{19}$F NMR (376 MHz, CDCl$_3$)** spectrum of**2-((6-(4-chlorophenyl)-7,7-difluoro-4,4-dimethylhept-6-en-1-yl)oxy)-1,4-dimethylbenzene (38)**

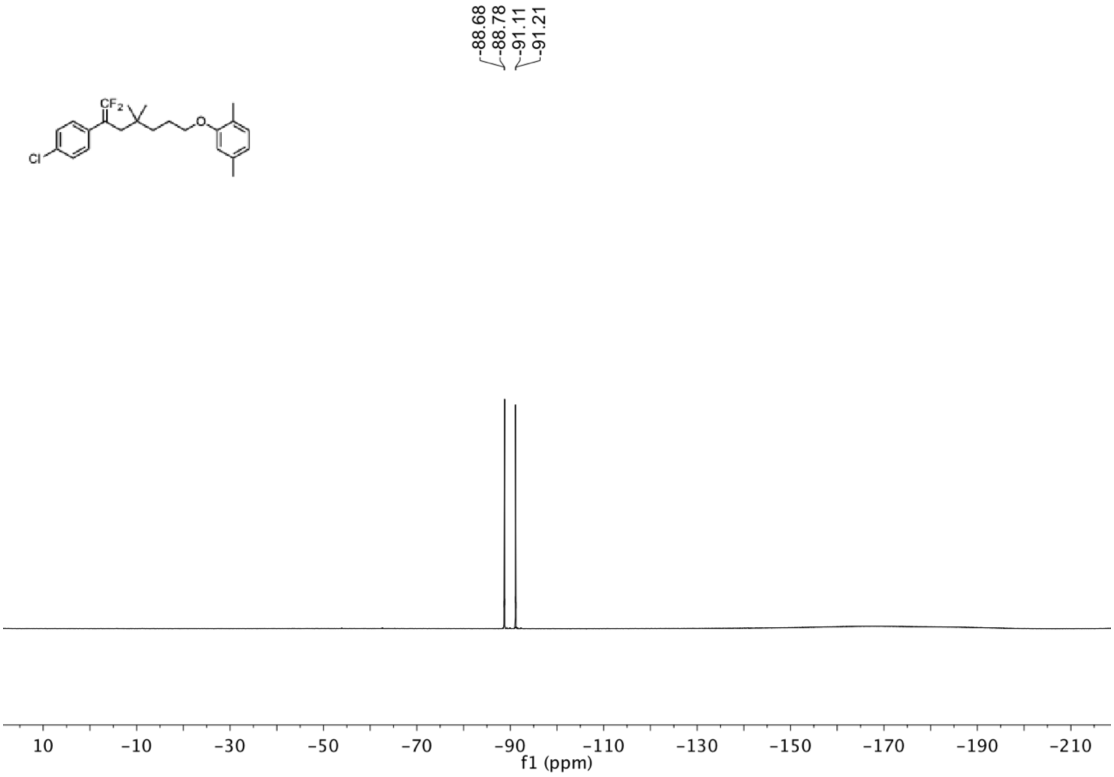

**$^{1}H$ NMR (400 MHz, $CDCl_3$)** spectrum of **2-((6-(4-bromophenyl)-7,7-difluoro-4,4-dimethylhept-6-en-1-yl)oxy)-1,4-dimethylbenzene (39)**

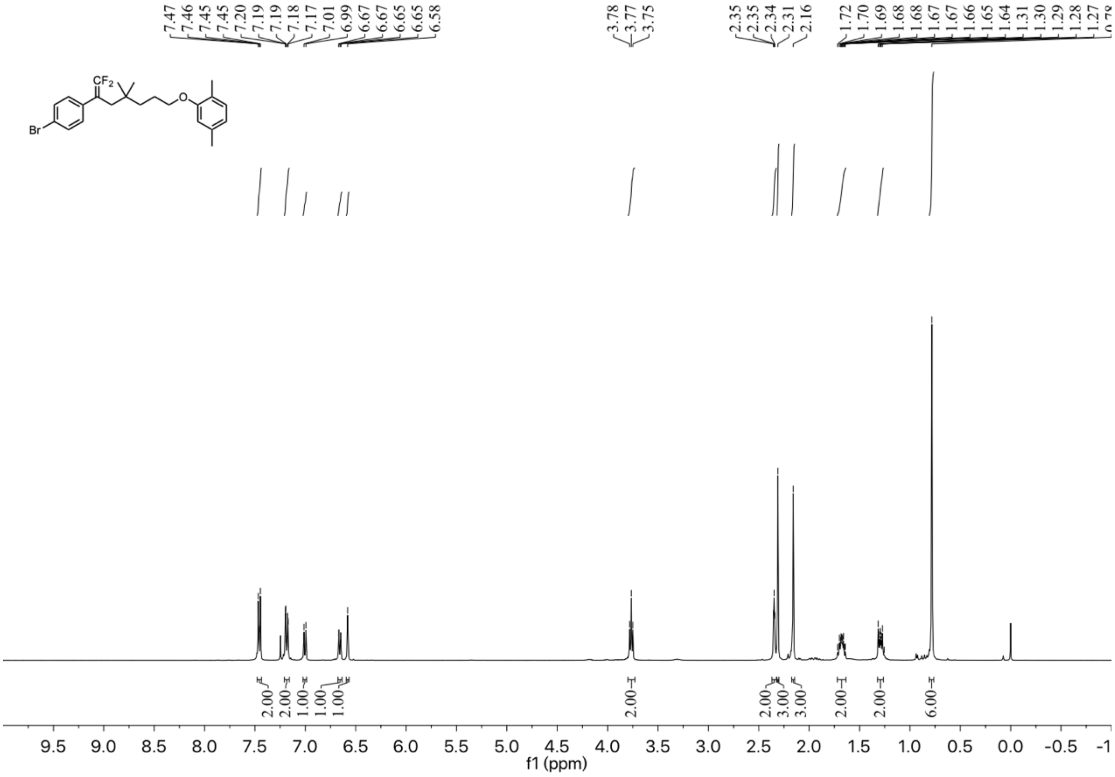


**$^{13}C$ NMR (126 MHz, $CDCl_3$)** spectrum of **2-((6-(4-bromophenyl)-7,7-difluoro-4,4-dimethylhept-6-en-1-yl)oxy)-1,4-dimethylbenzene (39)**

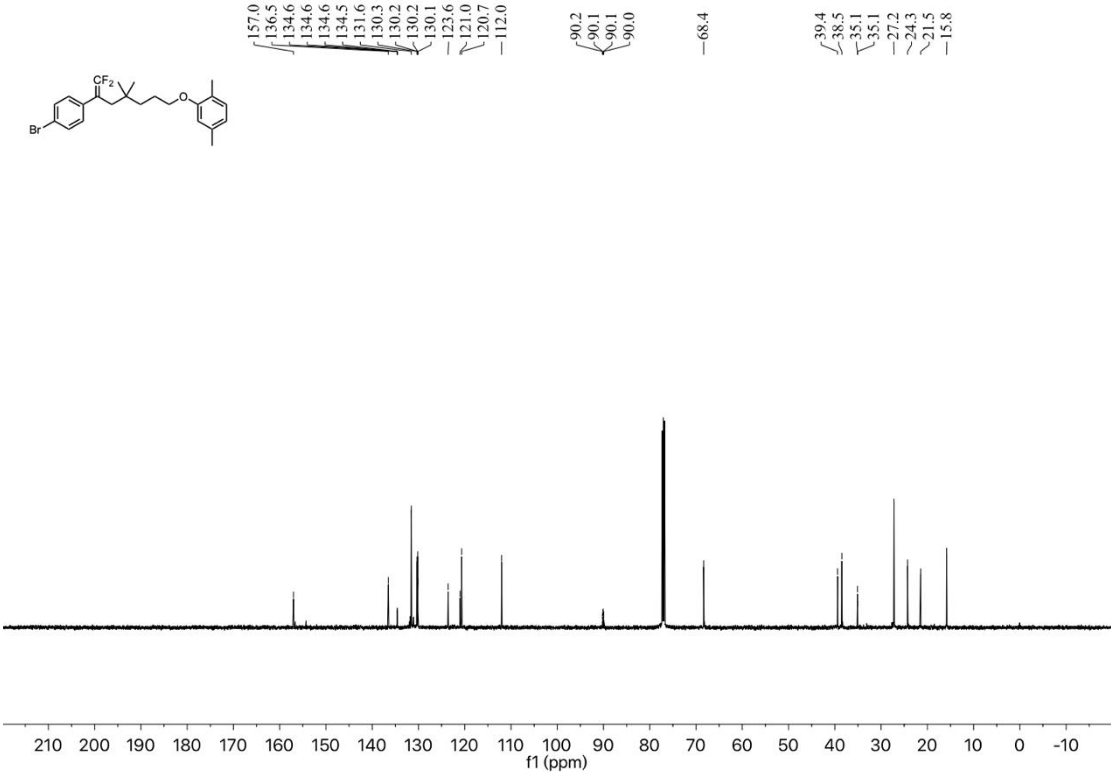

**$^{19}$F NMR (376 MHz, CDCl$_3$)** spectrum of **2-((6-(4-bromophenyl)-7,7-difluoro-4,4-dimethylhept-6-en-1-yl)oxy)-1,4-dimethylbenzene (39)**

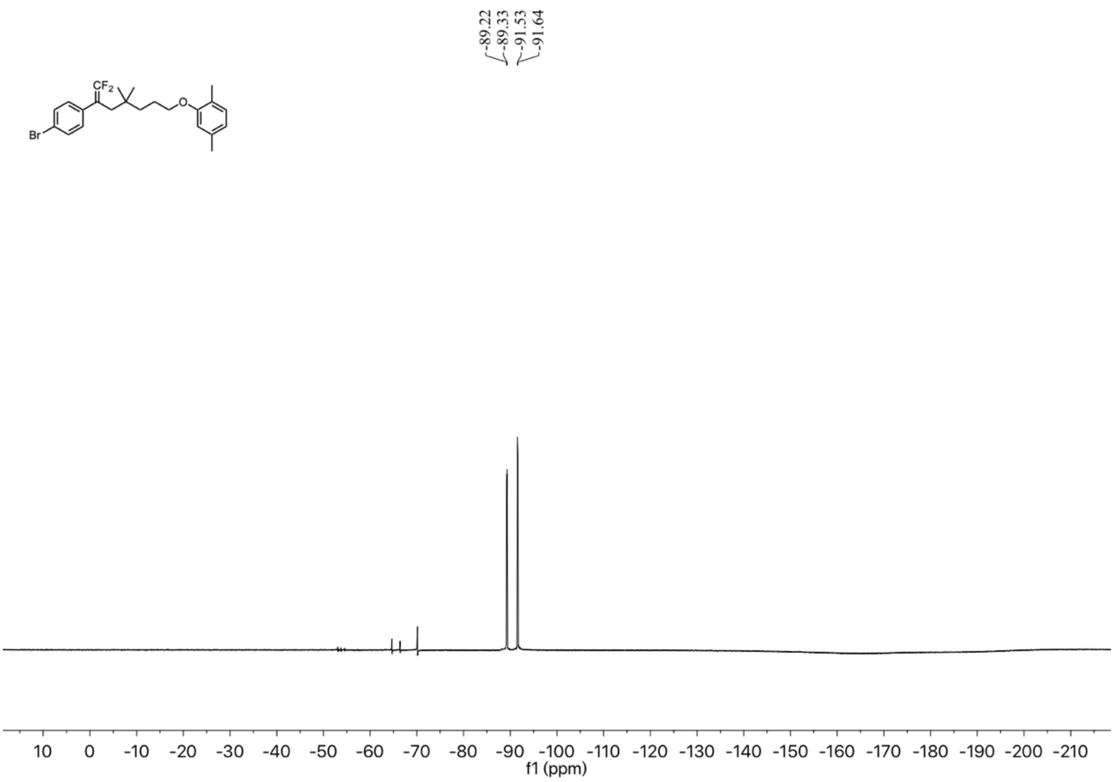


**$^{1}$H NMR (400 MHz, CDCl$_3$)** spectrum of **2-((7,7-difluoro-4,4-dimethyl-6-(4-(trifluoromethyl)phenyl)hept-6-en-1-yl)oxy)-1,4-dimethylbenzene (40)**

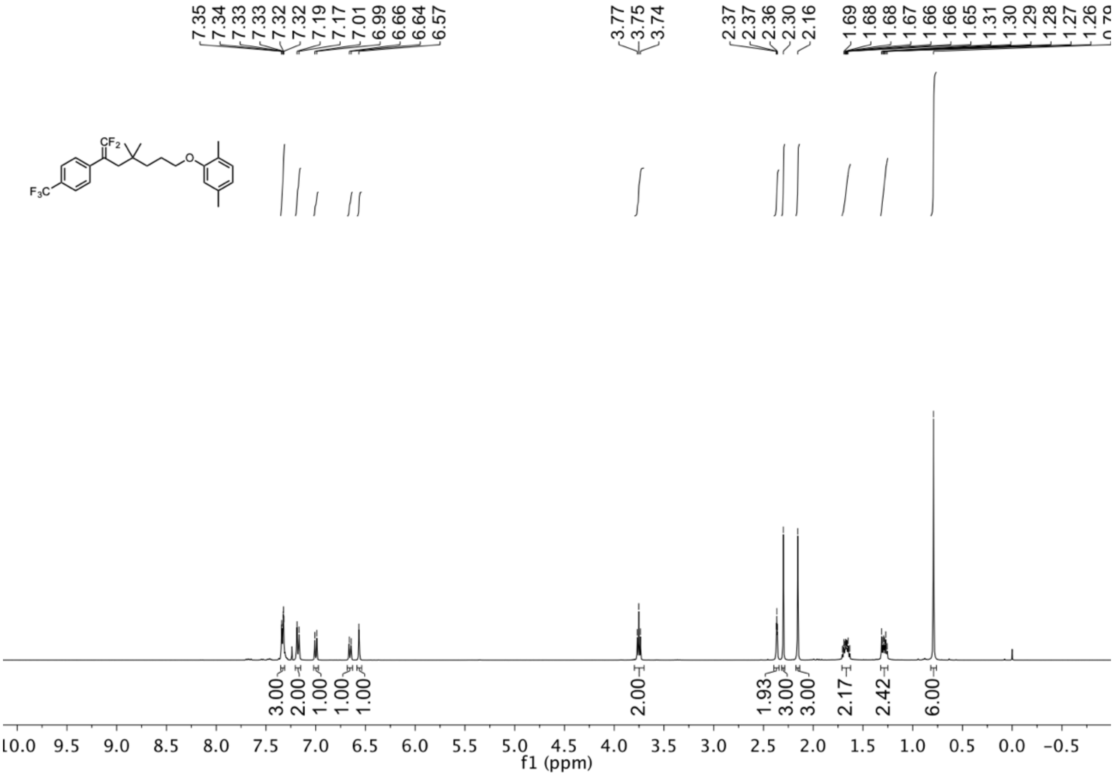

**$^{13}$C NMR (126 MHz, CDCl$_3$)** spectrum of **2-((7,7-difluoro-4,4-dimethyl-6-(4-(trifluoromethyl)phenyl)hept-6-en-1-yl)oxy)-1,4-dimethylbenzene (40)**

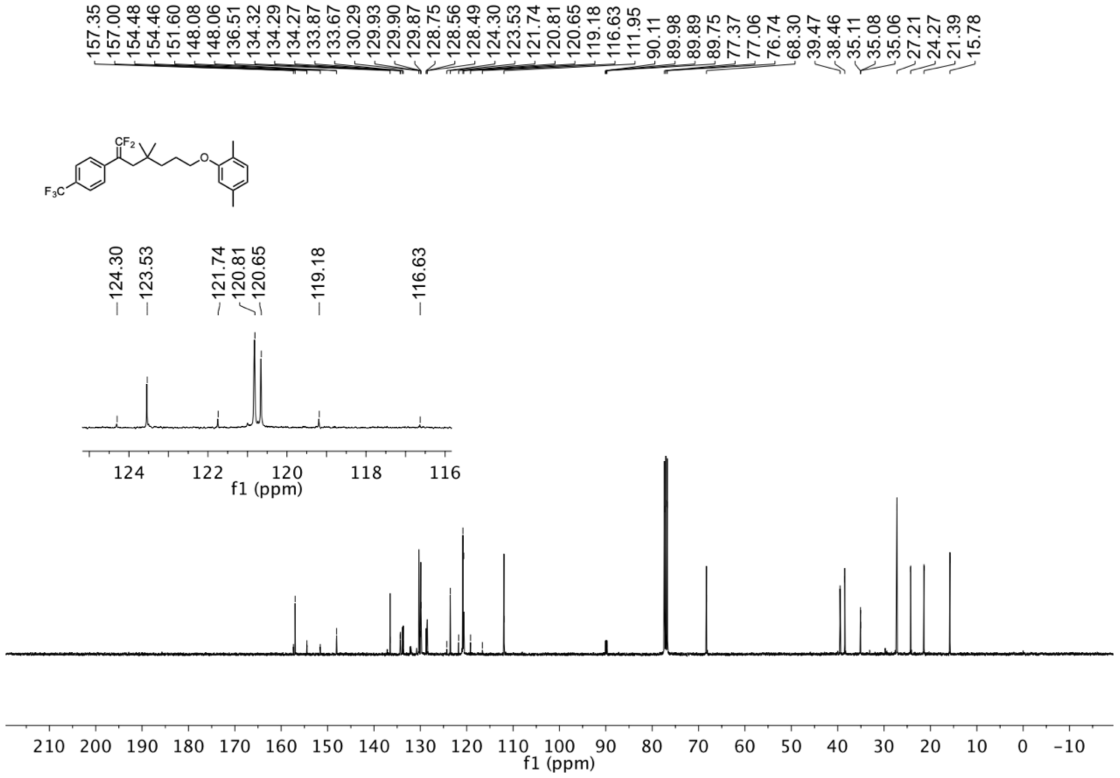

**$^{19}$F NMR (376 MHz, CDCl$_3$)** spectrum of **2-((7,7-difluoro-4,4-dimethyl-6-(4-(trifluoromethyl)phenyl)hept-6-en-1-yl)oxy)-1,4-dimethylbenzene (40)**

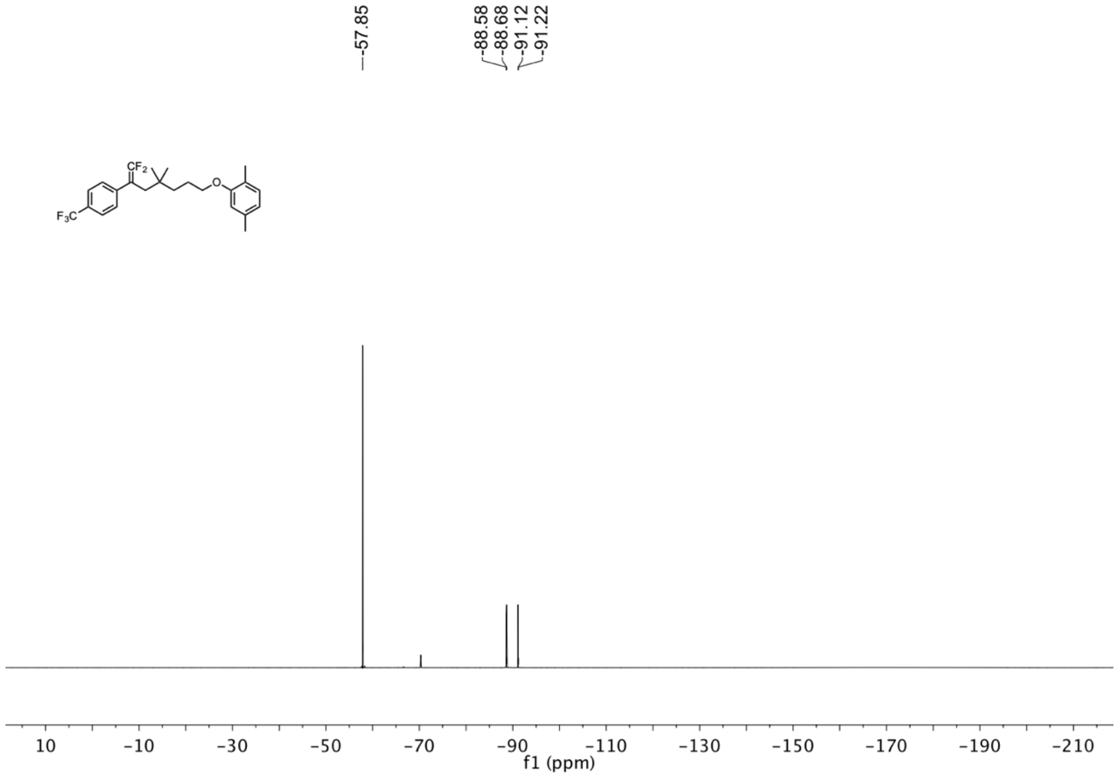

**$^{1}$H NMR (400 MHz, $CDCl_3$)** spectrum of **4-(7-(2,5-dimethylphenoxy)-1,1-difluoro-4,4-dimethylhept-1-en-2-yl)benzonitrile (41)**

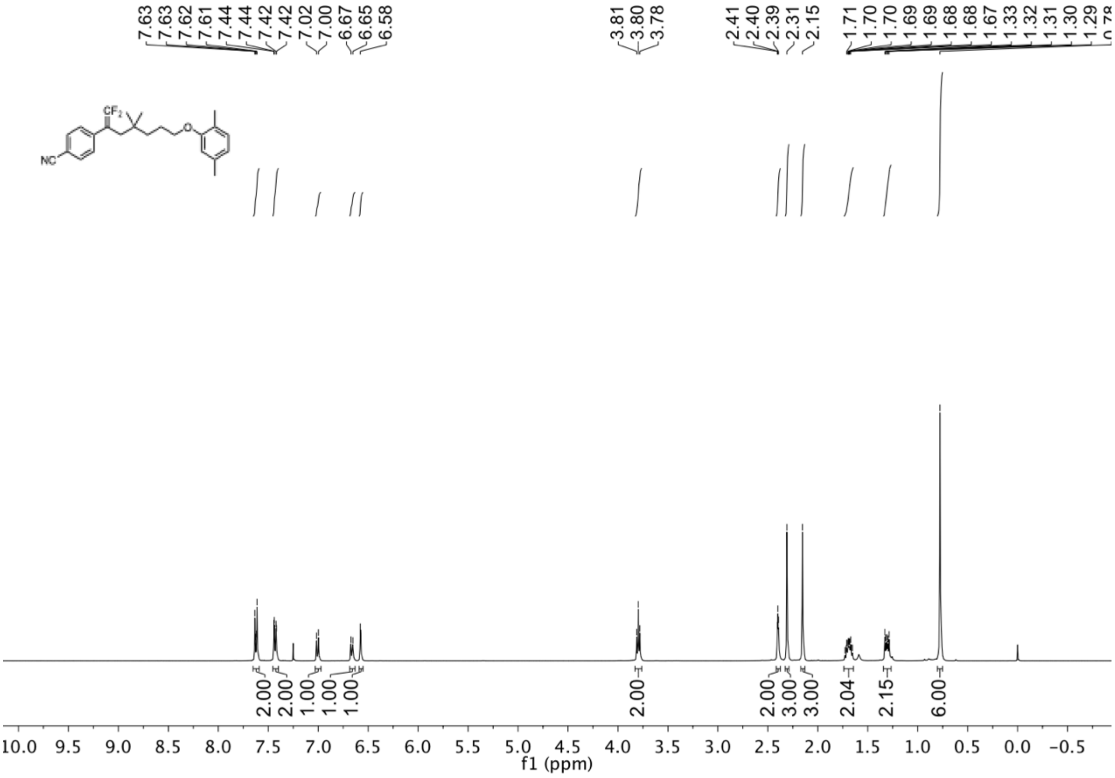


**$^{13}$C NMR (101 MHz, $CDCl_3$)** spectrum of **4-(7-(2,5-dimethylphenoxy)-1,1-difluoro-4,4-dimethylhept-1-en-2-yl)benzonitrile (41)**

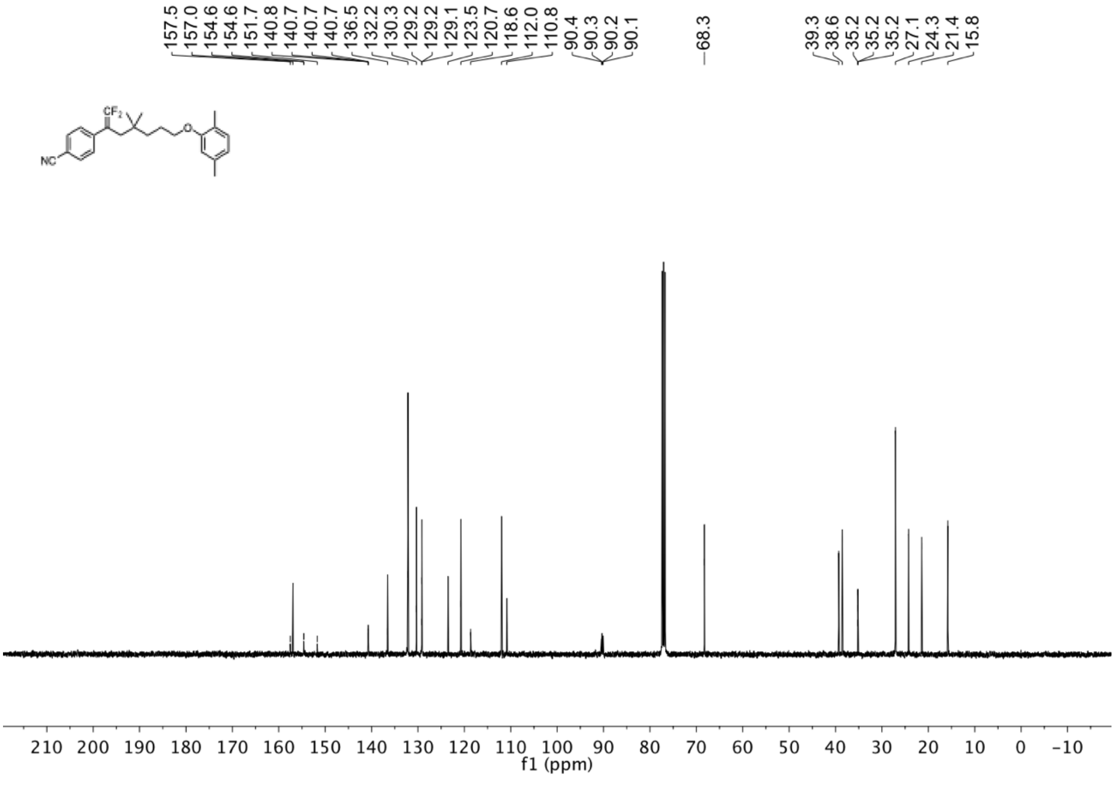

**$^{19}F$ NMR (376 MHz, $CDCl_3$)** spectrum of **4-(7-(2,5-dimethylphenoxy)-1,1-difluoro-4,4-dimethylhept-1-en-2-yl)benzonitrile (41)**

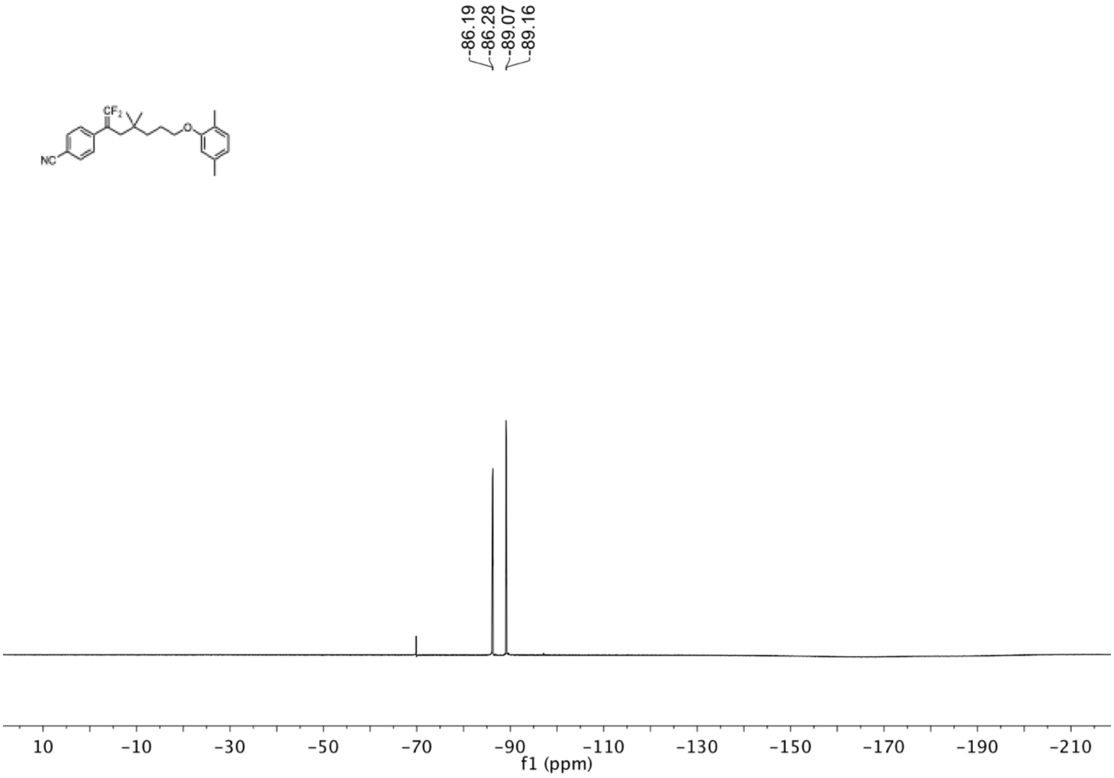


**$^{1}H$ NMR (400 MHz, $CDCl_3$)** spectrum of **ethyl 4-(7-(2,5-dimethylphenoxy)-1,1-difluoro-4,4-dimethylhept-1-en-2-yl)benzoate (42)**

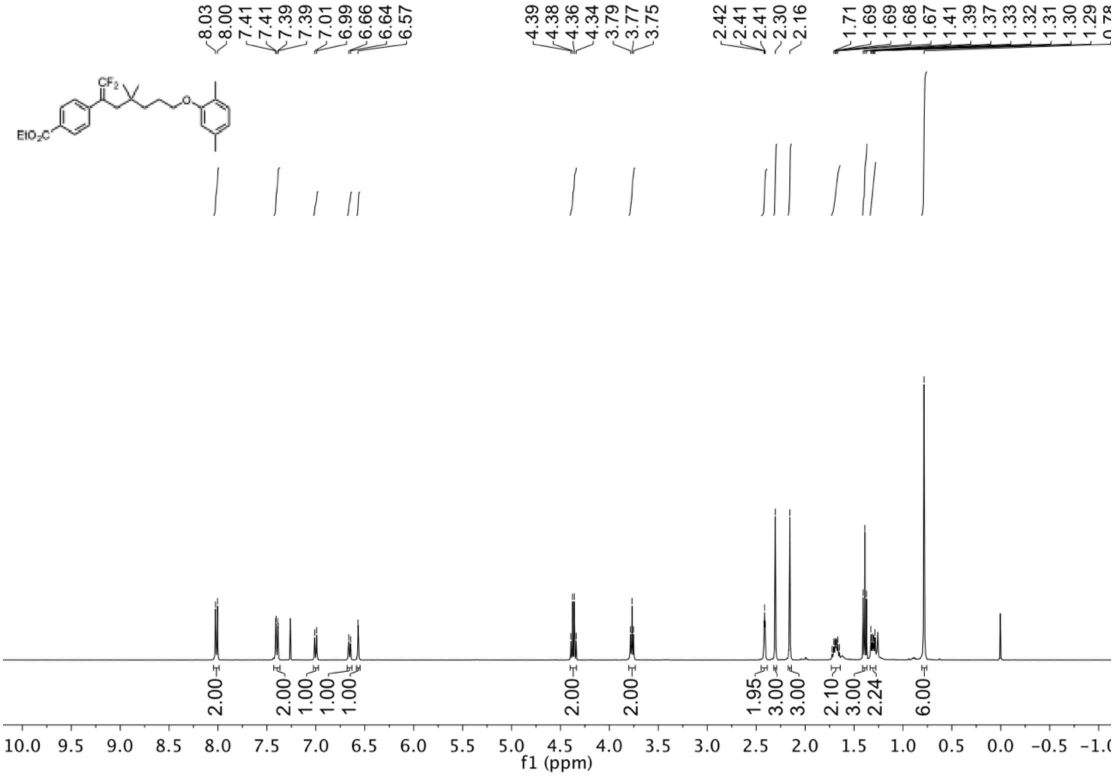

**$^{13}C$ NMR (101 MHz, $CDCl_3$)** spectrum of **ethyl 4-(7-(2,5-dimethylphenoxy)-1,1-difluoro-4,4-dimethylhept-1-en-2-yl)benzoate (42)**

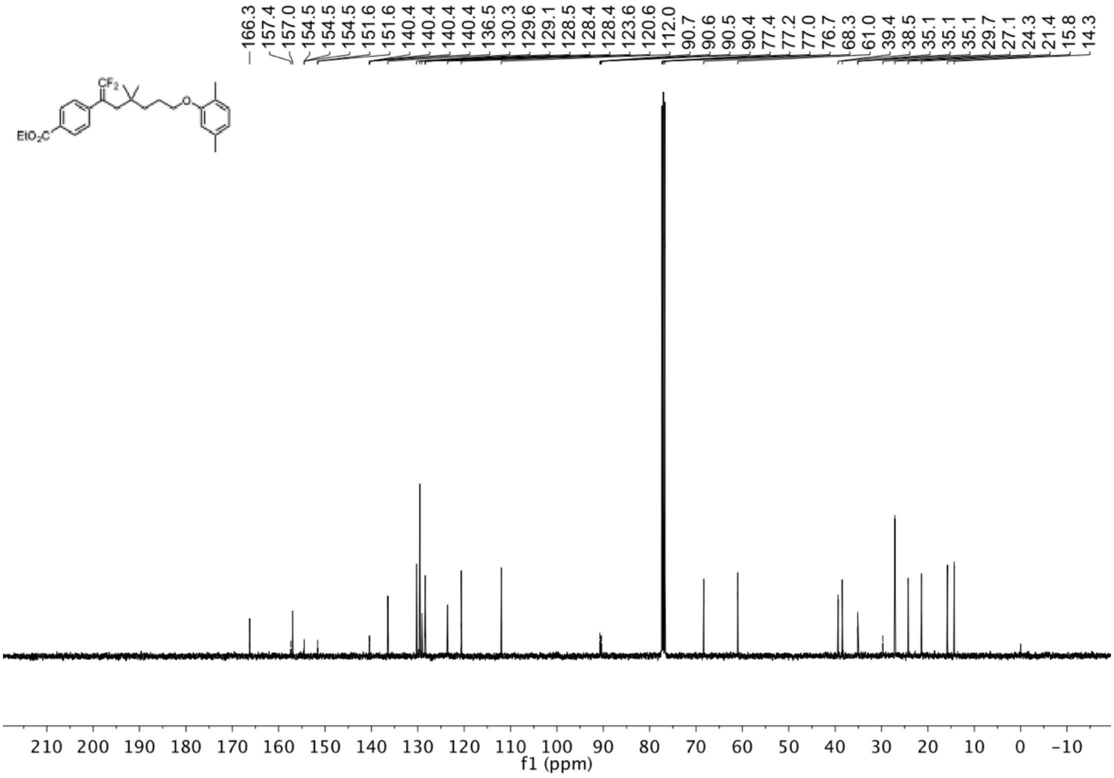


**$^{19}F$ NMR (376 MHz, $CDCl_3$)** spectrum of **ethyl 4-(7-(2,5-dimethylphenoxy)-1,1-difluoro-4,4-dimethylhept-1-en-2-yl)benzoate (42)**

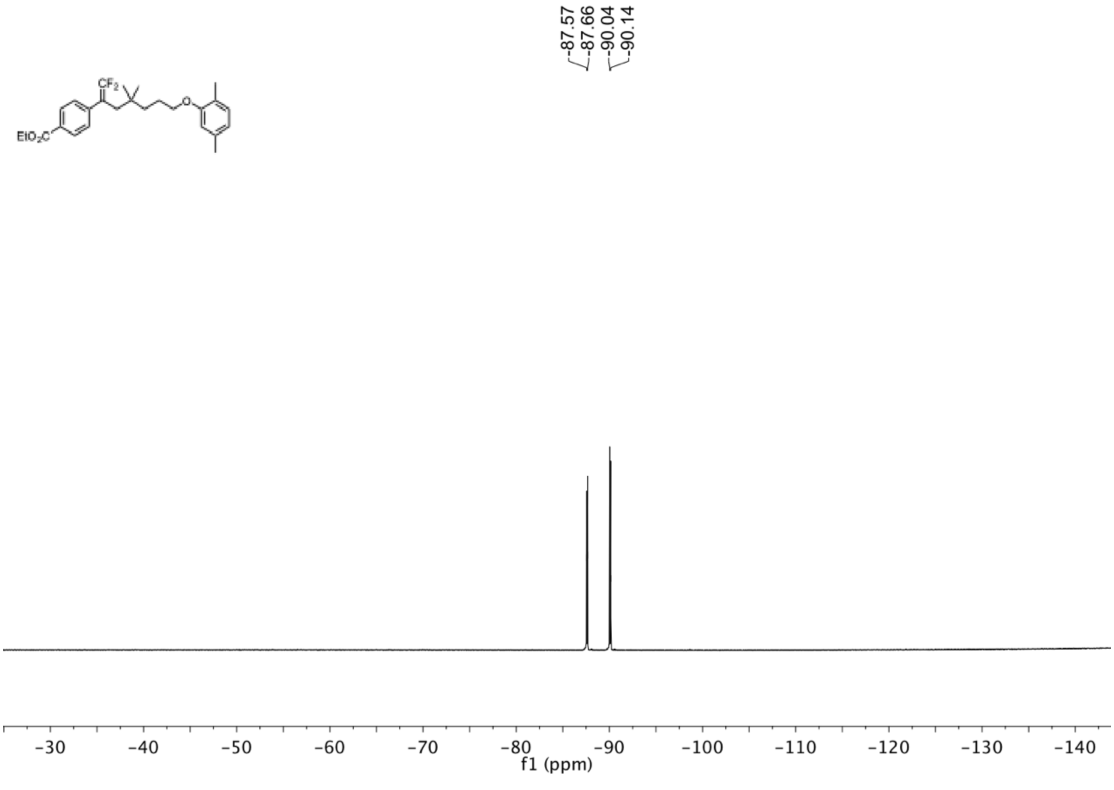

**$^{1}$H NMR (400 MHz, $CDCl_3$)** spectrum of **2-((6-(3,5-dimethoxyphenyl)-7,7-difluoro-4,4-dimethylhept-6-en-1-yl)oxy)-1,4-dimethylbenzene (43)**

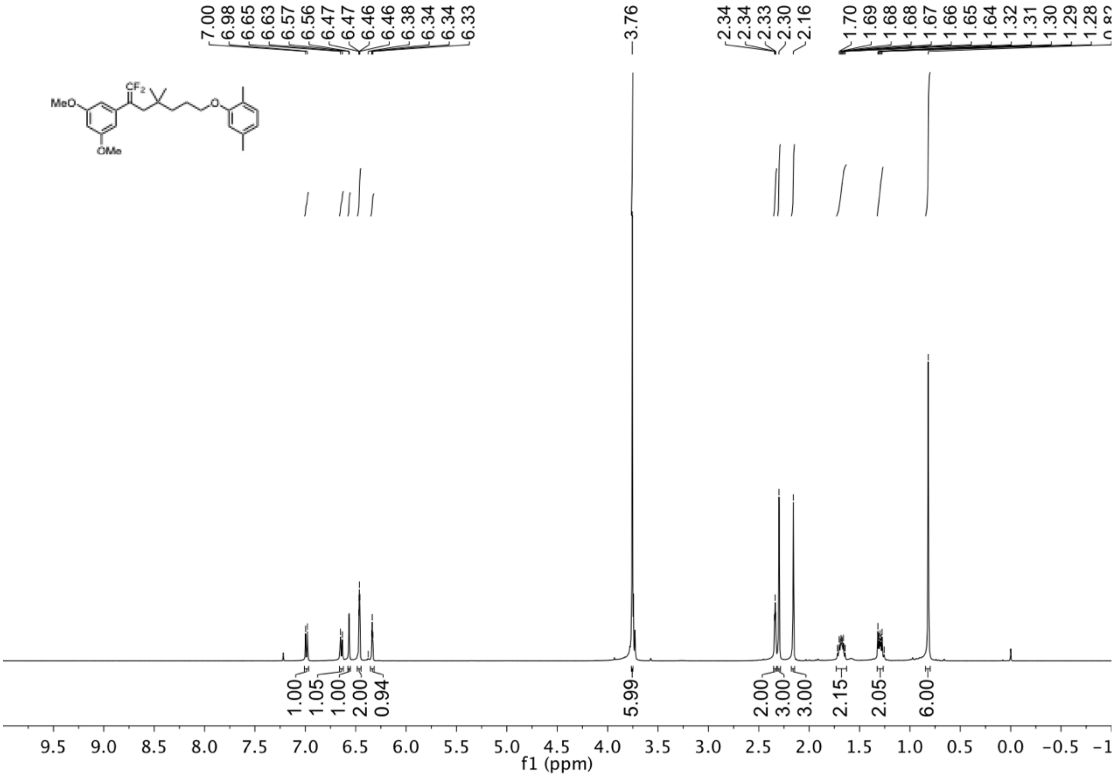


**$^{13}$C NMR (101 MHz, $CDCl_3$)** spectrum of **2-((6-(3,5-dimethoxyphenyl)-7,7-difluoro-4,4-dimethylhept-6-en-1-yl)oxy)-1,4-dimethylbenzene (43)**

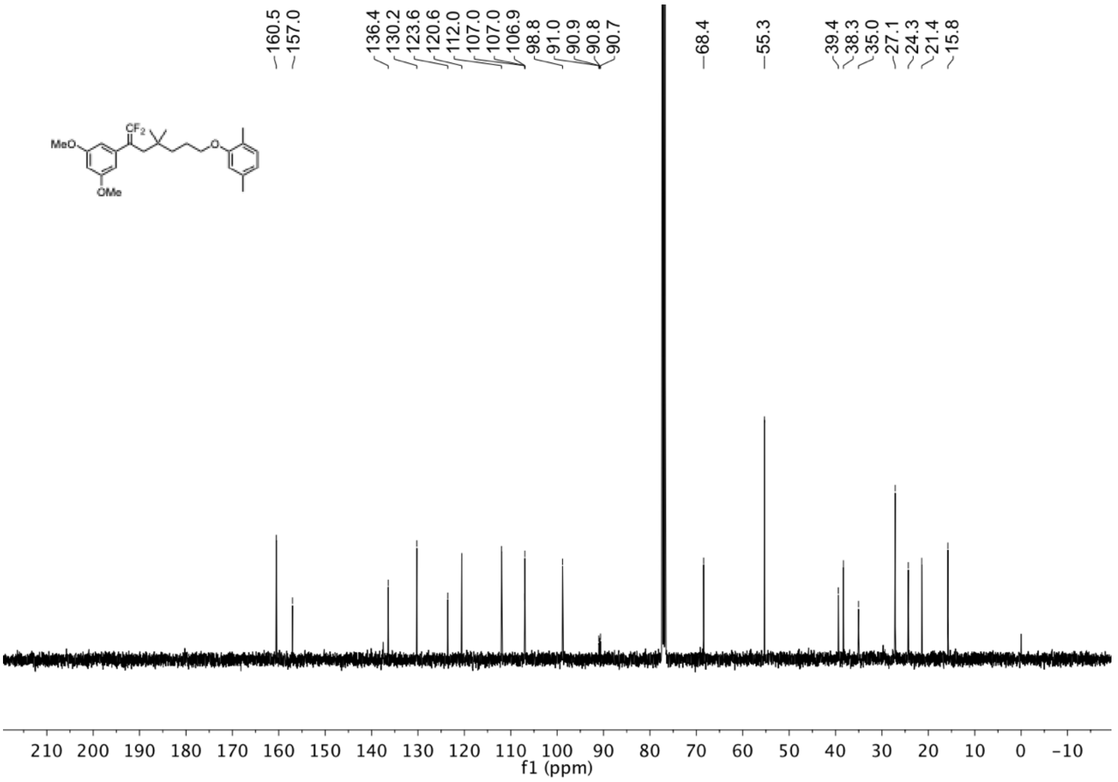

**$^{19}$F NMR (376 MHz, CDCl$_3$)** spectrum of **2-((6-(3,5-dimethoxyphenyl)-7,7-difluoro-4,4-dimethylhept-6-en-1-yl)oxy)-1,4-dimethylbenzene (43)**

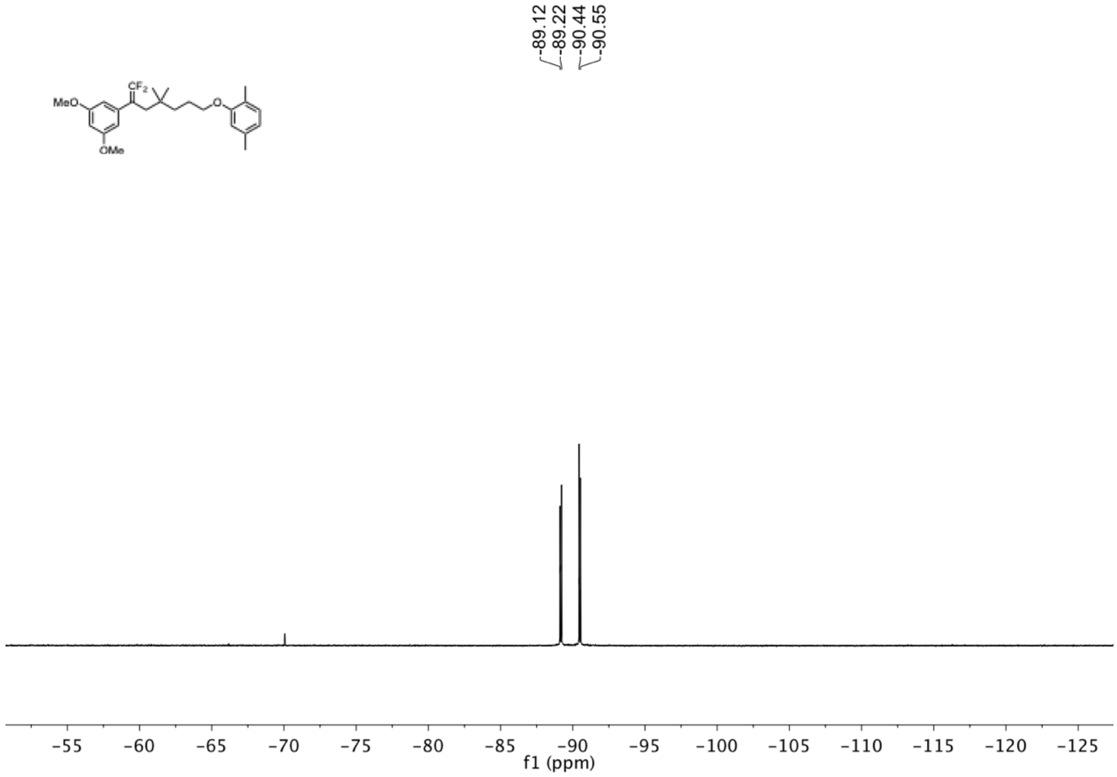


**$^{1}$H NMR (400 MHz, CDCl$_3$)** spectrum of **2-(7-(2,5-dimethylphenoxy)-1,1-difluoro-4,4-dimethylhept-1-en-2-yl)naphthalene (44)**

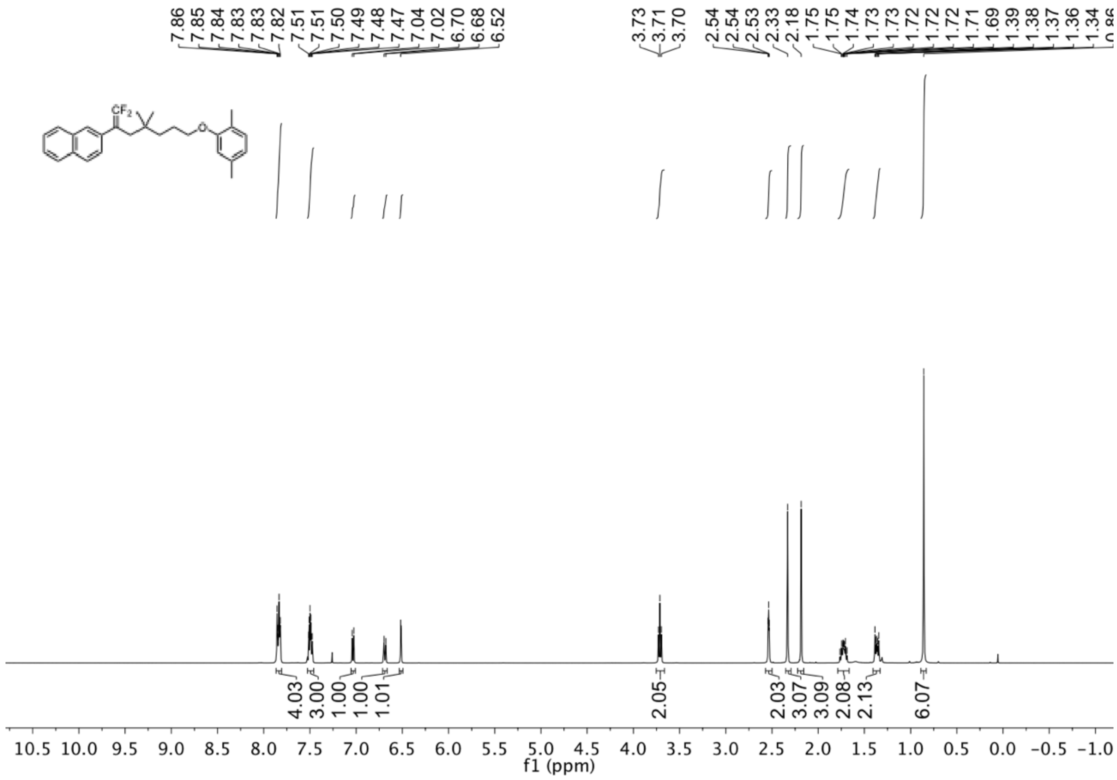

**$^{13}$C NMR (126 MHz, CDCl$_3$)** spectrum of **2-(7-(2,5-dimethylphenoxy)-1,1-difluoro-4,4-dimethylhept-1-en-2-yl)naphthalene (44)**

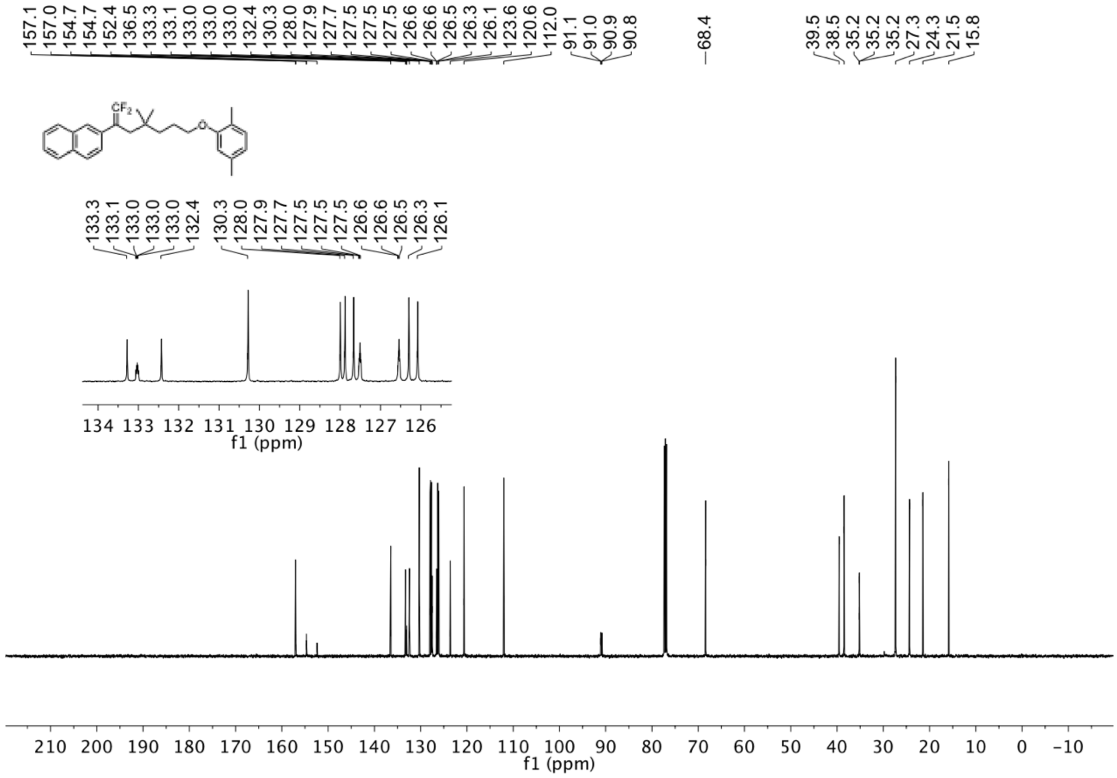


**$^{19}$F NMR (376 MHz, CDCl$_3$)** spectrum of **2-(7-(2,5-dimethylphenoxy)-1,1-difluoro-4,4-dimethylhept-1-en-2-yl)naphthalene (44)**

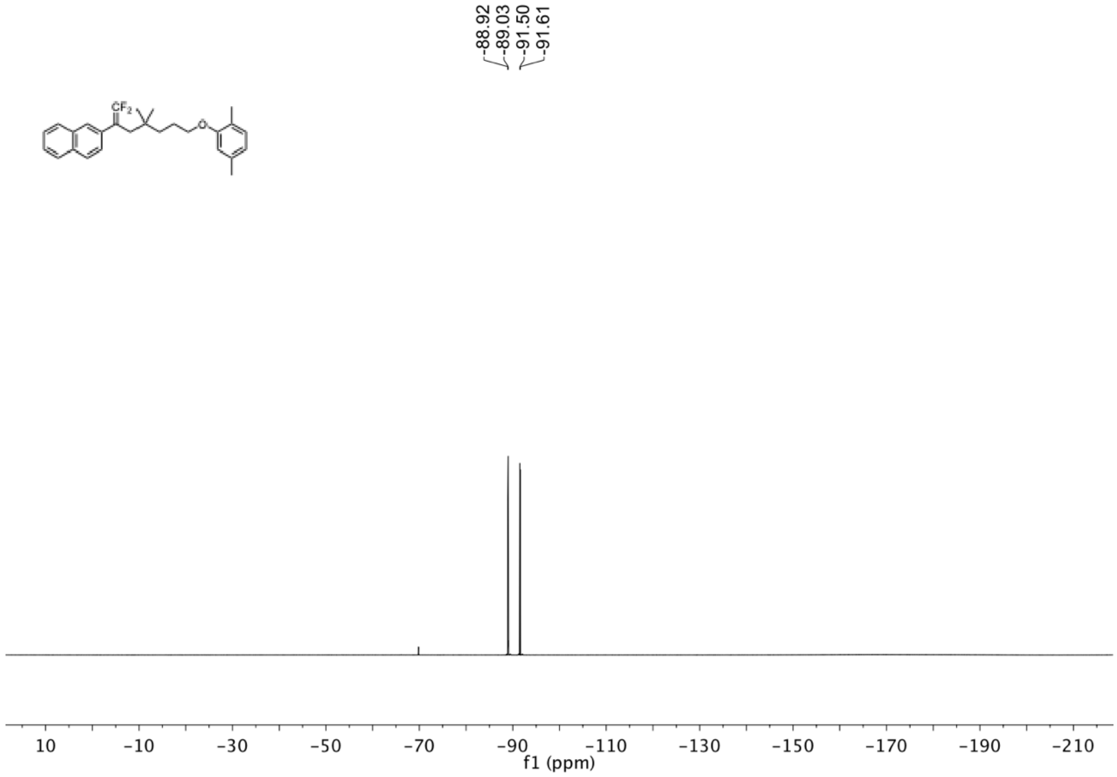

**$^{1}$H NMR (400 MHz, CDCl$_3$)** spectrum of **5-(7-(2,5-dimethylphenoxy)-1,1-difluoro-4,4-dimethylhept-1-en-2-yl)benzo[*d*][1,3]dioxole (45)**

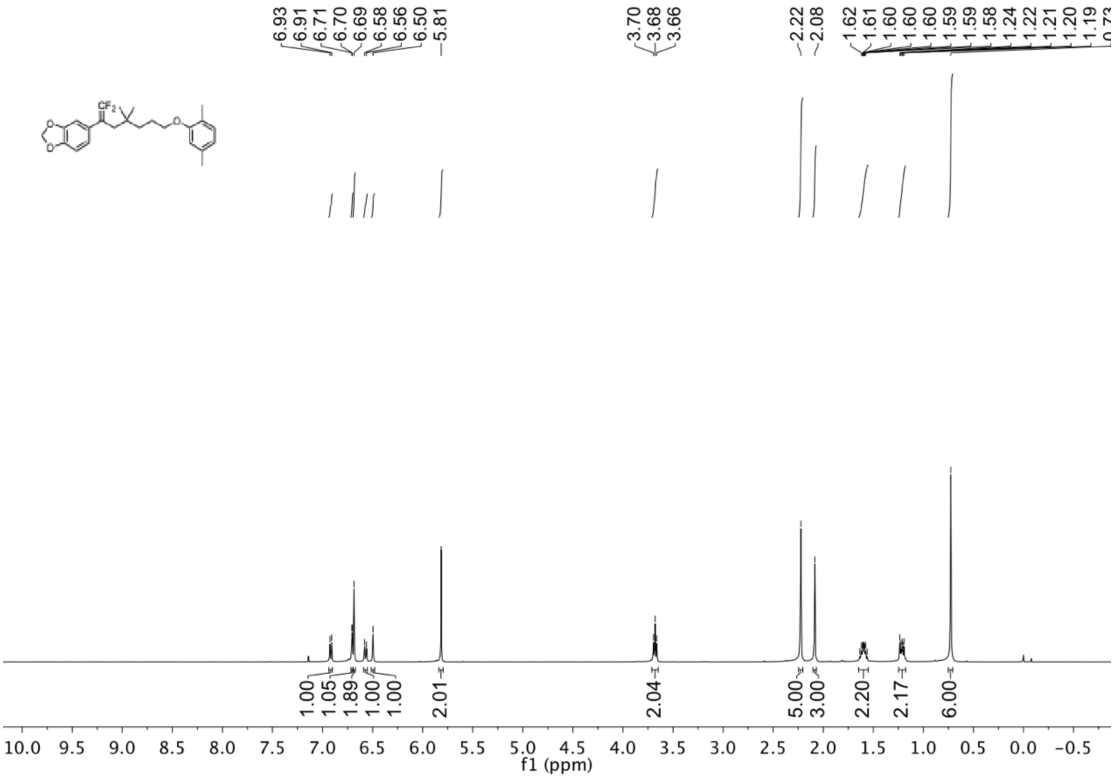


**$^{13}$C NMR (101 MHz, CDCl$_3$)** spectrum of **5-(7-(2,5-dimethylphenoxy)-1,1-difluoro-4,4-dimethylhept-1-en-2-yl)benzo[*d*][1,3]dioxole (45)**

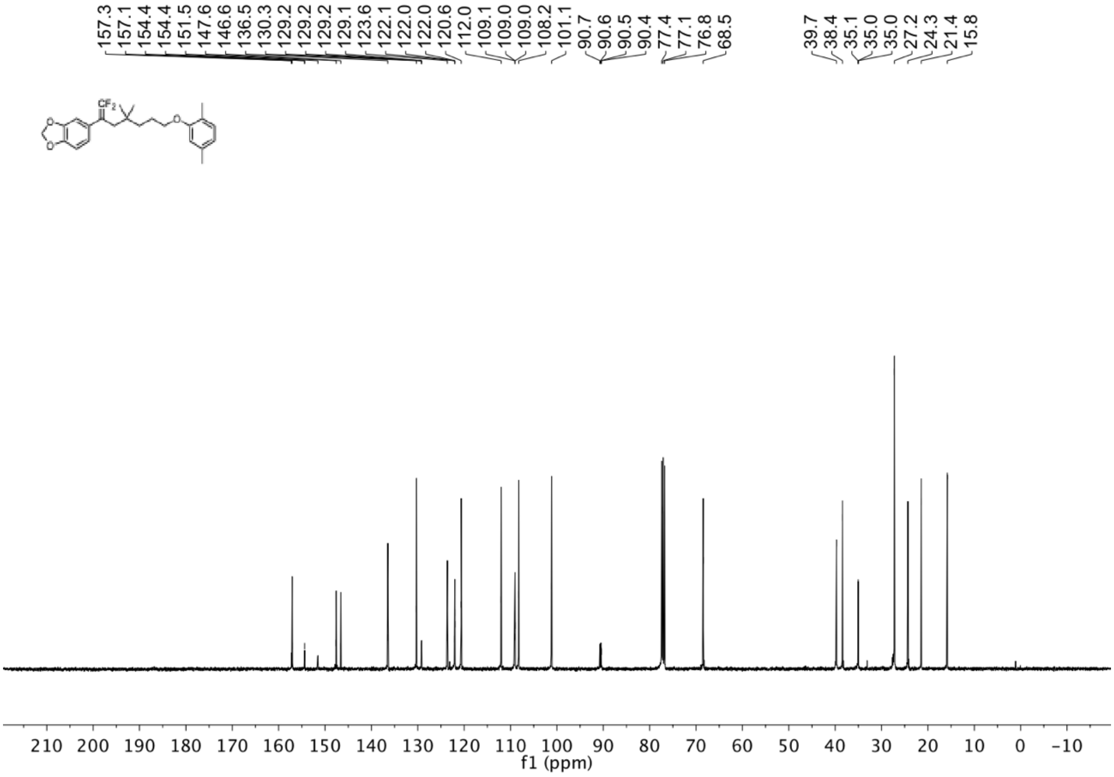

**$^{19}$F NMR (376 MHz, CDCl$_3$)** spectrum of **5-(7-(2,5-dimethylphenoxy)-1,1-difluoro-4,4-dimethylhept-1-en-2-yl)benzo[*d*][1,3]dioxole (45)**

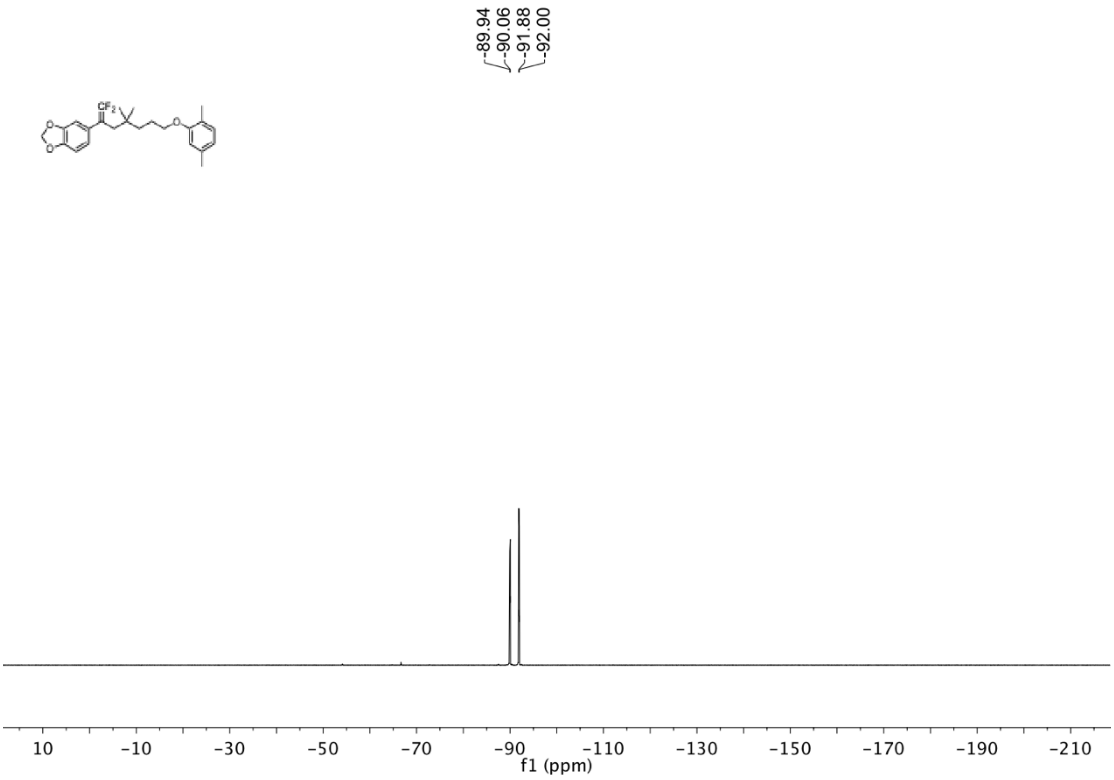

**$^{1}$H NMR (400 MHz, CDCl$_3$)** spectrum of **5-(7-(2,5-dimethylphenoxy)-1,1-difluoro-4,4-dimethylhept-1-en-2-yl)benzo[*d*][1,3]dioxole (46)**

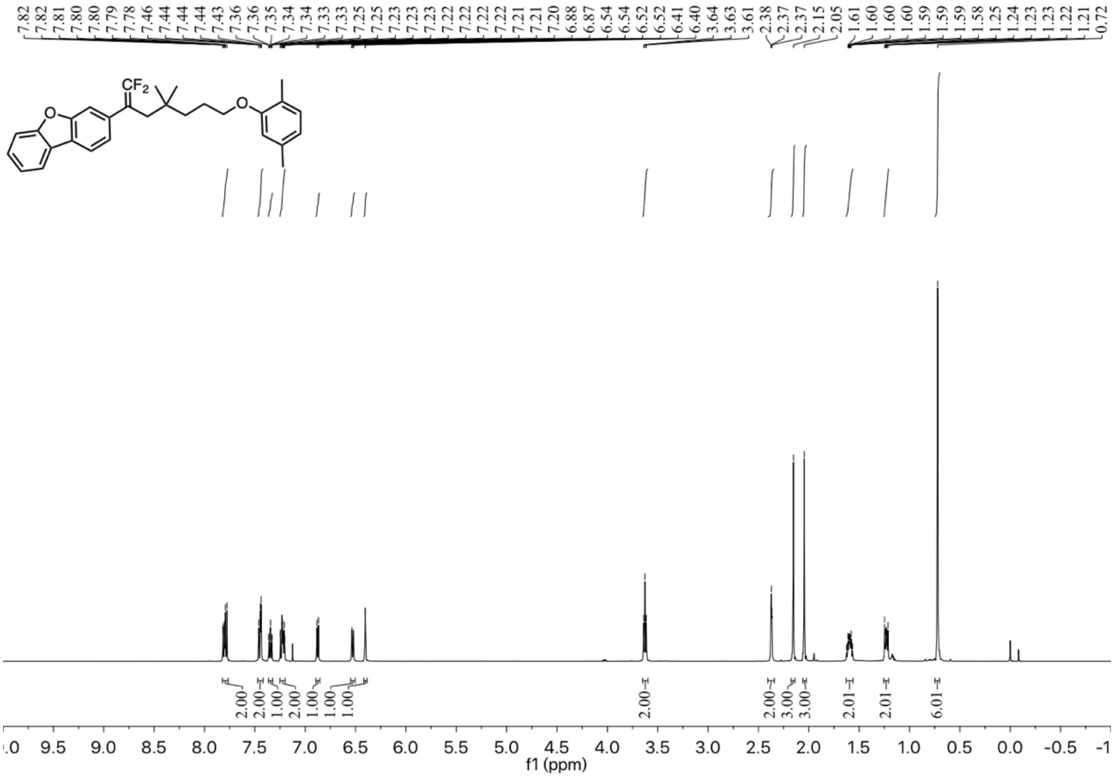

**$^{13}C$ NMR (101 MHz, $CDCl_3$)** spectrum of **5-(7-(2,5-dimethylphenoxy)-1,1-difluoro-4,4-dimethylhept-1-en-2-yl)benzo[*d*][1,3]dioxole (46)**

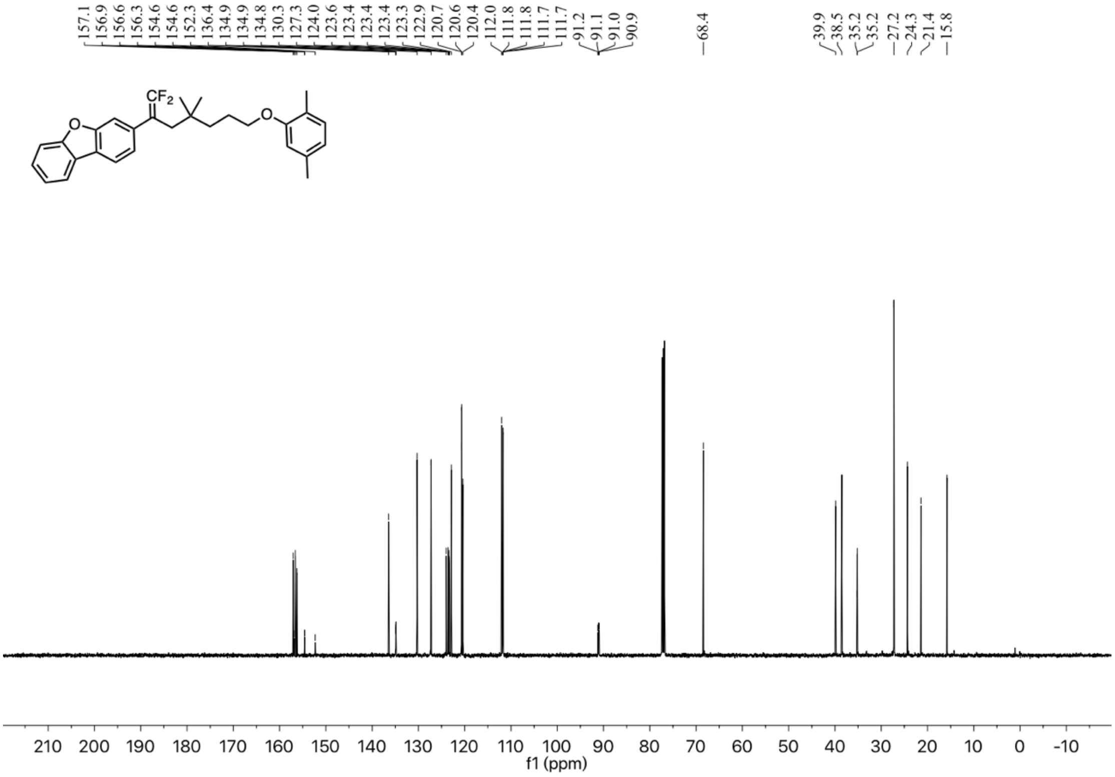

**$^{19}F$ NMR (376 MHz, $CDCl_3$)** spectrum of **5-(7-(2,5-dimethylphenoxy)-1,1-difluoro-4,4-dimethylhept-1-en-2-yl)benzo[*d*][1,3]dioxole (46)**

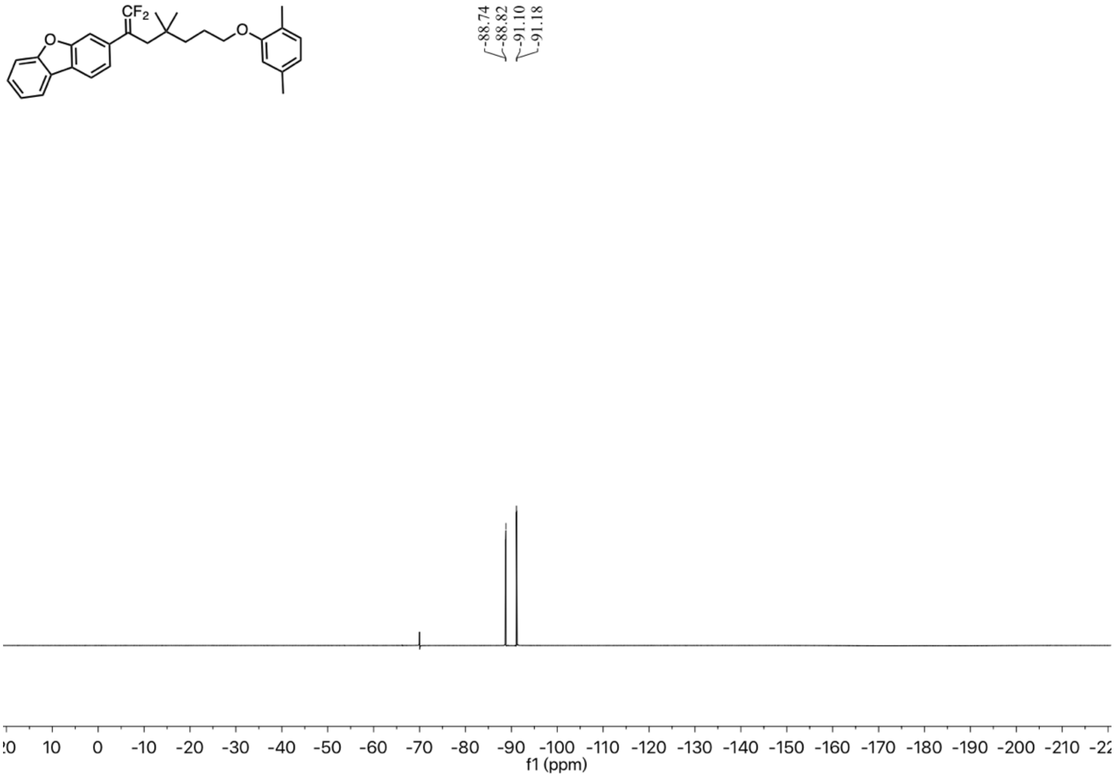

**$^{1}$H NMR (400 MHz, CDCl$_3$)** spectrum of **4-(7-(2,5-dimethylphenoxy)-1,1-difluoro-4,4-dimethylhept-1-en-2-yl)dibenzo[*b*,*d*]thiophene (47)**

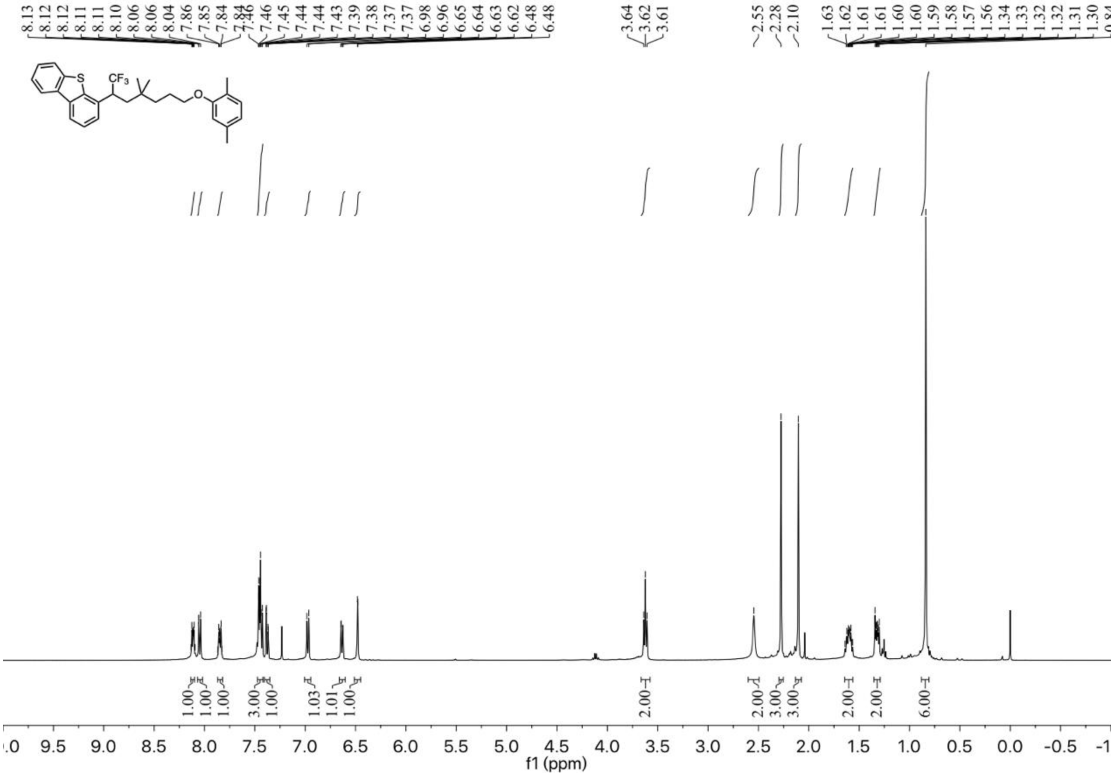


**$^{13}$C NMR (151 MHz, CDCl$_3$)** spectrum of **4-(7-(2,5-dimethylphenoxy)-1,1-difluoro-4,4-dimethylhept-1-en-2-yl)dibenzo[*b*,*d*]thiophene (47)**

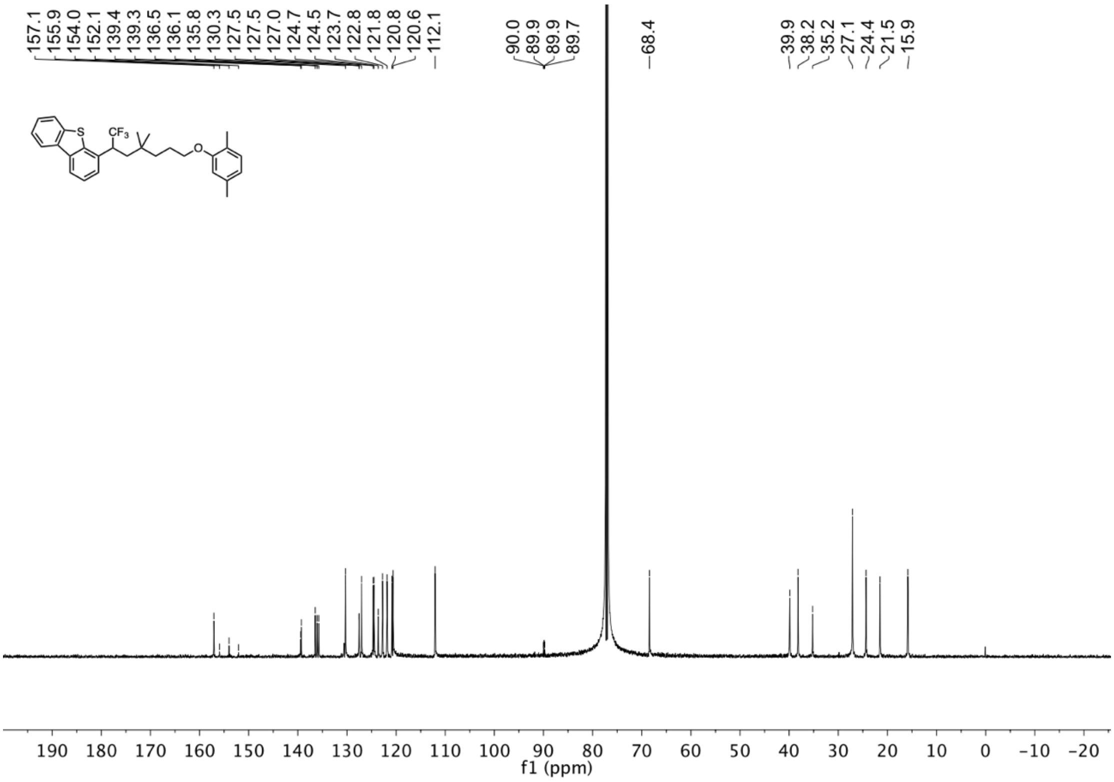

**$^{19}$F NMR (376 MHz, CDCl$_3$)** spectrum of **4-(7-(2,5-dimethylphenoxy)-1,1-difluoro-4,4-dimethylhept-1-en-2-yl)dibenzo[*b*,*d*]thiophene (47)**

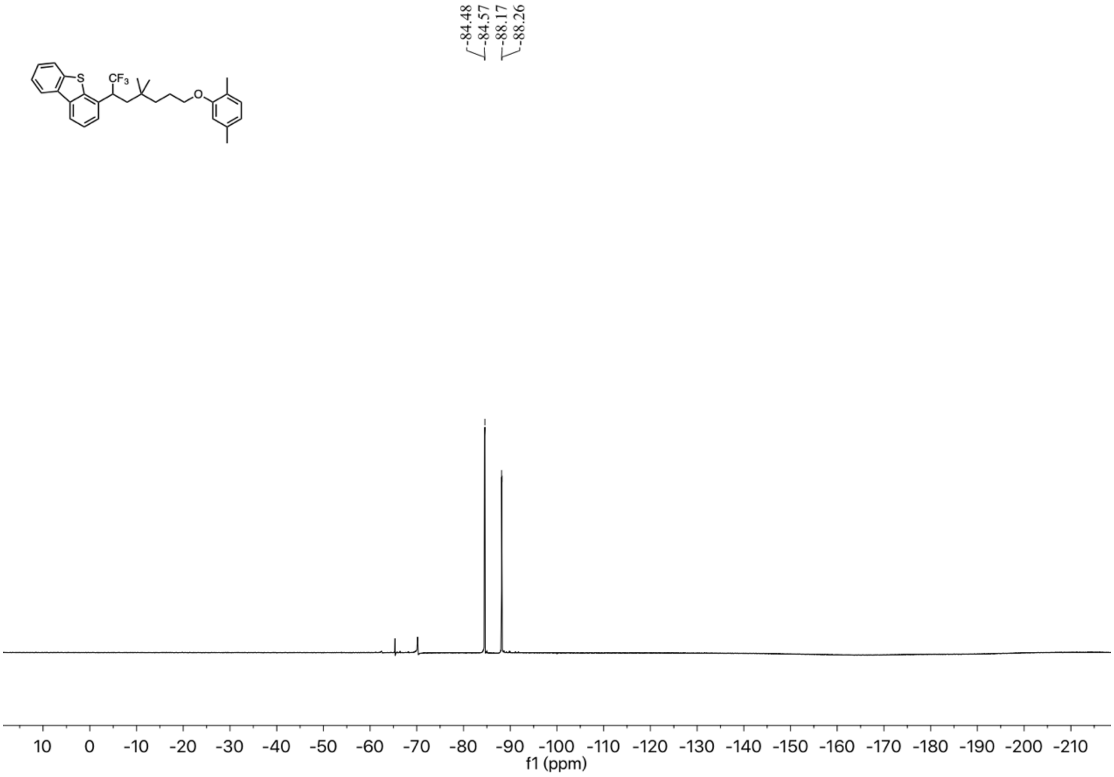


**$^{1}$H NMR (500 MHz, CDCl$_3$)** spectrum of **2-(7-(2,5-dimethylphenoxy)-1,1-difluoro-4,4-dimethylhept-1-en-2-yl)benzo[*b*]thiophene (48)**

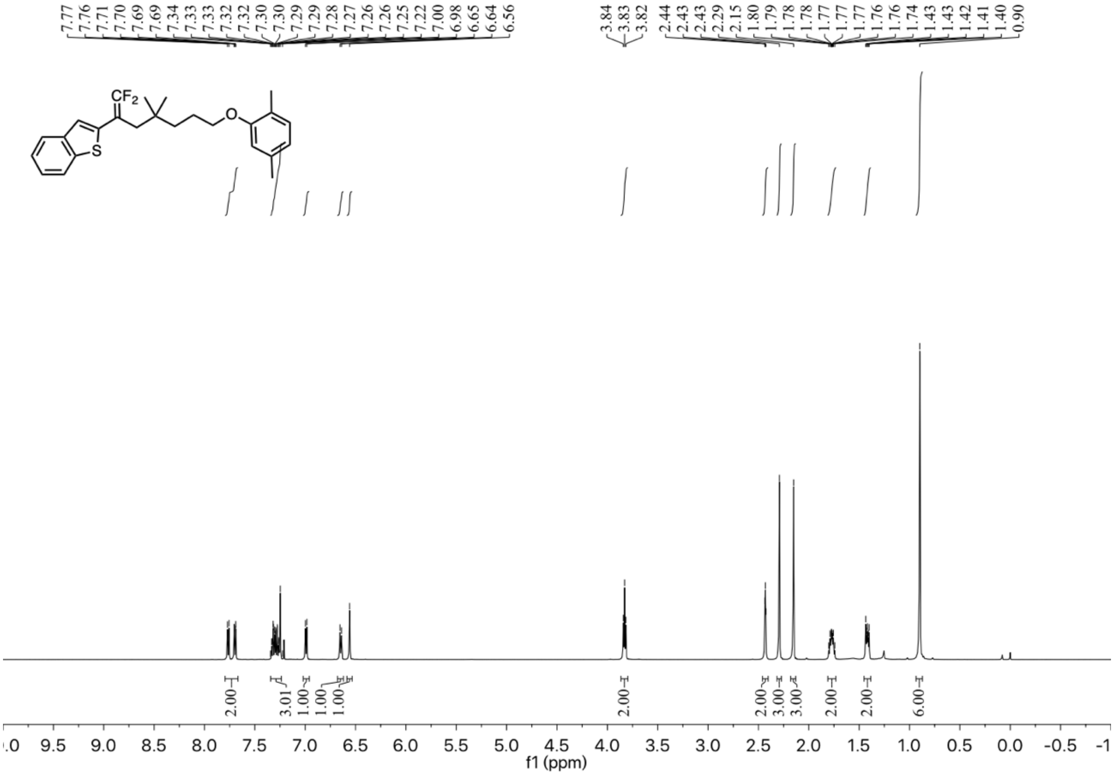

**$^{13}$C NMR (126 MHz, CDCl$_3$)** spectrum of **2-(7-(2,5-dimethylphenoxy)-1,1-difluoro-4,4-dimethylhept-1-en-2-yl)benzo[*b*]thiophene (48)**

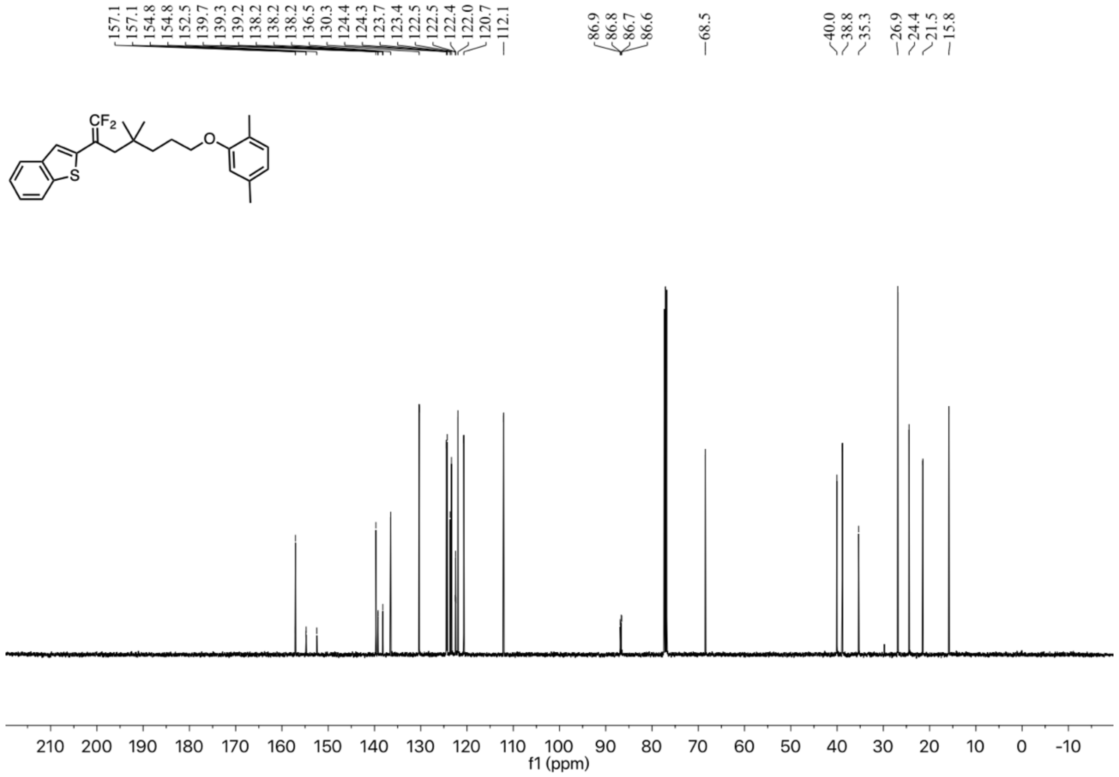

**$^{19}$F NMR (471 MHz, CDCl$_3$)** spectrum of **2-(7-(2,5-dimethylphenoxy)-1,1-difluoro-4,4-dimethylhept-1-en-2-yl)benzo[*b*]thiophene (48)**

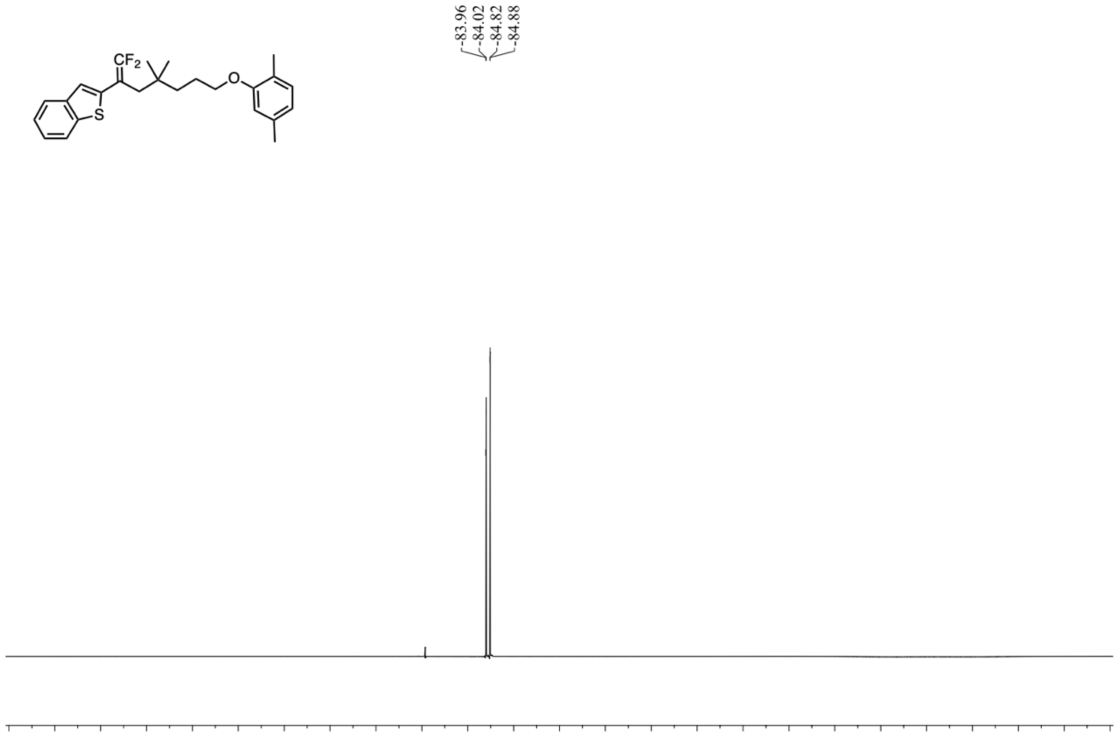

$^{1}$H NMR (400 MHz, $CDCl_3$) spectrum of **4-(1,1-difluoro-3-(1-methylcyclohexyl)prop-1-en-2-yl)-1,1'-biphenyl (49)**

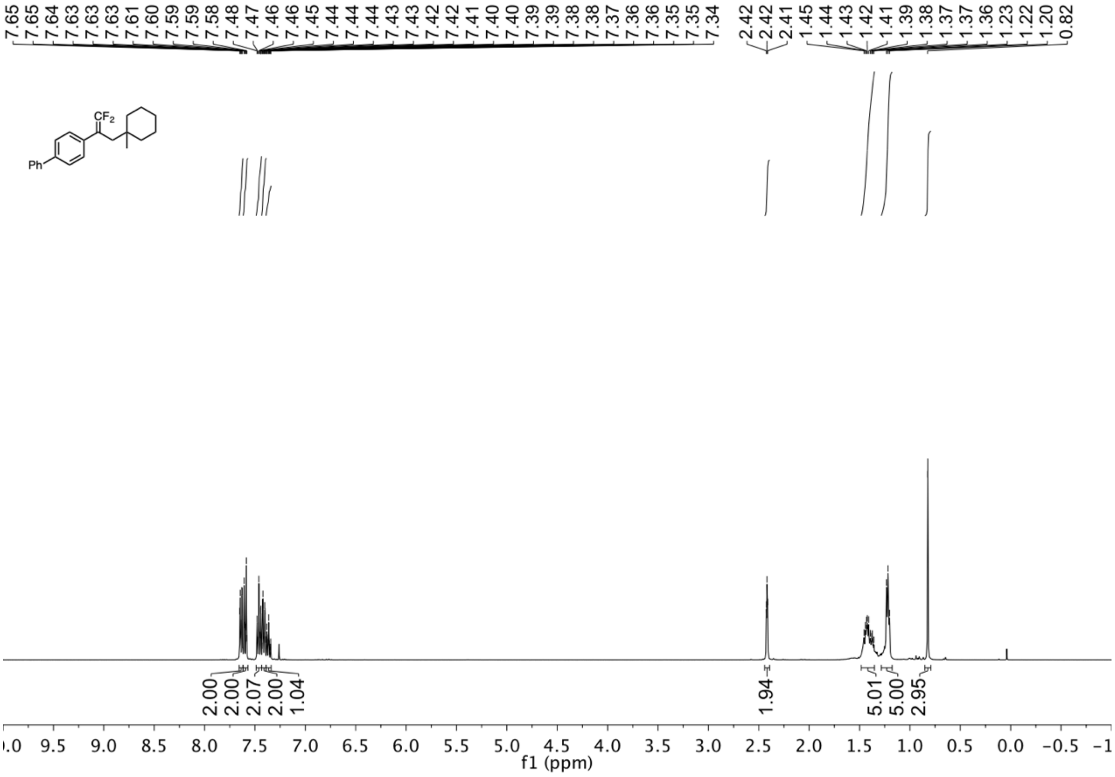


$^{13}$C NMR (101 MHz, $CDCl_3$) spectrum of **4-(1,1-difluoro-3-(1-methylcyclohexyl)prop-1-en-2-yl)-1,1'-biphenyl (49)**

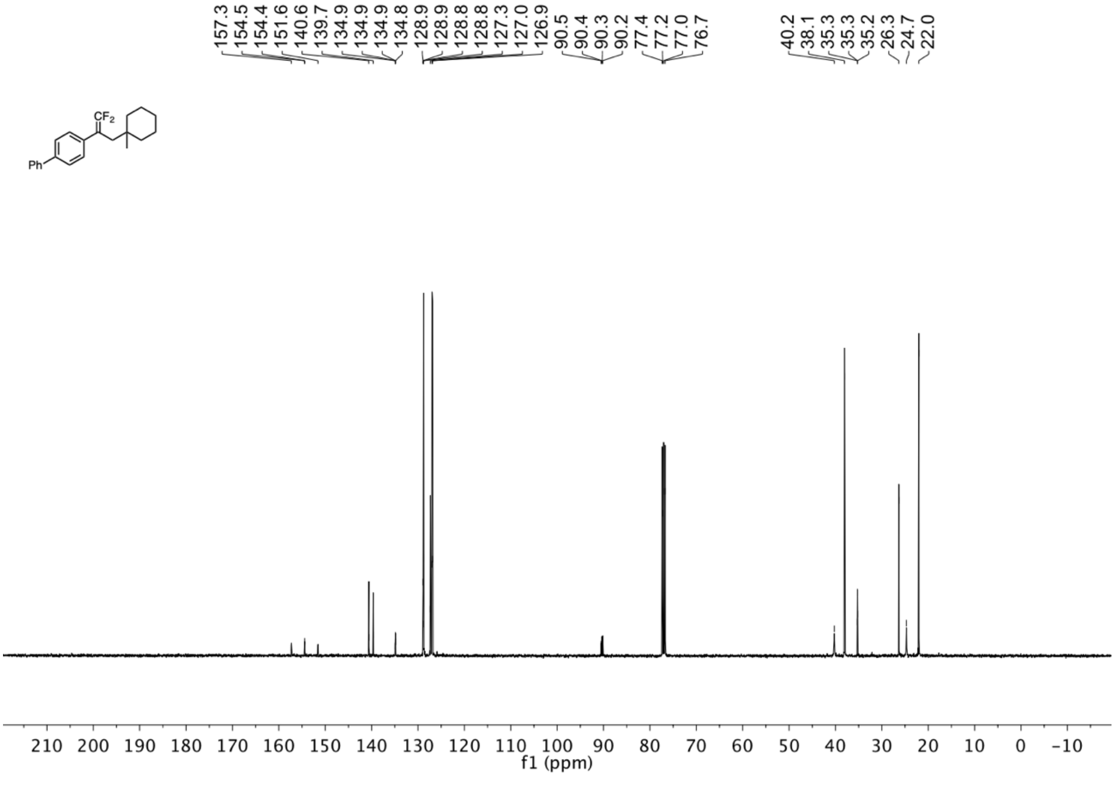

**$^{19}$F NMR (376 MHz, $CDCl_3$)** spectrum of **4-(1,1-difluoro-3-(1-methylcyclohexyl)prop-1-en-2-yl)-1,1'-biphenyl (49)**

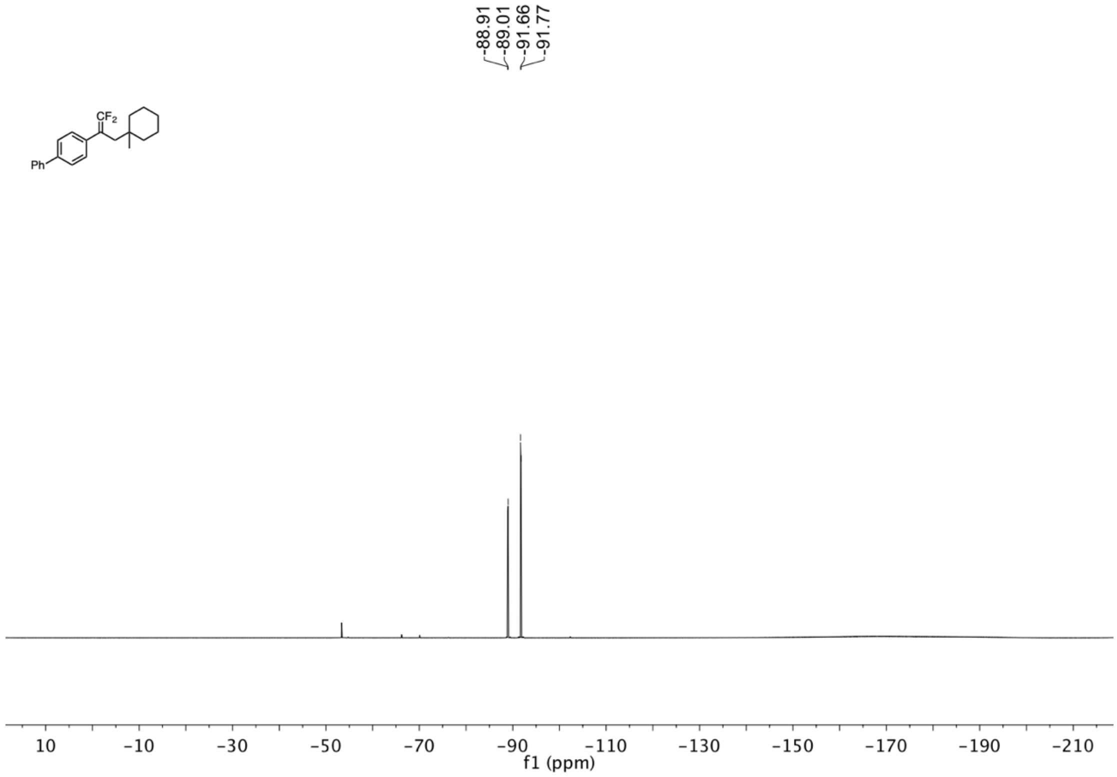


**$^{1}$H NMR (400 MHz, $CDCl_3$)** spectrum of **(3*r*,5*r*,7*r*)-1-(2-([1,1'-biphenyl]-4-yl)-3,3-difluoroallyl)adamantane (50)**

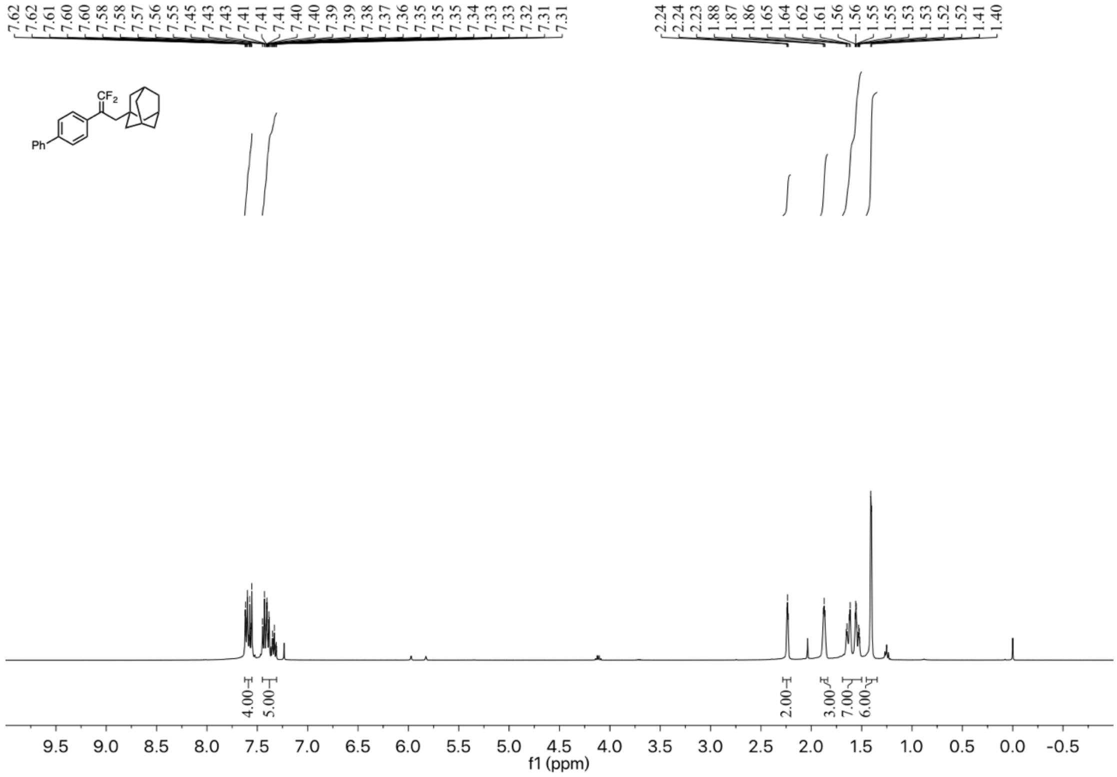

**$^{13}$C NMR (101 MHz, CDCl$_3$)** spectrum of **(3*r*,5*r*,7*r*)-1-(2-([1,1'-biphenyl]-4-yl)-3,3-difluoroallyl)adamantane (50)**

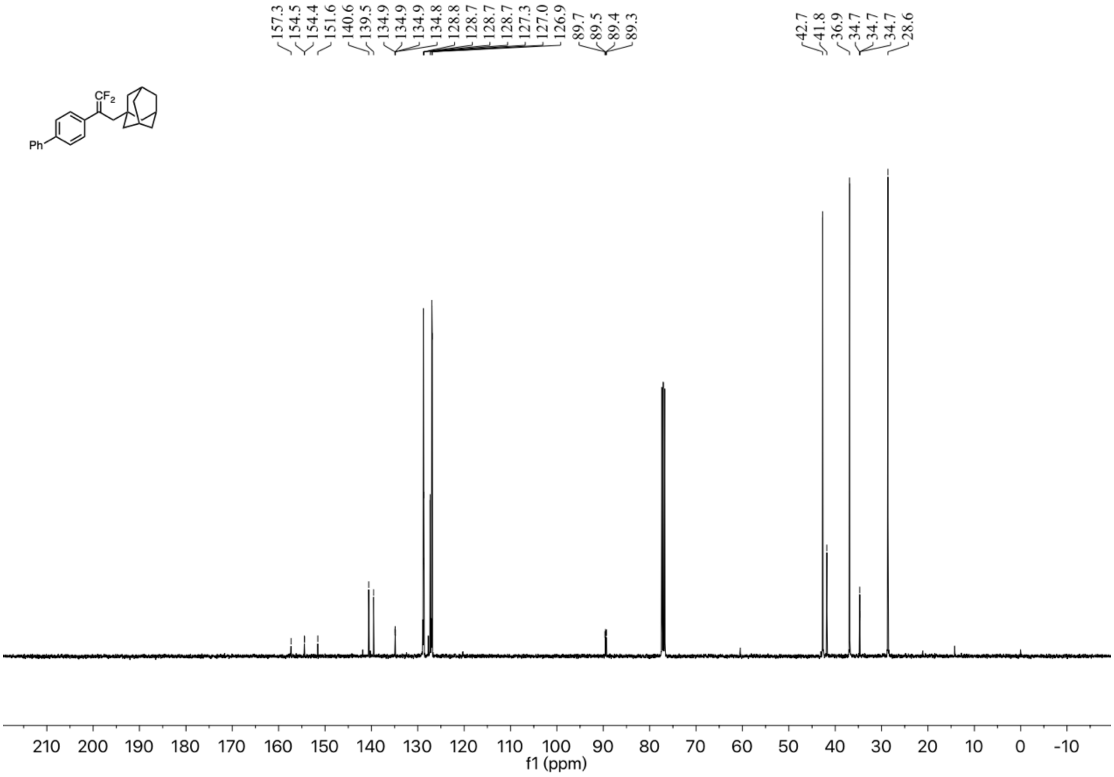


**$^{19}$F NMR (376 MHz, CDCl$_3$)** spectrum of **(3*r*,5*r*,7*r*)-1-(2-([1,1'-biphenyl]-4-yl)-3,3-difluoroallyl)adamantane (50)**

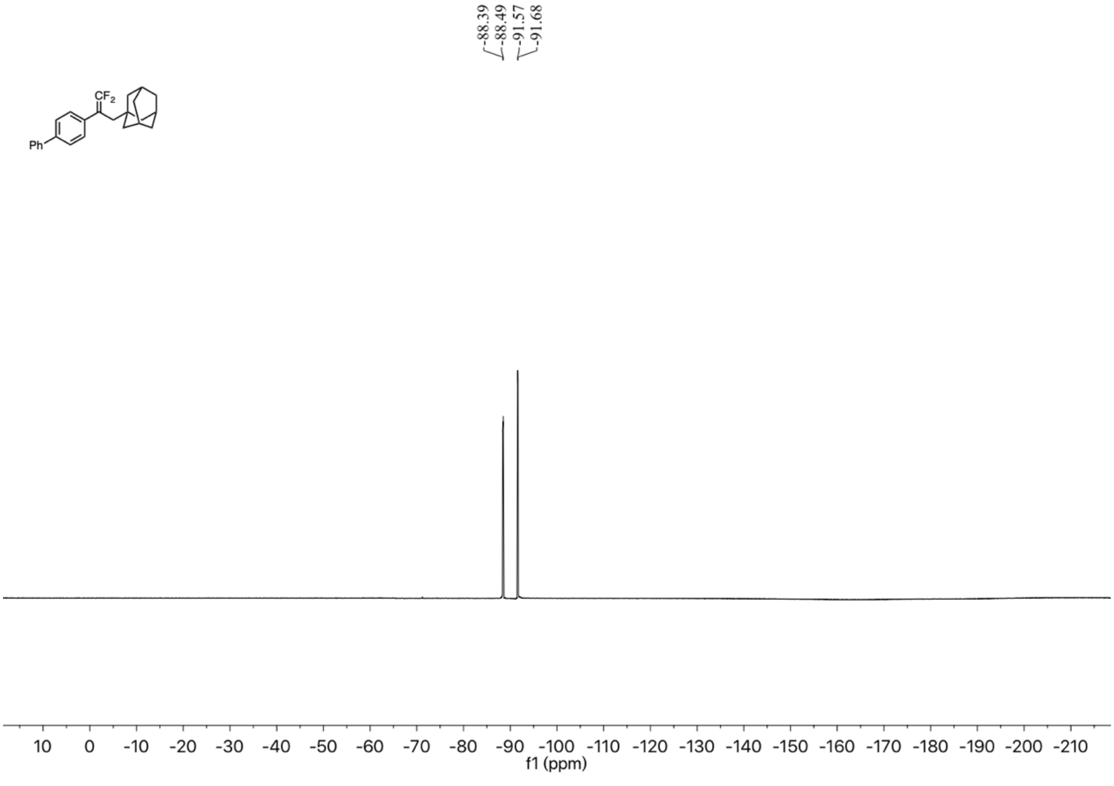

**$^1$H NMR (400 MHz, $CDCl_3$)** spectrum of **methyl 4-(2-([1,1'-biphenyl]-4-yl)-3,3-difluoroallyl)bicyclo[2.2.2]octane-1-carboxylate (51)**

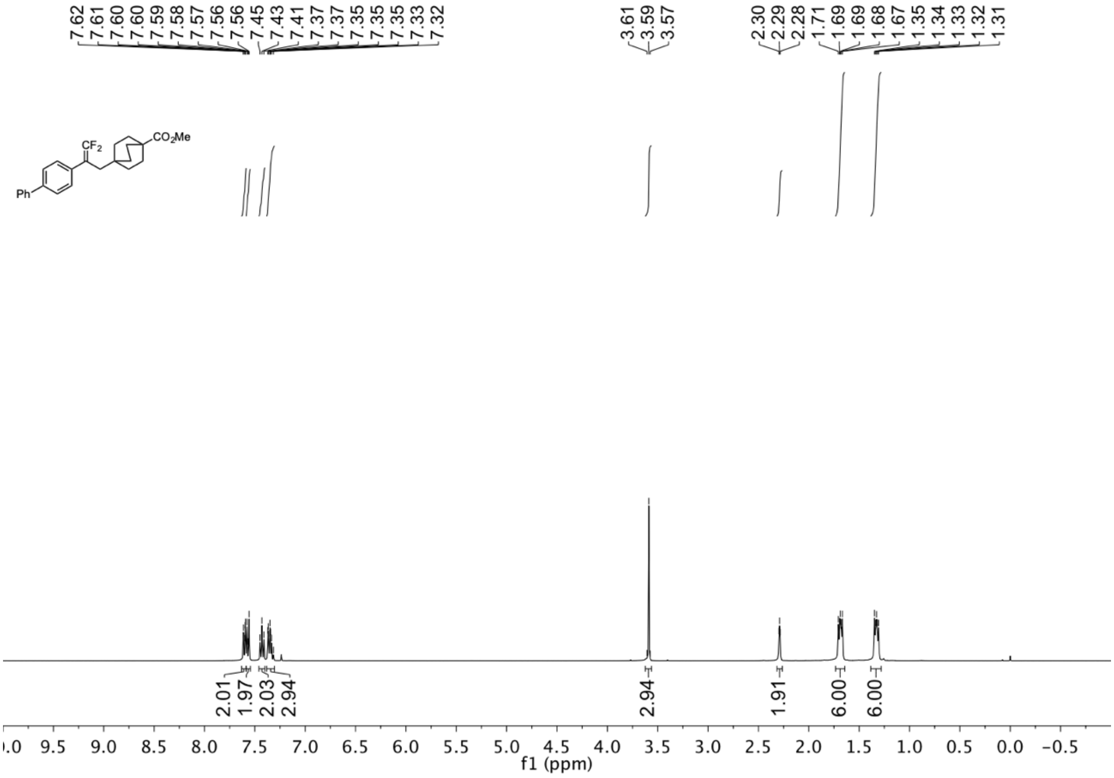


**$^{13}$C NMR (126 MHz, $CDCl_3$)** spectrum of **methyl 4-(2-([1,1'-biphenyl]-4-yl)-3,3-difluoroallyl)bicyclo[2.2.2]octane-1-carboxylate (51)**

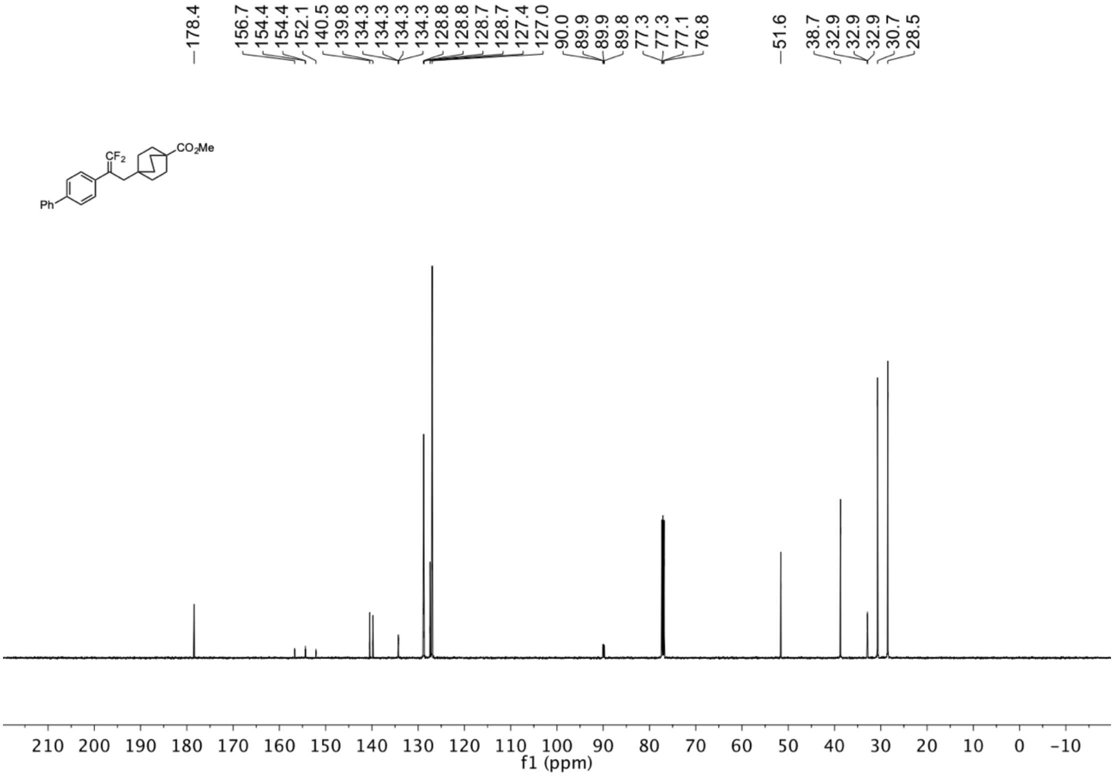

**$^{19}F$ NMR (376 MHz, $CDCl_3$)** spectrum of **methyl 4-(2-([1,1'-biphenyl]-4-yl)-3,3-difluoroallyl)bicyclo[2.2.2]octane-1-carboxylate (51)**

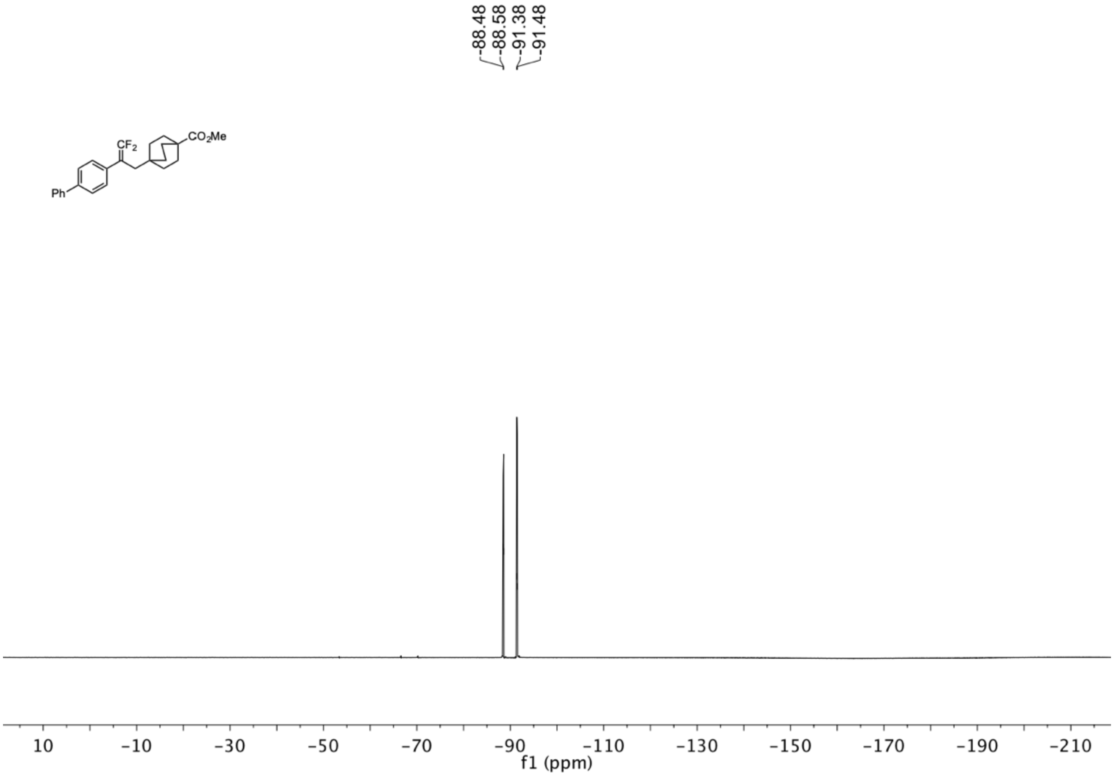


**$^{1}H$ NMR (400 MHz, $CDCl_3$)** spectrum of ***tert*-butyl 4-((3-(dibenzo[*b*,*d*]thiophen-4-yl)-4,4-difluorobut-3-enoyl)oxy)piperidine-1-carboxylate (52)**

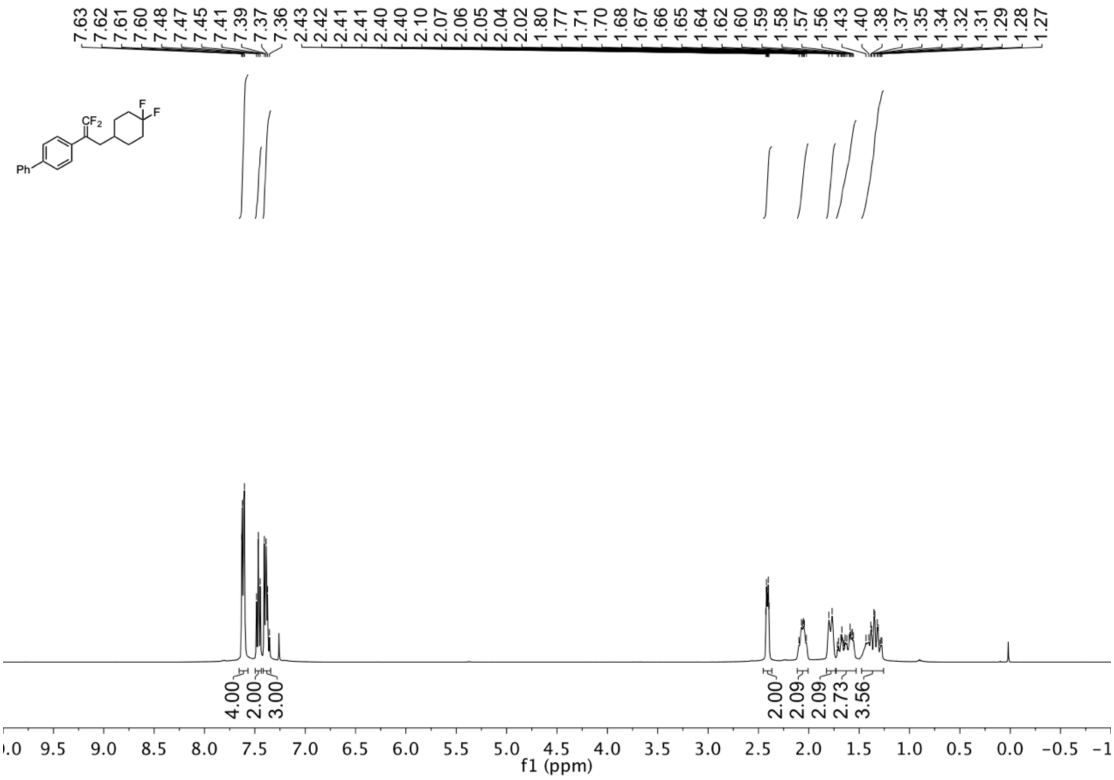

**$^{13}C$ NMR (126 MHz, $CDCl_3$)** spectrum of ***tert*-butyl 4-((3-(dibenzo[*b*,*d*]thiophen-4-yl)-4,4-difluorobut-3-enoyl)oxy)piperidine-1-carboxylate (52)**

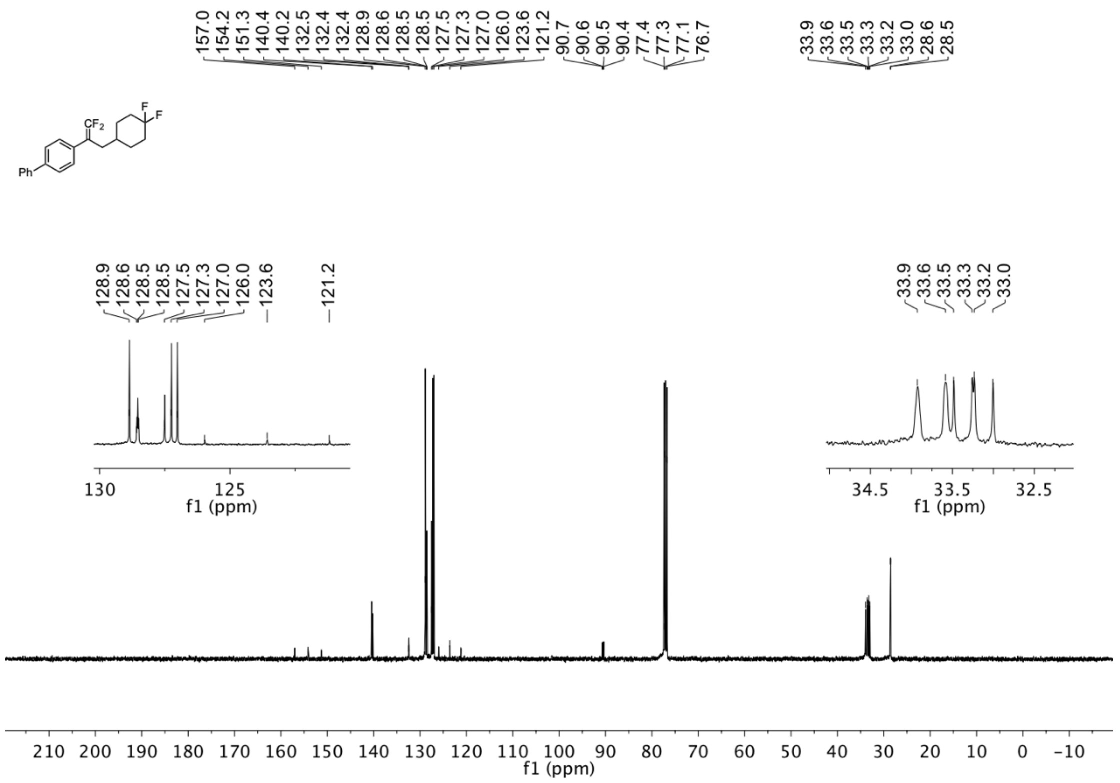


**$^{19}F$ NMR (376 MHz, $CDCl_3$)** spectrum of ***tert*-butyl 4-((3-(dibenzo[*b*,*d*]thiophen-4-yl)-4,4-difluorobut-3-enoyl)oxy)piperidine-1-carboxylate (52)**

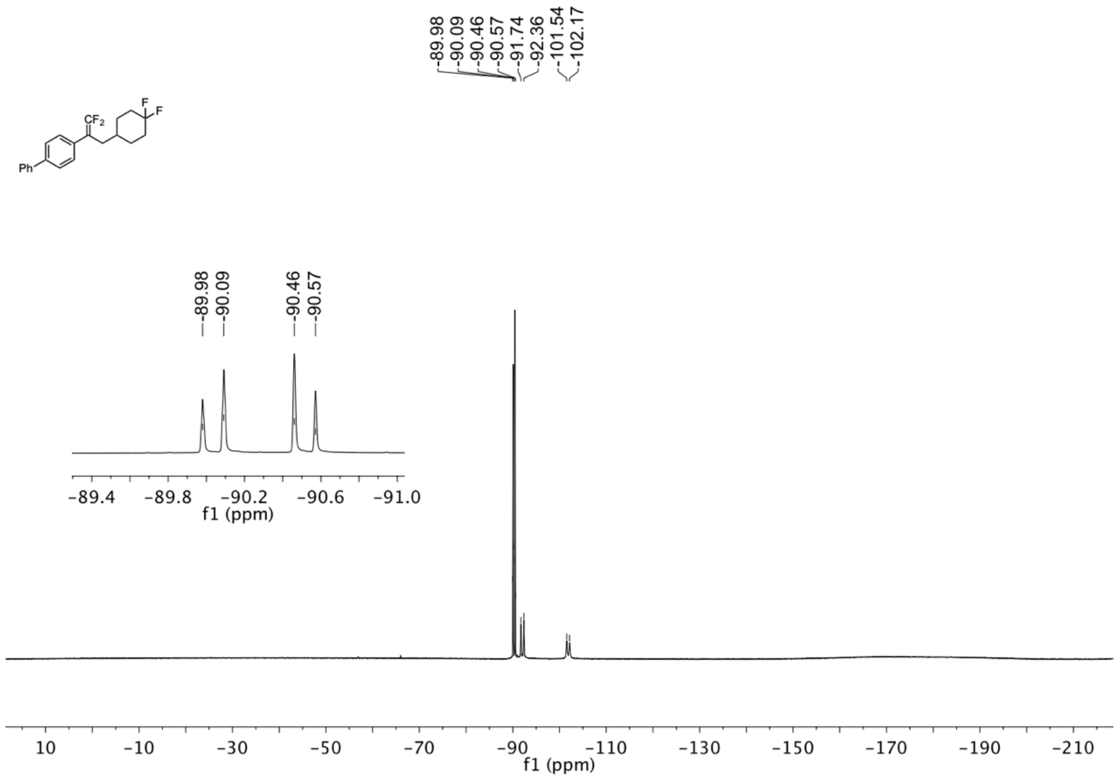

**$^{1}H$ NMR (400 MHz, $CDCl_3$)** spectrum of **4-(1,1-difluoro-4-methylhex-1-en-2-yl)-1,1'-biphenyl (53)**

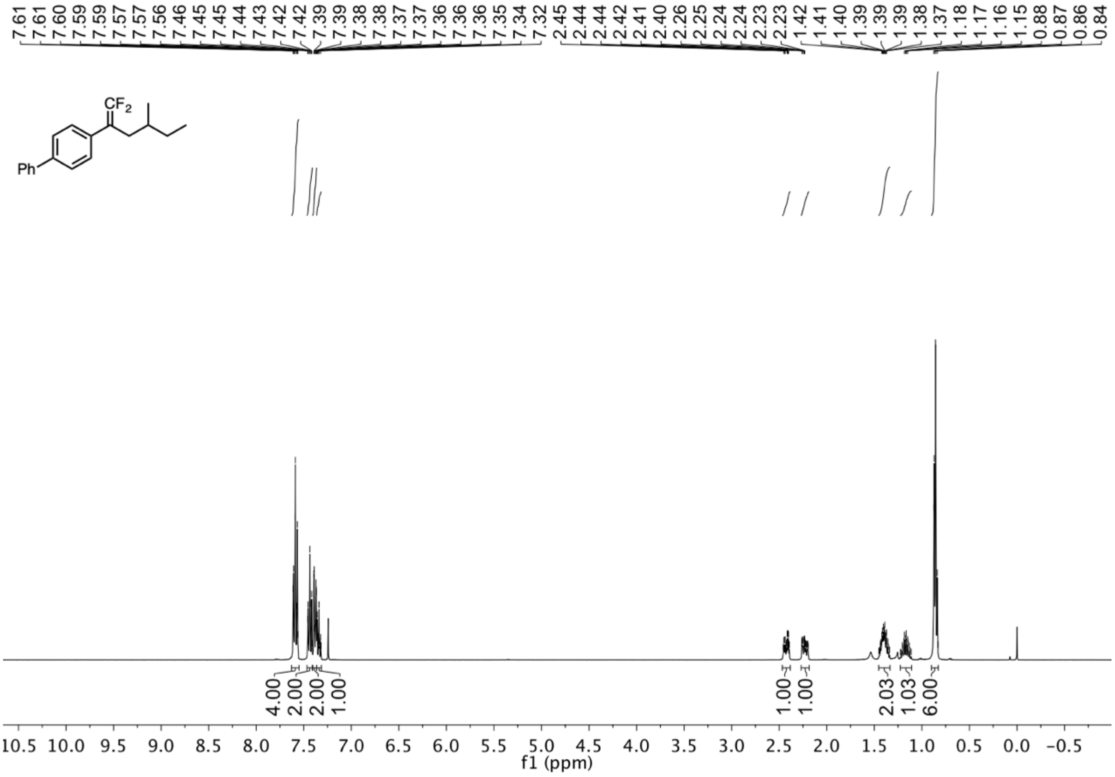


**$^{13}C$ NMR (126 MHz, $CDCl_3$)** spectrum of **4-(1,1-difluoro-4-methylhex-1-en-2-yl)-1,1'-biphenyl (53)**

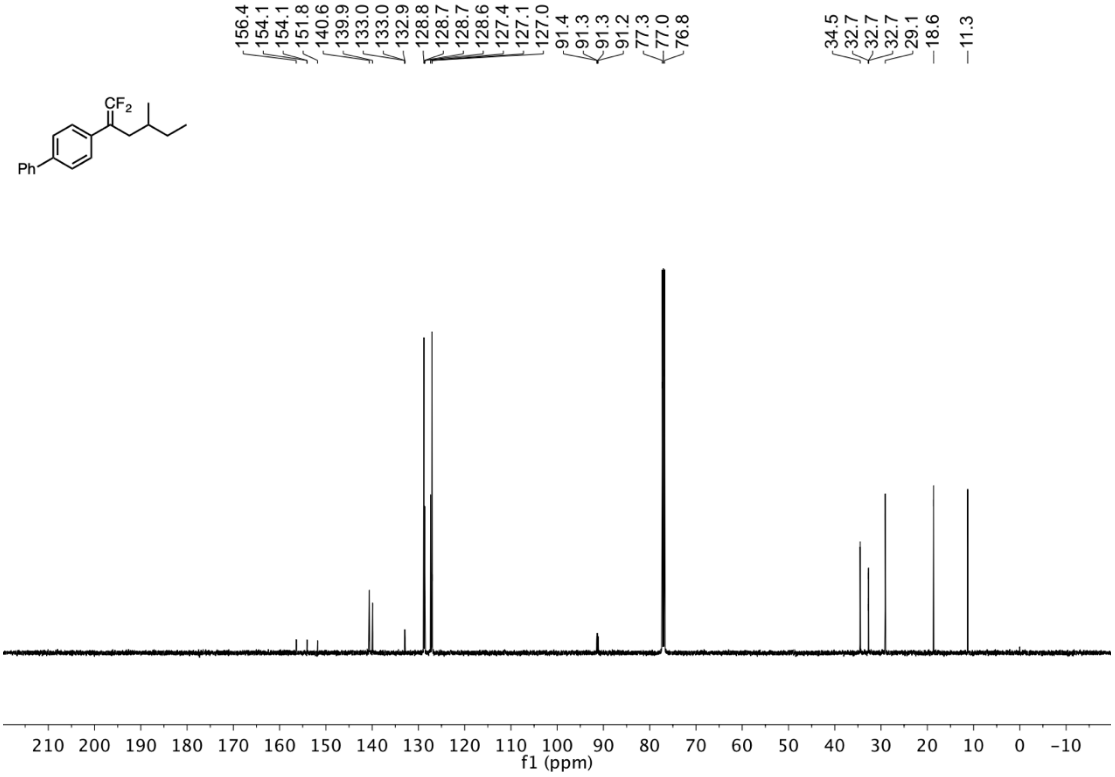

**$^{19}$F NMR (376 MHz, $CDCl_3$)** spectrum of **4-(1,1-difluoro-4-methylhex-1-en-2-yl)-1,1'-biphenyl (53)**

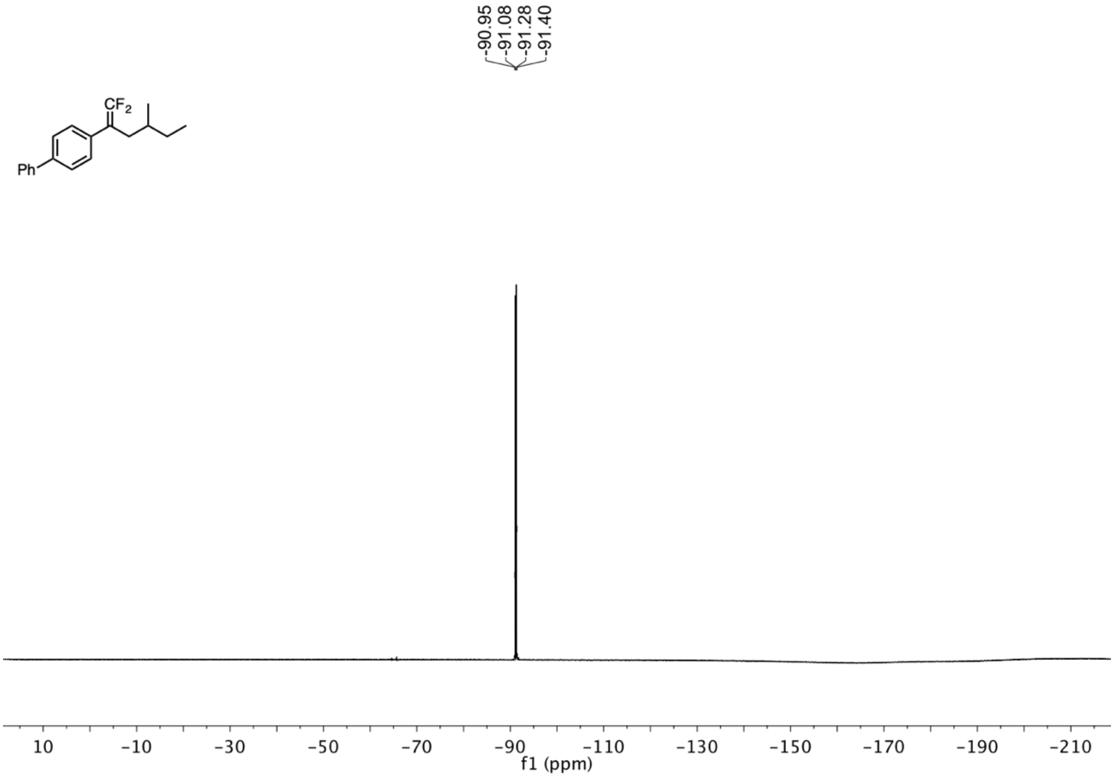


**$^{1}$H NMR (400 MHz, $CDCl_3$)** spectrum of **4-(1,1-difluoro-3-(1-phenylcyclopropyl)prop-1-en-2-yl)-1,1'-biphenyl (54)**

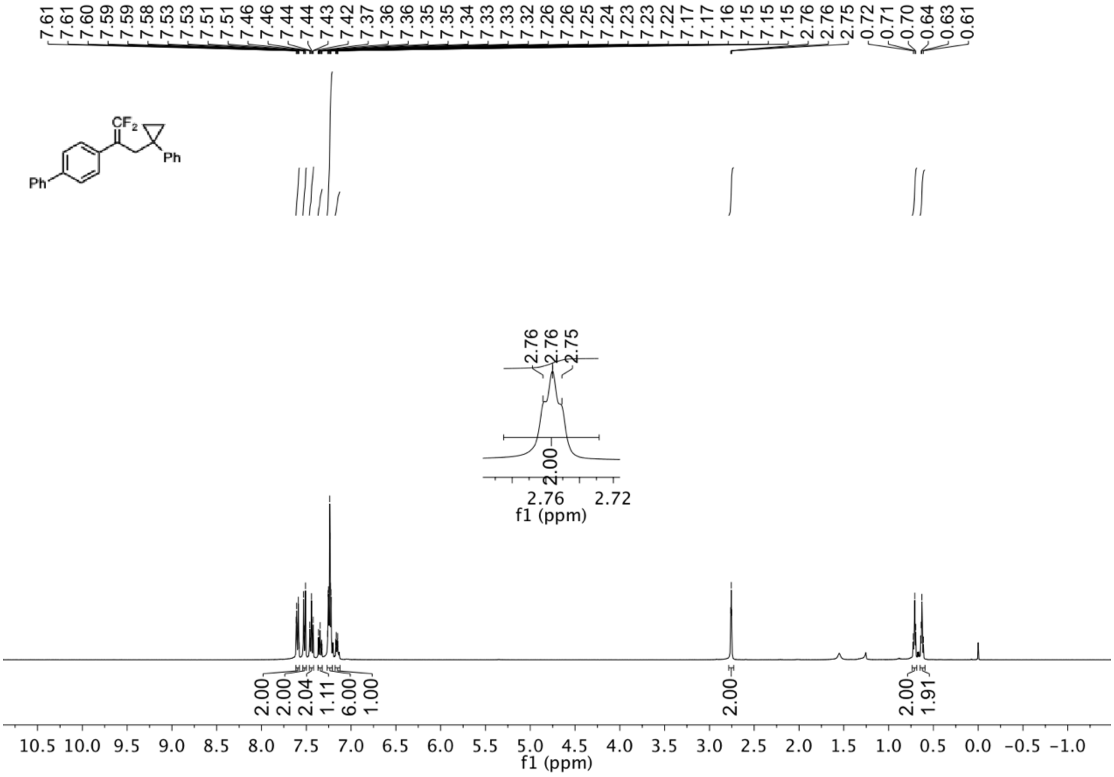

$^{13}$C NMR (101 MHz, CDCl$_3$) spectrum of **4-(1,1-difluoro-3-(1-phenylcyclopropyl)prop-1-en-2-yl)-1,1'-biphenyl (54)**

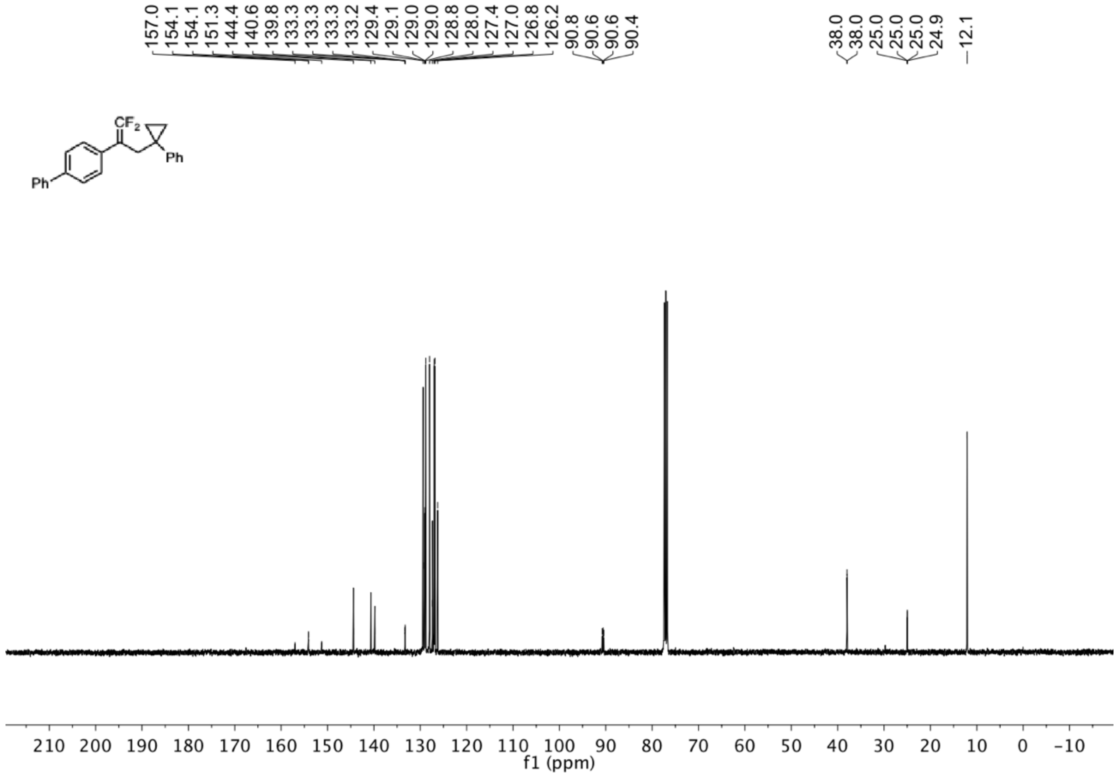


$^{19}$F NMR (376 MHz, CDCl$_3$) spectrum of **4-(1,1-difluoro-3-(1-phenylcyclopropyl)prop-1-en-2-yl)-1,1'-biphenyl (54)**

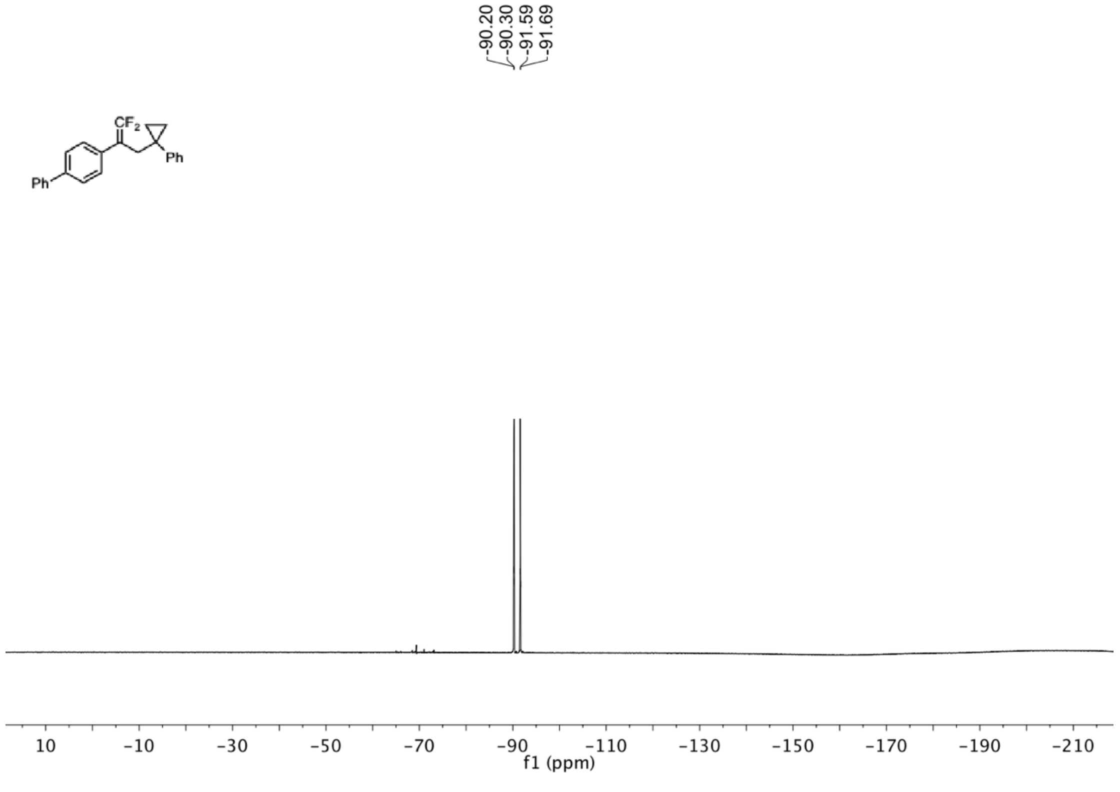

**$^{1}$H NMR (400 MHz, CDCl$_3$)** spectrum of ***tert*-butyl (1-(2-([1,1'-biphenyl]-4-yl)-3,3-difluoroallyl)cyclobutyl)carbamate (55)**

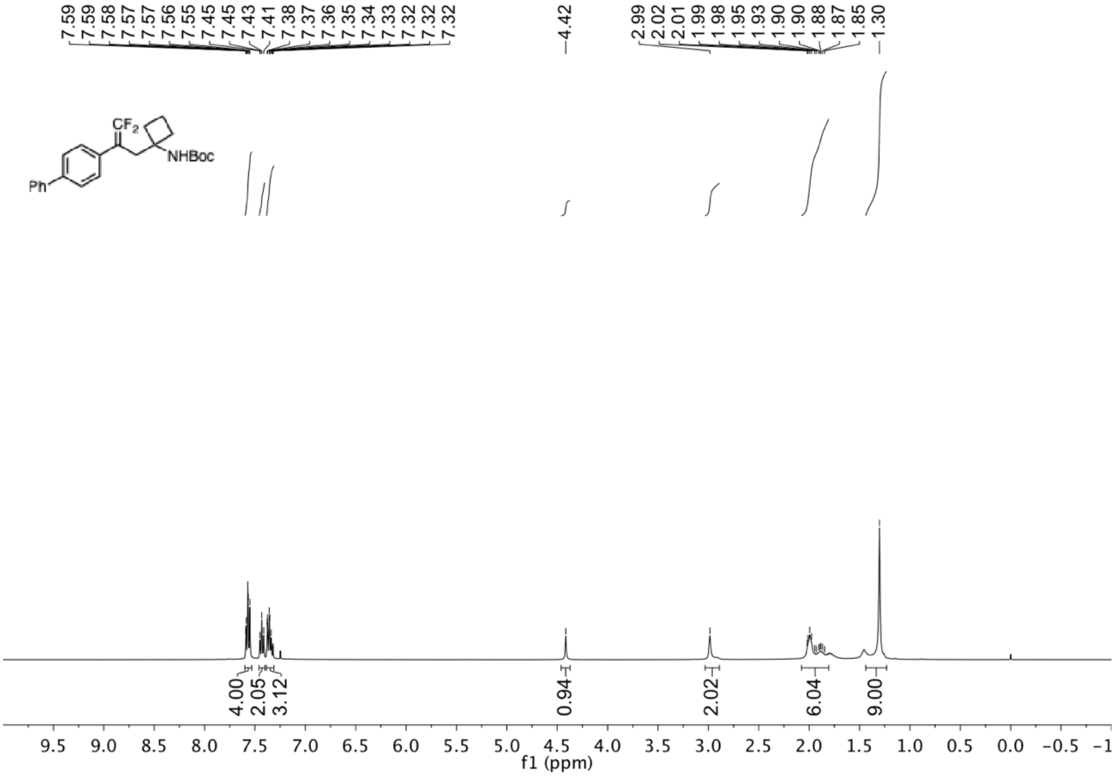

**$^{13}$C NMR (101 MHz, CDCl$_3$)** spectrum of ***tert*-butyl (1-(2-([1,1'-biphenyl]-4-yl)-3,3-difluoroallyl)cyclobutyl)carbamate (55)**

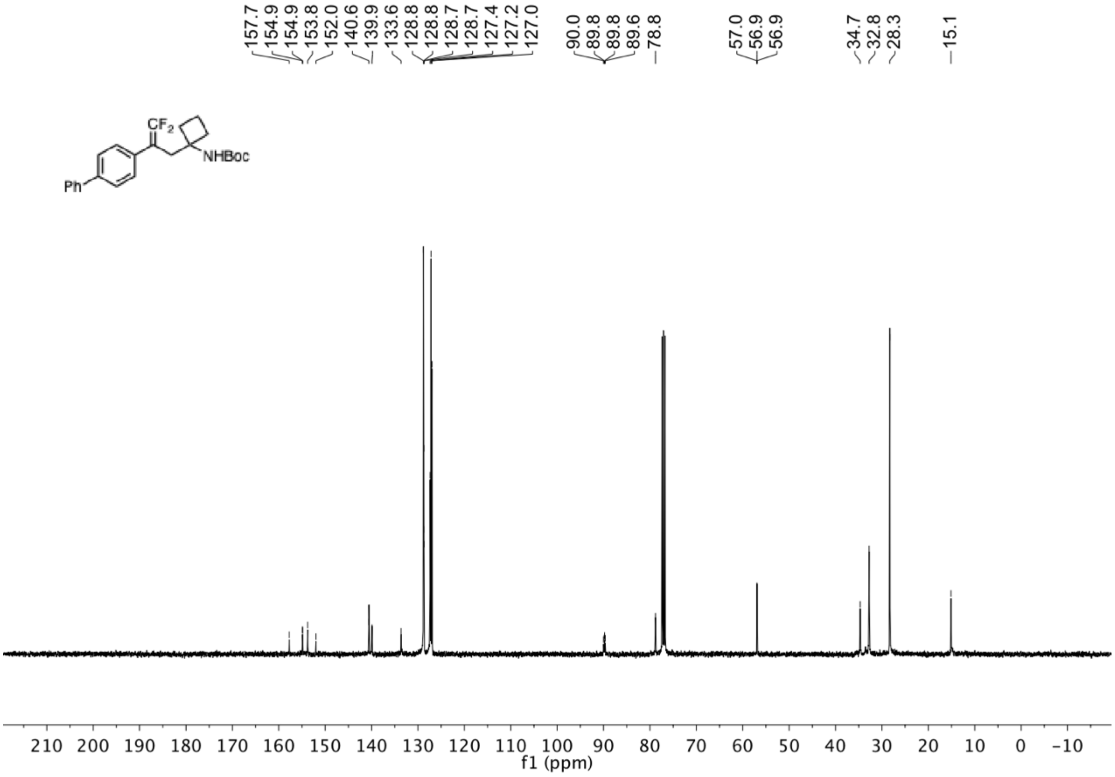

**$^{19}$F NMR (376 MHz, CDCl$_3$)** spectrum of ***tert*-butyl (1-(2-([1,1'-biphenyl]-4-yl)-3,3-difluoroallyl)cyclobutyl)carbamate (55)**

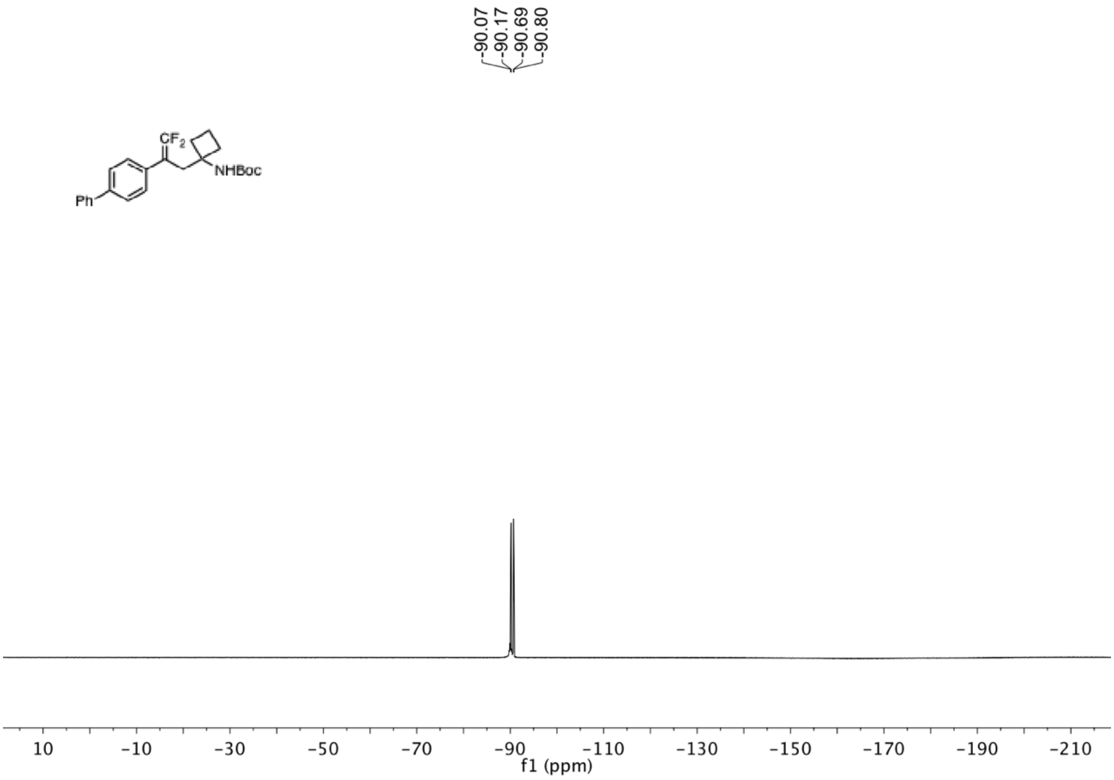


**$^{1}$H NMR (500 MHz, CDCl$_3$)** spectrum of **4-(1,1-difluoro-4-(*p*-tolyl)but-1-en-2-yl)-1,1'-biphenyl (56)**

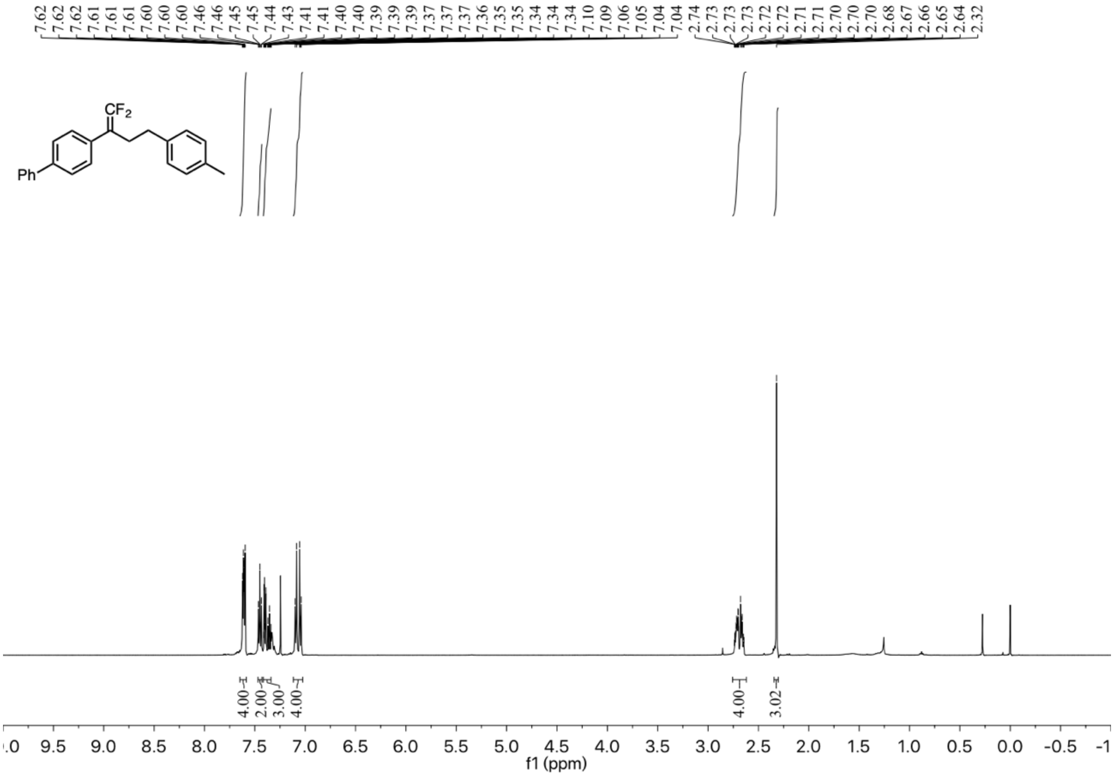

$^{13}$C NMR (126 MHz, $CDCl_3$)** spectrum of **4-(1,1-difluoro-4-(*p*-tolyl)but-1-en-2-yl)-1,1'-biphenyl (56)**

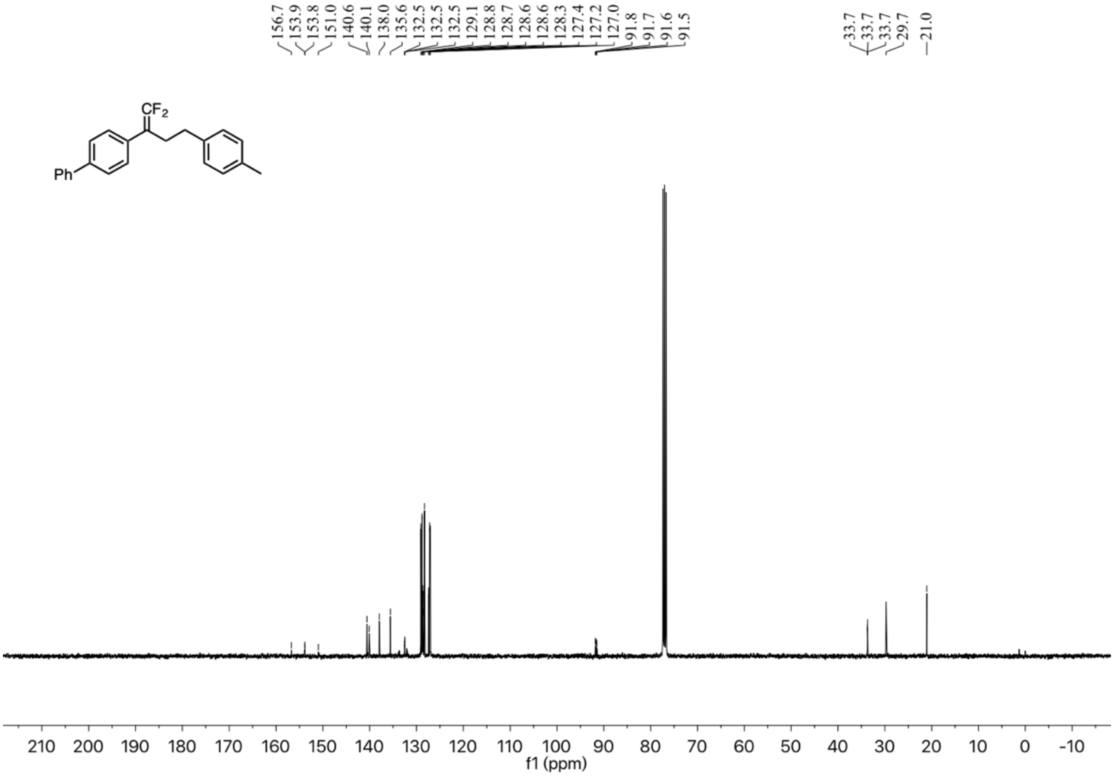


$^{19}$F NMR (471 MHz, $CDCl_3$)** spectrum of **4-(1,1-difluoro-4-(*p*-tolyl)but-1-en-2-yl)-1,1'-biphenyl (56)**

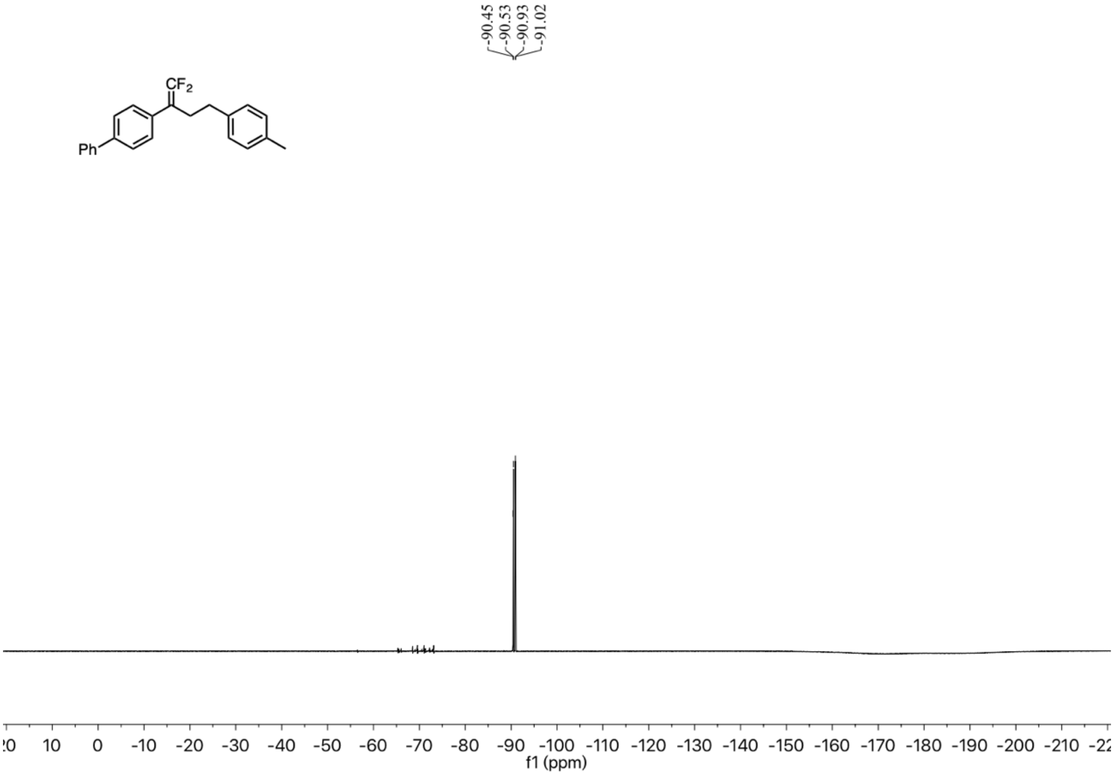

**$^{1}$H NMR (500 MHz, $CDCl_3$)** spectrum of **4-(1,1-difluoro-4-(3-methoxyphenyl)but-1-en-2-yl)-1,1'-biphenyl (57)**

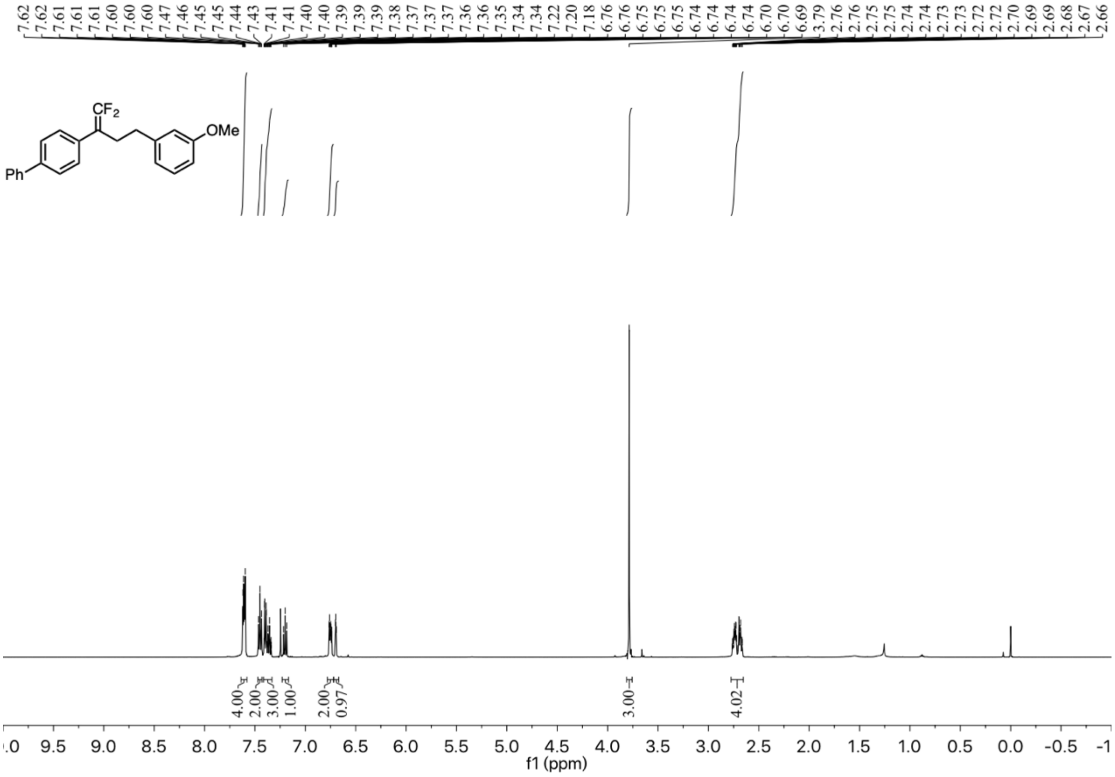

**$^{13}$C NMR (101 MHz, $CDCl_3$)** spectrum of **4-(1,1-difluoro-4-(3-methoxyphenyl)but-1-en-2-yl)-1,1'-biphenyl (57)**

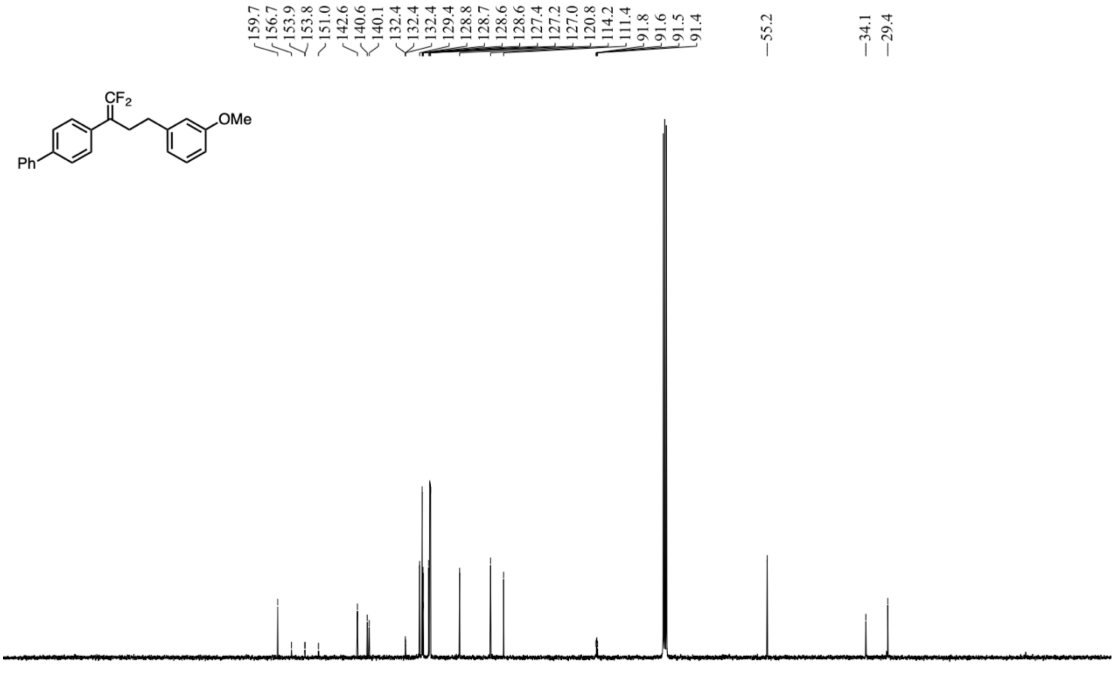

**$^{19}$F NMR (471 MHz, CDCl$_3$)** spectrum of **4-(1,1-difluoro-4-(3-methoxyphenyl)but-1-en-2-yl)-1,1'-biphenyl (57)**

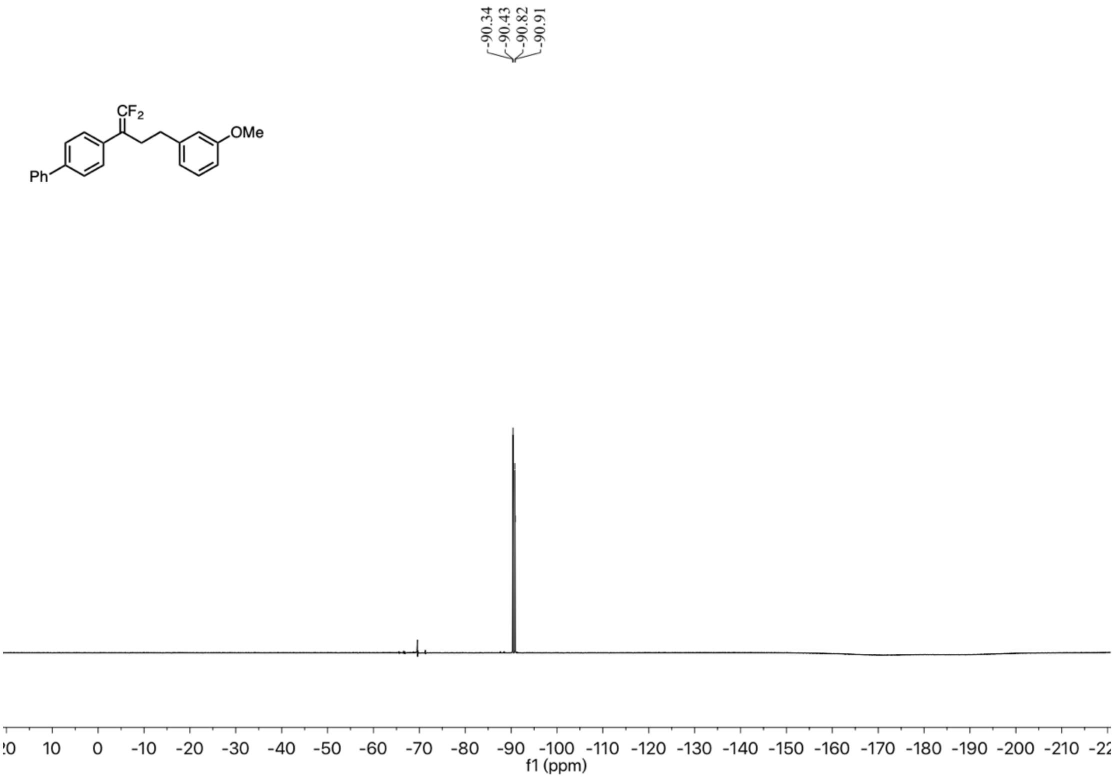


**$^{1}$H NMR (500 MHz, CDCl$_3$)** spectrum of **4-(1,1-difluoro-4-(4-fluorophenyl)but-1-en-2-yl)-1,1'-biphenyl (58)**

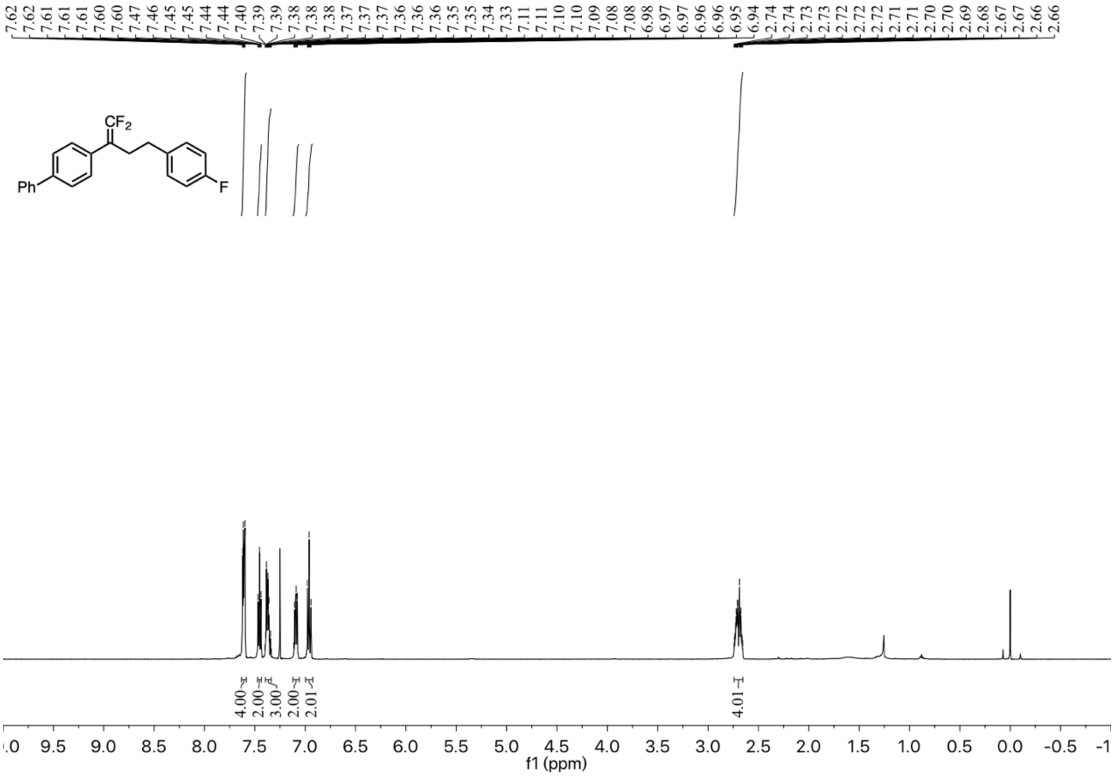

**$^{13}C$ NMR (101 MHz, $CDCl_3$)** spectrum of **4-(1,1-difluoro-4-(4-fluorophenyl)but-1-en-2-yl)-1,1'-biphenyl (58)**

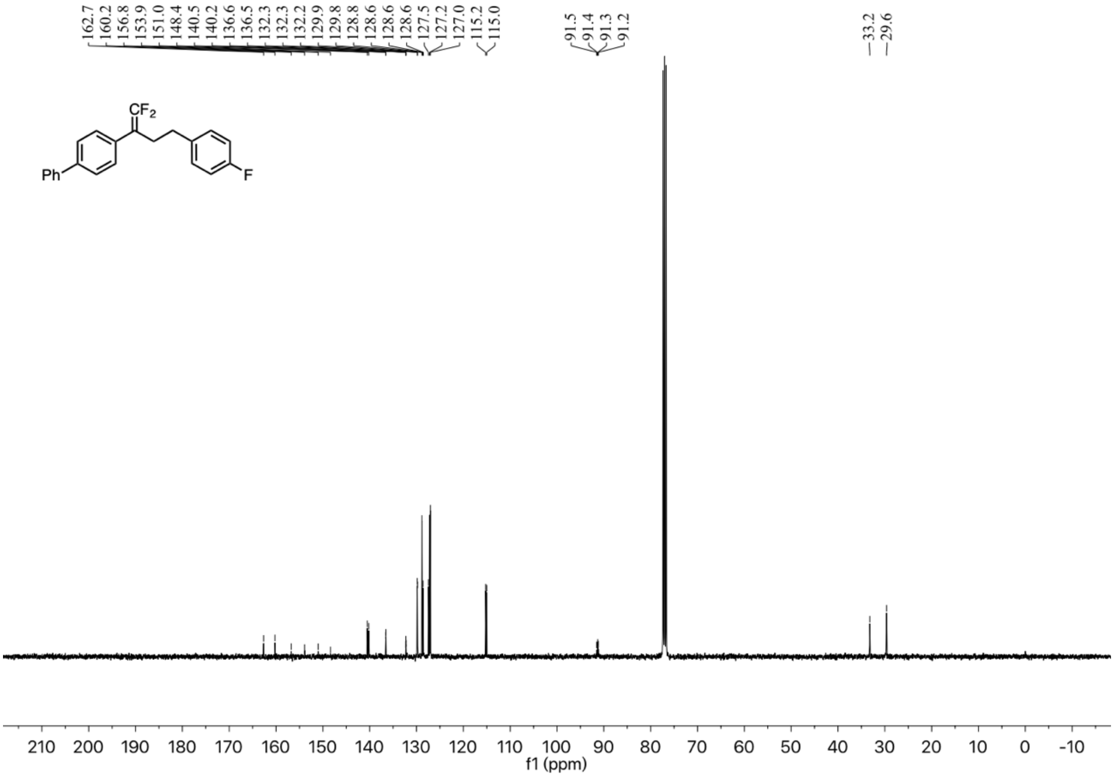


**$^{19}F$ NMR (471 MHz, $CDCl_3$)** spectrum of **4-(1,1-difluoro-4-(4-fluorophenyl)but-1-en-2-yl)-1,1'-biphenyl (58)**

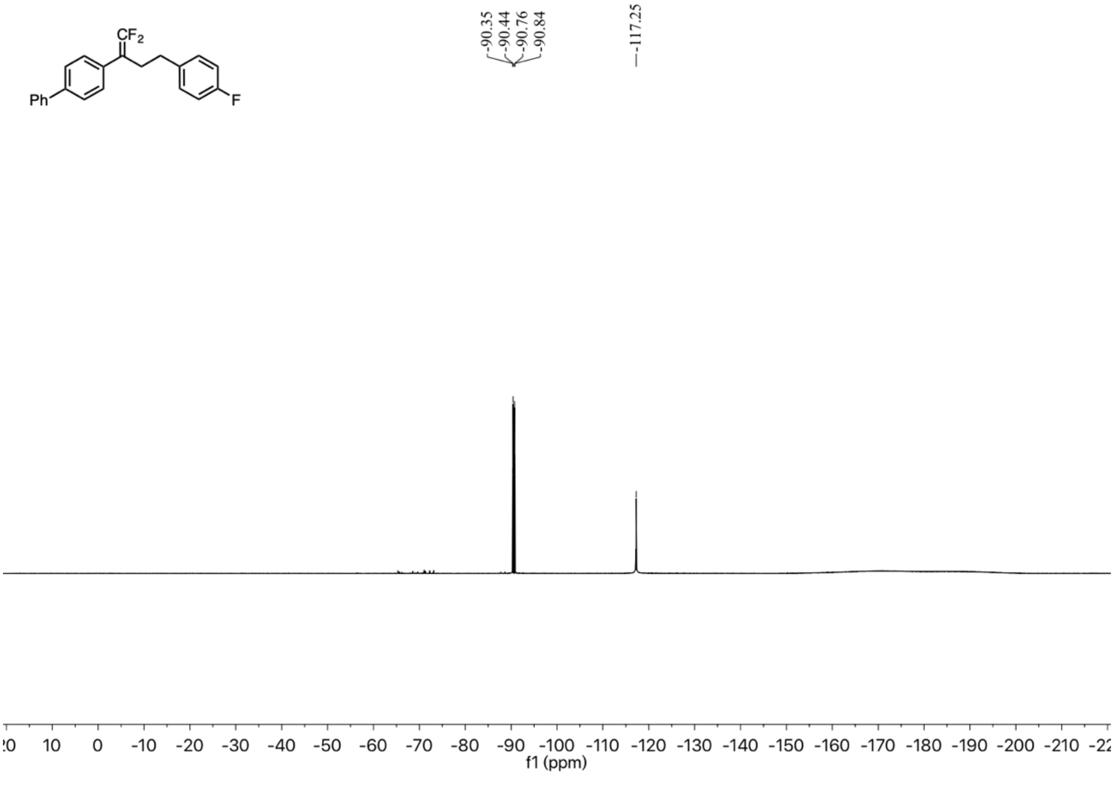

**$^1$H NMR (500 MHz, CDCl$_3$)** spectrum of ***N*-(3-(1,1-difluoro-4,4-dimethylpent-1-en-2-yl)phenyl)-4-(*N*,*N*-dipropylsulfamoyl)benzamide (59)**

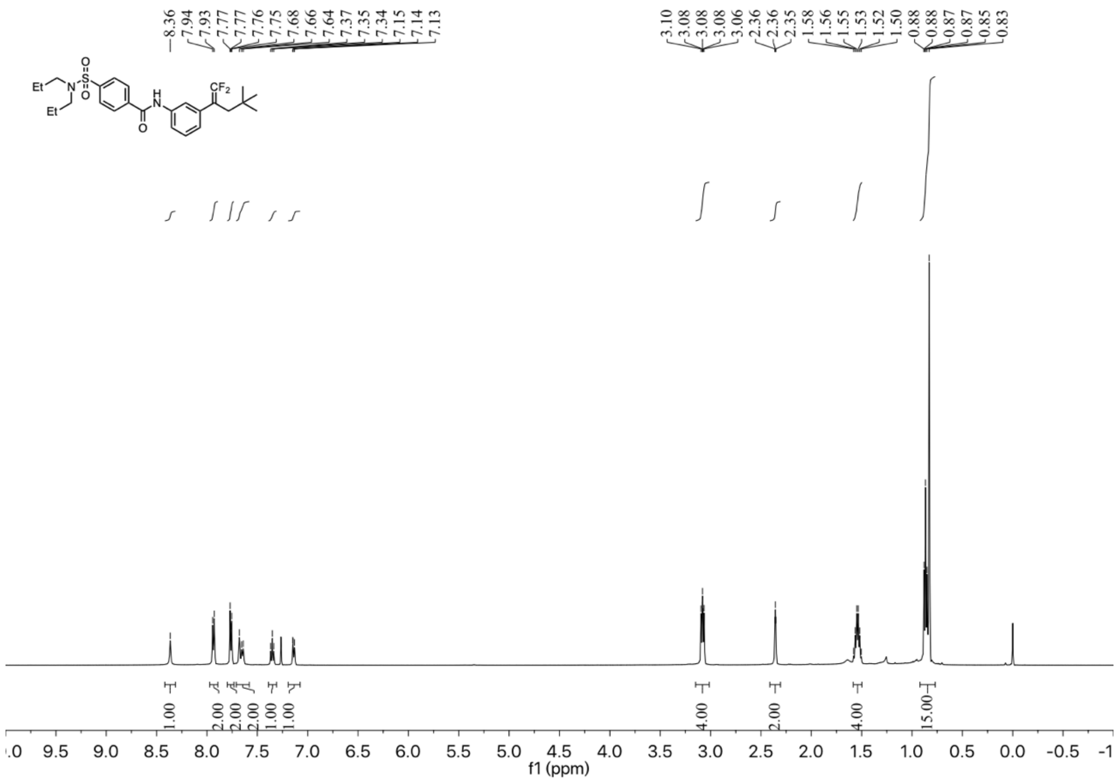

**$^{13}$C NMR (126 MHz, CDCl$_3$)** spectrum of ***N*-(3-(1,1-difluoro-4,4-dimethylpent-1-en-2-yl)phenyl)-4-(*N*,*N*-dipropylsulfamoyl)benzamide (59)**

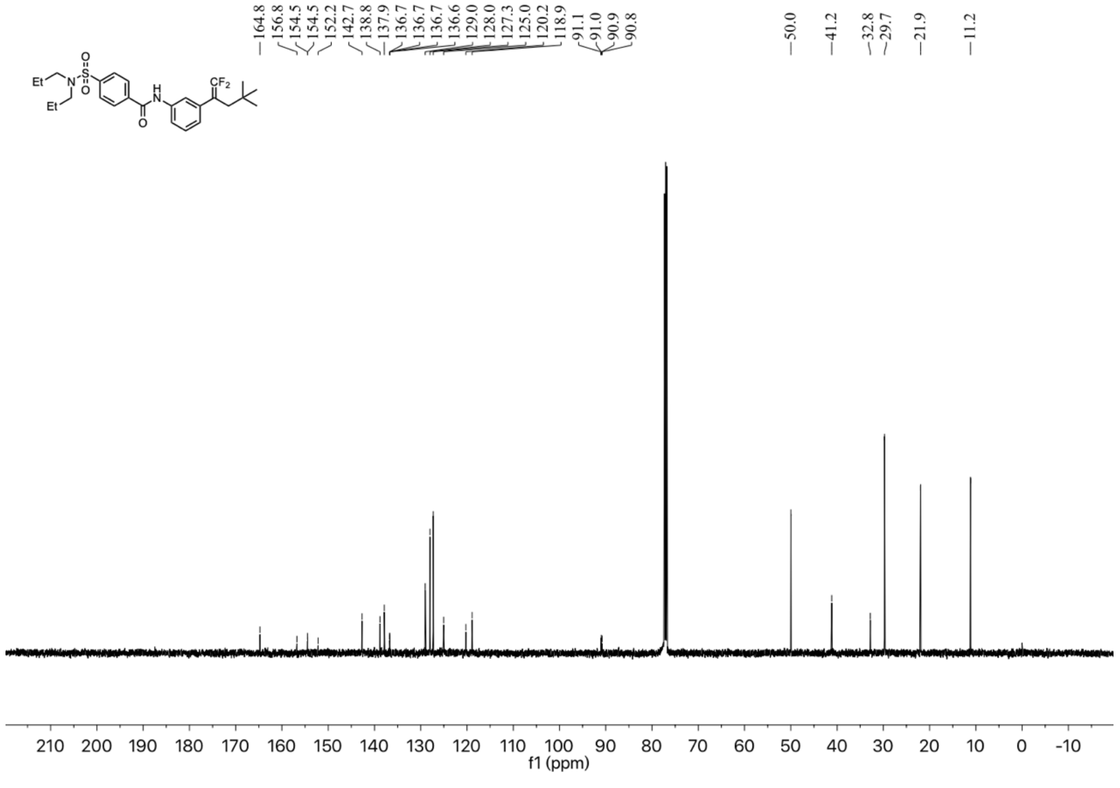

**$^{19}$F NMR (471 MHz, $CDCl_3$)** spectrum of ***N*-(3-(1,1-difluoro-4,4-dimethylpent-1-en-2-yl)phenyl)-4-(*N*,*N*-dipropylsulfamoyl)benzamide (59)**

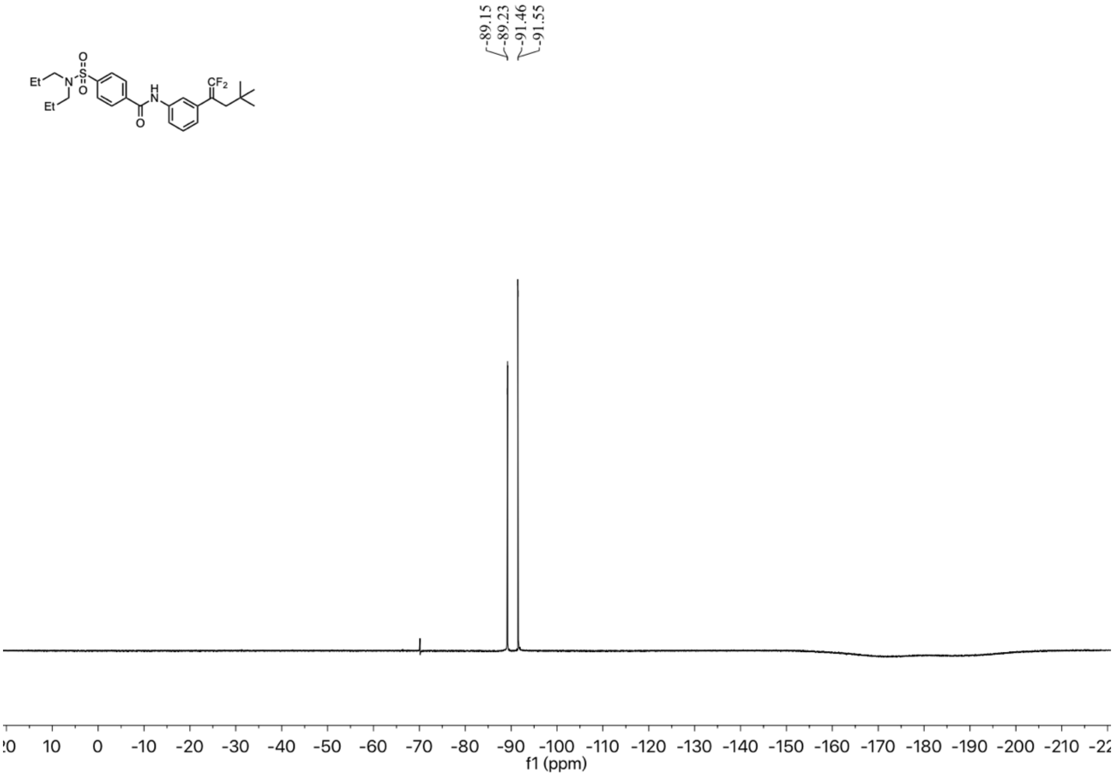

**$^{1}$H NMR (400 MHz, $CDCl_3$)** spectrum of **4-(1,1-difluoro-4,4-dimethylpent-1-en-2-yl)phenyl 2-(2-((2,6-dichlorophenyl)amino)phenyl)acetate (60)**

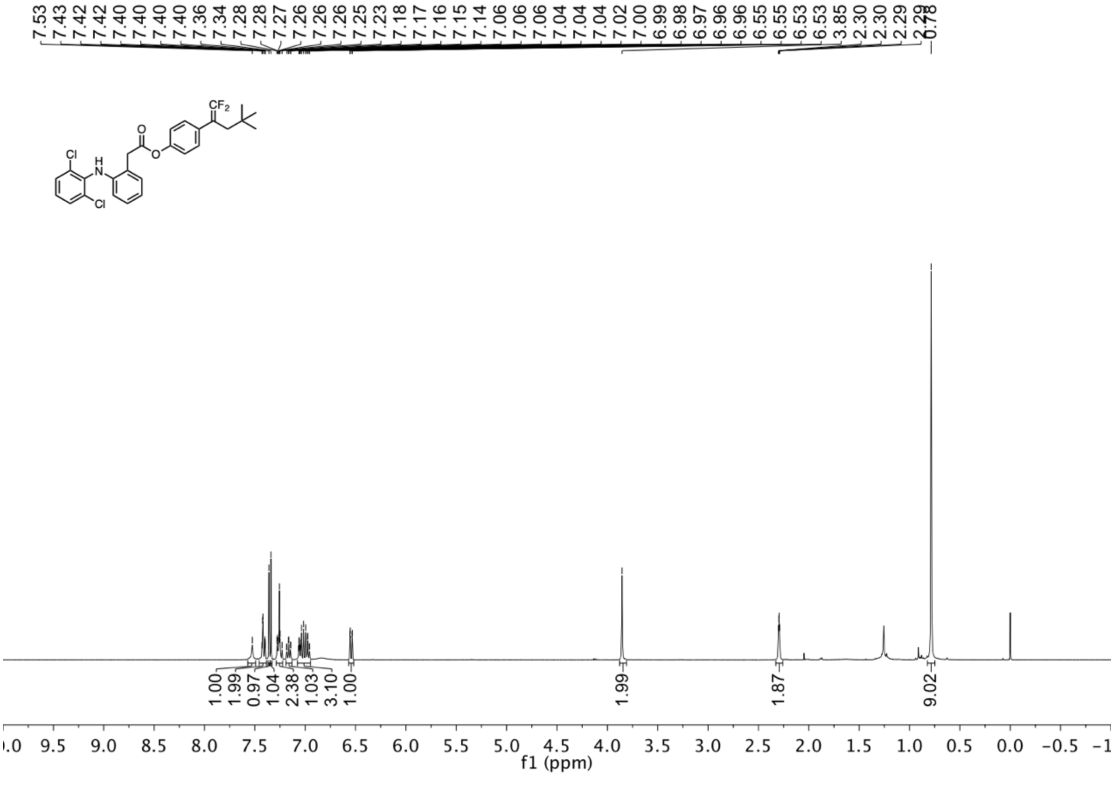

$^{13}$C NMR (101 MHz, CDCl$_3$) spectrum of **4-(1,1-difluoro-4,4-dimethylpent-1-en-2-yl)phenyl 2-(2-((2,6-dichlorophenyl)amino)phenyl)acetate (60)**

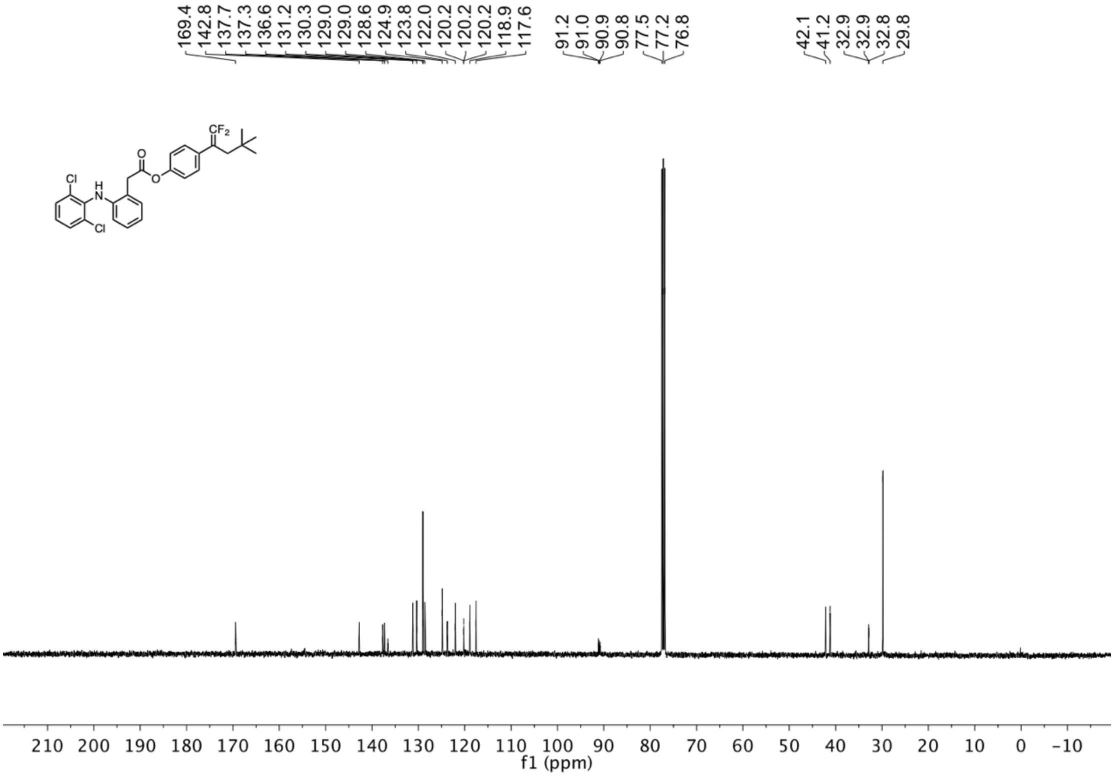


$^{19}$F NMR (376 MHz, CDCl$_3$) spectrum of **4-(1,1-difluoro-4,4-dimethylpent-1-en-2-yl)phenyl 2-(2-((2,6-dichlorophenyl)amino)phenyl)acetate (60)**

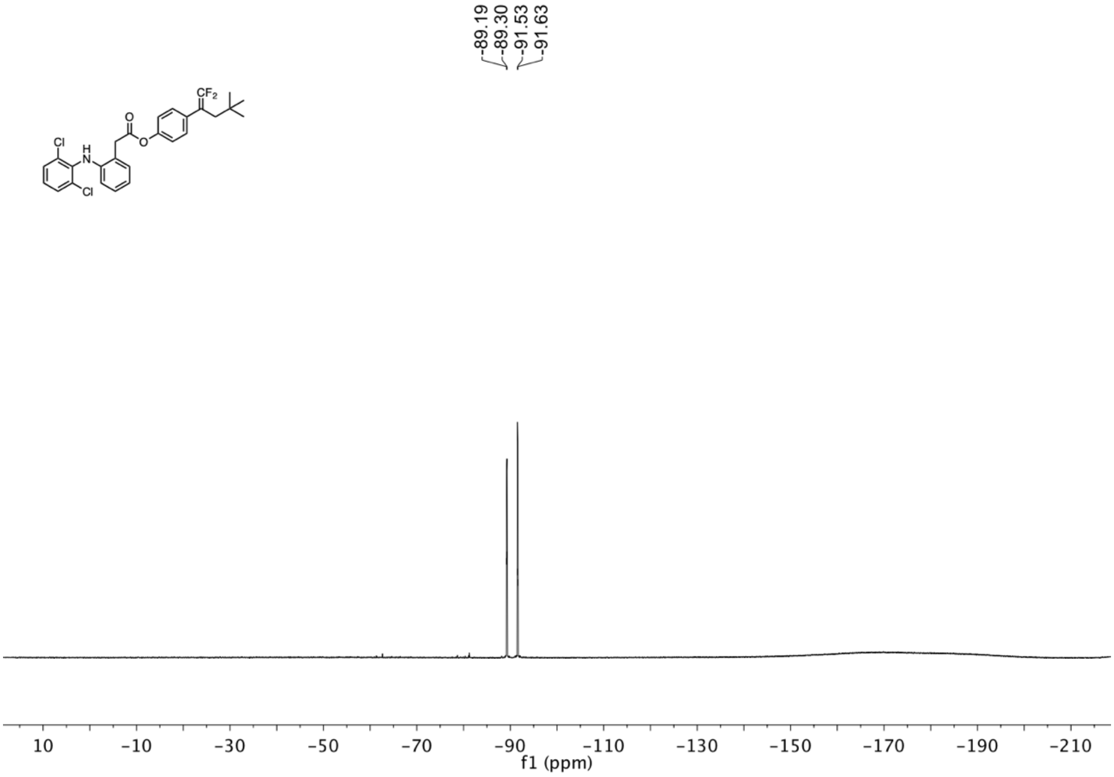

**$^{1}H$ NMR (500 MHz, $CDCl_3$)** spectrum of **4-(1,1-difluoro-4,4-dimethylpent-1-en-2-yl)phenyl 2-(1-(4-chlorobenzoyl)-5-methoxy-2-methyl-1*H*-indol-3-yl)acetate (61)**

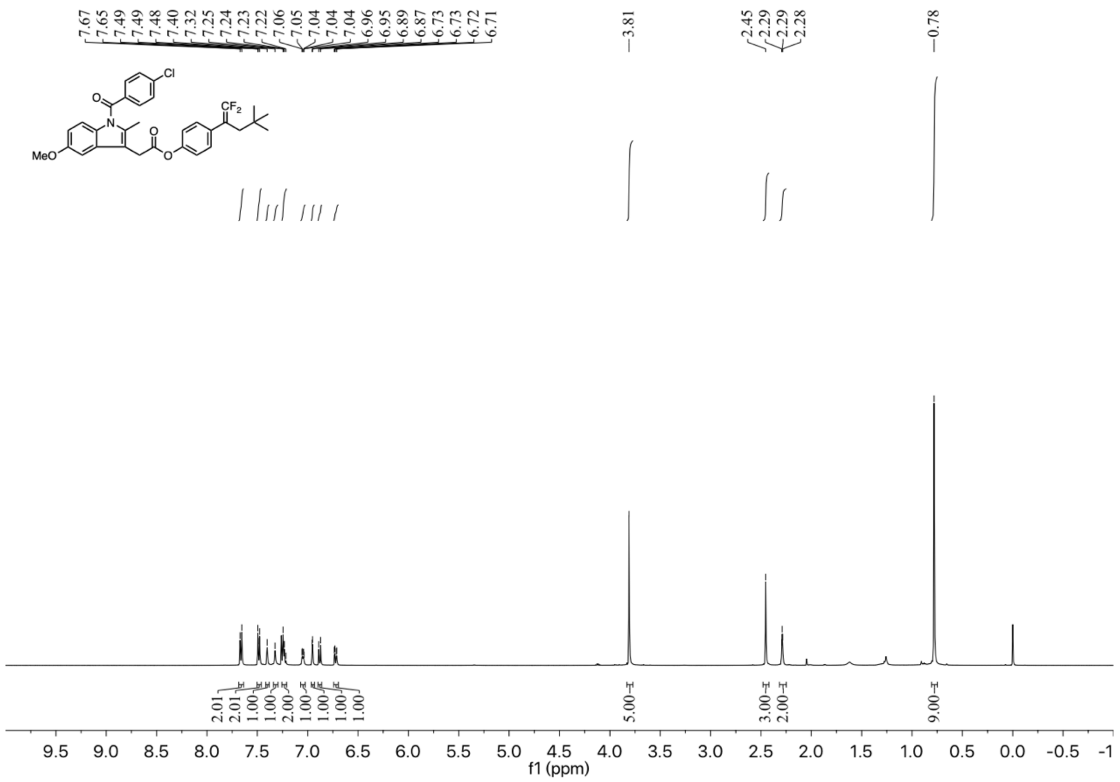

**$^{13}C$ NMR (126 MHz, $CDCl_3$)** spectrum of **4-(1,1-difluoro-4,4-dimethylpent-1-en-2-yl)phenyl 2-(1-(4-chlorobenzoyl)-5-methoxy-2-methyl-1*H*-indol-3-yl)acetate (61)**

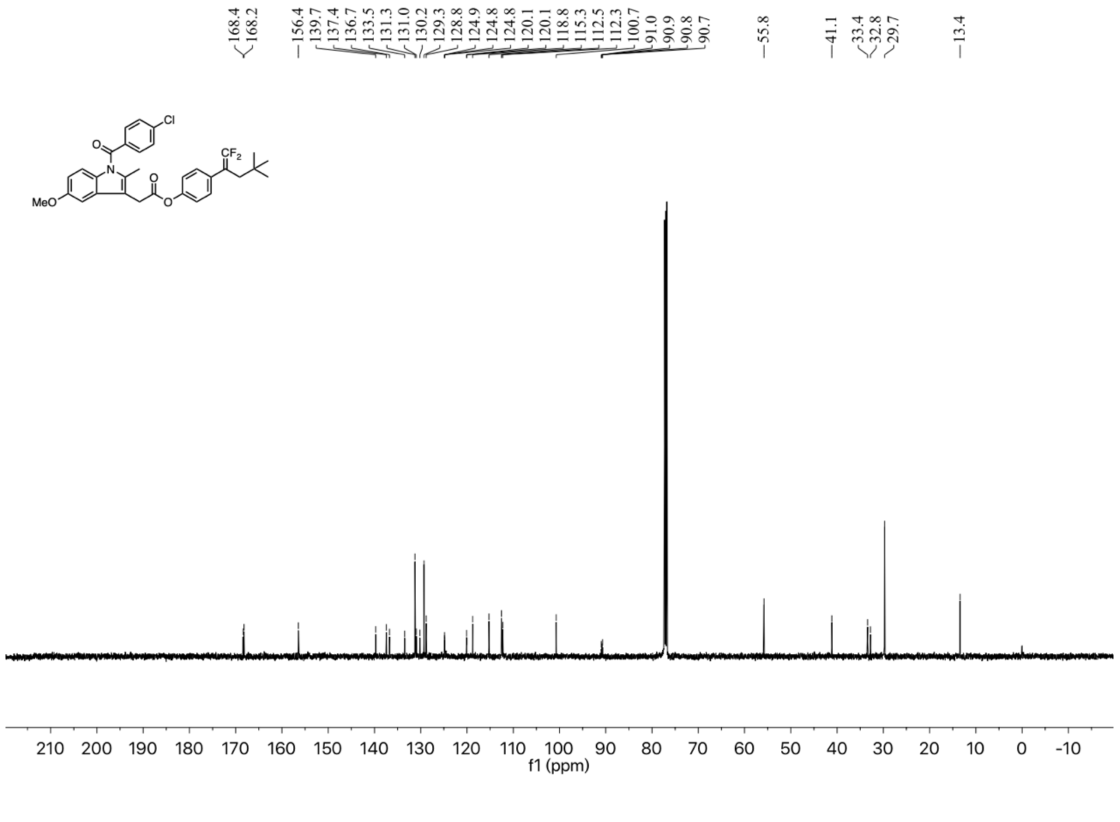

**$^{19}F$ NMR (471 MHz, $CDCl_3$)** spectrum of **4-(1,1-difluoro-4,4-dimethylpent-1-en-2-yl)phenyl 2-(1-(4-chlorobenzoyl)-5-methoxy-2-methyl-1*H*-indol-3-yl)acetate (61)**

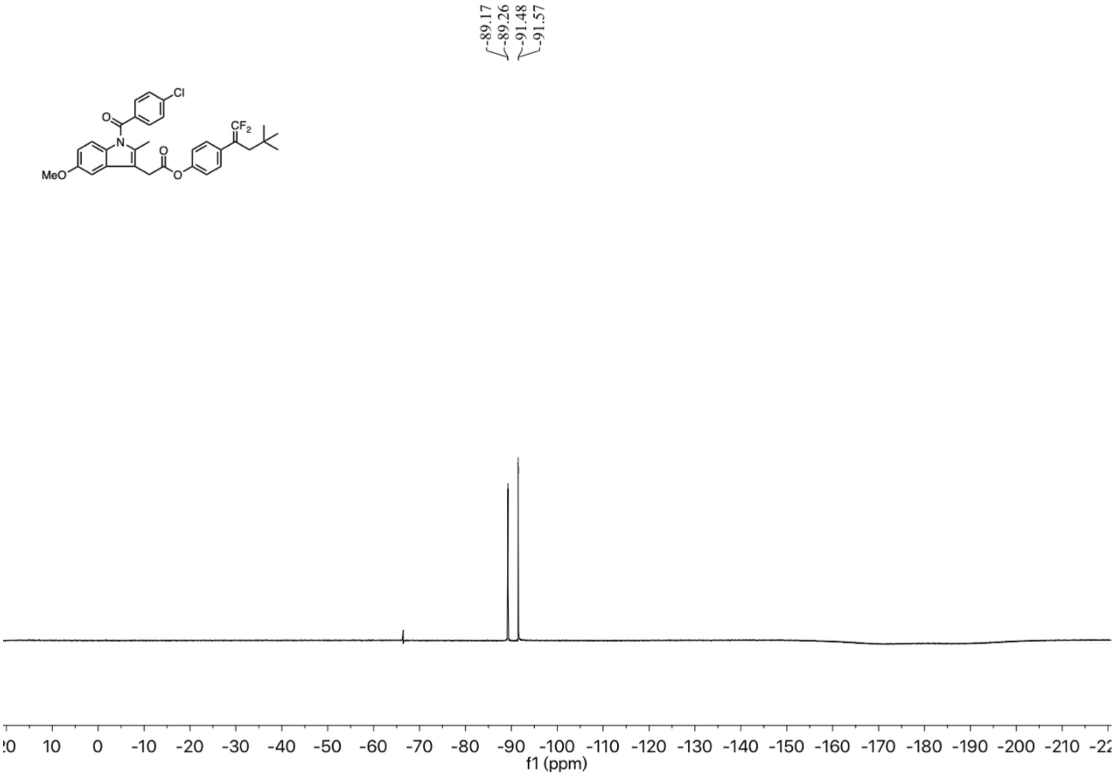

**$^{1}H$ NMR (400 MHz, $CDCl_3$)** spectrum of **((3,3-dimethylbutyl)sulfonyl)benzene (62)**

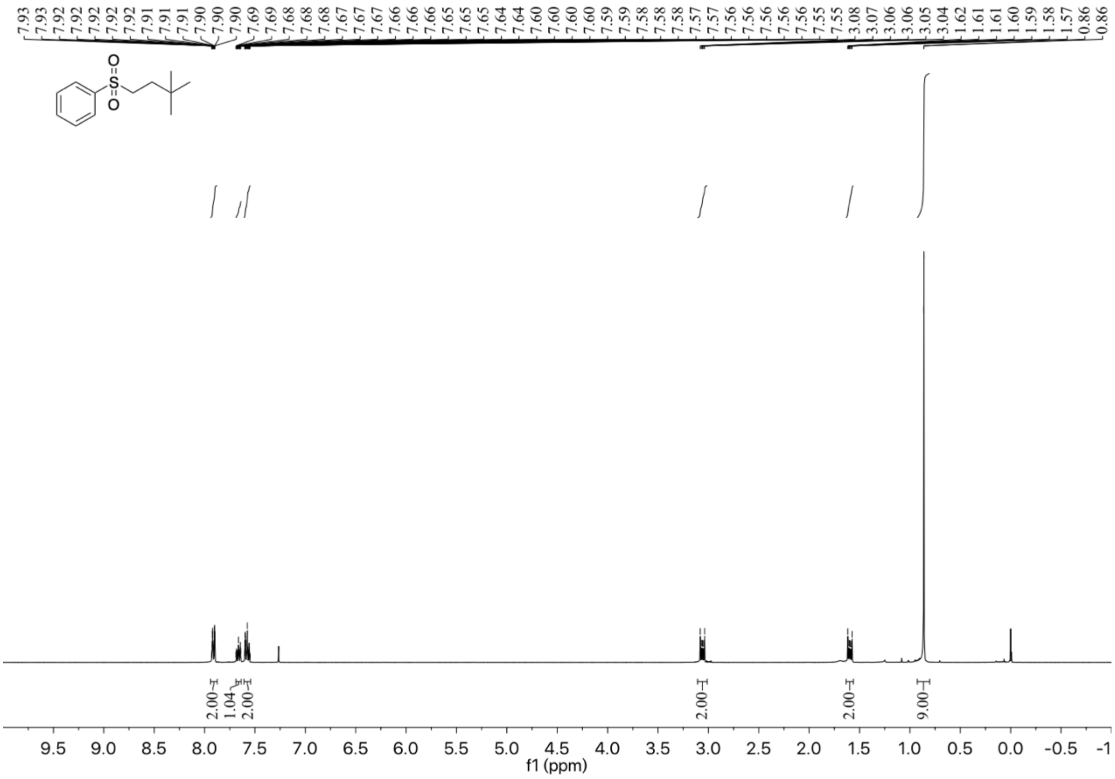

**$^{13}$C NMR (126 MHz, CDCl$_3$)** spectrum of **((3,3-dimethylbutyl)sulfonyl)benzene (62)**

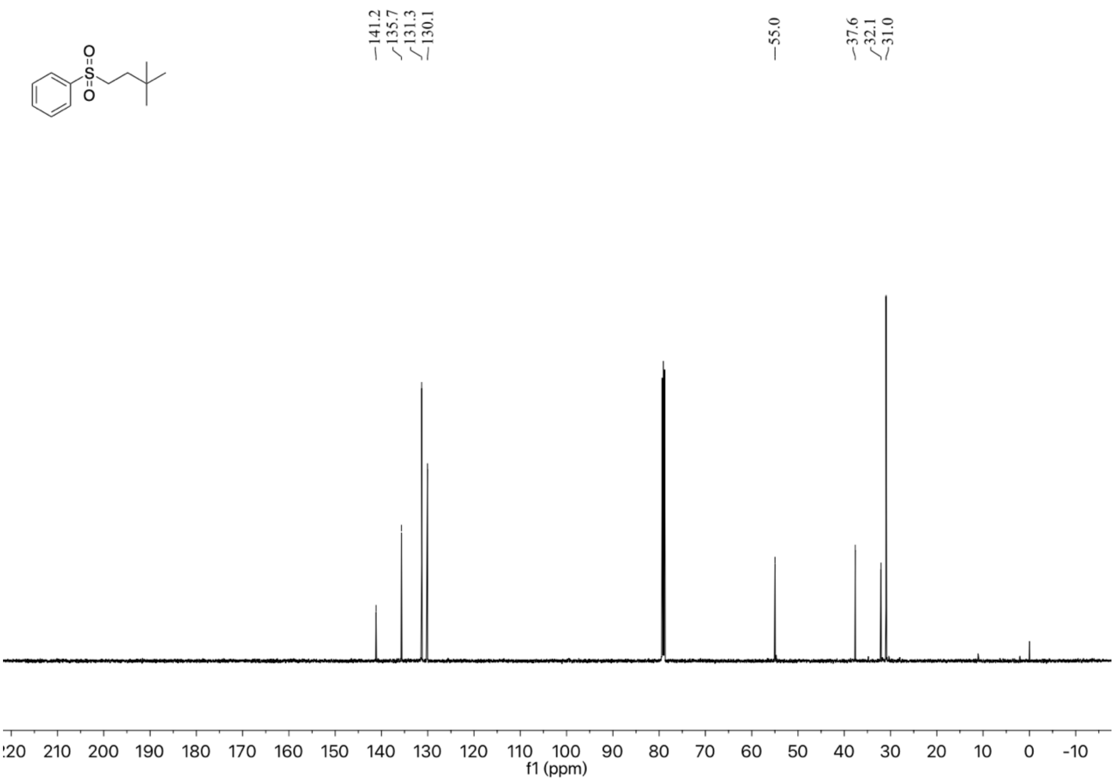

**$^1$H NMR (400 MHz, CDCl$_3$)** spectrum of **((2-(1-methylcyclohexyl)ethyl)sulfonyl)benzene (63)**

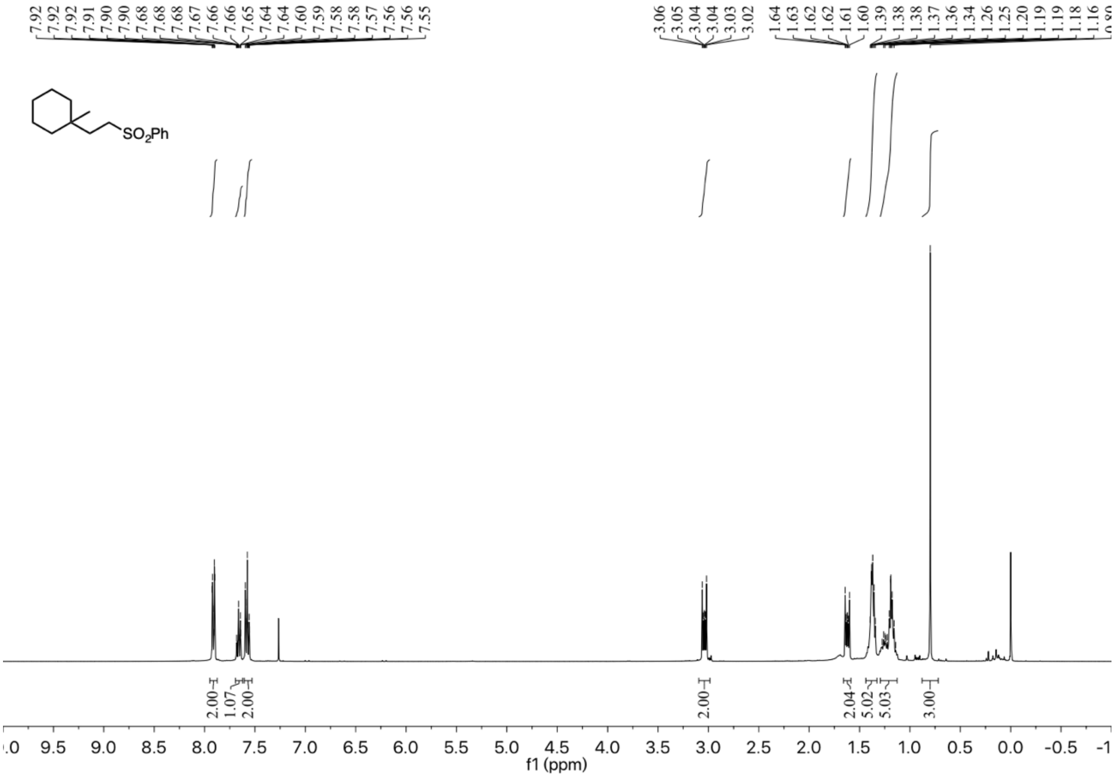

**$^{13}C$ NMR (126 MHz, $CDCl_3$)** spectrum of **((2-(1-methylcyclohexyl)ethyl)sulfonyl)benzene (63)**

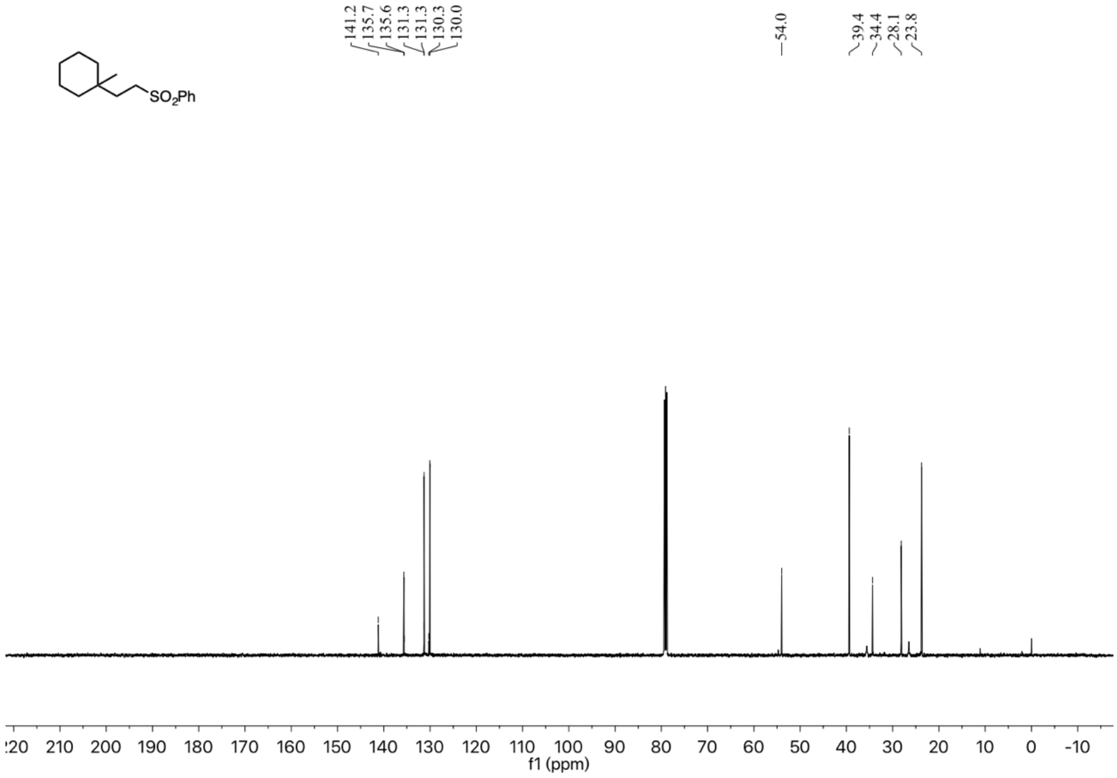

**$^{1}H$ NMR (500 MHz, $CDCl_3$)** spectrum of **((2-cyclohexylethyl)sulfonyl)benzene (64)**

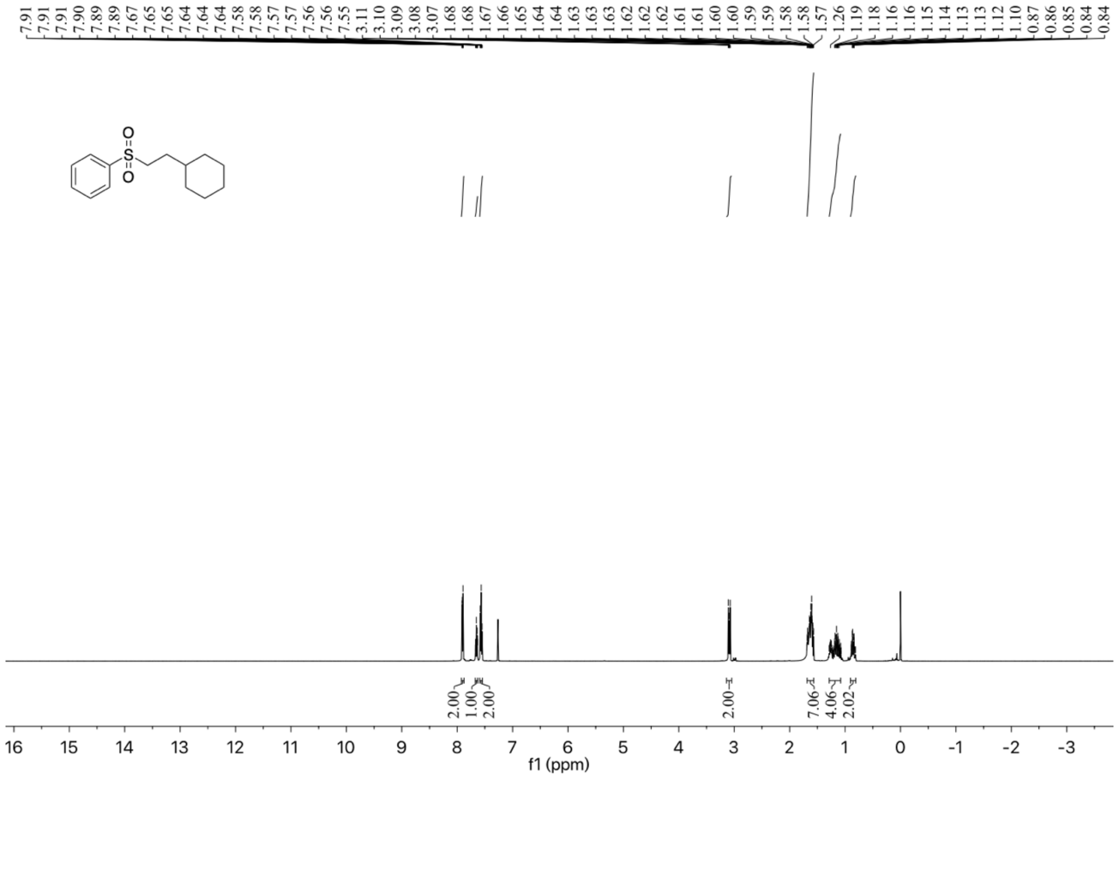

**$^{13}$C NMR (126 MHz, CDCl$_3$)** spectrum of **((2-cyclohexylethyl)sulfonyl)benzene (64)**

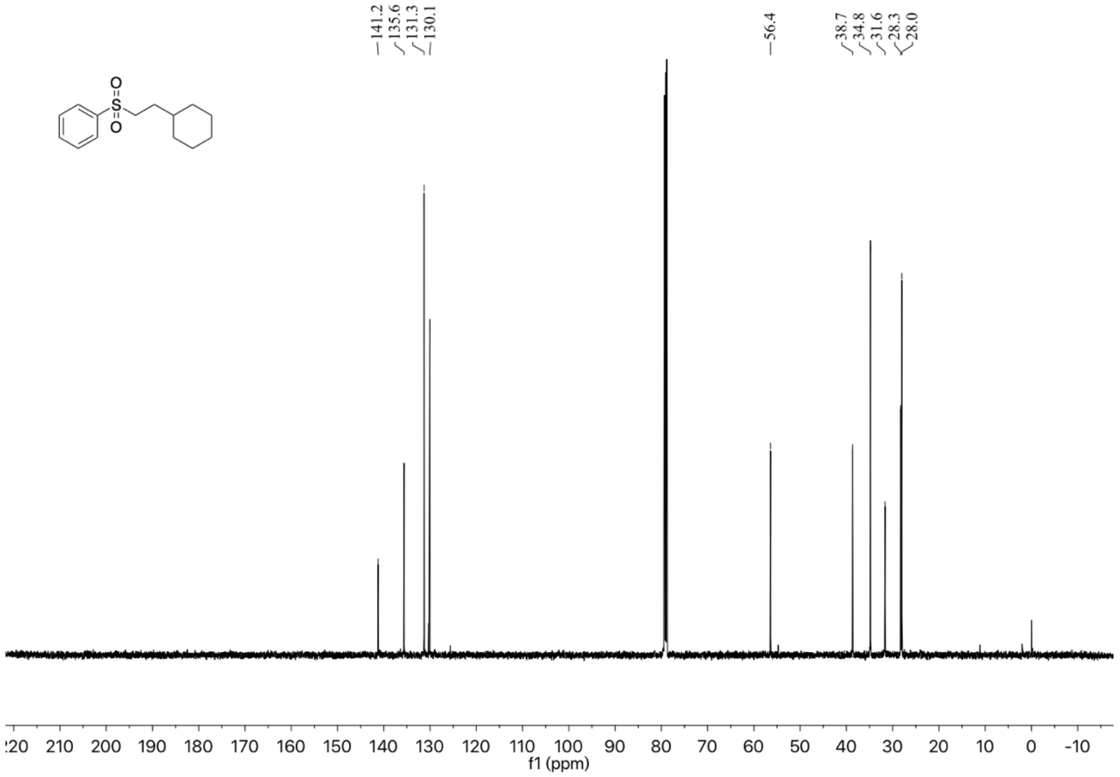


**$^{1}$H NMR (500 MHz, CDCl$_3$)** spectrum of **((4-cyclohexylbutyl)sulfonyl)benzene (65)**

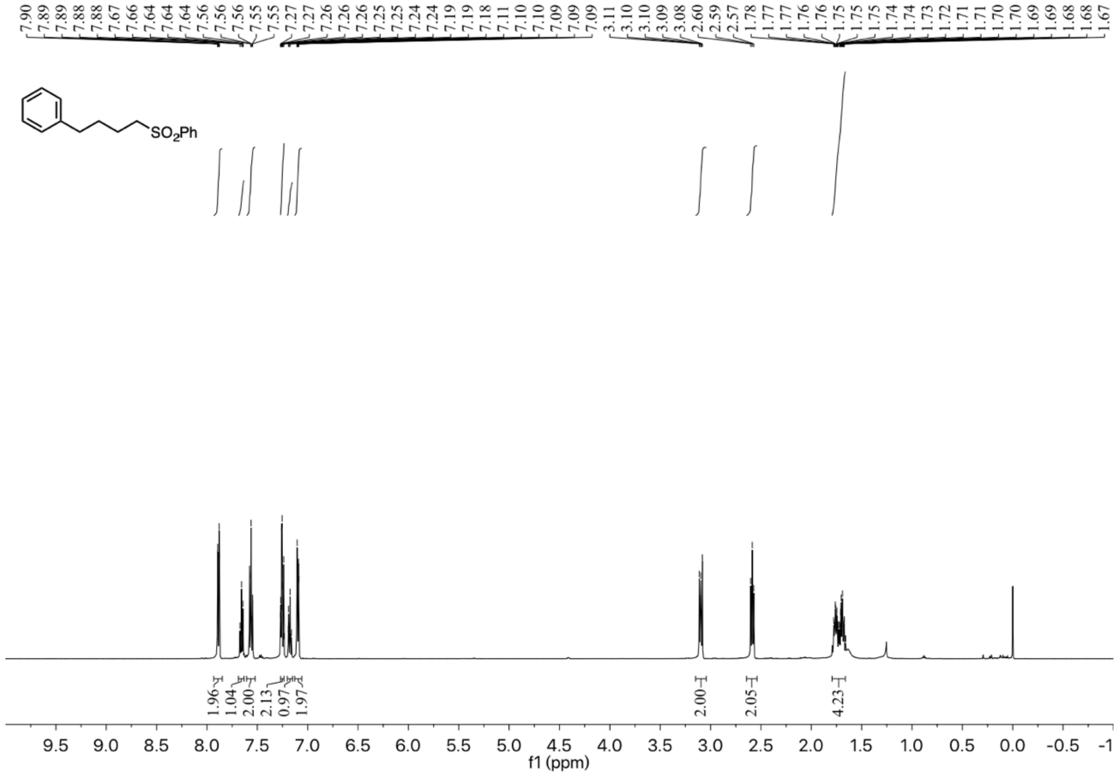

**$^{13}$C NMR (126 MHz, CDCl$_3$)** spectrum of **((4-cyclohexylbutyl)sulfonyl)benzene (65)**

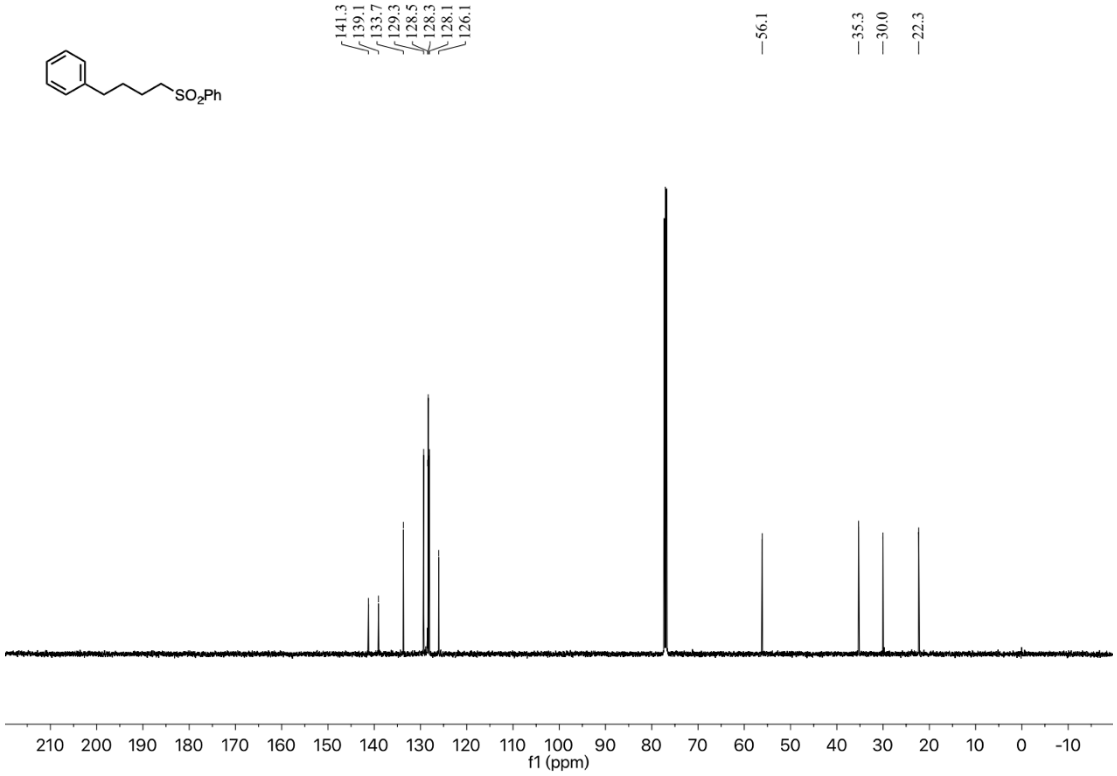

**$^{1}$H NMR (500 MHz, CDCl$_3$)** spectrum of **N-(4-(phenylsulfonyl)butan-2-yl)benzamide (66)**

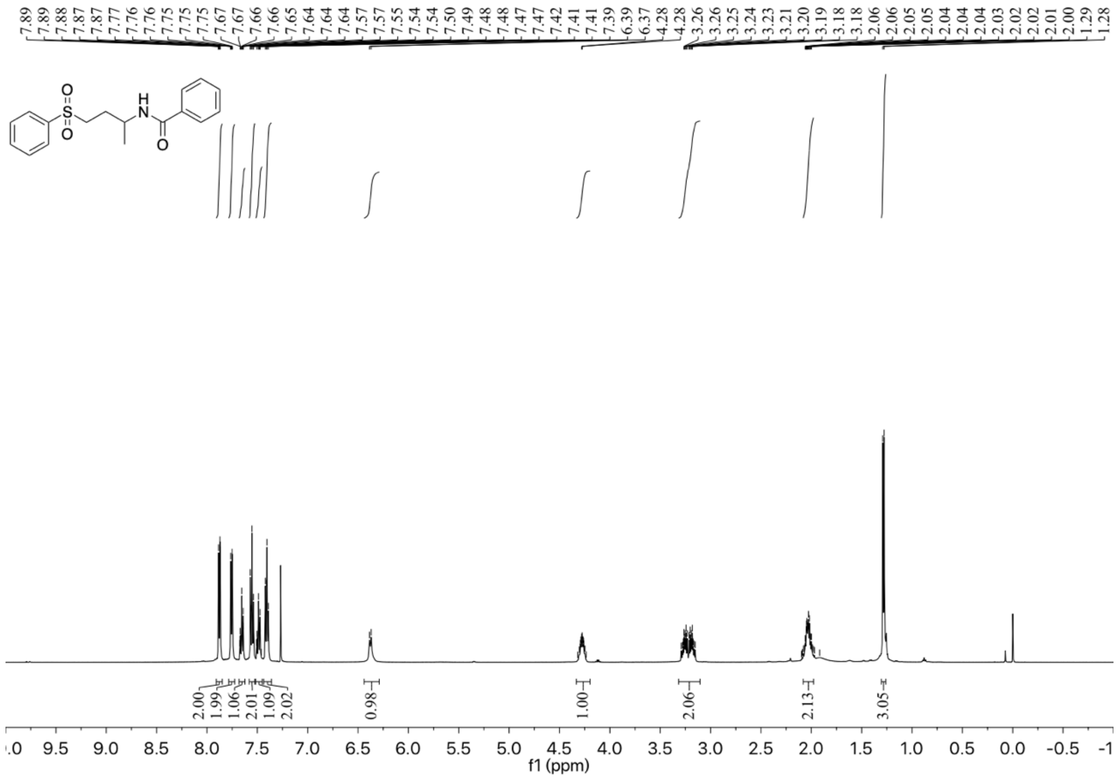

**$^{13}$C NMR (126 MHz, CDCl$_3$)** spectrum of **N-(4-(phenylsulfonyl)butan-2-yl)benzamide (66)**

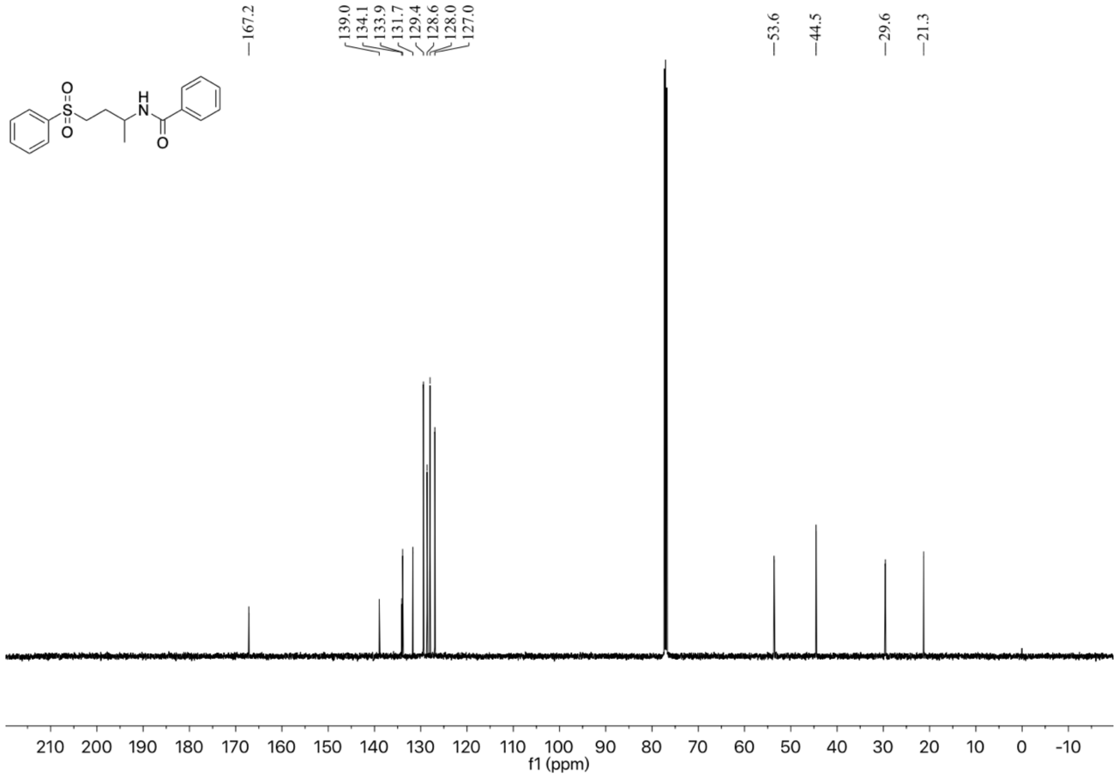


**$^{1}$H NMR (500 MHz, CDCl$_3$)** spectrum of **7-(2,5-dimethylphenoxy)-4,4-dimethylheptanenitrile (67)**

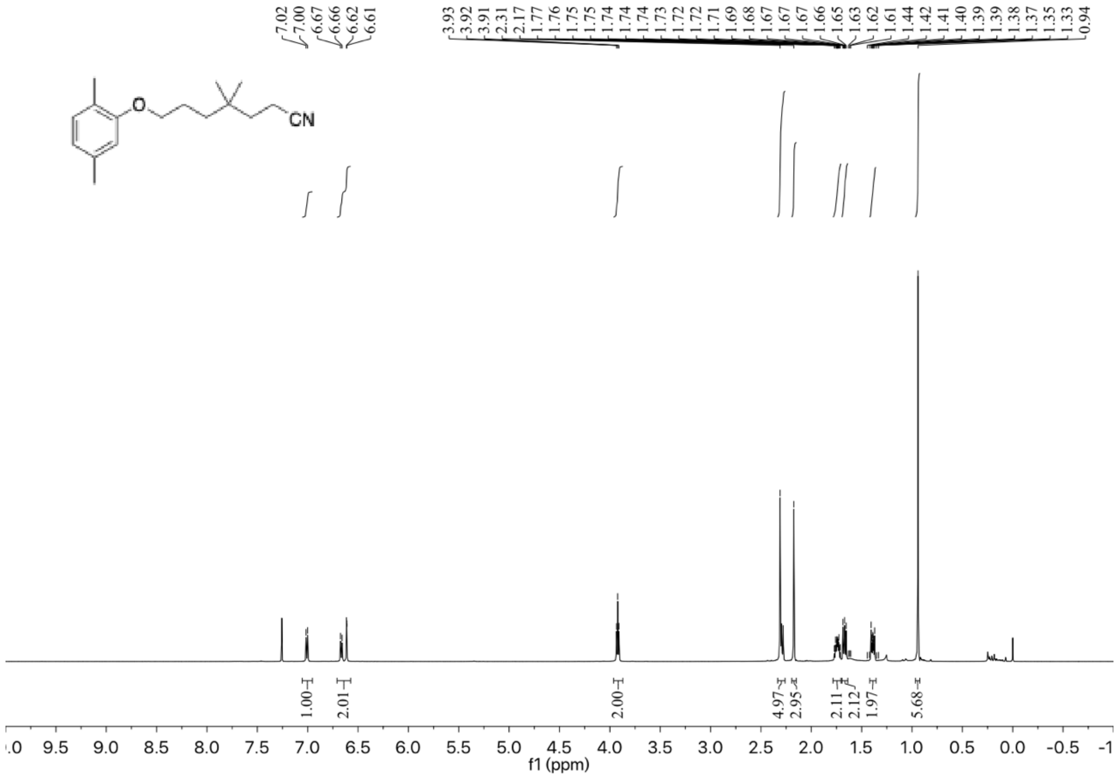

**$^{13}C$ NMR (126 MHz, $CDCl_3$)** spectrum of **7-(2,5-dimethylphenoxy)-4,4-dimethylheptanenitrile (67)**

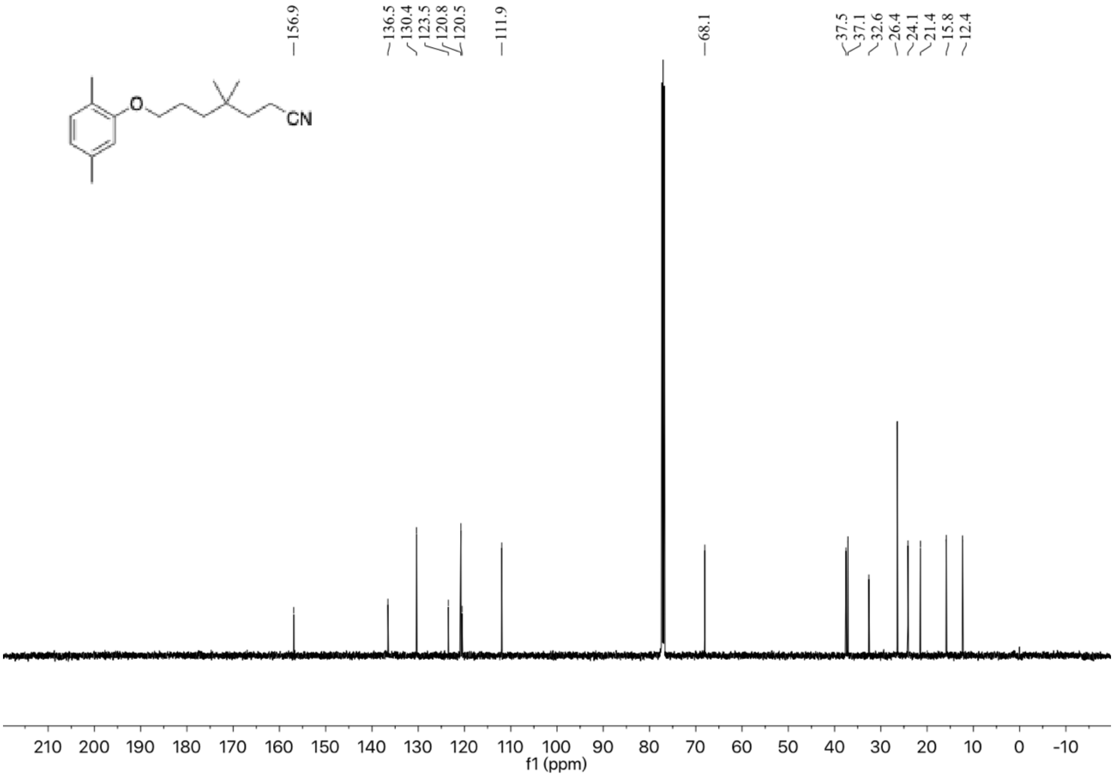


**$^{1}H$ NMR (500 MHz, $CDCl_3$)** spectrum of **diethyl (6-(2,5-dimethylphenoxy)-3,3-dimethylhexyl)phosphonate (68)**

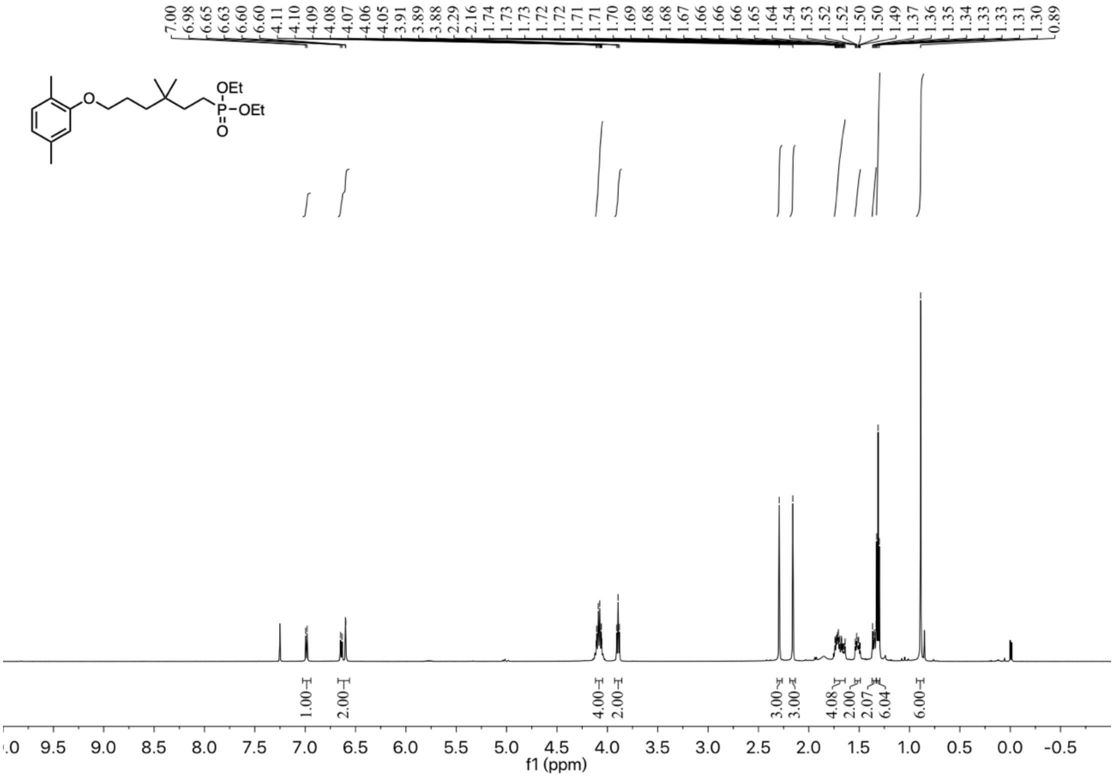

$^{13}$C NMR (126 MHz, $CDCl_3$) spectrum of **diethyl (6-(2,5-dimethylphenoxy)-3,3-dimethylhexyl)phosphonate (68)**

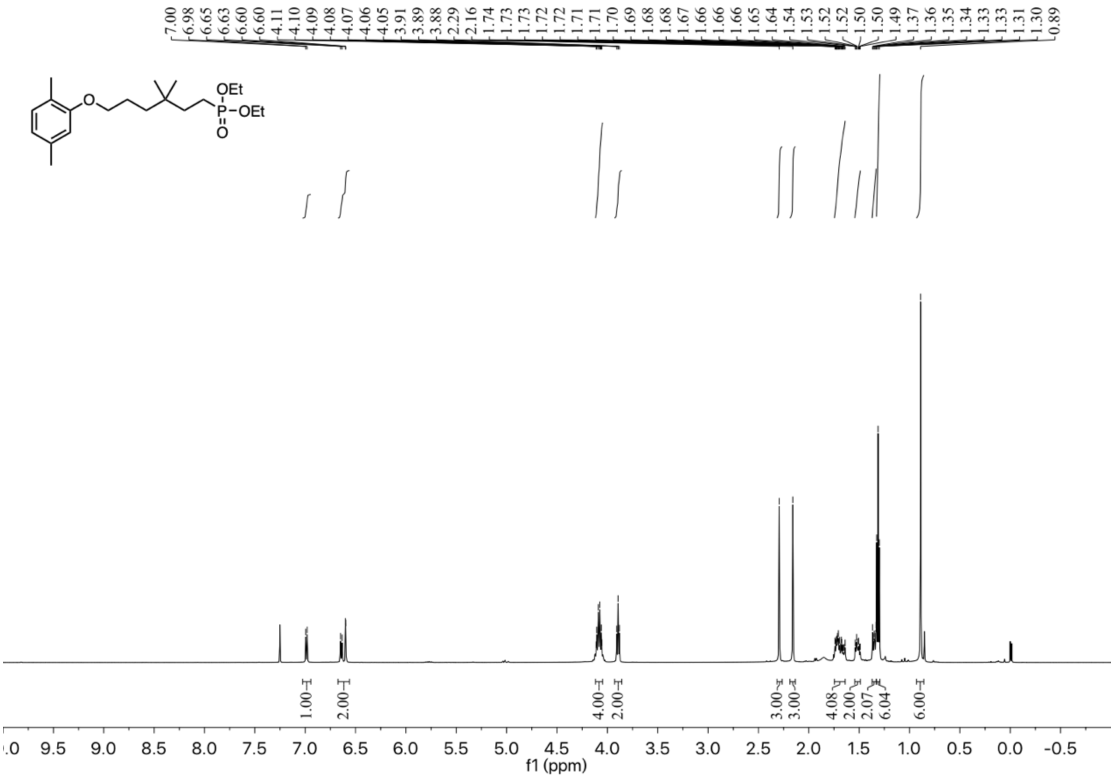


**$^{31}$P NMR (202 MHz, Chloroform-*d*)** spectrum of **((2-(1-methylcyclohexyl)ethyl)sulfonyl)benzene (68)**

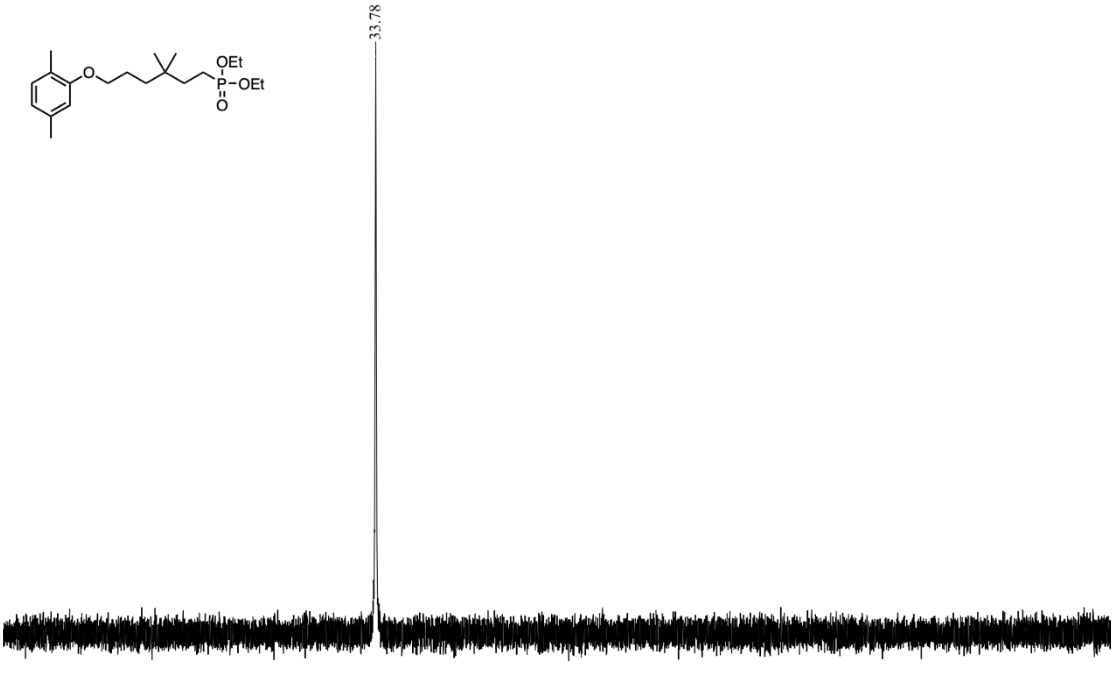

**$^{1}$H NMR (500 MHz, $CDCl_3$)** spectrum of **dimethyl 2-(5-(2,5-dimethylphenoxy)-2-methylpentan-2-yl)succinate (69)**

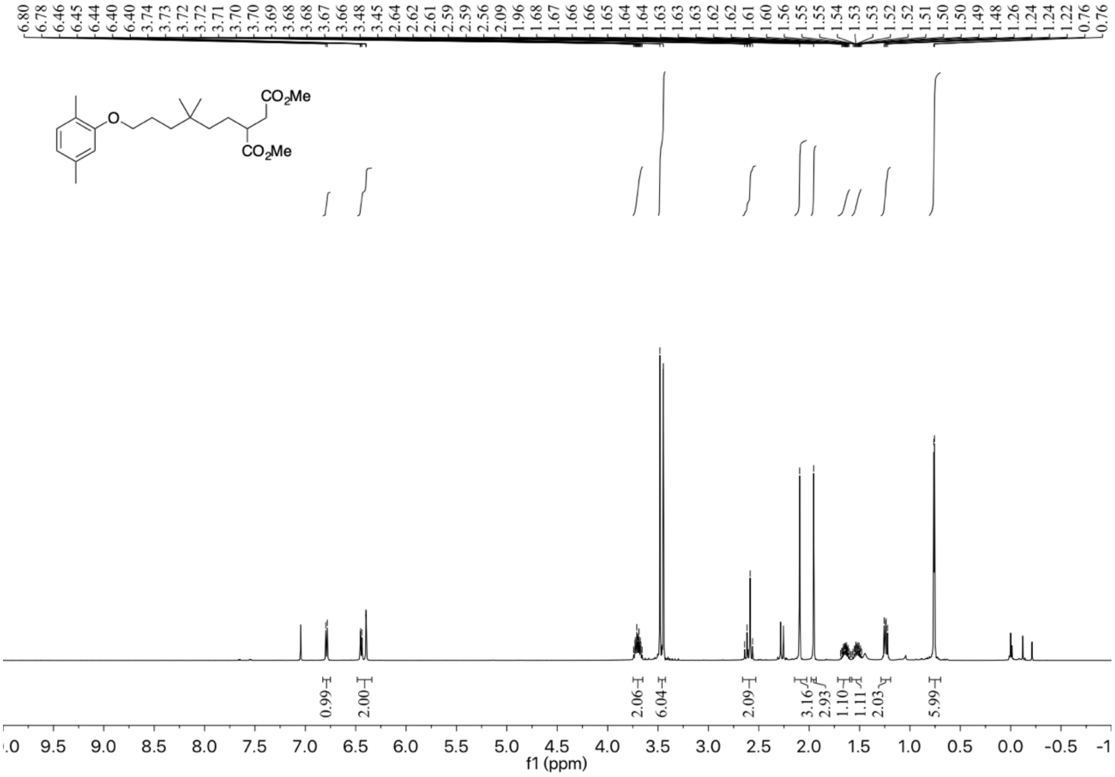


**$^{13}$C NMR (126 MHz, $CDCl_3$)** spectrum of **dimethyl 2-(5-(2,5-dimethylphenoxy)-2-methylpentan-2-yl)succinate (69)**

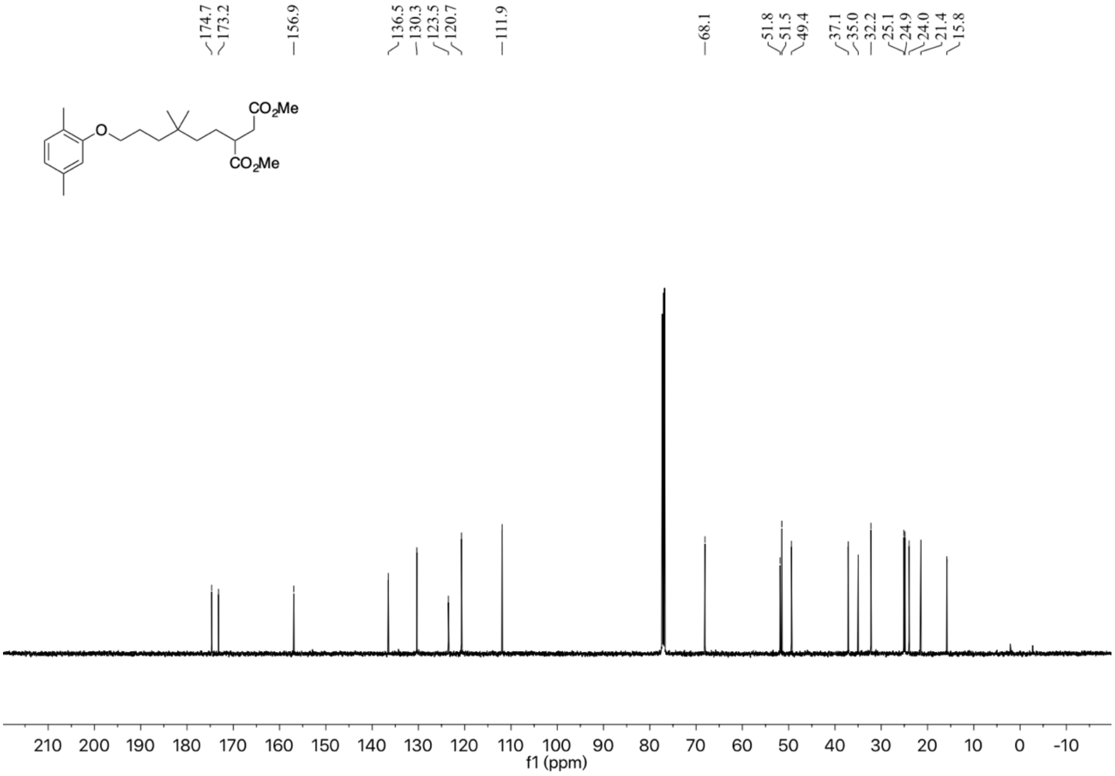

**$^{1}$H NMR (400 MHz, CDCl$_3$)** spectrum of **diethyl 2-(6-(2,5-dimethylphenoxy)-3,3-dimethylhexan-2-yl)malonate (70)**

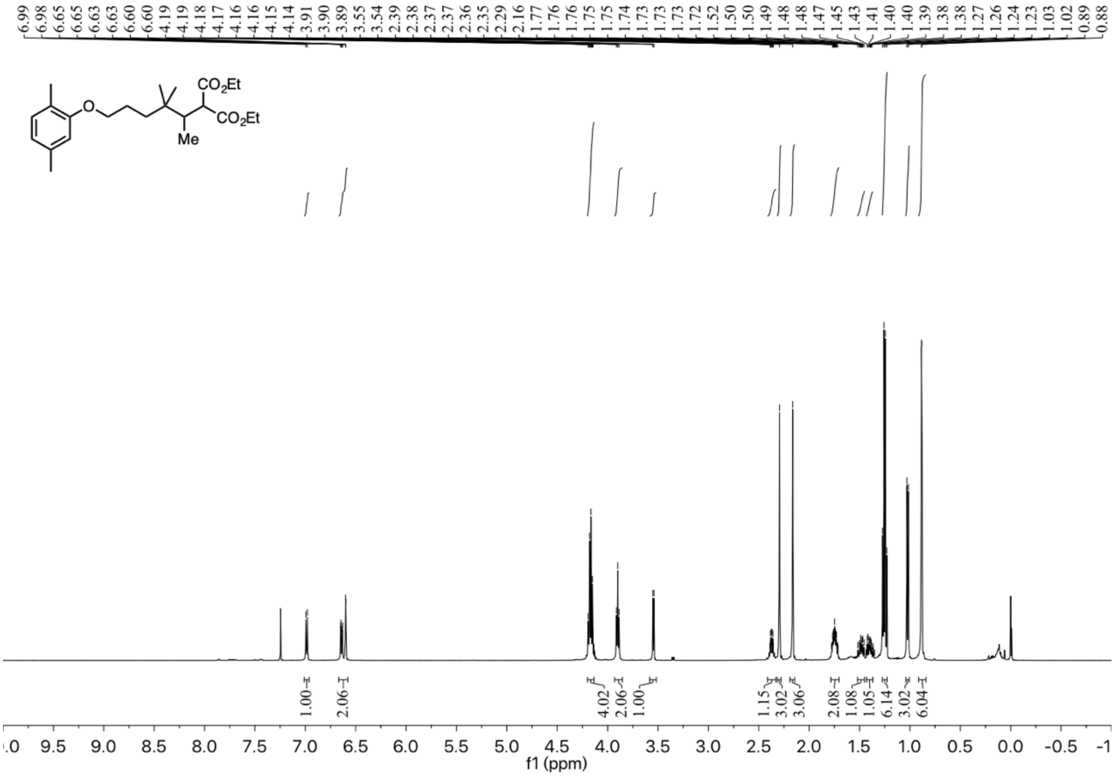

**$^{13}$C NMR (151 MHz, CDCl$_3$)** spectrum of **diethyl 2-(6-(2,5-dimethylphenoxy)-3,3-dimethylhexan-2-yl)malonate (70)**

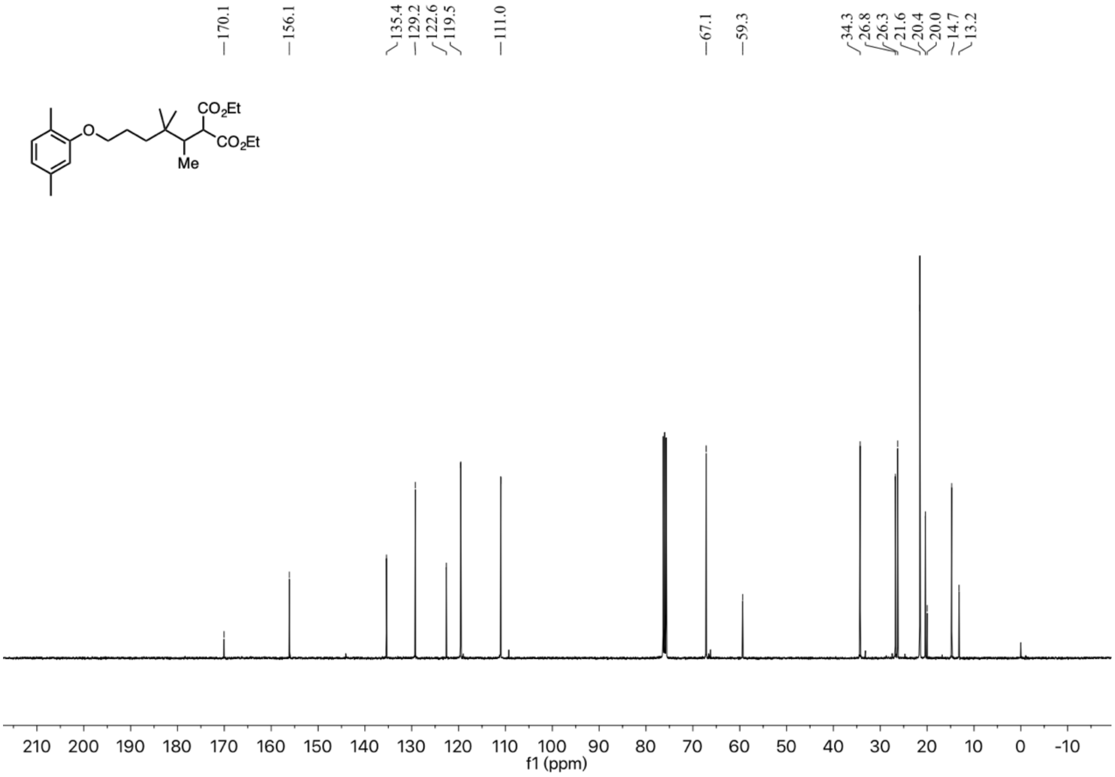

$^{1}H$ NMR (400 MHz, $CDCl_3$) spectrum of **4-(1,1-difluoro-4-methyl-4-(3-methylcyclopentyl)pent-1-en-2-yl)-1,1'-biphenyl (73)**

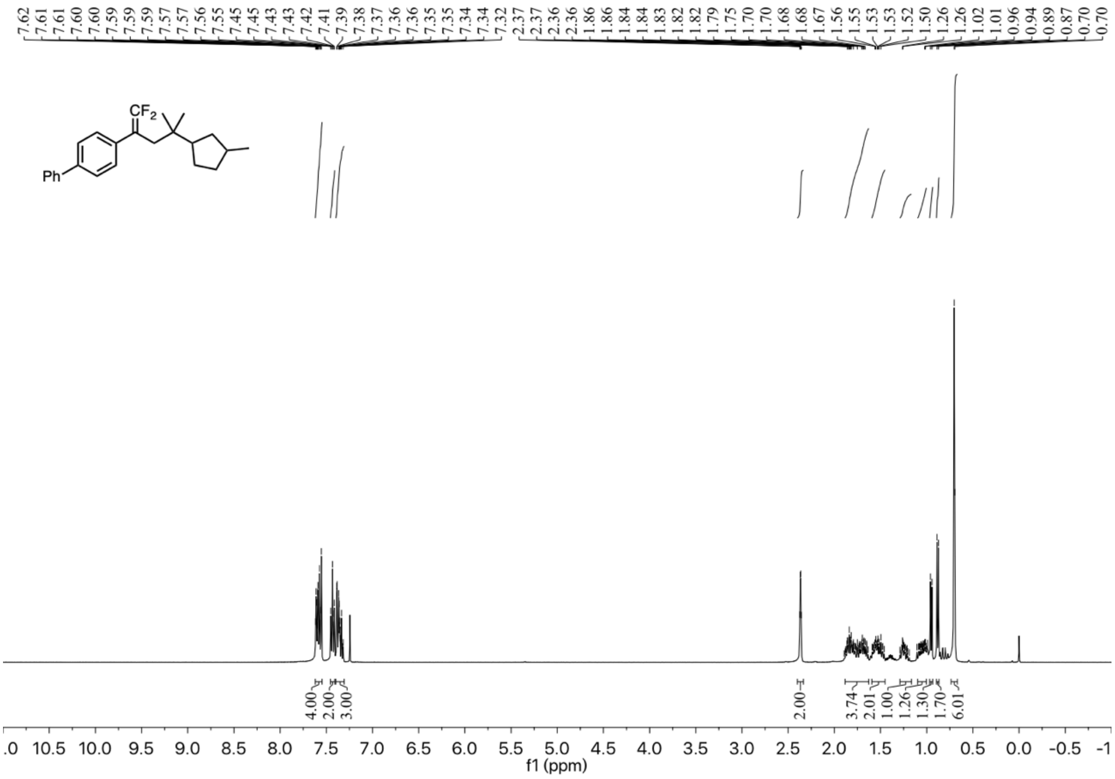


$^{13}C$ NMR (101 MHz, $CDCl_3$) spectrum of **4-(1,1-difluoro-4-methyl-4-(3-methylcyclopentyl)pent-1-en-2-yl)-1,1'-biphenyl (73)**

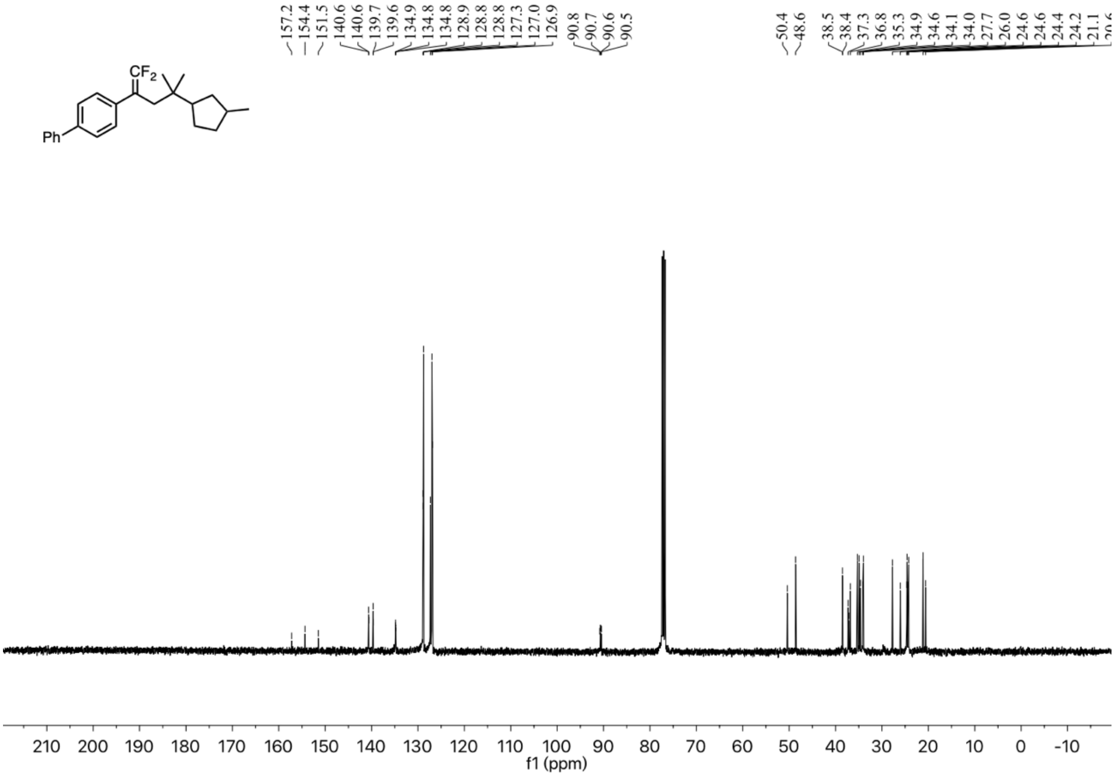

**$^{19}$F NMR (376 MHz, CDCl$_3$)** spectrum of **4-(1,1-difluoro-4-methyl-4-(3-methylcyclopentyl)pent-1-en-2-yl)-1,1'-biphenyl (73)**

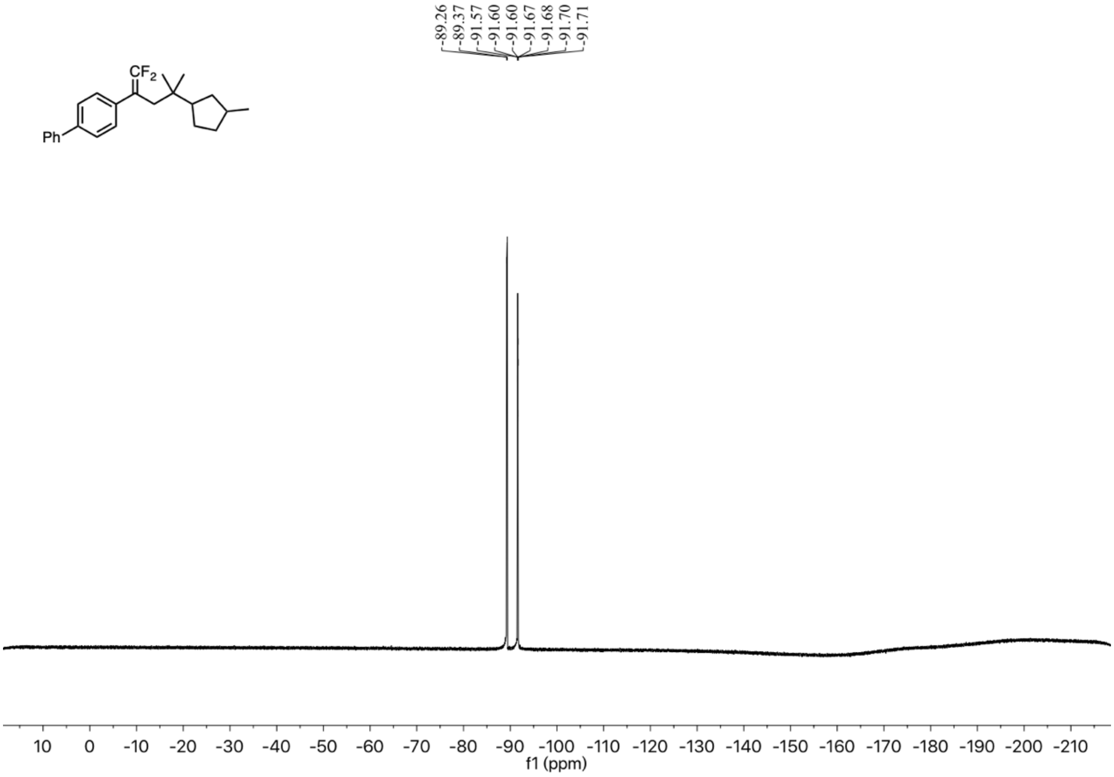


**$^{1}$H NMR (400 MHz, CDCl$_3$)** spectrum of **2-(1,1,1,3,3,3-hexamethyl-2-(trimethylsilyl)trisilan-2-yl)isoindoline-1,3-dione (75)**

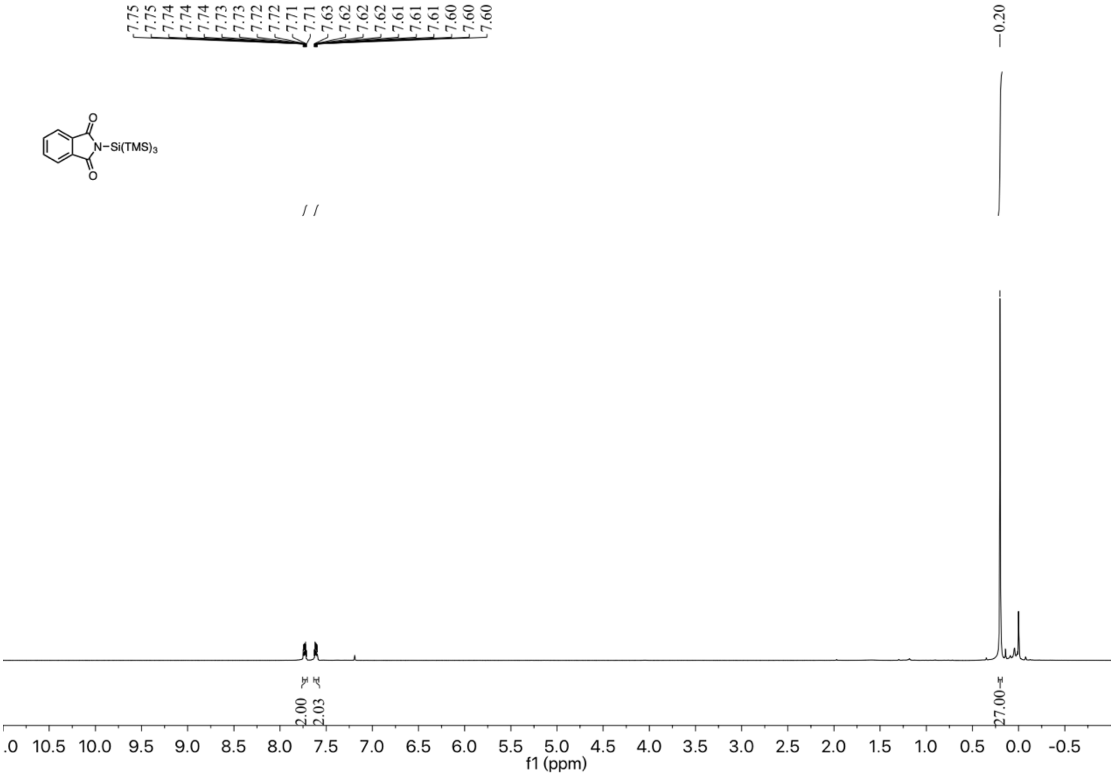

**$^{13}$C NMR (101 MHz, $CDCl_3$)** spectrum of **2-(1,1,1,3,3,3-hexamethyl-2-(trimethylsilyl)trisilan-2-yl)isoindoline-1,3-dione (75)**

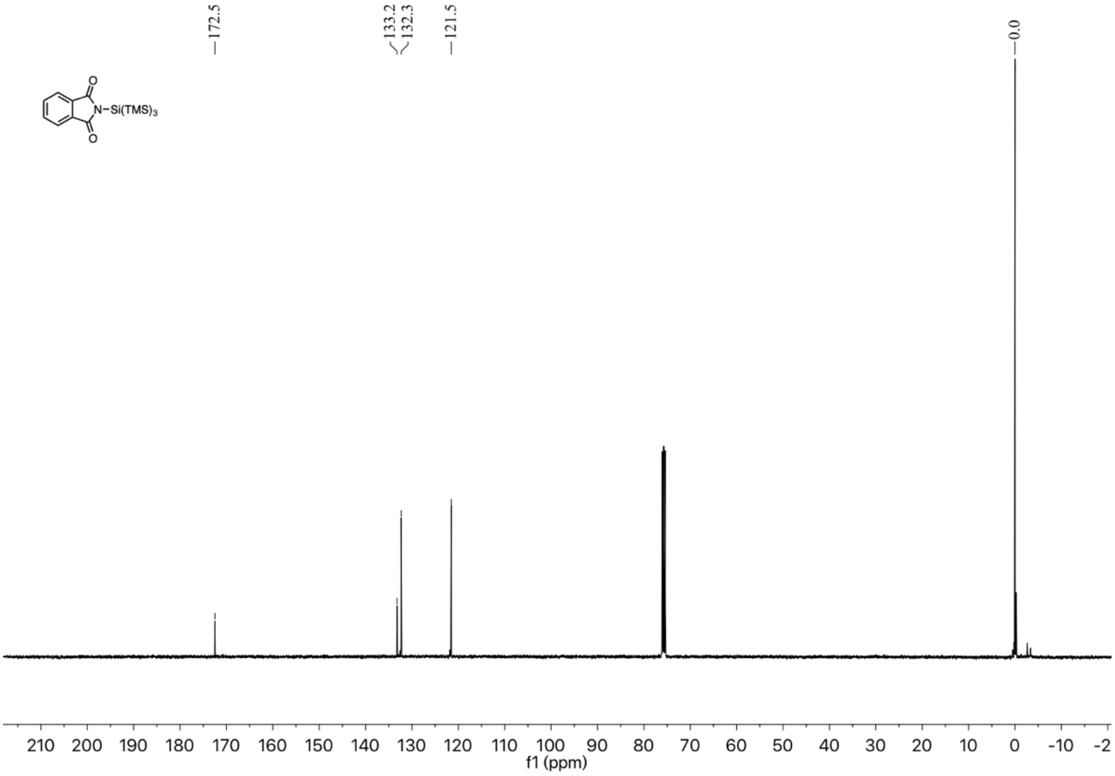

**$^{1}$H NMR (400 MHz, $CDCl_3$)** spectrum of **triphenylphosphine oxide (76)**

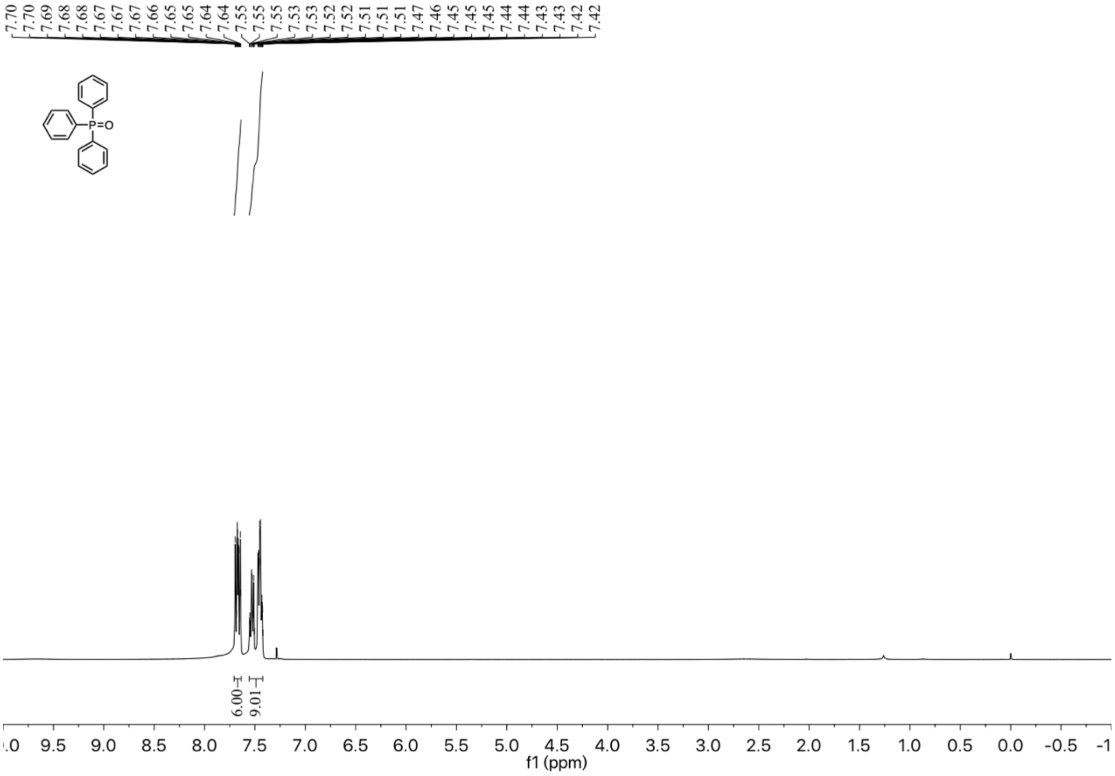

<sup></sup>

**$^{13}$C NMR (101 MHz, $CDCl_3$)** spectrum of **triphenylphosphine oxide (76)**

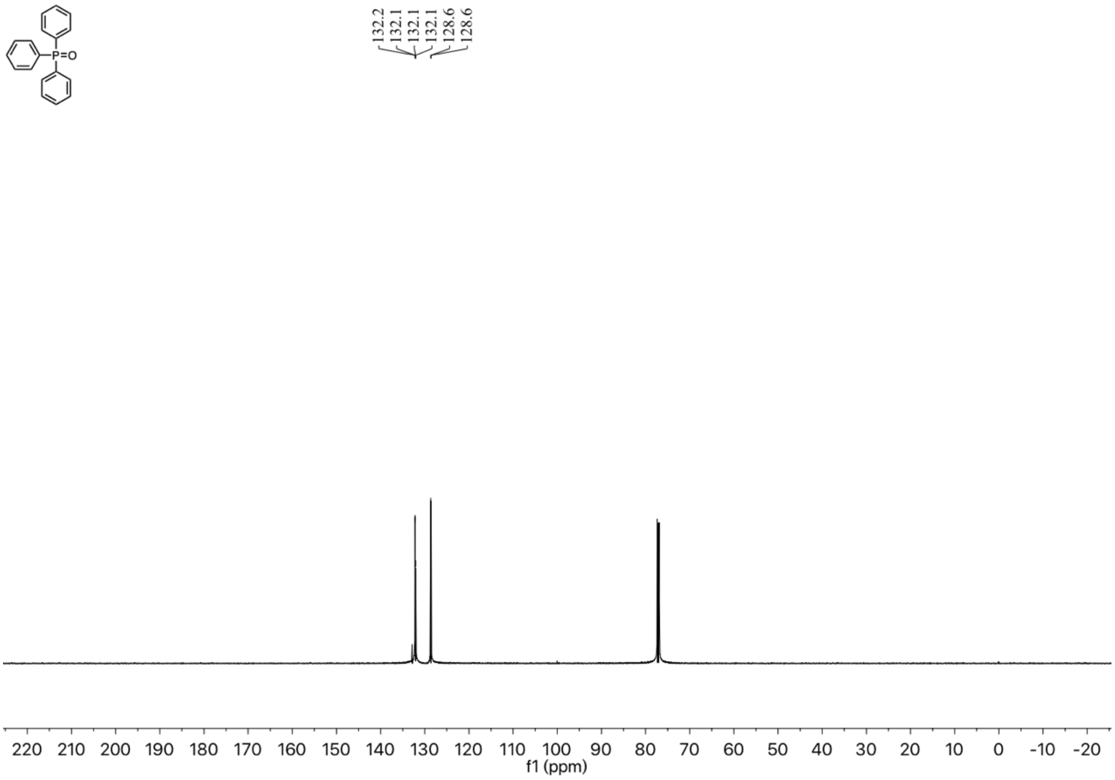


**$^{31}$P NMR (162 MHz, $CDCl_3$)** spectrum of **triphenylphosphine oxide (76)**

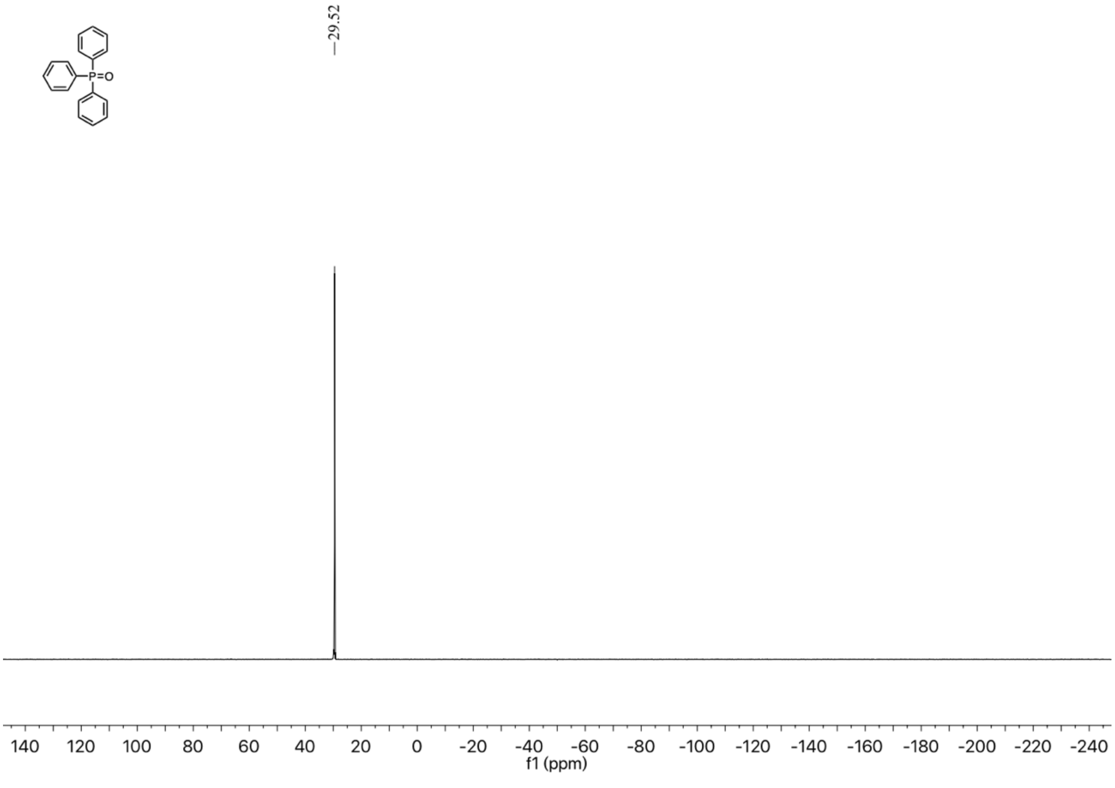

Supplement: Supplementary file 1 — Supporting Information [file ADVS-11-2307241-s001.pdf]
